# Synthesis of tertiary alkyl fluorides and chlorides by site-selective nucleophilic ring-opening reaction of α-aryl azetidinium salts†

**DOI:** 10.1039/d1ra08706a

**Published:** 2021-12-13

**Authors:** Eiji Tayama, Kohei Kawai

**Affiliations:** Department of Chemistry, Faculty of Science, Niigata University Niigata 950-2181 Japan tayama@chem.sc.niigata-u.ac.jp; Graduate School of Science and Technology, Niigata University Niigata 950-2181 Japan

## Abstract

Site-selective nucleophilic ring-opening reactions of 2-arylazetidine-2-carboxylic acid ester-derived tetraalkyl ammonium salts 2 with tetrabutylammonium halides (Bu_4_NX) to give tertiary alkyl halides are successfully demonstrated. For example, a nucleophilic ring-opening reaction of 2-(*o*-tolyl) derivative 2a with 1.2 equivalents of tetrabutylammonium fluoride (Bu_4_NF) in THF at 60 °C preferentially proceeded at a more substituted carbon atom (2-position) compared to a less-substituted carbon atom (4-position) and afforded *tert*-butyl 4-(dimethylamino)-2-fluoro-2-(*o*-tolyl)butanoate 3aa in 71% yield as the corresponding tertiary alkyl fluoride. This result was applied to synthesize optically active organofluorine compounds starting from commercially available (*R*)-1-phenylethylamine.

## Abstract 2

Site-selective nucleophilic ring-opening of 2 with Bu_4_NX proceeded at a much-substituted 2-position preferentially and produced tertiary alkyl halides 3.

## Graphical abstract

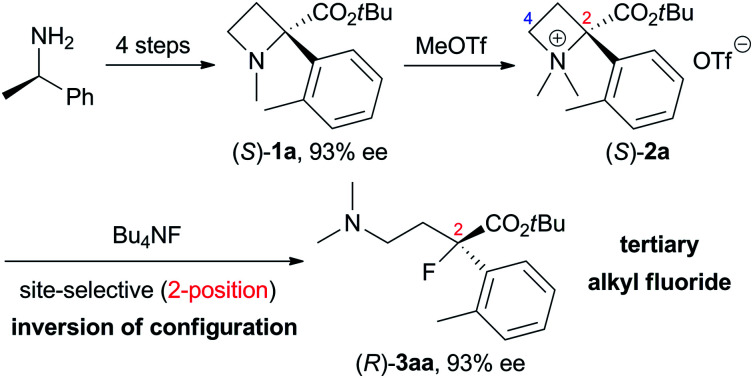

## Introduction

**Scheme 1:**
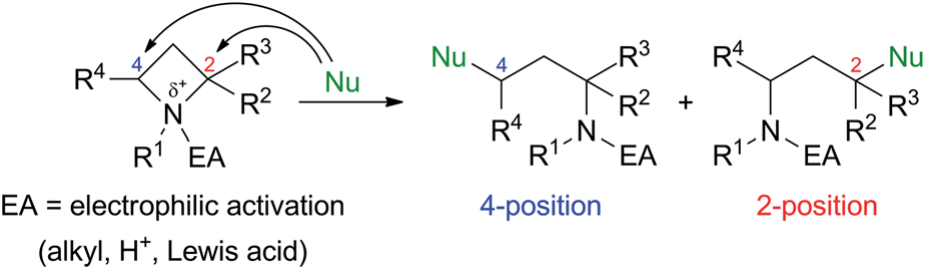
Nucleophilic ring-opening of azetidine derivatives.

Ring-strained four-membered N-heterocycle azetidines are valuable building blocks in organic synthesis. Although they are chemically stable without any additives, nucleophilic ring-opening reactions proceed to give various types of functionalized nitrogen-containing compounds by electrophilic activation of the nitrogen atom by *N*-quaternization,^1,2^ or addition of Brønsted acid (H^+^)^3^ or Lewis acids^4^ (Scheme 1).^5^ These transformations are applicable for the synthesis of amino acids, alkaloids, and biologically active drugs.

**Scheme 2:**
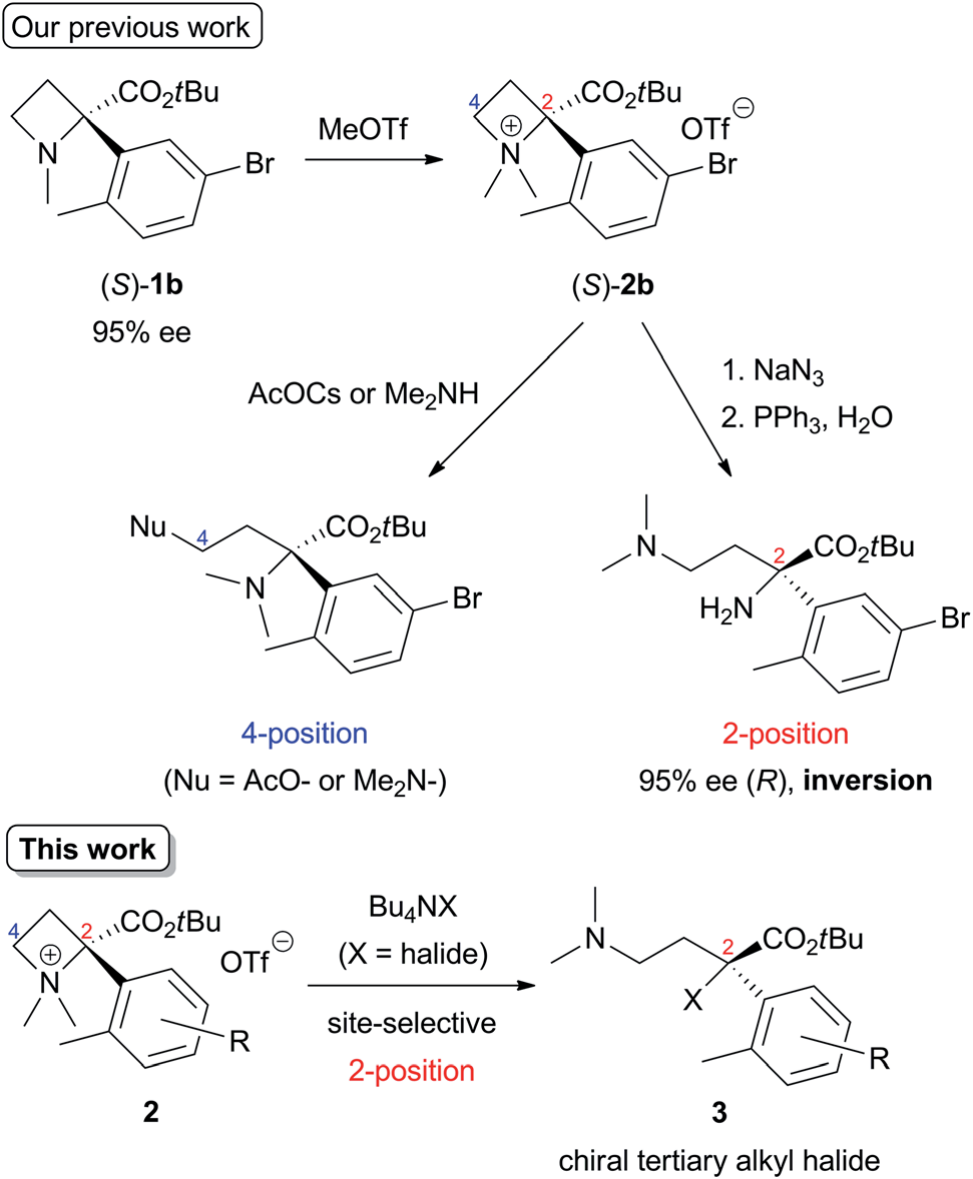
Nucleophilic ring-opening of α-arylazetidine-2-carboxylic acid ester-derived tetraalkylammonium salts 2.

The initial studies of this ring-opening reaction were mainly performed by Couty's group using tetraalkylazetidinium salts as substrates.^1^ One point to consider in this reaction is site-selectivity at the 2- and 4-positions, which reacts with a nucleophile (Nu). In many cases, a less-substituted and/or electron-deficient carbon atom is attacked by a nucleophile because of the S_N_2 process. For example, a reaction of a substrate with a nucleophile in Scheme 1 proceeded at the 4-position preferentially to afford the corresponding product. However, some nucleophiles do not act according to this tendency, and the reaction occurs at the 2-position, which is a much-substituted carbon atom. Although these phenomena are currently difficult to explain, the site selectivity at the 2- and 4-positions can be determined based on the properties of nucleophiles, substituents at the 2- and 4-positions, and reaction conditions.^1*d*,*g*,*h*^ Previously, our group reported that the site-selective nucleophilic ring-opening reaction of α-arylazetidine-2-carboxylic acid ester-derived tetraalkylammonium salt (*S*)-2b prepared from 95% ee of (*S*)-1b (Scheme 2, Our previous work).^6^ Cesium acetate (AcOCs) and dimethylamine (Me_2_NH) as nucleophiles reacted at the 4-position. In contrast, sodium azide (NaN_3_) reacted at the 2-position with inversion of the configuration. This result shows that the S_N_2 substitution at the tertiary carbon atom (2-position) proceeded.^7^ With the results, our group started to further investigate the scope of this reaction, since some nucleophiles such as fluoride (F^−^) provide valuable compounds. Furthermore, previous examples of the ring-opening reaction of azetidine derivatives with F^−^ to give organofluorine compounds are rare^2*a*,8^ compared to the reaction of three-membered N-heterocycle aziridine derivatives.^9^ Herein, we wish to report the site-selective nucleophilic ring-opening reaction of α-aryl azetidinium salts 2 with halides to afford α-aryl-α-halo-carboxylic acid esters 3 (Scheme 2, this work). Further synthetic applications of the resulting products 3, *e.g.*, asymmetric synthesis of organofluorine compounds, are also demonstrated.

## Results and discussion

We started investigating the nucleophilic ring-opening reaction of 2a with a halide source (Table 1). First, the reaction of 2a with sodium fluoride (NaF) as an F^−^ source in DMF at room temperature for 2 h was examined to obtain the corresponding organofluorine compounds 3aa and 4aa; however, no products were obtained (entry 1). Although a reaction with potassium fluoride (KF) gave the same result (entry 2), the use of cesium fluoride (CsF) afforded 3aa in 13% yield (entry 3). We expected that tetrabutylammonium fluoride (Bu_4_NF) might be more reactive, and its solubility in organic solvents would improve the yields of 3aa and 4aa. In addition, Ghorai *et al.* reported the Lewis acid-promoted nucleophilic ring-opening reaction of *N*-tosylazetidines with tetrabutylammonium chloride (Bu_4_NCl) and bromide (Bu_4_NBr).^10^ Thus, we attempted a reaction with a THF solution of Bu_4_NF, and the desired 3aa was obtained in 33% yield with trace amounts of 4aa (<4% yield) (entry 4). The use of THF as a solvent and other F^−^ sources, such as Bu_4_NF·3H_2_O, did not show any improvements (entries 5 and 6). We found that the yield of 3aa could be improved to 71% with minimization of the formation of 4aa (7% yield) when the reaction was performed at 60 °C (entry 7).

**Table 1:** Nucleophilic ring-opening of α-aryl azetidinium salt 2a with various salts

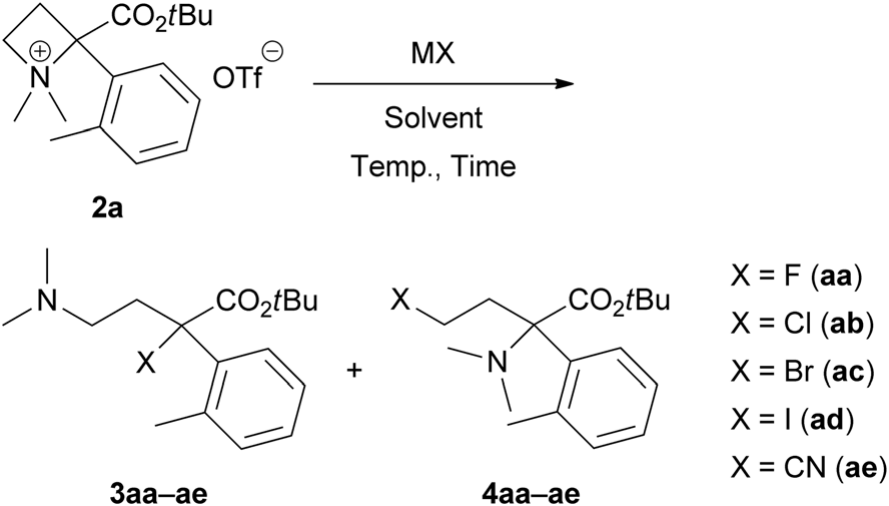					
Entry	MX (equiv.)	Solvent	Temp., time	3^a^ (%)	4^a^ (%)
1	NaF (5)	DMF	rt, 2 h	0	0
2	KF (5)	DMF	rt, 2 h	0	0
3	CsF (5)	DMF	rt, 2 h	13	0
4	Bu_4_NF in THF (1.2)	DMF	rt, 2 h	33	<4
5	Bu_4_NF in THF (1.2)	THF	rt, 2 h	35	2
6	Bu_4_NF·3H_2_O (1.2)	THF	rt, 2 h	41	<3
7	Bu_4_NF in THF (1.2)	THF	60 °C, 1 h	71	7
8	Bu_4_NCl (1.2)	DMF	rt, 2 h	74	14
9	Bu_4_NCl (1.2)	THF	rt, 2 h	76	23
10	Bu_4_NCl (1.2)	CH_2_Cl_2_	rt, 2 h	69	14
11	Bu_4_NCl (1.2)	THF	0 °C, 2 h	34	10
12	Bu_4_NCl (1.2)	THF	60 °C, 2 h	70	27
13	Bu_4_NBr (1.2)	THF	rt, 1 h	61	21
14	Bu_4_NI (1.2)	THF	rt, 1 h	0	0
15	KCN (5)	DMF	rt, 2 h	42	55
16	Bu_4_NCN (1.2)	THF	rt, 2 h	38	62

a Isolated yield.

Next, we examined the same reaction with other tetrabutylammonium salts (Bu_4_NX) to define the scope of this site-selective ring-opening reaction. Reactions with Bu_4_NCl in THF, DMF and CH_2_Cl_2_ proceeded even at room temperature, and similar yields of 3ab (69–76% yields) and 4ab (14–23% yields) were observed (entries 8–10). At 0 °C, the yields of 3ab (34% yield) and 4ab (10% yield) decreased (entry 11). When the reaction was performed at 60 °C, the yield of undesired 4ab was slightly improved (27% yield) (entry 12). The use of Bu_4_NBr is also applicable; however, the selectivity between 3ac (61% yield) and 4ac (21% yield) was insufficient (entry 13). Additionally, the resulting isolated bromo products 3ac and 4ac were unstable because of the self-*N*-quaternization. Therefore, a reaction with tetrabutylammonium iodide (Bu_4_NI) did not give 3ad and 4ad (entry 14). Finally, we applied this reaction for pseudohalogen salts (MCN) to provide α-cyano derivative 3ae with an all-carbon quaternary stereocentre (entries 15 and 16). Unfortunately, both reactions with potassium cyanide (KCN) and tetrabutylammonium cyanide (Bu_4_NCN) gave similar results to provide 3ae and 4ae without selectivities.^11^

**Fig. 1:**
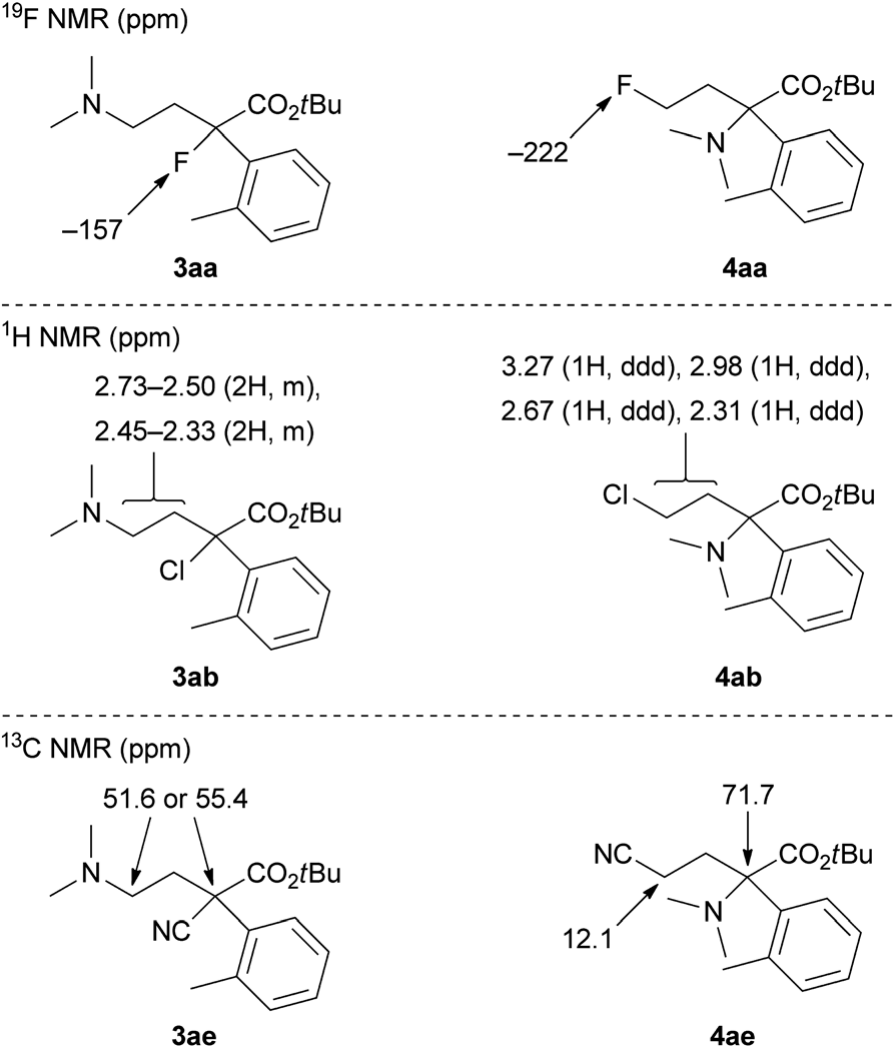
Representative NMR chemical shifts for product assignments of 3 and 4.

The ring-opening products 3 and 4 in Table 1 were assigned by NMR analyses, and their representative results are shown in Fig. 1. Fluorine derivatives 3aa and 4aa were clearly identified by the ^19^F NMR analysis. Tertiary alkyl fluoride 3aa showed a chemical shift of −157 ppm. Primary alkyl fluoride 4aa showed a chemical shift of −222 ppm. These values are reasonable for the corresponding alkyl fluorides. In contrast, chlorine derivatives 3ab and 4ab did not show clear differences in ^1^H and ^13^C NMR analyses. Consequently, we assigned these by comparison of ^1^H NMR chemical shifts of methylene protons. Primary alkyl chloride 4ab had low-field chemical shifts due to an electron-withdrawing effect of chloride. One of the two products (3ab or 4ab) with chemical shifts of 3.27 and 2.98 ppm was assigned to 4ab. Another product was assigned to tertiary alkyl chloride 3ab, which showed chemical shifts of 2.73–2.33 ppm. Bromine derivatives 3ac and 4ac were assigned by analogy to 3ab and 4ab. Meanwhile, nitrile derivatives 3ae and 4ae could be clearly identified by ^13^C NMR analysis. 4ae showed a chemical shift of 12.1 ppm, which is a reasonable value as a primary nitrile.^1*d*^

To define the scope and limitations of this site-selective ring-opening reaction to produce tertiary alkyl halides 3, we prepared various azetidinium salts 2b–h and examined their reactions with Bu_4_NF or Bu_4_NCl under identical conditions (Table 2). First, we attempted the reactions of 5-substituted aryl derivatives 2a–e with Bu_4_NF and obtained the corresponding organofluorine compounds 3ba–ea in moderate yields (entries 1–4). The minor products 4 were not isolated (N.D.), although their formations were observed by TLC analysis. The pure products of these organofluorine 4 for spectroscopic characterizations were difficult to isolate because of small amounts (*ca.* 5% yield). Electron-withdrawing substituents on the α-aryl substituent, such as bromo (2b) and trifluoromethyl (2c), might be desirable to yield 3 (entries 1 and 2, approximately 75%). Reactions of methyl (2d) and methoxy (2e) derivatives resulted in lower yields of 3 (entries 3 and 4, approximately 60%). Thus, we next examined the reactions of 4-bromo (2f) and 4-trifluoromethyl (2g) derivatives and obtained 3fa–ga in approximately 70% yields (entries 5 and 6). However, the reaction of 3-bromo derivative 2h was resulted in a 58% yield of 3ha (entry 7). The use of Bu_4_NCl for the reactions of 2b, 2d, 2f, and 2h provided the corresponding organochlorine compounds 3bb–hb (entries 8–11) with a similar tendency to the reaction with Bu_4_NF. In these cases, the minor products 4bb–hb could be isolated as a pure form to perform their spectroscopic characterizations.

**Table 2:** Substrate scope of the site-selective ring-opening of 2 with Bu_4_NX

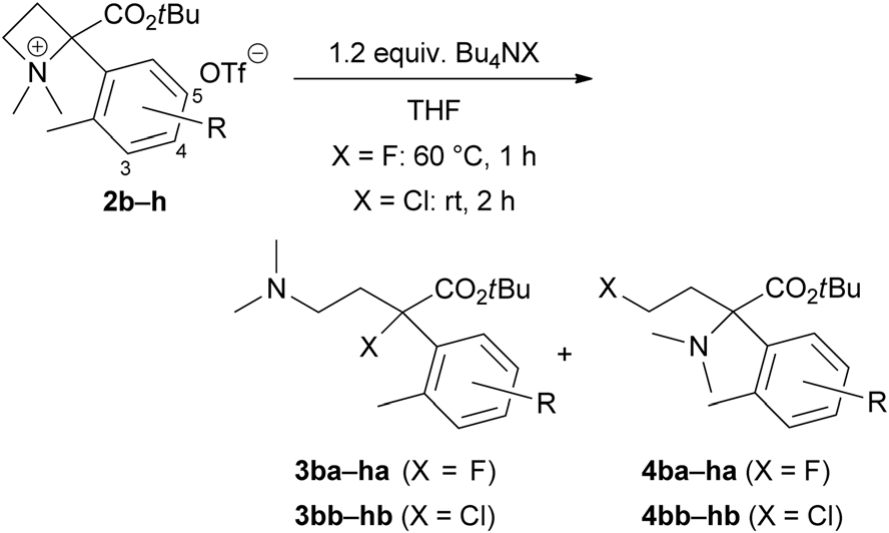					
Entry	X	R		3^a^ (%)	4^a^^,^^b^ (%)
1	F	5-Br	2b	77 (3ba)	N.D.
2	F	5-CF_3_	2c	75 (3ca)	N.D.
3	F	5-Me	2d	59 (3da)	N.D.
4	F	5-OMe	2e	61 (3ea)	N.D.
5	F	4-Br	2f	72 (3fa)	N.D.
6	F	4-CF_3_	2g	68 (3ga)	N.D.
7	F	3-Br	2h	58 (3ha)	N.D.
8	Cl	5-Br	2b	82 (3bb)	17 (4bb)
9	Cl	5-Me	2d	53 (3db)	19 (4db)
10	Cl	4-Br	2f	83 (3fb)	17 (4fb)
11	Cl	3-Br	2h	65 (3hb)	33 (4hb)

a Isolated yields.

b N.D. = not determined.

**Scheme 3:**
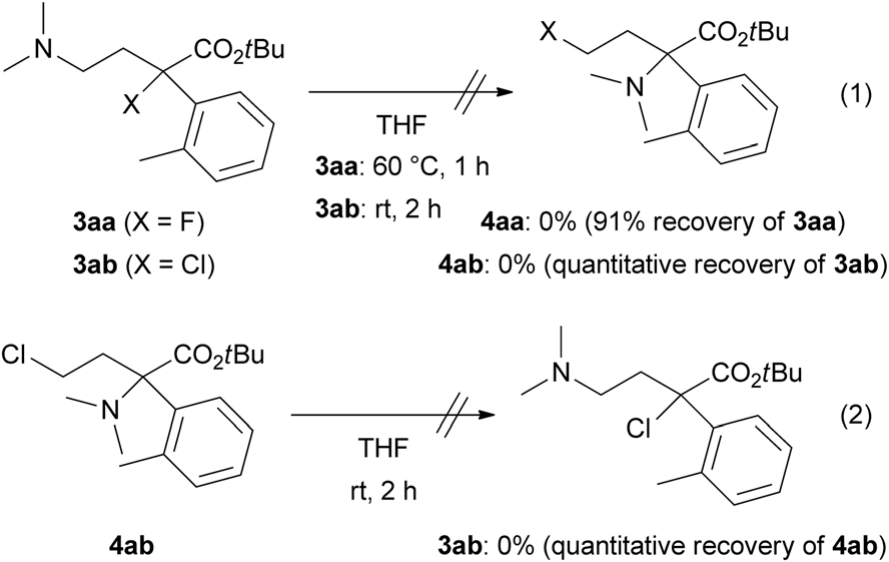
Chemical stability of ring-opening products 3aa, 3ab and 4ab.

We confirmed the chemical stability of products 3 and 4 (Scheme 3) because a transformation between 3 and 4 might proceed *via* the formation of ammonium salts generated from the alkyl halides and dimethylamino substituents as in the products (self-*N*-quaternization). A THF solution of tertiary alkyl halides 3aa (X = F) or 3ab (X = Cl) was subjected to the reaction temperature depicted in Table 1. The removal of THF by evaporation and ^1^H NMR analysis of the residue did not show any formation of 4aa or 4ab, respectively (eqn (1)). Similarly, a stirring at room temperature of a THF solution of primary alkyl chloride 4ab did not afford 3ab (eqn (2)).

**Scheme 4:**
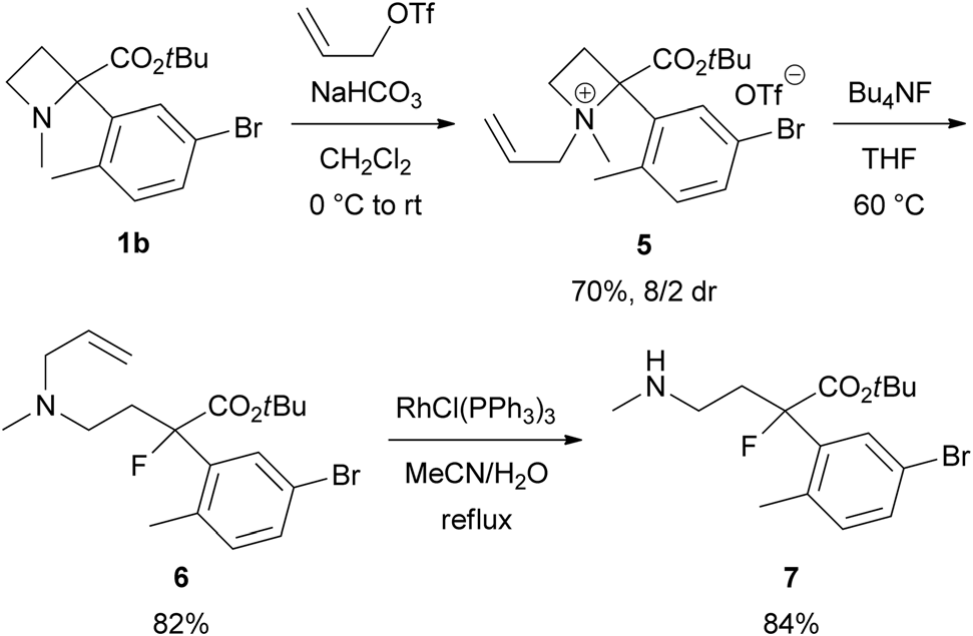
Synthesis of *N*-allyl derivative 5 and 6 and deallylation into 7.

The *N*,*N*-dimethylamino substituent, as in product 3, is not synthetically valuable because of the impossibility of removing the *N*-methyl substituents. One *N*-methyl substituent could be changed into an *N*-allyl, which would be removable *via* Rh-catalysed isomerization, by *N*-quaternization of 1 with allyl triflate^12^ (Scheme 4). For example, azetidinium salt 5 was prepared from 1b in 70% yield as an 8/2 mixture of diastereomers followed by ring-opening with Bu_4_NF to provide *N*-allyl derivative 6 in 82% yield. Rh-catalyzed deallylation of 6 gave secondary amine 7 in 84% yield.

**Scheme 5:**
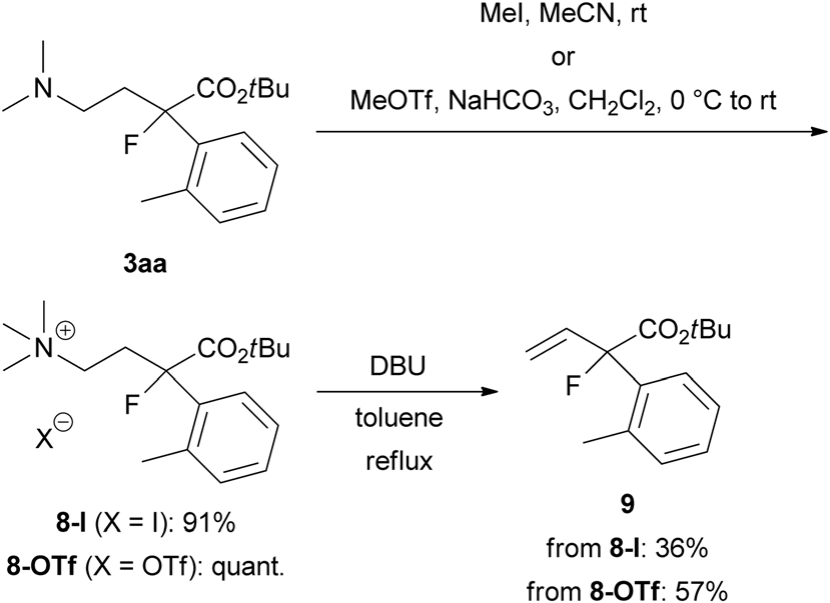
Synthesis of α-aryl-α-fluoro-α-vinylacetic acid ester 9 from 3aa by Hofmann elimination.

To demonstrate the utility of this ring-opening reaction, we attempted further synthetic transformations of organofluorine product 3aa. First, Hofmann elimination of 3aa to produce α-aryl-α-fluoro-α-vinylacetic acid ester 9 was examined (Scheme 5). *N*-Quaternization with iodomethane (MeI) or methyl trifluoromethanesulfonate (MeOTf) gave 8-I or 8-OTf in good yields (8-I: 91% yield, 8-OTf: quant.). Treatment of iodide salt 8-I with 1,8-diazabicyclo[5.4.0]undec-7-ene (DBU) in refluxing toluene for 1 day gave desired 9 in 36% yield. We expected that the iodide ion in the reaction mixture might cause undesirable side reactions such as nucleophilic substitutions, and the reaction resulted in a low yield. Thus, we examined the same reaction using triflate salt 8-OTf. As expected, the yield of 9 was improved to 57%.

**Scheme 6:**
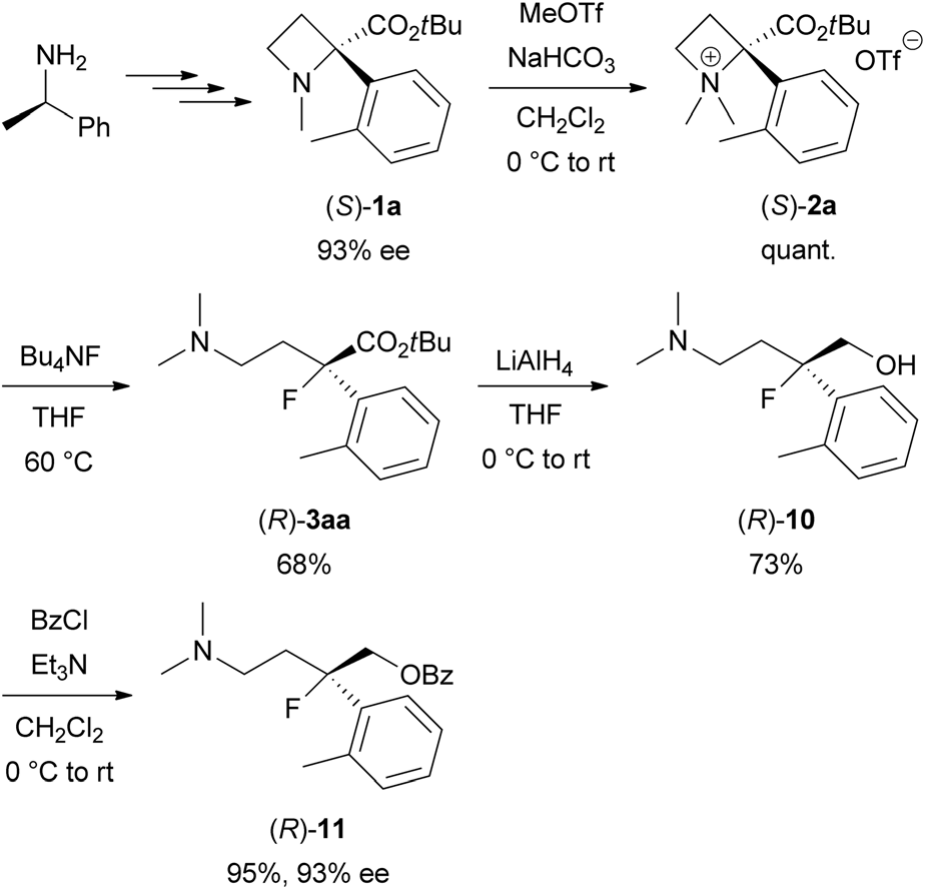
Synthesis of optically active organofluorine compound (*R*)-3aa starting from (*R*)-1-phenylethylamine.

Next, the synthesis of optically active tertiary organofluorine compounds from chiral (*R*)-1-phenylethylamine, which is one of the least expensive chiral sources, was examined (Scheme 6). 93% ee of (*S*)-1a was prepared according to our previous work.^6,13^*N*-Quaternization of (*S*)-1a with MeOTf to prepare (*S*)-2a (quant.) followed by the ring-opening reaction with Bu_4_NF under the conditions in Table 1 afforded (*R*)-3aa (68% yield). The ee of the obtained 3aa was determined after conversion into (*R*)-11 because of the low sensitivity of 3aa towards a UV/vis detector in chiral HPLC analysis. Reduction of (*R*)-3aa with LiAlH_4_ to amino alcohol (*R*)-10 (73% yield) followed by *O*-benzoylation gave benzoate (*R*)-11 (95% yield). The ee of (*R*)-11 was determined to be 93% ee by the chiral HPLC analysis. No lack of the ee was confirmed during the transformations from (*S*)-1a into (*R*)-11. This result indicates that the Bu_4_NF-promoted ring-opening reaction of (*S*)-2a affording (*R*)-3aa proceeds by inverting the tertiary carbon configuration (S_N_2) in the same manner as the reaction of (*S*)-2b with NaN_3_, which was previously reported by our group.^6^ Therefore, the absolute configuration of 3aa was determined to be (*R*).

**Scheme 7:**
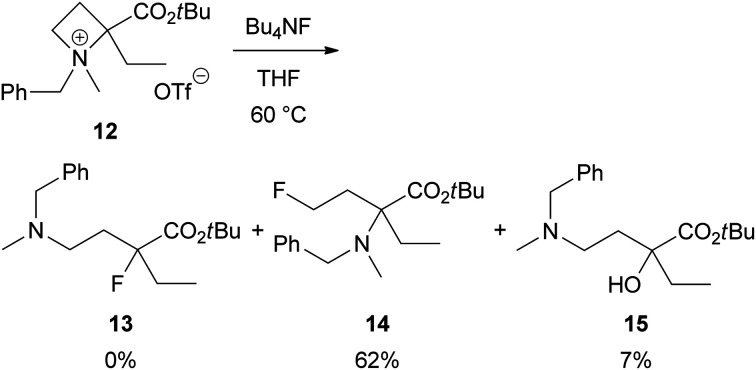
Nucleophilic ring-opening of α-ethyl azetidinium salt 12 with Bu_4_NF.

To clarify that the α-aryl substituent as in 2 is necessary for this site-selective ring-opening reaction to produce 3, we investigated a reaction α-ethyl derivative 12 with Bu_4_NF (Scheme 7). As expected, the reaction proceeded at 4-position preferentially to give γ-fluoro product 14 in 62% yield. Identifiable amount of the corresponding α-fluoro product 13 was not obtained. Instead, α-hydroxy derivative 15, which might be derived from 13, was isolated in 7% yield.

Couty's group described in the previous literature^1*d*^ that the nucleophilic ring-opening of *α*,*α*-disubstituted azetidinium ions at the quaternary α-carbon (2-position) is intrinsically favoured. Steric repulsions generated by substituents as in the azetidine ring affect the site-selectivity. The highly nucleophilic azide anion (N_3_^−^) reacts at 2-position, the less nucleophilic cyanide anion (CN^−^) reacts at 2- and 4-positions, and the poor nucleophilic acetate anion (AcO^−^) reacts at 4-position. The exact reason of the site-selective ring-opening reaction to produce 3 demonstrated by our group are difficult to explain at present, a size of the nucleophiles might affect the site-selectivity. F^−^ and Cl^−^ are small and enable to react at the quaternary α-carbon (2-position) although they are poor nucleophilic anion. Further experimental studies are needed to discuss.

## Conclusions

In conclusion, we described that the site-selective nucleophilic ring-opening reaction of 2-arylazetidine-2-carboxylic acid ester-derived ammonium salts 2 with Bu_4_NF or Bu_4_NCl proceeded at a much-substituted 2-position preferentially over a less-substituted 4-position and produced the corresponding tertiary alkyl fluorides and chlorides 3. Our result is a rare successful example of the fluoride ion-promoted ring-opening reaction of azetidine derivatives that yields organofluorine compounds. Further synthetic transformations of the product 3 were also successfully demonstrated. Our protocol enables the production of optically active organofluorine compound (*R*)-3aa starting from commercially available chiral (*R*)-1-phenylethylamine, which is an inexpensive chiral compound.

## Experimental

### General

Specific rotations were recorded on a JASCO polarimeter P-1010. Normal phase chiral HPLC analyses were performed using a JASCO HPLC pump (PU-2089) and a UV/vis detector (UV-2075). Infrared spectra (IR) were recorded on a JASCO FT/IR-4600 spectrometer. ^1^H, ^13^C and ^19^F NMR spectra were measured on a Varian (^1^H: 400 MHz, ^13^C: 101 MHz, ^19^F: 376 MHz) or a Bruker (^1^H: 400 MHz, ^13^C: 101 MHz) spectrometer. ^19^F NMR analysis were performed for representative products. As an internal standard in CDCl_3_, Me_4_Si (*δ* 0 ppm) for ^1^H NMR and CDCl_3_ (*δ* 77.00 ppm) for ^13^C NMR were used. As an internal standard in acetone-*d*_6_, the residual protons (*δ* 2.05 ppm) for ^1^H NMR and acetone-*d*_6_ (*δ* 29.92 ppm) for ^13^C NMR were used. In ^19^F NMR, hexafluorobenzene (C_6_F_6_) was used as an internal standard (*δ* −162.9 ppm). In ^1^H and ^13^C NMR, the splitting patterns are denoted as follows: s, singlet; d, doublet; t, triplet; q, quartet; m, multiplet; and br, broad peak. In ^19^F NMR, the splitting patterns are not denoted. High-resolution mass spectra (ESI) were measured on a Thermo Fisher Scientific LC/FT-MS spectrometer. Reactions involving air- or moisture-sensitive compounds were conducted in appropriate round-bottomed flasks with a magnetic stirring bar under an argon (Ar) atmosphere. A 1 M tetrabutylammonium fluoride (Bu_4_NF) THF solution was purchased from Tokyo Chemical Industry Co., Ltd. (TCI). Anhydrous tetrahydrofuran (THF) was purchased from KANTO Chemical Co., Inc. For the thin layer chromatography (TLC) analysis throughout this work, Silicagel 70 TLC Plate-Wako purchased from FUJIFILM Wako Chemical Corporation was used. The products were purified by column chromatography on silica gel (Wakosil 60, 64–210 μm) purchased from FUJIFILM Wako Chemical Corporation. For strong basic compound such as (*S*)-10, NH TLC plates and amino-functionalized silica gel (Chromatorex NH-DM1020) purchased from Fuji Silysia Chemical Ltd. (Japan) were used.

### Representative procedure for ring-opening of 2a with Bu_4_NF in THF to afford 3aa and 4aa (Table 1, entry 7)

A solution of 2-(*tert*-butoxycarbonyl)-1,1-dimethyl-2-(*o*-tolyl)azetidin-1-ium trifluoromethanesulfonate (2a) (62.3 mg, 0.146 mmol) in THF (0.55 mL) was stirred at 60 °C under an Ar atmosphere and treated with a 1 M Bu_4_NF THF solution (175 μL, 0.175 mmol). After stirring for 1 h at 60 °C, the resulting mixture was cooled to room temperature and diluted with H_2_O. The mixture was extracted with cyclohexane and the combined extracts were washed with H_2_O. The organic solution was dried over Na_2_SO_4_ and concentrated by evaporation. The residue was purified by chromatography on silica gel (CH_2_Cl_2_/MeOH = 100/0 to 10/1 as the eluent, *R*_f_: 3aa < 4aa) to obtain *tert*-butyl 4-(dimethylamino)-2-fluoro-2-(*o*-tolyl)butanoate (3aa) (30.7 mg, 71% yield) as a colourless oil and *tert*-butyl 2-(dimethylamino)-4-fluoro-2-(*o*-tolyl)butanoate (4aa) (3.1 mg, 7% yield) as a colourless oil. 3aa: IR (ATR) *ν*_max_/cm^−1^ 3063, 2976, 2938, 2862, 2818, 2766, 1748, 1731, 1459, 1392, 1368, 1281, 1250, 1148, 1091, 1064, 1042, 988, 965, 939, 844, 749; ^1^H NMR (400 MHz, CDCl_3_) *δ* 7.39 (1H, ddd, *J* = 7.6, 1.4, 1.4 Hz, ArH), 7.26–7.12 (3H, m, ArH), 2.68–2.37 (4H, m, CH_2_), 2.43 (3H, d, ^5^*J*_FH_ = 3.6 Hz, ArCH_3_), 2.28 (6H, s, N(CH_3_)_2_), 1.42 (9H, s, *t*Bu); ^13^C{^1^H} NMR (101 MHz, CDCl_3_) *δ* 169.2 (d, ^2^*J*_FC_ = 27 Hz), 136.8 (d, ^3^*J*_FC_ = 2 Hz), 136.1 (d, ^2^*J*_FC_ = 21 Hz), 132.1 (d, *J*_FC_ = 1 Hz), 128.5, 126.1 (d, ^3^*J*_FC_ = 7 Hz), 125.6, 96.5 (d, ^1^*J*_FC_ = 188 Hz), 82.6, 53.7 (d, ^3^*J*_FC_ = 5 Hz), 45.5, 34.7 (d, ^2^*J*_FC_ = 22 Hz), 27.7, 20.7 (d, ^4^*J*_FC_ = 7 Hz); ^19^F NMR (376 MHz, CDCl_3_) *δ* −157; HRMS (ESI): calcd for C_17_H_27_FNO_2_ [M + H]^+^ 296.2020, found 296.2016. 4aa: IR (ATR) *ν*_max_/cm^−1^ 3061, 2978, 2930, 2874, 2837, 2796, 1713, 1474, 1456, 1392, 1366, 1305, 1289, 1240, 1207, 1153, 1130, 1080, 1049, 1011, 986, 950, 883, 844, 814, 782, 749; ^1^H NMR (400 MHz, CDCl_3_) *δ* 7.43–7.37 (1H, m, ArH), 7.18–7.08 (3H, m, ArH), 4.24 (1H, dddd, ^2^*J*_FH_ = 47.2 Hz, *J* = 9.4, 9.2, 5.4 Hz, 4H), 4.06 (1H, dddd, ^2^*J*_FH_ = 46.8 Hz, *J* = 9.6, 9.2, 5.6 Hz, 4H), 2.66 (1H, dddd, ^3^*J*_FH_ = 15.3 Hz, *J* = 14.2, 9.6, 5.4 Hz, 3H), 2.42–2.24 (1H, m, 3H), 2.36 (6H, s, N(CH_3_)_2_), 2.35 (3H, s, ArCH_3_), 1.54 (9H, s, *t*Bu); ^13^C{^1^H} NMR (101 MHz, CDCl_3_) *δ* 168.5, 137.8, 136.2, 132.2, 128.1, 127.0, 124.9, 81.9, 81.5 (d, ^1^*J*_FC_ = 161 Hz), 71.4 (d, ^3^*J*_FC_ = 11 Hz), 40.0, 34.5 (d, ^2^*J*_FC_ = 21 Hz), 28.5, 21.1; ^19^F NMR (376 MHz, CDCl_3_) *δ* −222; HRMS (ESI): calcd for C_17_H_27_FNO_2_ [M + H]^+^ 296.2020, found 296.2013.

### Representative procedure for ring-opening of 2a with Bu_4_NCl in THF to afford 3ab and 4ab (Table 1, entry 9)

Bu_4_NCl (230 mg, 0.828 mmol) was added to a solution of 2a (293 mg, 0.689 mmol) in THF (3.6 mL) at room temperature and the mixture was degassed under reduced pressure and filled with an Ar. After stirring for 2 h, the resulting mixture was diluted with H_2_O. The mixture was extracted with *n*-hexane/EtOAc = 3/1 mixed solvent and the combined extracts were washed with H_2_O. The organic solution was dried over Na_2_SO_4_ and concentrated by evaporation. The residue was purified by chromatography on silica gel (CH_2_Cl_2_/MeOH = 100/0 to 30/1 as the eluent, *R*_f_: 3ab < 4ab) to obtain *tert*-butyl 2-chloro-4-(dimethylamino)-2-(*o*-tolyl)butanoate (3ab) (163 mg, 76% yield) as a pale yellow oil and *tert*-butyl 4-chloro-2-(dimethylamino)-2-(*o*-tolyl)butanoate (4ab) (49.8 mg, 23% yield) as a colourless oil. 3ab: IR (ATR) *ν*_max_/cm^−1^ 3062, 2976, 2939, 2861, 2818, 2766, 1732, 1458, 1392, 1368, 1254, 1145, 1080, 1028, 967, 934, 898, 843, 752, 722, 694; ^1^H NMR (400 MHz, CDCl_3_) *δ* 7.60 (1H, dd, *J* = 7.0, 1.8 Hz, ArH), 7.26–7.13 (3H, m, ArH), 2.73–2.50 (2H, m, CH_2_), 2.45–2.33 (2H, m, CH_2_), 2.35 (3H, s, ArCH_3_), 2.24 (6H, s, N(CH_3_)_2_), 1.43 (9H, s, *t*Bu); ^13^C{^1^H} NMR (101 MHz, CDCl_3_) *δ* 169.2, 138.1, 135.9, 132.0, 128.1, 126.2, 125.7, 83.0, 73.7, 55.1, 45.7, 38.0, 27.6, 20.5; HRMS (ESI): calcd for C_17_H_27_ClNO_2_ [M + H]^+^ 312.1725, found 312.1715. 4ab: IR (ATR) *ν*_max_/cm^−1^ 3060, 2977, 2931, 2872, 2836, 2795, 1712, 1479, 1454, 1392, 1366, 1336, 1294, 1230, 1151, 1081, 1046, 981, 961, 893, 843, 821, 774, 755, 725; ^1^H NMR (400 MHz, CDCl_3_) *δ* 7.41 (1H, d, *J* = 6.8 Hz, ArH), 7.19–7.09 (3H, m, ArH), 3.27 (1H, ddd, *J* = 12.0, 10.9, 4.3 Hz, 4H), 2.98 (1H, ddd, *J* = 12.1, 10.9, 5.2 Hz, 4H), 2.67 (1H, ddd, *J* = 13.9, 12.1, 4.3 Hz, 3H), 2.35 (6H, s, N(CH_3_)_2_), 2.33 (3H, S, ArCH_3_), 2.31 (1H, ddd, *J* = 13.9, 12.0, 5.2 Hz, 3H), 1.54 (9H, s, *t*Bu); ^13^C{^1^H} NMR (101 MHz, CDCl_3_) *δ* 168.3, 137.4, 136.1, 132.3, 128.3, 127.0, 124.9, 82.0, 72.3, 40.8, 40.0, 37.7, 28.5, 21.1; HRMS (ESI): calcd for C_17_H_27_ClNO_2_ [M + H]^+^ 312.1725, found 312.1719.

### Ring-opening of 2a with Bu_4_NBr in THF to afford 3ac and 4ac (Table 1, entry 13)

The procedure was similar to the synthesis of 3ab and 4ab. The reaction was performed at room temperature for 1 h using 2a (67.2 mg, 0.158 mmol) as a substrate. Purification by chromatography on silica gel (CH_2_Cl_2_/MeOH = 100/0 to 30/1 as the eluent, *R*_f_: 3ac < 4ac) gave *tert*-butyl 2-bromo-4-(dimethylamino)-2-(*o*-tolyl)butanoate (3ac) (34.1 mg, 61% yield) as a colourless oil and *tert*-butyl 4-bromo-2-(dimethylamino)-2-(*o*-tolyl)butanoate (4ac) (12.1 mg, 21% yield) as a colourless oil. 3ac: IR (ATR) *ν*_max_/cm^−1^ 3062, 2975, 2938, 2860, 2818, 2765, 1726, 1681, 1457, 1392, 1367, 1252, 1144, 1078, 1040, 1028, 965, 889, 843, 791, 751, 721, 689; ^1^H NMR (400 MHz, CDCl_3_) *δ* 7.68–7.61 (1H, m, ArH), 7.24–7.10 (3H, m, ArH), 2.72 (1H, ddd, *J* = 14.0, 11.0, 4.8 Hz, CH_2_), 2.64 (1H, ddd, *J* = 14.0, 10.8, 4.8 Hz, CH_2_), 2.44 (1H, ddd, *J* = 12.0, 10.8, 4.8 Hz, CH_2_), 2.38–2.28 (1H, m, CH_2_), 2.33 (3H, s, ArCH_3_), 2.25 (6H, s, N(CH_3_)_2_), 1.45 (9H, s, *t*Bu); ^13^C{^1^H} NMR (101 MHz, CDCl_3_) *δ* 169.3, 138.0, 135.4, 131.9, 128.2, 127.8, 125.8, 83.0, 69.2, 56.2, 45.6, 38.7, 27.5, 20.7; HRMS (ESI): calcd for C_17_H_27_BrNO_2_ [M + H]^+^ 356.1220, found 356.1218. 4ac: IR (ATR) *ν*_max_/cm^−1^ 3060, 2977, 2931, 2872, 2835, 2795, 1712, 1476, 1455, 1392, 1367, 1328, 1292, 1249, 1238, 1212, 1151, 1108, 1077, 1045, 1007, 979, 957, 892, 843, 819, 754; ^1^H NMR (400 MHz, CDCl_3_) *δ* 7.41 (1H, d, *J* = 7.2 Hz, ArH), 7.19–7.09 (3H, m, ArH), 3.11 (1H, ddd, *J* = 12.7, 9.0, 3.6 Hz, 4H), 2.84 (1H, ddd, *J* = 13.1, 9.0, 4.1 Hz, 4H), 2.75 (1H, ddd, *J* = 13.1, 12.8, 3.6 Hz, 3H), 2.39 (1H, ddd, *J* = 12.8, 12.7, 4.1 Hz, 3H), 2.35 (6H, s, N(CH_3_)_2_), 2.33 (3H, s, ArCH_3_), 1.54 (9H, s, *t*Bu); ^13^C{^1^H} NMR (101 MHz, CDCl_3_) *δ* 168.2, 137.3, 136.1, 132.3, 128.5, 127.0, 124.9, 82.0, 73.2, 40.0, 38.2, 28.9, 28.4, 21.1; HRMS (ESI): calcd for C_17_H_27_BrNO_2_ [M + H]^+^ 356.1220, found 356.1217.

### Ring-opening of 2a with KCN in DMF to afford 3ae and 4ae (Table 1, entry 15)

KCN (50.5 mg, 0.775 mmol) was added to a solution of 2a (67.0 mg, 0.157 mmol) in DMF (0.8 mL) at room temperature. The mixture was degassed under reduced pressure and filled with Ar. After stirring for 2 h under an Ar atmosphere, the resulting mixture was diluted with H_2_O and extracted with *n*-hexane/EtOAc = 3/1 mixed solvent. The combined extracts were washed with brine, dried over Na_2_SO_4_, and concentrated by evaporation. Purification of the residue by chromatography on silica gel (CH_2_Cl_2_/MeOH = 100/0 to 10/1 as the eluent, *R*_f_: 3ae < 4ae) to obtain *tert*-butyl 2-cyano-4-(dimethylamino)-2-(*o*-tolyl)butanoate (3ae) (20.1 mg, 42% yield) as pale yellow crystals and *tert*-butyl 4-cyano-2-(dimethylamino)-2-(*o*-tolyl)butanoate (4ae) (26.3 mg, 55% yield) as a colourless crystals. 3ae: mp 29–31 °C; IR (ATR) *ν*_max_/cm^−1^ 3065, 2977, 2941, 2863, 2820, 2769, 2240, 1734, 1459, 1393, 1369, 1244, 1147, 1098, 1041, 969, 936, 838, 753, 730; ^1^H NMR (400 MHz, CDCl_3_) *δ* 7.40 (1H, dd, *J* = 7.8, 1.8 Hz, ArH), 7.30–7.19 (3H, m, ArH), 2.65–2.50 (3H, m, CH_2_), 2.48 (3H, s, ArCH_3_), 2.40 (1H, ddd, *J* = 11.4, 8.6, 4.4 Hz, CH_2_), 1.47 (9H, s, *t*Bu); ^13^C{^1^H} NMR (101 MHz, CDCl_3_) *δ* 166.7, 136.4, 133.1, 132.3, 128.5, 126.5, 126.4, 118.5, 84.2, 55.4, 51.6, 45.5, 33.9, 27.5, 20.3; HRMS (ESI): calcd for C_18_H_27_N_2_O_2_ [M + H]^+^ 303.2067, found 303.2057. 4ae: mp 31–33 °C; IR (ATR) *ν*_max_/cm^−1^ 3061, 2977, 2933, 2875, 2837, 2796, 2246, 1708, 1475, 1455, 1441, 1392, 1367, 1234, 1151, 1083, 1041, 1028, 987, 968, 915, 873, 842, 813, 756; ^1^H NMR (400 MHz, CDCl_3_) *δ* 7.42–7.36 (1H, m, ArH), 7.21–7.11 (3H, m, ArH), 2.64–2.53 (1H, m, CH_2_), 2.34 (6H, s, N(CH_3_)_2_), 2.32 (3H, s, ArCH_3_), 2.20–2.07 (2H, m, CH_2_), 1.92–1.75 (1H, m, CH_2_), 1.54 (9H, s, *t*Bu); ^13^C{^1^H} NMR (101 MHz, CDCl_3_) *δ* 168.0, 136.6, 135.9, 132.5, 128.6, 127.4, 125.0, 120.1, 82.3, 71.7, 40.0, 30.3, 28.4, 21.0, 12.1; HRMS (ESI): calcd for C_18_H_27_N_2_O_2_ [M + H]^+^ 303.2067, found 303.2058.

### *tert*-Butyl 2-(5-bromo-2-methylphenyl)-4-(dimethylamino)-2-fluorobutanoate (3ba) (Table 2, entry 1)

Obtained from 2b (76.0 mg, 0.151 mmol) by the same procedure with 3aa. Purification by chromatography on silica gel (CH_2_Cl_2_/MeOH = 30/1 to 20/1 as the eluent) gave 3ba (43.3 mg, 77% yield) as a pale yellow oil. IR (ATR) *ν*_max_/cm^−1^ 2976, 2938, 2861, 2818, 2767, 1749, 1731, 1592, 1565, 1481, 1459, 1391, 1368, 1283, 1251, 1218, 1149, 1114, 1092, 1041, 989, 969, 944, 876, 842, 809, 769, 738, 704; ^1^H NMR (400 MHz, CDCl_3_) *δ* 7.53 (1H, dd, *J* = 1.8, 1.8 Hz, ArH), 7.35 (1H, dd, *J* = 8.0, 1.8 Hz, ArH), 7.03 (1H, d, *J* = 8.0 Hz, ArH), 2.73–2.30 (4H, m, CH_2_), 2.37 (3H, d, ^5^*J*_FH_ = 3.6 Hz, ArCH_3_), 2.27 (6H, s, N(CH_3_)_2_), 1.43 (9H, s, *t*Bu); ^13^C{^1^H} NMR (101 MHz, CDCl_3_) *δ* 168.4 (d, ^2^*J*_FC_ = 27 Hz), 138.3 (d, ^2^*J*_FC_ = 21 Hz), 135.5 (d, ^3^*J*_FC_ = 2 Hz), 133.7, 131.4, 129.1 (d, ^3^*J*_FC_ = 9 Hz), 119.3, 95.8 (d, ^1^*J*_FC_ = 190 Hz), 83.1, 53.4 (d, ^3^*J*_FC_ = 5 Hz), 45.4, 34.4 (d, ^2^*J*_FC_ = 22 Hz), 27.7, 20.2 (d, ^4^*J*_FC_ = 7 Hz); HRMS (ESI): calcd for C_17_H_26_BrFNO_2_ [M + H]^+^ 374.1125, found 374.1124.

### *tert*-Butyl 4-(dimethylamino)-2-fluoro-2-(2-methyl-5-(trifluoromethyl)phenyl)butanoate (3ca) (Table 2, entry 2)

Obtained from 2c (167 mg, 0.338 mmol) by the same procedure with 3aa. Purification by chromatography on silica gel (CH_2_Cl_2_/MeOH = 30/1 to 20/1 as the eluent) gave 3ca (92.2 mg, 75% yield) as a pale yellow oil. IR (ATR) *ν*_max_/cm^−1^ 2979, 2942, 2864, 2821, 2769, 1752, 1732, 1621, 1460, 1393, 1370, 1331, 1286, 1252, 1151, 1120, 1091, 1040, 1006, 990, 976, 948, 896, 842, 829, 771, 748, 721; ^1^H NMR (400 MHz, CDCl_3_) *δ* 7.66 (1H, s, ArH), 7.49 (1H, d, *J* = 8.0 Hz, ArH), 7.29 (1H, d, *J* = 8.0 Hz, ArH), 2.78–2.31 (4H, m, CH_2_), 2.49 (3H, d, ^5^*J*_FH_ = 3.2 Hz, ArCH_3_), 2.28 (6H, s, N(CH_3_)_2_), 1.43 (9H, s, *t*Bu); ^13^C{^1^H} NMR (101 MHz, CDCl_3_) *δ* 168.4 (d, ^2^*J*_FC_ = 27 Hz), 140.9 (d, ^3^*J*_FC_ = 2 Hz), 137.2 (d, ^2^*J*_FC_ = 22 Hz), 132.6, 128.1 (q, ^2^*J*_FC_ = 33 Hz), 125.3–125.0 (m), 124.1 (q, ^1^*J*_FC_ = 273 Hz), 123.2 (dq, ^3^*J*_FC_, ^3^*J*_FC_ = 8, 4 Hz), 96.0 (d, ^1^*J*_FC_ = 190 Hz), 83.1, 53.4 (d, ^3^*J*_FC_ = 5 Hz), 45.4, 34.7 (d, ^2^*J*_FC_ = 22 Hz), 27.6, 20.7 (d, ^4^*J*_FC_ = 8 Hz); HRMS (ESI): calcd for C_18_H_26_F_4_NO_2_ [M + H]^+^ 364.1894, found 364.1876.

### *tert*-Butyl 4-(dimethylamino)-2-(2,5-dimethylphenyl)-2-fluorobutanoate (3da) (Table 2, entry 3)

Obtained from 2d (105 mg, 0.239 mmol) by the same procedure with 3aa. Purification by chromatography on silica gel (CH_2_Cl_2_/MeOH = 30/1 to 20/1 as the eluent) gave 3da (43.8 mg, 59% yield) as a pale yellow oil. IR (ATR) *ν*_max_/cm^−1^ 2976, 2936, 2862, 2818, 2766, 1749, 1731, 1499, 1459, 1392, 1368, 1285, 1250, 1149, 1090, 1040, 990, 952, 844, 811, 771, 748, 707; ^1^H NMR (400 MHz, CDCl_3_) *δ* 7.19 (1H, s, ArH), 7.07–6.99 (2H, m, ArH), 2.67–2.39 (4H, m, CH_2_), 2.38 (3H, d, ^5^*J*_FH_ = 4.0 Hz, ArCH_3_), 2.32 (3H, s, ArCH_3_), 2.29 (6H, s, N(CH_3_)_2_), 1.43 (9H, s, *t*Bu); ^13^C{^1^H} NMR (101 MHz, CDCl_3_) *δ* 169.2 (d, ^2^*J*_FC_ = 26 Hz), 135.8 (d, ^2^*J*_FC_ = 21 Hz), 134.9, 133.4 (d, ^3^*J*_FC_ = 2 Hz), 132.0 (d, *J*_FC_ = 2 Hz), 129.1, 126.8 (d, ^3^*J*_FC_ = 8 Hz), 96.5 (d, ^1^*J*_FC_ = 187 Hz), 82.6, 53.6 (d, ^3^*J*_FC_ = 5 Hz), 45.4, 34.6 (d, ^2^*J*_FC_ = 22 Hz), 27.7, 21.1, 20.2 (d, ^4^*J*_FC_ = 8 Hz); HRMS (ESI): calcd for C_18_H_29_FNO_2_ [M + H]^+^ 310.2177, found 310.2163.

### *tert*-Butyl 4-(dimethylamino)-2-fluoro-2-(5-methoxy-2-methylphenyl)butanoate (3ea) (Table 2, entry 4)

Obtained from 2e (127 mg, 0.279 mmol) by the same procedure with 3aa. Purification by chromatography on silica gel (CH_2_Cl_2_/MeOH = 30/1 to 20/1 as the eluent) gave 3ea (55.4 mg, 61% yield) as a pale yellow oil. IR (ATR) *ν*_max_/cm^−1^ 2976, 2938, 2862, 2818, 2766, 1748, 1731, 1612, 1577, 1498, 1459, 1392, 1368, 1290, 1249, 1149, 1090, 1078, 1041, 978, 957, 863, 844, 810, 771, 746, 735, 708; ^1^H NMR (400 MHz, CDCl_3_) *δ* 7.07 (1H, d, *J* = 8.4 Hz, ArH), 6.97 (1H, dd, *J* = 2.4, 1.2 Hz, ArH), 6.77 (1H, dd, *J* = 8.4, 2.4 Hz, ArH), 3.79 (3H, s, OCH_3_), 2.64–2.37 (4H, m, CH_2_), 2.35 (3H, d, ^5^*J*_FH_ = 3.6 Hz, ArCH_3_), 2.27 (6H, s, N(CH_3_)_2_), 1.43 (9H, s, *t*Bu); ^13^C{^1^H} NMR (101 MHz, CDCl_3_) *δ* 168.9 (d, ^2^*J*_FC_ = 26 Hz), 157.4 (d, ^4^*J*_FC_ = 2 Hz), 137.2 (d, ^2^*J*_FC_ = 21 Hz), 132.9, 128.3 (d, ^3^*J*_FC_ = 2 Hz), 113.1 (d, ^5^*J*_FC_ = 2 Hz), 112.6 (d, ^3^*J*_FC_ = 9 Hz), 96.3 (d, ^1^*J*_FC_ = 189 Hz), 82.6, 55.3, 53.6 (d, ^3^*J*_FC_ = 5 Hz), 45.4, 34.6 (d, ^2^*J*_FC_ = 22 Hz), 27.7, 19.7 (d, ^4^*J*_FC_ = 7 Hz); HRMS (ESI): calcd for C_18_H_29_FNO_3_ [M + H]^+^ 326.2126, found 326.2115.

### *tert*-Butyl 2-(4-bromo-2-methylphenyl)-4-(dimethylamino)-2-fluorobutanoate (3fa) (Table 2, entry 5)

Obtained from 2f (318 mg, 0.631 mmol) by the same procedure with 3aa. Purification by chromatography on silica gel (CH_2_Cl_2_/MeOH = 30/1 to 20/1 as the eluent) gave 3fa (170 mg, 72% yield) as a pale yellow oil. IR (ATR) *ν*_max_/cm^−1^ 2976, 2938, 2862, 2818, 2766, 1749, 1731, 1590, 1561, 1480, 1459, 1391, 1368, 1275, 1250, 1216, 1148, 1092, 1042, 988, 960, 940, 900, 844, 811, 768, 747, 704; ^1^H NMR (400 MHz, CDCl_3_) *δ* 7.35–7.29 (2H, m, ArH), 7.29–7.23 (1H, m, ArH), 2.66–2.30 (4H, m, CH_2_), 2.40 (3H, d, ^5^*J*_FH_ = 3.6 Hz, ArCH_3_), 2.26 (6H, s, N(CH_3_)_2_), 1.42 (9H, s, *t*Bu); ^13^C{^1^H} NMR (101 MHz, CDCl_3_) *δ* 168.6 (d, ^2^*J*_FC_ = 27 Hz), 139.0 (d, ^3^*J*_FC_ = 2 Hz), 135.3 (d, ^2^*J*_FC_ = 21 Hz), 134.7, 128.6, 127.8 (d, ^3^*J*_FC_ = 8 Hz), 122.4 (d, *J*_FC_ = 2 Hz), 96.0 (d, ^1^*J*_FC_ = 189 Hz), 82.8, 53.4 (d, ^3^*J*_FC_ = 5 Hz), 45.4, 34.6 (d, ^2^*J*_FC_ = 22 Hz), 27.6, 20.4 (d, ^4^*J*_FC_ = 8 Hz); HRMS (ESI): calcd for C_17_H_26_BrFNO_2_ [M + H]^+^ 374.1125, found 374.1110.

### *tert*-Butyl 4-(dimethylamino)-2-fluoro-2-(2-methyl-4-(trifluoromethyl)phenyl)butanoate (3ga) (Table 2, entry 6)

Obtained from 2g (74.8 mg, 0.152 mmol) by the same procedure with 3aa. Purification by chromatography on silica gel (CH_2_Cl_2_/MeOH = 30/1 to 20/1 as the eluent) gave 3ga (37.4 mg, 68% yield) as a colourless oil. IR (ATR) *ν*_max_/cm^−1^ 2979, 2940, 2864, 2821, 2769, 1750, 1732, 1619, 1460, 1411, 1394, 1370, 1333, 1285, 1252, 1217, 1150, 1122, 1090, 1042, 1008, 990, 965, 944, 890, 840, 814, 774, 741, 711; ^1^H NMR (400 MHz, CDCl_3_) *δ* 7.52 (1H, d, *J* = 8.6 Hz, ArH), 7.46 (1H, d, *J* = 8.6 Hz, ArH), 7.42 (1H, s, ArH), 2.71–2.35 (4H, m, CH_2_), 2.49 (3H, d, ^5^*J*_FH_ = 4.0 Hz, ArCH_3_), 2.28 (6H, s, N(CH_3_)_2_), 1.42 (9H, s, *t*Bu); ^13^C{^1^H} NMR (101 MHz, CDCl_3_) *δ* 168.4 (d, ^2^*J*_FC_ = 26 Hz), 139.9 (d, ^2^*J*_FC_ = 21 Hz), 137.6 (d, ^3^*J*_FC_ = 1 Hz), 130.5 (qd, ^2^*J*_FC_, ^5^*J*_FC_ = 33, 2 Hz), 128.8 (q, ^3^*J*_FC_ = 4 Hz), 126.7 (d, ^3^*J*_FC_ = 9 Hz), 123.9 (q, ^1^*J*_FC_ = 273 Hz), 122.5 (q, ^3^*J*_FC_ = 4 Hz), 96.1 (d, ^1^*J*_FC_ = 189 Hz), 83.2, 53.4 (d, ^3^*J*_FC_ = 5 Hz), 45.4, 34.6 (d, ^2^*J*_FC_ = 22 Hz), 27.7, 20.8 (d, ^4^*J*_FC_ = 8 Hz); HRMS (ESI): calcd for C_18_H_26_F_4_NO_2_ [M + H]^+^ 364.1894, found 364.1882.

### *tert*-Butyl 2-(3-bromo-2-methylphenyl)-4-(dimethylamino)-2-fluorobutanoate (3ha) (Table 2, entry 7)

Obtained from 2h (155 mg, 0.307 mmol) by the same procedure with 3aa. Purification by chromatography on silica gel (CH_2_Cl_2_/MeOH = 30/1 to 20/1 as the eluent) gave 3ha (66.7 mg, 58% yield) as a pale yellow oil. IR (ATR) *ν*_max_/cm^−1^ 2976, 2938, 2861, 2818, 2766, 1748, 1732, 1562, 1459, 1432, 1392, 1368, 1283, 1250, 1148, 1092, 1076, 1033, 1005, 965, 944, 903, 842, 783, 763, 742, 715; ^1^H NMR (400 MHz, CDCl_3_) *δ* 7.57 (1H, d, *J* = 8.0 Hz, ArH), 7.38 (1H, d, *J* = 8.0 Hz, ArH), 7.06 (1H, ddq, *J* = 8.0, 8.0, 0.5 Hz, ArH), 2.66–2.34 (4H, m, CH_2_), 2.47 (3H, d, ^5^*J*_FH_ = 2.8 Hz, ArCH_3_), 2.26 (6H, s, N(CH_3_)_2_), 1.43 (9H, s, *t*Bu); ^13^C{^1^H} NMR (101 MHz, CDCl_3_) *δ* 169.0 (d, ^2^*J*_FC_ = 26 Hz), 138.4 (d, ^2^*J*_FC_ = 21 Hz), 136.4, 133.1 (d, ^3^*J*_FC_ = 2 Hz), 127.7 (d, *J*_FC_ = 2 Hz), 126.6, 125.5 (d, ^3^*J*_FC_ = 8 Hz), 96.0 (d, ^1^*J*_FC_ = 190 Hz), 83.0, 53.5 (d, ^3^*J*_FC_ = 5 Hz), 45.5, 34.9 (d, ^2^*J*_FC_ = 22 Hz), 27.7, 20.3 (d, ^4^*J*_FC_ = 7 Hz); HRMS (ESI): calcd for C_17_H_26_BrFNO_2_ [M + H]^+^ 374.1125, found 374.1120.

### *tert*-Butyl 2-(5-bromo-2-methylphenyl)-2-chloro-4-(dimethylamino)butanoate (3bb) and *tert*-butyl 2-(5-bromo-2-methylphenyl)-4-chloro-2-(dimethylamino)butanoate (4bb) (Table 2, entry 8)

Obtained from 2b (135 mg, 0.268 mmol) by the same procedure with 3ab and 4ab. Purification by chromatography on silica gel (CH_2_Cl_2_/MeOH = 100/0 to 30/1 as the eluent *R*_f_: 3bb < 4bb) gave 3bb (85.5 mg, 82% yield) as colourless crystals and 4bb (18.0 mg, 17% yield) as a colourless oil. 3bb: mp 57–59 °C; IR (ATR) *ν*_max_/cm^−1^ 2998, 2975, 2939, 2857, 2813, 2759, 1739, 1593, 1566, 1481, 1459, 1393, 1366, 1288, 1263, 1232, 1179, 1143, 1100, 1080, 1063, 1041, 1029, 920, 875, 849, 811, 796, 768, 755, 722; ^1^H NMR (400 MHz, CDCl_3_) *δ* 7.74 (1H, d, *J* = 2.0 Hz, ArH), 7.35 (1H, dd, *J* = 8.2, 2.0 Hz, ArH), 7.03 (1H, d, *J* = 8.2 Hz, ArH), 2.63 (1H, ddd, *J* = 13.5, 10.1, 5.6 Hz, CH_2_), 2.52 (1H, ddd, *J* = 13.5, 9.6, 5.8 Hz, CH_2_), 2.44–2.31 (2H, m, CH_2_), 2.29 (3H, s, ArCH_3_), 2.25 (6H, s, N(CH_3_)_2_), 1.44 (9H, s, *t*Bu); ^13^C{^1^H} NMR (101 MHz, CDCl_3_) *δ* 168.6, 140.1, 134.8, 133.5, 131.1, 129.5, 119.4, 83.4, 72.8, 54.9, 45.6, 37.8, 27.6, 20.0; HRMS (ESI): calcd for C_17_H_26_BrClNO_2_ [M + H]^+^ 390.0830, found 390.0822. 4bb: IR (ATR) *ν*_max_/cm^−1^ 2977, 2931, 2872, 2837, 2797, 1712, 1589, 1563, 1476, 1455, 1391, 1367, 1336, 1294, 1231, 1151, 1119, 1100, 1082, 1049, 1030, 984, 967, 912, 876, 843, 803, 772, 734; ^1^H NMR (400 MHz, CDCl_3_) *δ* 7.61 (1H, d, *J* = 2.0 Hz, ArH), 7.29 (1H, dd, *J* = 8.2, 2.0 Hz, ArH), 7.01 (1H, d, *J* = 8.2 Hz, ArH), 3.33 (1H, ddd, *J* = 12.0, 10.8, 4.0 Hz, CH_2_), 2.93 (1H, ddd, *J* = 12.2, 10.8, 5.5 Hz, CH_2_), 2.68 (1H, ddd, *J* = 14.4, 12.2, 4.0 Hz, CH_2_), 2.34 (6H, s, N(CH_3_)_2_), 2.31–2.16 (1H, m, CH_2_), 2.25 (3H, s, ArCH_3_), 1.53 (9H, s, *t*Bu); ^13^C{^1^H} NMR (101 MHz, CDCl_3_) *δ* 167.3, 139.9, 135.0, 133.9, 131.2, 130.0, 119.1, 82.4, 71.8, 40.3, 40.0, 37.2, 28.4, 20.7; HRMS (ESI): calcd for C_17_H_26_BrClNO_2_ [M + H]^+^ 390.0830, found 390.0826.

### *tert*-Butyl 2-chloro-4-(dimethylamino)-2-(2,5-dimethylphenyl)butanoate (3db) and *tert*-butyl 4-chloro-2-(dimethylamino)-2-(2,5-dimethylphenyl)butanoate (4db) (Table 2, entry 9)

Obtained from 2d (85.8 mg, 0.195 mmol) by the same procedure with 3ab and 4ab. Purification by chromatography on silica gel (CH_2_Cl_2_/MeOH = 100/0 to 30/1 as the eluent *R*_f_: 3db < 4db) gave 3db (33.9 mg, 53% yield) as colourless crystals and 4db (12.3 mg, 19% yield) as a colourless oil. 3db: mp 41–42 °C; IR (ATR) *ν*_max_/cm^−1^ 2976, 2938, 2861, 2818, 2765, 1732, 1615, 1497, 1458, 1392, 1367, 1252, 1146, 1080, 1039, 992, 969, 928, 911, 846, 809, 771, 745, 718, 700; ^1^H NMR (400 MHz, CDCl_3_) *δ* 7.40 (1H, s, ArH), 7.07–6.99 (2H, m, ArH), 2.71–2.52 (2H, m, CH_2_), 2.47–2.35 (2H, m, CH_2_), 2.34 (3H, s, ArCH_3_), 2.30 (3H, s, ArCH_3_), 2.26 (6H, s, N(CH_3_)_2_), 1.44 (9H, s, *t*Bu); ^13^C{^1^H} NMR (101 MHz, CDCl_3_) *δ* 169.3, 137.8, 135.0, 132.6, 131.9, 128.8, 127.0, 82.9, 73.8, 55.1, 45.6, 37.9, 27.6, 21.2, 20.0; HRMS (ESI): calcd for C_18_H_29_ClNO_2_ [M + H]^+^ 326.1881, found 326.1873. 4db: IR (ATR) *ν*_max_/cm^−1^ 2977, 2929, 2871, 2836, 2795, 1712, 1613, 1497, 1455, 1392, 1366, 1336, 1297, 1234, 1151, 1081, 1055, 1035, 978, 967, 898, 846, 810, 773, 757, 725; ^1^H NMR (400 MHz, CDCl_3_) *δ* 7.17 (1H, br s, ArH), 7.01 (1H, d, *J* = 7.7 Hz, ArH), 6.96 (1H, dd, *J* = 7.7, 1.8 Hz, ArH), 3.22 (1H, ddd, *J* = 12.0, 10.8, 4.5 Hz, CH_2_), 3.03 (1H, ddd, *J* = 12.4, 10.8, 5.1 Hz, CH_2_), 2.63 (1H, ddd, *J* = 13.8, 12.4, 4.5 Hz, CH_2_), 2.37–2.27 (1H, m, CH_2_), 2.35 (6H, s, N(CH_3_)_2_), 2.294 (3H, s, ArCH_3_), 2.288 (3H, s, ArCH_3_), 1.54 (9H, s, *t*Bu); ^13^C{^1^H} NMR (101 MHz, CDCl_3_) *δ* 168.7, 137.1, 134.2, 132.9, 132.2, 129.0, 127.7, 81.9, 72.5, 41.0, 40.0, 37.9, 28.5, 21.2, 20.6; HRMS (ESI): calcd for C_18_H_29_ClNO_2_ [M + H]^+^ 326.1881, found 326.1874.

### *tert*-Butyl 2-(4-bromo-2-methylphenyl)-2-chloro-4-(dimethylamino)butanoate (3fb) and *tert*-butyl 2-(4-bromo-2-methylphenyl)-4-chloro-2-(dimethylamino)butanoate (4fb) (Table 2, entry 10)

Obtained from 2f (95.6 mg, 0.190 mmol) by the same procedure with 3ab and 4ab. Purification by chromatography on silica gel (CH_2_Cl_2_/MeOH = 100/0 to 30/1 as the eluent *R*_f_: 3fb < 4fb) gave 3fb (61.4 mg, 83% yield) as a pale yellow oil and 4fb (12.9 mg, 17% yield) as colourless crystals. 3fb: IR (ATR) *ν*_max_/cm^−1^ 2975, 2939, 2861, 2818, 2766, 1734, 1589, 1560, 1458, 1391, 1368, 1252, 1145, 1079, 1029, 967, 934, 899, 871, 843, 803, 770, 739, 722, 699; ^1^H NMR (400 MHz, CDCl_3_) *δ* 7.47 (1H, dd, *J* = 7.8, 1.0 Hz, ArH), 7.35–7.30 (2H, m, ArH), 2.63 (1H, ddd, *J* = 13.6, 10.1, 5.7 Hz, CH_2_), 2.52 (1H, ddd, *J* = 13.6, 9.3, 5.9 Hz, CH_2_), 2.43–2.31 (2H, m, CH_2_), 2.33 (3H, s, ArCH_3_), 2.24 (6H, s, N(CH_3_)_2_), 1.43 (9H, s, *t*Bu); ^13^C{^1^H} NMR (101 MHz, CDCl_3_) *δ* 168.7, 138.2, 137.3, 134.6, 128.7, 128.0, 122.1, 83.3, 73.1, 54.9, 45.6, 37.8, 27.5, 20.3; HRMS (ESI): calcd for C_17_H_26_BrClNO_2_ [M + H]^+^ 390.0830, found 390.0820. 4fb: mp 132–134 °C; IR (ATR) *ν*_max_/cm^−1^ 3001, 2974, 2943, 2927, 2869, 2850, 2839, 2800, 1714, 1587, 1560, 1473, 1453, 1389, 1367, 1338, 1300, 1261, 1229, 1177, 1152, 1083, 1049, 1027, 984, 961, 871, 831, 814, 771, 756, 739, 724; ^1^H NMR (400 MHz, CDCl_3_) *δ* 7.36–7.23 (3H, m, ArH), 3.29 (1H, ddd, *J* = 12.3, 10.8, 4.3 Hz, CH_2_), 2.93 (1H, ddd, *J* = 12.1, 10.8, 5.3 Hz, CH_2_), 2.67 (1H, ddd, *J* = 14.3, 12.1, 4.3 Hz, CH_2_), 2.33 (6H, s, N(CH_3_)_2_), 2.29 (3H, s, ArCH_3_), 2.24 (1H, ddd, *J* = 14.3, 12.3, 5.3 Hz, CH_2_), 1.53 (9H, s, *t*Bu); ^13^C{^1^H} NMR (101 MHz, CDCl_3_) *δ* 167.6, 138.4, 136.8, 134.9, 130.2, 128.0, 120.9, 82.4, 71.9, 40.3, 40.0, 37.3, 28.4, 20.9; HRMS (ESI): calcd for C_17_H_26_BrClNO_2_ [M + H]^+^ 390.0830, found 390.0824.

### *tert*-Butyl 2-(3-bromo-2-methylphenyl)-2-chloro-4-(dimethylamino)butanoate (3hb) and *tert*-butyl 2-(3-bromo-2-methylphenyl)-4-chloro-2-(dimethylamino)butanoate (4hb) (Table 2, entry 11)

Obtained from 2h (149 mg, 0.295 mmol) by the same procedure with 3ab and 4ab. Purification by chromatography on silica gel (CH_2_Cl_2_/MeOH = 100/0 to 30/1 as the eluent *R*_f_: 3hb < 4hb) gave 3hb (74.7 mg, 65% yield) as colourless crystals and 4hb (37.5 mg, 33% yield) as a colourless oil. 3hb: mp 45–47 °C; IR (ATR) *ν*_max_/cm^−1^ 2976, 2940, 2861, 2818, 2766, 1733, 1561, 1459, 1429, 1392, 1368, 1304, 1251, 1146, 1078, 1031, 997, 967, 939, 912, 841, 783, 765, 742, 714; ^1^H NMR (400 MHz, CDCl_3_) *δ* 7.59 (1H, dd, *J* = 8.1, 1.0 Hz, ArH), 7.55 (1H, dd, *J* = 8.0, 1.0 Hz, ArH), 7.07 (1H, dd, *J* = 8.1, 8.0 Hz, ArH), 2.66 (1H, ddd, *J* = 13.4, 11.1, 4.8 Hz, CH_2_), 2.52 (1H, ddd, *J* = 13.4, 10.4, 4.8 Hz, CH_2_), 2.41 (1H, ddd, *J* = 12.0, 10.4, 4.8 Hz, CH_2_), 2.38 (3H, s, ArCH_3_), 2.34 (1H, ddd, *J* = 12.0, 11.1, 4.8 Hz, CH_2_), 2.24 (6H, s, N(CH_3_)_2_), 1.44 (9H, s, *t*Bu); ^13^C{^1^H} NMR (101 MHz, CDCl_3_) *δ* 169.1, 140.1, 135.6, 132.7, 127.5, 126.6, 125.7, 83.4, 73.5, 54.9, 45.6, 38.3, 27.5, 21.1; HRMS (ESI): calcd for C_17_H_26_BrClNO_2_ [M + H]^+^ 390.0830, found 390.0823. 4hb: IR (ATR) *ν*_max_/cm^−1^ 2976, 2930, 2872, 2837, 2797, 1713, 1560, 1455, 1428, 1392, 1366, 1336, 1295, 1233, 1151, 1098, 1081, 1048, 986, 962, 875, 842, 821, 789, 736, 719; ^1^H NMR (400 MHz, CDCl_3_) *δ* 7.50 (1H, dd, *J* = 7.9, 1.0 Hz, ArH), 7.46 (1H, br d, *J* = 8.0 Hz, ArH), 7.01 (1H, ddd, *J* = 8.0, 7.9, 0.4 Hz, ArH), 3.33 (1H, ddd, *J* = 11.6, 10.8, 3.7 Hz, CH_2_), 2.92 (1H, ddd, *J* = 12.1, 10.8, 5.2 Hz, CH_2_), 2.71 (1H, ddd, *J* = 14.2, 12.1, 3.7 Hz, CH_2_), 2.40–2.15 (1H, m, CH_2_), 2.35 (3H, s, ArCH_3_), 2.33 (6H, s, N(CH_3_)_2_), 1.53 (9H, s, *t*Bu); ^13^C{^1^H} NMR (101 MHz, CDCl_3_) *δ* 167.4, 139.8, 135.6, 131.7, 127.8, 127.6, 126.0, 82.4, 72.4, 40.3, 40.1, 37.5, 28.4, 21.8; HRMS (ESI): calcd for C_17_H_26_BrClNO_2_ [M + H]^+^ 390.0830, found 390.0826.

### 1-Allyl-2-(5-bromo-2-methylphenyl)-2-(*tert*-butoxycarbonyl)-1-methylazetidin-1-ium trifluoromethanesulfonate (5)

A solution of allyl alcohol (50 μL, 0.74 mmol) and pyridine (55 μL, 0.68 mmol) in CCl_4_ (1.7 mL) was treated with trifluoromethanesulfonic anhydride (0.11 mL, 0.65 mmol) at 0 °C. The mixture was stirred for 20 min at the same temperature to precipitate a pale-brown solid. The generated allyl triflate in CCl_4_ (ref. 12) was added by decantation to a mixture of 1b^6^ (156 mg, 0.458 mmol) and NaHCO_3_ (122 mg, 1.45 mmol) in CH_2_Cl_2_ (2.3 mL) at 0 °C. After stirring for 1 h at room temperature, the resulting mixture was purified by chromatography on silica gel (CH_2_Cl_2_/MeOH = 20/1 to 10/1 as the eluent) to obtain 5 (170 mg, 70% yield, 8/2 mixture of diastereomers) as a colourless gum. IR (ATR) *ν*_max_/cm^−1^ 2980, 2936, 1731, 1484, 1459, 1427, 1397, 1372, 1252, 1223, 1149, 1139, 1029, 952, 911, 831, 808, 790, 755, 728; ^1^H NMR (400 MHz, acetone-*d*_6_) *δ* 7.81 (0.8H, d, *J* = 2.0 Hz, ArH), 7.76 (0.2H, br, ArH), 7.70–7.61 (0.2H, m, ArH), 7.65 (0.8H, dd, *J* = 8.2, 2.0 Hz, ArH), 7.36 (1H, d, *J* = 8.2 Hz, ArH), 6.19 (0.2H, ddt, *J* = 17.0, 9.6, 7.2 Hz, CH_2_CH=CH_2_), 6.05 (0.8H, ddt, *J* = 17.1, 10.1, 7.2 Hz, CH_2_CH=CH_2_), 5.86 (0.2H, ddt, *J* = 17.0, 1.2, 1.2 Hz, CH_2_CH=C*H*_2_), 5.76–5.70 (0.2H, m, CH_2_CH=C*H*_2_), 5.74 (0.8H, ddt, *J* = 17.1, 1.4, 1.2 Hz, CH_2_CH=CH_2_), 5.65 (0.8H, ddt, *J* = 10.1, 1.4, 0.8 Hz, CH_2_CH=CH_2_), 4.90 (0.2H, ddd, *J* = 9.6, 9.6, 9.6 Hz, 4H), 4.61 (0.8H, dddd, *J* = 10.6, 10.6, 9.2, 1.6 Hz, 4H), 4.48–4.32 (0.2H, br, CH_2_), 4.38 (0.8H, ddd, *J* = 10.3, 9.2, 3.2 Hz, 4H), 4.24–4.09 (1.8H, m, CH_2_), 4.09–4.00 (0.2H, br m, CH_2_), 3.82–3.66 (0.4H, br m, CH_2_), 3.71 (3H, s, NCH_3_), 3.24–3.04 (1.6H, br m, CH_2_), 2.42 (2.4H, s, ArCH_3_), 2.41 (0.6H, s, ArCH_3_), 1.473 (1.8H, s, *t*Bu), 1.466 (7.2H, s, *t*Bu); ^13^C{^1^H} NMR (101 MHz, acetone-*d*_6_) *δ* 167.2, 137.8, 137.4 (minor), 135.2, 134.5, 133.2, 133.0, 129.8, 128.9 (minor), 126.3 (minor), 125.9, 122.7 (q, *J* = 324 Hz), 120.6, 87.8, 87.7 (minor), 87.5, 64.0, 59.3, 48.2 (minor), 47.9, 27.8, 27.64 (minor), 27.63, 21.1; HRMS (ESI): calcd for C_19_H_27_BrNO_2_ [M + H]^+^ 380.1220, found 380.1207.

### *tert*-Butyl 4-(allyl(methyl)amino)-2-(5-bromo-2-methylphenyl)-2-fluorobutanoate (6)

Performed by the same procedure with 3aa using 5 (97.5 mg, 0.184 mmol) as a substrate. Purification by chromatography on silica gel (CH_2_Cl_2_/MeOH = 30/1 to 20/1 as the eluent) gave 6 (60.1 mg, 82% yield) as a pale yellow oil. IR (ATR) *ν*_max_/cm^−1^ 3076, 2977, 2934, 2874, 2846, 2780, 1749, 1731, 1644, 1592, 1564, 1481, 1456, 1392, 1368, 1282, 1251, 1142, 1054, 1033, 995, 920, 875, 841, 810, 771, 748, 705; ^1^H NMR (400 MHz, CDCl_3_) *δ* 7.54 (1H, dd, *J* = 1.9, 1.9 Hz, ArH), 7.34 (1H, dd, *J* = 8.1, 1.9 Hz, ArH), 7.03 (1H, d, *J* = 8.1 Hz, ArH), 5.86 (1H, ddt, *J* = 17.2, 10.0, 6.6 Hz, CH_2_CH=CH_2_), 5.22–5.12 (2H, m, CH_2_CH=CH_2_), 3.04 (2H, ddd, *J* = 6.6, 1.1, 1.1 Hz, C*H*_2_CH=CH_2_), 2.66–2.44 (3H, m, CH_2_), 2.44–2.28 (1H, m, CH_2_), 2.36 (3H, d, ^5^*J*_FH_ = 3.6 Hz, ArCH_3_), 2.27 (3H, s, NCH_3_), 1.43 (9H, s, *t*Bu); ^13^C{^1^H} NMR (101 MHz, CDCl_3_) *δ* 168.5 (d, ^2^*J*_FC_ = 27 Hz), 138.3 (d, ^2^*J*_FC_ = 22 Hz), 135.6 (d, ^3^*J*_FC_ = 2 Hz), 135.2, 133.6, 131.4 (d, *J*_FC_ = 2 Hz), 129.2 (d, ^3^*J*_FC_ = 8 Hz), 119.3, 117.9, 95.8 (d, ^1^*J*_FC_ = 190 Hz), 83.0, 61.0, 50.8 (d, ^3^*J*_FC_ = 5 Hz), 42.1, 34.2 (d, ^2^*J*_FC_ = 22 Hz), 27.7, 20.2 (d, ^4^*J*_FC_ = 8 Hz); HRMS (ESI): calcd for C_19_H_28_BrFNO_2_ [M + H]^+^ 400.1282, found 400.1273.

### *tert*-Butyl 2-(5-bromo-2-methylphenyl)-2-fluoro-4-(methylamino)butanoate (7)

A mixture of 6 (103 mg, 0.257 mmol) and RhCl(PPh_3_)_3_ (5 mg, 0.005 mmol) in MeCN (2.2 mL) and H_2_O (0.4 mL) was refluxed for 3 h under an Ar atmosphere. The resulting mixture was cooled to room temperature and treated with saturated aqueous NaHCO_3_. The mixture was extracted with EtOAc and the combined extracts were washed with brine. The organic solution was dried over Na_2_SO_4_ and concentrated by evaporation. Purification of the residue by chromatography on silica gel (CH_2_Cl_2_/MeOH = 15/1 to 5/1 as the eluent) gave 7 (77.7 mg, 84% yield) as pale yellow crystals, mp 38–42 °C. IR (ATR) *ν*_max_/cm^−1^ 3040, 2981, 2942, 2887, 1733, 1499, 1461, 1425, 1397, 1372, 1357, 1327, 1254, 1223, 1141, 1078, 1029, 958, 930, 877, 837, 802, 768, 754, 736, 706; ^1^H NMR (400 MHz, CDCl_3_) *δ* 7.53 (1H, dd, *J* = 1.9, 1.9 Hz, ArH), 7.35 (1H, dd, *J* = 8.1, 1.9 Hz, ArH), 7.04 (1H, d, *J* = 8.1 Hz, ArH), 2.78 (1H, ddd, *J* = 12.0, 9.2, 5.8 Hz, CH_2_), 2.72 (1H, ddd, *J* = 12.0, 9.2, 5.8 Hz, CH_2_), 2.64–2.33 (2H, m, CH_2_), 2.45 (3H, s, NCH_3_), 2.36 (3H, d, ^5^*J*_FH_ = 3.6 Hz, ArCH_3_), 1.59 (1H, br, NH), 1.44 (9H, s, *t*Bu); ^13^C{^1^H} NMR (101 MHz, CDCl_3_) *δ* 168.6 (d, ^2^*J*_FC_ = 27 Hz), 138.3 (d, ^2^*J*_FC_ = 21 Hz), 135.5 (d, ^3^*J*_FC_ = 2 Hz), 133.7, 131.4 (d, *J*_FC_ = 2 Hz), 129.1 (d, ^3^*J*_FC_ = 9 Hz), 119.3, 96.2 (d, ^1^*J*_FC_ = 189 Hz), 83.2, 46.2 (d, ^3^*J*_FC_ = 5 Hz), 36.43 (d, ^2^*J*_FC_ = 22 Hz), 36.36, 27.7, 20.2 (d, ^4^*J*_FC_ = 7 Hz); HRMS (ESI): calcd for C_16_H_24_BrFNO_2_ [M + H]^+^ 360.0969, found 360.0964.

### 4-(*tert*-Butoxy)-3-fluoro-*N*,*N*,*N*-trimethyl-4-oxo-3-(*o*-tolyl)butan-1-aminium iodide (8-I)

A mixture of 3aa (67.0 mg, 0.227 mmol) and MeI (21 μL, 0.34 mmol) in MeCN (1.1 mL) was stirred for 2 h at room temperature. The resulting mixture was concentrated by evaporation and the residue was purified by chromatography on silica gel (CH_2_Cl_2_/MeOH = 20/1 to 10/1 as the eluent) to obtain 8-I (90.3 mg, 91% yield) as a yellow solid, mp 162–164 °C. IR (ATR) *ν*_max_/cm^−1^ 3002, 2978, 2932, 1738, 1484, 1455, 1418, 1394, 1368, 1335, 1294, 1259, 1243, 1215, 1145, 1089, 1062, 1009, 992, 975, 934, 912, 838, 777, 748; ^1^H NMR (400 MHz, CDCl_3_) *δ* 7.51–7.44 (1H, m, ArH), 7.32–7.23 (2H, m, ArH), 7.22–7.15 (1H, m, ArH), 3.70 (1H, ddd, *J* = 13.1, 8.7, 7.3 Hz, CH_2_), 3.60–3.40 (1H, m, CH_2_), 3.52 (9H, s, N(CH_3_)_3_), 2.93–2.76 (2H, m, CH_2_), 2.41 (3H, d, ^5^*J*_FH_ = 3.6 Hz, ArCH_3_), 1.43 (9H, s, *t*Bu); ^13^C{^1^H} NMR (101 MHz, CDCl_3_) *δ* 167.7 (d, ^2^*J*_FC_ = 25 Hz), 135.7 (d, ^3^*J*_FC_ = 2 Hz), 133.8 (d, ^2^*J*_FC_ = 21 Hz), 132.5, 129.2 (d, *J*_FC_ = 2 Hz), 126.4 (d, *J*_FC_ = 2 Hz), 126.1 (d, ^3^*J*_FC_ = 9 Hz), 96.1 (d, ^1^*J*_FC_ = 190 Hz), 84.4, 62.7–62.4 (m), 54.0, 30.3 (d, ^2^*J*_FC_ = 22 Hz), 27.8, 20.7 (d, ^4^*J*_FC_ = 7 Hz); ^19^F NMR (376 MHz, CDCl_3_) *δ* −157; HRMS (ESI): calcd for C_18_H_29_FNO_2_ [M − I]^+^ 310.2177, found 310.2166.

### 4-(*tert*-Butoxy)-3-fluoro-*N*,*N*,*N*-trimethyl-4-oxo-3-(*o*-tolyl)butan-1-aminium trifluoromethanesulfonate (8-OTf)

A mixture of 3aa (91.3 mg, 0.309 mmol) and NaHCO_3_ (82 mg, 0.98 mmol) in CH_2_Cl_2_ (1.5 mL) was treated with methyl trifluoromethanesulfonate (52 μL, 0.46 mmol) at 0 °C and stirred for 2 h at room temperature. The resulting mixture was purified by chromatography on silica gel (CH_2_Cl_2_/MeOH = 20/1 to 10/1 as the eluent) to obtain 8-OTf (143 mg, quant.) as a white solid, mp 140–142 °C. IR (ATR) *ν*_max_/cm^−1^ 3039, 2979, 2936, 1760, 1733, 1484, 1459, 1420, 1395, 1371, 1257, 1227, 1155, 1084, 1030, 1008, 976, 933, 910, 841, 793, 746; ^1^H NMR (400 MHz, CDCl_3_) *δ* 7.42–7.35 (1H, m, ArH), 7.30–7.21 (2H, m, ArH), 7.21–7.13 (1H, m, ArH), 3.55–3.43 (1H, m, CH_2_), 3.41–3.27 (1H, m, CH_2_), 3.21 (9H, s, N(CH_3_)_3_), 2.86–2.69 (2H, m, CH_2_), 2.38 (3H, d, ^5^*J*_FH_ = 3.6 Hz, ArCH_3_), 1.41 (9H, s, *t*Bu); ^13^C{^1^H} NMR (101 MHz, CDCl_3_) *δ* 167.8 (d, ^2^*J*_FC_ = 26 Hz), 135.9 (d, ^3^*J*_FC_ = 2 Hz), 134.0 (d, ^2^*J*_FC_ = 21 Hz), 132.4, 129.2, 126.3, 125.9 (d, ^3^*J*_FC_ = 9 Hz), 120.5 (q, ^1^*J*_FC_ = 321 Hz), 95.9 (d, ^1^*J*_FC_ = 190 Hz), 84.3, 62.1 (d, ^3^*J*_FC_ = 5 Hz), 53.3, 30.0 (d, ^2^*J*_FC_ = 22 Hz), 27.6, 20.5 (d, ^4^*J*_FC_ = 8 Hz); ^19^F NMR (376 MHz, CDCl_3_) *δ* −80, −157; HRMS (ESI): calcd for C_18_H_29_FNO_2_ [M − OTf]^+^ 310.2177, found 310.2171.

### *tert*-Butyl 2-fluoro-2-(*o*-tolyl)but-3-enoate (9)

A mixture of 8-OTf (67.9 mg, 0.148 mmol) and DBU (66 μL, 0.44 mmol) in toluene (1.5 mL) was refluxed for 1 day under an Ar atmosphere. The resulting mixture was cooled to room temperature and treated with aqueous saturated NH_4_Cl. The mixture was extracted with EtOAc and the combined extracts were washed with saturated aqueous NaHCO_3_ followed by brine. The solution was dried over Na_2_SO_4_ and concentrated by evaporation. The residue was purified by chromatography on silica gel (*n*-hexane/EtOAc = 30/1 to 20/1 as the eluent) to obtain 9 (21.0 mg, 57% yield) as a colourless oil. IR (ATR) *ν*_max_/cm^−1^ 2979, 2934, 1751, 1731, 1476, 1457, 1406, 1394, 1369, 1282, 1253, 1223, 1146, 1119, 1061, 1042, 1024, 992, 973, 935, 841, 828, 800, 769, 747; ^1^H NMR (400 MHz, CDCl_3_) *δ* 7.40–7.35 (1H, m, ArH), 7.29–7.22 (1H, m, ArH), 7.21–7.14 (2H, m, ArH), 6.37 (1H, ddd, ^3^*J*_FH_ = 17.6, *J* = 17.4, 11.1 Hz, 3H), 5.60 (1H, ddd, ^4^*J*_FH_ = 0.8 Hz, *J* = 17.4, 0.8 Hz, 4H), 5.51 (1H, ddd, ^4^*J*_FH_ = 1.6 Hz, *J* = 11.1, 0.8 Hz, 4H), 2.40 (3H, d, ^5^*J*_FH_ = 2.8 Hz, ArCH_3_), 1.46 (9H, s, *t*Bu); ^13^C{^1^H} NMR (101 MHz, CDCl_3_) *δ* 168.6 (d, ^2^*J*_FC_ = 26 Hz), 137.2, 136.1 (d, ^2^*J*_FC_ = 22 Hz), 134.7 (d, ^2^*J*_FC_ = 23 Hz), 131.8, 129.0, 127.9 (d, ^3^*J*_FC_ = 6 Hz), 125.5, 117.7 (d, ^3^*J*_FC_ = 12 Hz), 96.1 (d, ^1^*J*_FC_ = 190 Hz), 83.0, 27.8, 20.1 (d, ^4^*J*_FC_ = 5 Hz); ^19^F NMR (376 MHz, CDCl_3_) *δ* −153; HRMS (ESI): calcd for C_15_H_19_FO_2_Na [M + Na]^+^ 273.1261, found 273.1260.

### (*S*)-2-(*tert*-Butoxycarbonyl)-1,1-dimethyl-2-(*o*-tolyl)azetidin-1-ium trifluoromethanesulfoante [(*S*)-2a]

A mixture of (*S*)-1a (171 mg, 0.654 mmol, 93% ee) and NaHCO_3_ (168 mg, 2.00 mmol) in CH_2_Cl_2_ (3.3 mL) was treated with methyl trifluoromethanesulfonate (148 μL, 1.31 mmol) at 0 °C and stirred for 2.5 h at room temperature. The resulting mixture was evaporated to *ca.* 1/2 volume and purified by chromatography on silica gel (CH_2_Cl_2_/MeOH = 15/1 to 6/1 as the eluent) to obtain (*S*)-2a (277 mg, quant.) as colourless crystals, mp 110–112 °C. [*α*]^24^_589_ +34.6 (*c* 1.0 in CHCl_3_); IR (ATR) *ν*_max_/cm^−1^ 3076, 3041, 2979, 2940, 1728, 1459, 1395, 1371, 1302, 1254, 1225, 1145, 1105, 1078, 1030, 995, 969, 946, 856, 832, 770, 753, 728, 988, 965, 939, 844, 749; ^1^H NMR (400 MHz, CDCl_3_) *δ* 7.45–7.33 (3H, m, ArH), 7.29–7.23 (1H, m, ArH), 4.52 (1H, ddd, *J* = 10.6, 10.4, 9.4 Hz, 4H), 4.27 (1H, ddd, *J* = 9.6, 9.4, 2.4 Hz, 4H), 3.90 (1H, ddd, *J* = 12.4, 10.6, 9.6 Hz, 3H), 3.63 (3H, s, NCH_3_), 3.02–2.84 (1H, br m, 3H), 2.97 (3H, s, NCH_3_), 2.33 (3H, s, ArCH_3_), 1.39 (9H, s, *t*Bu); ^13^C{^1^H} NMR (101 MHz, CDCl_3_) *δ* 166.4, 135.9, 132.5, 130.6, 129.7, 128.5, 126.9, 120.6 (q, *J* = 322 Hz), 86.8, 86.3, 62.7, 50.9, 50.7, 27.4, 27.1, 20.7; HRMS (ESI): calcd for C_17_H_26_NO_2_ [M − OTf]^+^ 276.1958, found 276.1955.

### (*R*)-*tert*-Butyl 4-(dimethylamino)-2-fluoro-2-(*o*-tolyl)butanoate [(*R*)-3aa]

Obtained from (*S*)-2a (277 mg, 0.651 mmol) by the same procedure with 3aa. Purification by chromatography on silica gel (CH_2_Cl_2_/MeOH = 20/1 to 10/1 as the eluent) gave (*R*)-3aa (131 mg, 68% yield) as a colourless oil. [*α*]^24^_589_ −5.2 (*c* 1.0 in EtOH).

### (*R*)-4-(Dimethylamino)-2-fluoro-2-(*o*-tolyl)butan-1-ol [(*R*)-10]

A solution of (*R*)-3aa (131 mg, 0.443 mmol) in THF (2.2 mL) was added to a suspension of LiAlH_4_ (35 mg, 0.92 mmol) in THF (2.2 mL) at 0 °C under an Ar atmosphere. After stirring for 4 h at room temperature, the resulting mixture was cooled at 0 °C and diluted with Et_2_O (4 mL). The mixture was quenched at 0 °C by addition of H_2_O (35 μL), 15 wt% NaOH·H_2_O solution (35 μL), and H_2_O (105 μL). The suspension was diluted with EtOH (4 mL) and stirred for 12 h at room temperature. The resulting mixture was filtered through a pad of Celite and the filtrate was concentrated by evaporation. The residue was purified by chromatography on amino-functionalized silica gel (Chromatorex NH-DM1020, *n*-hexane/EtOAc = 2/1 to 1/1 as the eluent) to obtain (*R*)-10 (73.0 mg, 73% yield) as a colourless oil. [*α*]^24^_589_ +9.9 (*c* 1.0 in EtOH); IR (ATR) *ν*_max_/cm^−1^ 3339, 3061, 3020, 2947, 2863, 2825, 2778, 1462, 1385, 1312, 1290, 1258, 1217, 1180, 1163, 1095, 1057, 1038, 1000, 944, 878, 846, 800, 757, 726; ^1^H NMR (400 MHz, CDCl_3_) *δ* 7.47–7.40 (1H, m, ArH), 7.23–7.10 (3H, m, ArH), 3.92 (1H, dd, ^3^*J*_FH_ = 26.8 Hz, *J* = 12.8 Hz, 1H), 3.88 (1H, dd, ^3^*J*_FH_ = 20.2 Hz, *J* = 12.8 Hz, 1H), 2.87–2.76 (1H, m, OH), 2.48 (3H, d, ^5^*J*_FH_ = 4.4 Hz, ArCH_3_), 2.45–2.13 (4H, m, CH_2_), 2.31 (6H, s, N(CH_3_)_2_); ^13^C{^1^H} NMR (101 MHz, CDCl_3_) *δ* 139.9 (d, ^2^*J*_FC_ = 20 Hz), 135.2 (d, ^3^*J*_FC_ = 2 Hz), 132.5, 127.7, 125.7 (d, *J*_FC_ = 2 Hz), 125.5 (d, ^3^*J*_FC_ = 11 Hz), 99.4 (d, ^1^*J*_FC_ = 179 Hz), 67.6 (d, ^2^*J*_FC_ = 28 Hz), 53.5 (d, ^3^*J*_FC_ = 6 Hz), 44.9, 35.2 (d, ^2^*J*_FC_ = 25 Hz), 21.8 (d, ^4^*J*_FC_ = 9 Hz); ^19^F NMR (376 MHz, CDCl_3_) *δ* −161; HRMS (ESI): calcd for C_13_H_21_FNO [M + H]^+^ 226.1602, found 226.1600.

### (*R*)-4-(Dimethylamino)-2-fluoro-2-(*o*-tolyl)butyl benzoate [(*R*)-11]

A solution of (*R*)-10 (73.0 mg, 0.324 mmol) and Et_3_N (135 μL, 0.969 mmol) in CH_2_Cl_2_ (3.2 mL) was treated with benzoyl chloride (45 μL, 0.39 mmol) at 0 °C and stirred for 2 h at room temperature. The resulting mixture was diluted with H_2_O and extracted with EtOAc. The combined extracts were washed with H_2_O, dried over Na_2_SO_4_, and concentrated by evaporation. Purification by chromatography on silica gel (CH_2_Cl_2_/MeOH = 20/1 to 10/1 as the eluent) gave (*R*)-11 (101 mg, 95% yield) as a colourless oil. 93% ee [determined by HPLC analysis: Daicel Chiralcel AD-H column (25 cm), *n*-hexane/EtOH/Et_2_NH = 100/2/0.1 as the eluent, flow rate: 0.50 mL min^−1^, *t*R = 14.7 min for (*R*)-11 (96.5%) and 19.1 min for (*S*)-11 (3.5%)]. [*α*]^23^_589_ +6.6 (*c* 1.0 in EtOH); IR (ATR) *ν*_max_/cm^−1^ 3062, 3022, 2970, 2944, 2861, 2818, 2765, 1719, 1602, 1584, 1491, 1450, 1377, 1314, 1265, 1176, 1156, 1111, 1095, 1068, 1042, 1026, 936, 893, 849, 802, 760, 727, 708; ^1^H NMR (400 MHz, CDCl_3_) *δ* 7.98–7.93 (2H, m, ArH), 7.54 (1H, tt, *J* = 7.6, 1.3 Hz, ArH), 7.43–7.35 (3H, m, ArH), 7.25–7.15 (3H, m, ArH), 4.73 (1H, dd, ^3^*J*_FH_ = 17.4 Hz, *J* = 12.6 Hz, 1H), 4.67 (1H, dd, ^3^*J*_FH_ = 15.8 Hz, *J* = 12.6 Hz, 1H), 2.50 (3H, d, ^5^*J*_FH_ = 3.6 Hz, ArCH_3_), 2.50–2.33 (3H, m, CH_2_), 2.25–2.09 (1H, m, CH_2_), 2.20 (6H, s, N(CH_3_)_2_); ^13^C{^1^H} NMR (101 MHz, CDCl_3_) *δ* 166.1, 136.5 (d, ^2^*J*_FC_ = 21 Hz), 134.8 (d, ^3^*J*_FC_ = 2 Hz), 133.1, 132.6, 129.7, 129.6, 128.3, 128.1, 126.1 (d, ^3^*J*_FC_ = 14 Hz), 125.9 (d, *J*_FC_ = 2 Hz), 98.8 (d, ^1^*J*_FC_ = 180 Hz), 68.4 (d, ^2^*J*_FC_ = 25 Hz), 53.7 (d, ^3^*J*_FC_ = 3 Hz), 45.5, 33.7 (d, ^2^*J*_FC_ = 24 Hz), 21.8 (d, ^4^*J*_FC_ = 8 Hz); ^19^F NMR (376 MHz, CDCl_3_) *δ* −157; HRMS (ESI): calcd for C_20_H_25_FNO_2_ [M + H]^+^ 330.1864, found 330.1859.

### *tert*-Butyl 2-(benzyl(methyl)amino)-2-ethyl-4-fluorobutanoate (14) and *tert*-butyl 4-(benzyl(methyl)amino)-2-ethyl-2-hydroxybutanoate (15)

Obtained from 12 (141 mg, 0.321 mmol) by the same procedure with 3aa and 4aa. Purification by chromatography on silica gel (CH_2_Cl_2_/MeOH = 100/0 to 30/1 as the eluent *R*_f_: 14 > 15) gave 14 (62.0 mg, 62% yield) as a colourless oil and 15 (7.2 mg, 7% yield) as a colourless oil. 14: IR (ATR) *ν*_max_/cm^−1^ 3086, 3062, 3027, 2975, 2934, 2882, 2801, 1715, 1604, 1495, 1454, 1391, 1366, 1238, 1165, 1124, 1064, 1029, 999, 961, 940, 888, 847, 831, 773, 732, 697; ^1^H NMR (400 MHz, CDCl_3_) *δ* 7.34–7.27 (4H, m, Ph), 7.26–7.18 (1H, m, Ph), 4.66 (1H, dddd, ^2^*J*_FH_ = 47.2 Hz, *J* = 9.2, 7.2, 7.2 Hz, 4H), 4.62 (1H, dddd, ^2^*J*_FH_ = 47.2 Hz, *J* = 9.2, 7.2, 5.6 Hz, 4H), 3.72 (1H, d, *J* = 14.4 Hz, C*H*_2_Ph), 3.65 (1H, d, *J* = 14.4 Hz, C*H*_2_Ph), 2.38–2.11 (2H, m, 3H), 2.20 (3H, s, NCH_3_), 1.97 (1H, dq, *J* = 14.0, 7.4 Hz, C*H*_2_CH_3_), 1.72 (1H, dq, *J* = 14.0, 7.4 Hz, C*H*_2_CH_3_), 1.52 (9H, s, *t*Bu), 0.95 (3H, dd, *J* = 7.4, 7.4 Hz, CH_2_C*H*_3_); ^13^C{^1^H} NMR (101 MHz, CDCl_3_) *δ* 172.6, 140.8, 128.2, 128.0, 126.6, 81.2 (d, ^1^*J*_FC_ = 163 Hz), 81.1, 67.4 (d, ^3^*J*_FC_ = 6 Hz), 55.3, 35.3, 31.4 (d, ^2^*J*_FC_ = 20 Hz), 28.3, 25.9, 8.7; ^19^F NMR (376 MHz, CDCl_3_) *δ* −221; HRMS (ESI): calcd for C_18_H_29_FNO_2_ [M + H]^+^ 310.2177, found 310.2167. 15: IR (ATR) *ν*_max_/cm^−1^ 3408, 3087, 3063, 3028, 2973, 2930, 2880, 2801, 1715, 1603, 1495, 1454, 1391, 1366, 1298, 1244, 1162, 1134, 1101, 1069, 1022, 951, 909, 892, 845, 829, 776, 729, 697; ^1^H NMR (400 MHz, CDCl_3_) *δ* 7.35–7.27 (4H, m, Ph), 7.27–7.21 (1H, m, Ph), 5.28 (1H, br, OH), 3.92 (1H, ddd, *J* = 11.3, 11.2, 3.2 Hz, CH_2_), 3.784 (1H, ddd, *J* = 11.2, 5.2, 3.2 Hz, CH_2_), 3.775 (2H, s, C*H*_2_Ph), 2.35 (1H, dddd, *J* = 15.6, 11.3, 5.2, 1.2 Hz, CH_2_), 2.27–2.15 (1H, m, C*H*_2_CH_3_), 2.23 (3H, s, NCH_3_), 1.90 (1H, dq, *J* = 13.4, 7.4 Hz, C*H*_2_CH_3_), 1.84 (1H, ddd, *J* = 15.6, 3.2, 3.2 Hz, CH_2_), 1.54 (9H, s, *t*Bu), 0.89 (3H, dd, *J* = 7.4, 7.4 Hz, CH_2_C*H*_3_); ^13^C{^1^H} NMR (101 MHz, CDCl_3_) *δ* 172.2, 139.1, 128.9, 128.5, 127.2, 81.5, 69.8, 59.5, 55.6, 35.2, 29.7, 28.4, 25.7, 9.4; HRMS (ESI): calcd for C_18_H_30_NO_3_ [M + H]^+^ 308.2220, found 308.2214.

## Author contributions

E. T. was supervisor of this project and conducted all area of this work, idea, development of the methodology, a part of experiments and writing the manuscript. K. K. performed the main experiments and compounds analyses.

## Conflicts of interest

There are no conflicts to declare.

## Supplementary Material

RA-011-D1RA08706A-s001
