# Supplementary material for: Synthesis of tertiary alkyl fluorides and chlorides by site-selective nucleophilic ring-opening reaction of α-aryl azetidinium salts
Source: RSC Adv. 2021 Dec 13;11(62):39607–18. doi: 10.1039/d1ra08706a (PMC9044468; doi:10.1039/d1ra08706a)
Supplement: RA-011-D1RA08706A-s001 [file RA-011-D1RA08706A-s001.pdf]

Electronic Supplementary Material (ESI) for RSC Advances.
This journal is © The Royal Society of Chemistry 2021

Synthesis of tertiary alkyl fluorides and chlorides by site-selective nucleophilic ring-opening reaction of α-aryl azetidinium salts

Eiji Tayama and Kohei Kawai
Department of Chemistry, Faculty of Science, Niigata University 950-2181, Japan
E-mail: tayama@chem.sc.niigata-u.ac.jp

Electronic Supplementary Information

Contents:

1. HPLC chromatogram for determination of enantiomeric excess (ee) S1–2

2. Preparation of substrates S3–12

3. Copies of ^{1}H, ^{13}C and ^{19}F NMR (representative) spectra of substrates and products S13–62

1. HPLC chromatogram for determination of enantiomeric excess (ee)

The ee were determined by HPLC analysis using chiral column in comparison with the racemic compounds.

(*S*)-**1a**: 93% ee, Daicel Chiralcel OJ-H column (25 cm), *n*-hexane/EtOH = 99/1 as the eluent, flow rate = 0.50 mL/min, t_R = 8.1 min for (*S*)-**1a** (96.7%) and 18.6 min for (*R*)-**1a** (3.3%).

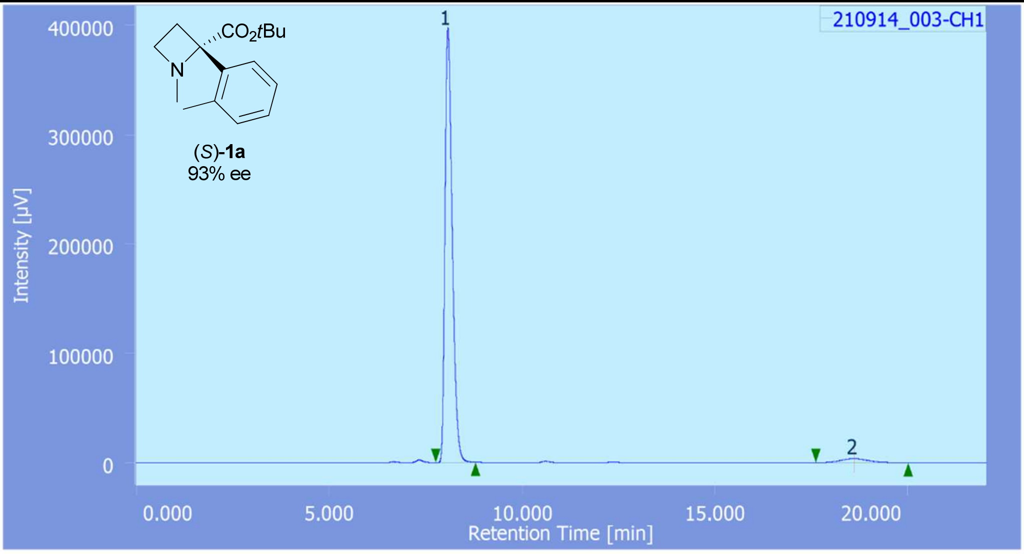

column OJ–H, 0.5mL/min
date 2021/09/14 7:14:48
solvent Hexane–ethanol(99–1)

#	tR [min]	area [µV·sec]	area%
1	8.077	5205819	96.693
2	18.590	178029	3.307

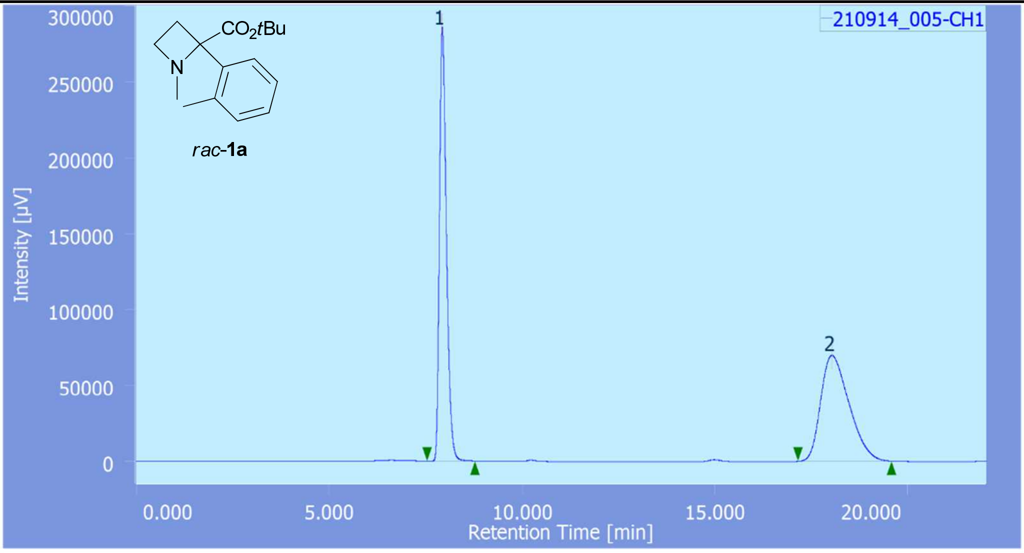

column OJ–H, 0.5mL/min
date 2021/09/14 8:02:59
solvent Hexane–ethanol(99–1)

#	tR [min]	area [µV·sec]	area%
1	7.932	3602006	50.355
2	18.013	3551203	49.645

(*R*)-**11**: 93% ee, Daicel Chiralpak AD-H column (25 cm), *n*-hexane/EtOH/Et_2NH = 100/2/0.1 as the eluent, flow rate = 0.50 mL/min, t_R = 14.7 min for (*R*)-**11** (96.5%) and 19.1 min for (*S*)-**11** (3.5%).

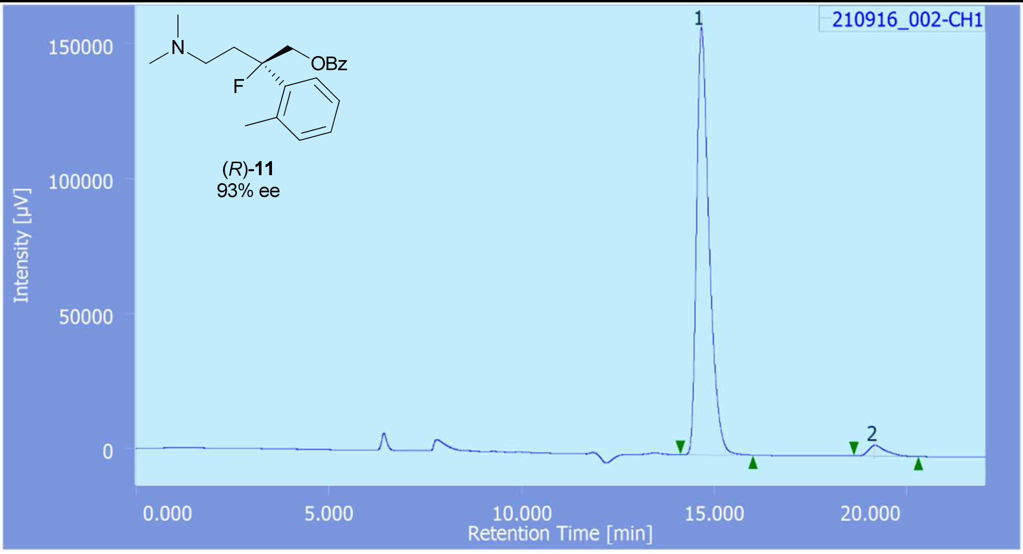

column AD-H, 0.5mL/min
date 2021/09/16 15:27:06
solvent Line D(100)_0.5mL

#	tR [min]	area [μV·sec]	area%
1	14.650	3579478	96.538
2	19.142	128358	3.462

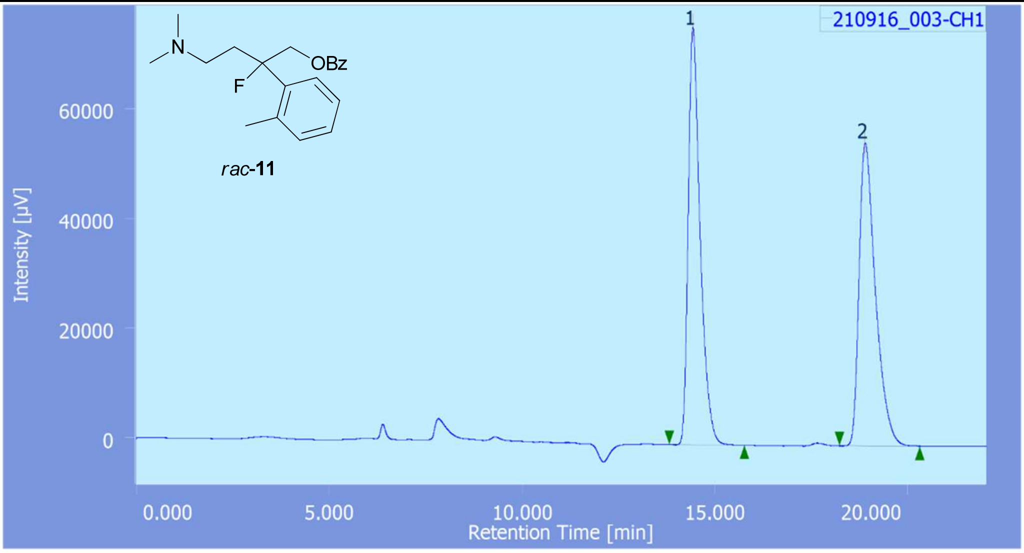

column AD-H, 0.5mL/min
date 2021/09/16 15:51:27
solvent Line D(100)_0.5mL

#	tR [min]	area [μV·sec]	area%
1	14.415	1637326	50.100
2	18.872	1630767	49.900

2. Preparation of substrates

2-1. Representative procedure for preparation of 2-(*tert*-butoxycarbonyl)-1,1-dimethyl-2-(*o*-tolyl)azetidin-1-ium trifluoromethanesulfonate (**2a**)[1]

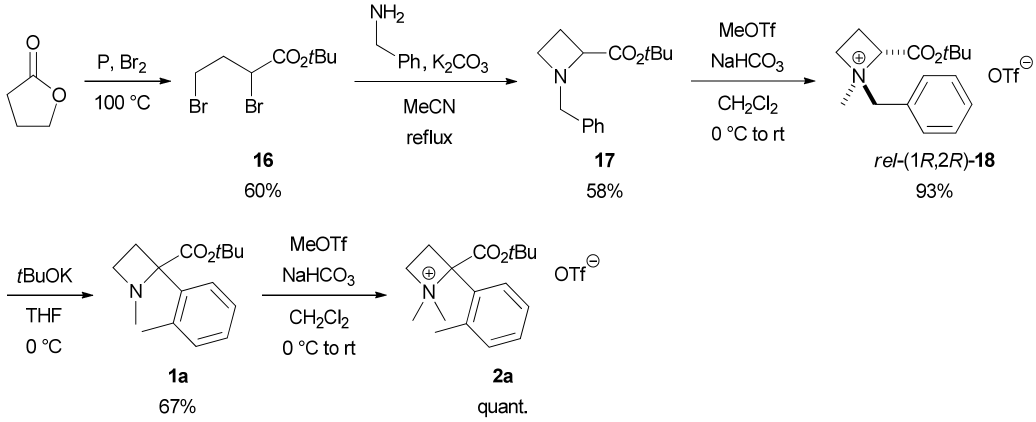

(Step 1) γ-Butyrolactone (6.0 mL, 78 mmol) and red-phosphorus (50 mg, 1.6 mmol) in a two-neck round-bottom flask was stirred at 100 °C under an Ar atmosphere. Br_2 (4.3 mL, 84 mmol) was added dropwise for 1 h to the mixture with stirring. The resulting mixture was cooled to room temperature and excess Br_2 was removed by flow of air. The residue was dissolved in CH_2Cl_2 (26 mL) and treated with *conc*. H_2SO_4 (0.4 mL). Isobutene gas (excess) was added to the solution in the reaction flask which equipped with a dry ice condenser. The solution was stirred for 2 days at room temperature. The resulting mixture was treated with saturated aqueous $NaHCO_3$ and extracted with CH_2Cl_2. The combined extracts were washed with saturated aqueous NaBr, dried over Na_2SO_4, and concentrated by evaporation. Purification of the residue by chromatography on silica gel (*n*-hexane/EtOAc = 30/1 to 20/1 as the eluent) gave *tert*-butyl 2,4-dibromobutanoate (**16**) (14.07 g, 60% yield) as a colourless oil. The product included impurities observed by ^{1}H NMR analysis. (Step 2) A mixture of benzylamine (276 μL, 2.53 mmol), **16** (764 mg, 2.53 mmol), and K_2CO_3 (1.05 g, 7.60 mmol) in MeCN (13 mL) was refluxed for 8 h. The resulting mixture was cooled to room temperature and filtered. The filtrate was concentrated by evaporation and the residue was purified by chromatography on silica gel (*n*-hexane/EtOAc = 5/1 to 3/1 as the eluent) to obtain *tert*-butyl 1-benzylazetidine-2-carboxylate (**17**)[1] (360 mg, 58% yield) as a pale yellow oil. (Step 3) A mixture of **17** (360 mg, 1.46 mmol) and $NaHCO_3$ (0.37 g, 4.4 mmol) in CH_2Cl_2 (7.3 mL) was treated with methyl trifluoromethanesulfonate (0.25 mL, 2.2 mmol) at 0 °C and stirred for 1 h at room temperature. The mixture was concentrated by evaporation to ca. 1/2 volume and purified by chromatography on silica gel (CH_2Cl_2/MeOH = 15/1 to 6/1 as the eluent) to obtain *rel*-(1*R*,2*R*)-1-benzyl-2-(*tert*-butoxycarbonyl)-1-methylazetidin-1-ium trifluoromethanesulfonate [*rel*-(1*R*,2*R*)-**18**][1] (556 mg, 93% yield) as colourless crystals. (Step 4) A 1.0 M *t*BuOK THF solution (1.6 mL, 1.6 mmol) was added to a solution of *rel*-(1*R*,2*R*)-**18** (556 mg, 1.35 mmol) in THF (12 mL) at 0 °C under an Ar atmosphere. After stirring for 3 h at the same temperature, the resulting mixture was quenched with saturated aqueous NH_4Cl and extracted with EtOAc. The combined extracts were washed with saturated aqueous $NaHCO_3$ followed by brine. The solution was dried over Na_2SO_4 and concentrated by evaporation. Purification of the residue by chromatography on silica gel (*n*-

[1] E. Tayama, K. Watanabe and Y. Matano, *Eur. J. Org. Chem.*, 2016, 3631.

hexane/EtOAc = 10/1 to 6/1 as the eluent) gave *tert*-butyl 1-methyl-2-(*o*-tolyl)azetidine-2-carboxylate (**1a**)[1] (236 mg, 67% yield) as a colourless oil. (Step 5) A mixture of **1a** (678 mg, 2.59 mmol) and $NaHCO_3$ (0.66 g, 7.9 mmol) in CH_2Cl_2 (13 mL) was treated with methyl trifluoromethanesulfonate (0.59 mL, 5.2 mmol) at 0 °C and stirred for 3 h at room temperature. The mixture was concentrated by evaporation to ca. 1/2 volume and purified by chromatography on silica gel (CH_2Cl_2/MeOH = 15/1 to 7/1 as the eluent) to obtain **2a** (1.11 g, quant.) as colourless crystals, mp 119-121 °C. IR (ATR) ν_{max}/cm^{-1} 3076, 3041, 2979, 2940, 1728, 1459, 1395, 1371, 1302, 1254, 1225, 1145, 1105, 1078, 1030, 995, 969, 946, 856, 832, 770, 753, 728; ^1H NMR (400 MHz, $CDCl_3$) δ 7.45-7.33 (3H, m, ArH), 7.29-7.23 (1H, m, ArH), 4.52 (1H, ddd, *J* = 10.6, 10.4, 9.4 Hz, 4-H), 4.27 (1H, ddd, *J* = 9.6, 9.4, 2.4 Hz, 4-H), 3.90 (1H, ddd, *J* = 12.4, 10.6, 9.6 Hz, 3-H), 3.63 (3H, s, NCH_3), 3.02-2.84 (1H, br m, 3-H), 2.97 (3H, s, NCH_3), 2.33 (3H, s, $ArCH_3$), 1.39 (9H, s, *t*Bu); $^{13}C\{^1H\}$ NMR (101 MHz, $CDCl_3$) δ 166.4, 135.9, 132.5, 130.6, 129.7, 128.5, 126.9, 120.6 (q, *J* = 322 Hz), 86.8, 86.3, 62.7, 50.9, 50.7, 27.4, 27.1, 20.7; HRMS (ESI): calcd. for $C_{17}H_{26}NO_2$ $[M - OTf]^+$ 276.1958, found 276.1955.

2-2. 2-(5-Bromo-2-methylphenyl)-2-(*tert*-butoxycarbonyl)-1,1-dimethylazetidin-1-ium trifluoromethanesulfonate (**2b**)

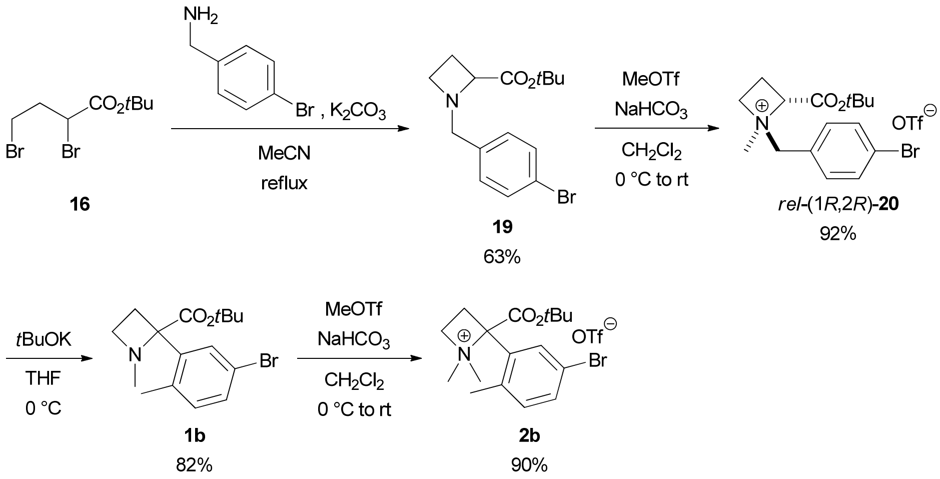

Prepared from **1b**[1] by the same procedures with the preparation of **2a** shown in the representative procedure (2-1). The yields were shown in the above scheme. Colourless gum; IR (ATR) ν_{max}/cm^{-1} 2980, 2938, 1731, 1461, 1397, 1372, 1250, 1224, 1141, 1095, 1075, 1029, 992, 942, 857, 831, 808, 791, 756, 724, 692; ^1H NMR (400 MHz, $CDCl_3$) δ 7.51 (1H, dd, *J* = 8.2, 2.0 Hz, ArH), 7.48 (1H, br s, ArH), 7.18 (1H, d, *J* = 8.2 Hz, ArH), 4.56 (1H, ddd, *J* = 10.6, 10.2, 9.6 Hz, 4-H), 4.31 (1H, ddd, *J* = 10.2, 9.3, 3.2 Hz, 4-H), 3.86 (1H, ddd, *J* = 12.9, 10.6, 9.3 Hz, 3-H), 3.64 (3H, s, NCH_3), 3.00 (3H, s, NCH_3), 2.95-2.80 (1H, br m, 3-H), 2.30 (3H, s, $ArCH_3$), 1.49 (9H, s, *t*Bu); $^{13}C\{^1H\}$ NMR (101 MHz, $CDCl_3$) δ 165.9, 135.4, 134.2, 133.7, 131.8, 131.2, 120.6 (q, *J* = 322 Hz), 120.3, 86.9, 85.8, 62.8, 51.1, 50.7, 27.4, 27.0, 20.4; HRMS (ESI): calcd. for $C_{17}H_{25}BrNO_2$ $[M - OTf]^+$ 354.1063; found 354.1058.

2-3. 2-(*tert*-Butoxycarbonyl)-1,1-dimethyl-2-(2-methyl-5-(trifluoromethyl)phenyl)azetidin-1-ium trifluoromethanesulfonate (**2c**)

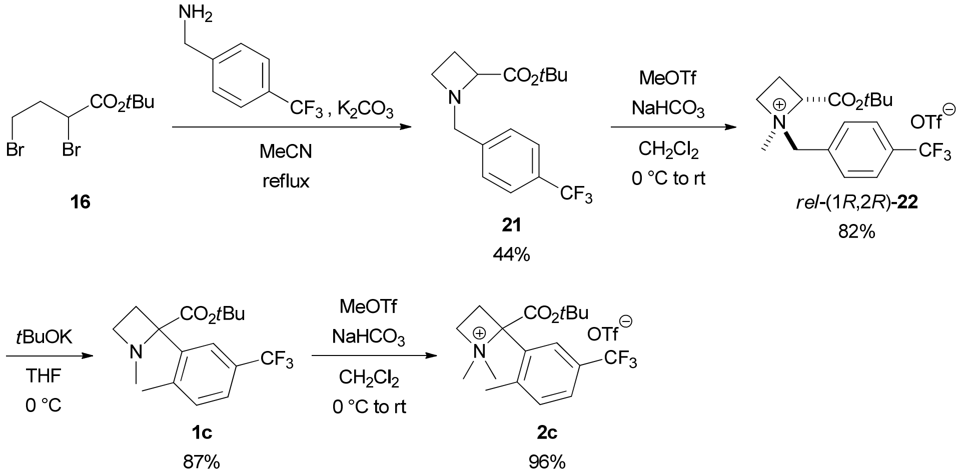

Prepared from **1c**[1] by the same procedures with the preparation of **2a** shown in the representative procedure (2-1). The yields were shown in the above scheme. Colourless gum; IR (ATR) ν_{max}/cm^{-1} 3041, 2982, 2939, 1733, 1624, 1462, 1415, 1398, 1373, 1334, 1253, 1225, 1123, 1086, 1029, 997, 974, 942, 896, 863, 836, 795, 768, 756, 747, 731, 703; ^{1}H NMR (400 MHz, $CDCl_3$) δ 7.65 (1H, d, J = 8.0 Hz, ArH), 7.59 (1H, br s, ArH), 7.47 (1H, d, J = 8.0 Hz, ArH), 4.60 (1H, ddd, J = 10.0, 10.0, 10.0 Hz, 4-H), 4.27 (1H, ddd, J = 10.0, 10.0, 2.6 Hz, 4-H), 3.97 (1H, ddd, J = 12.0, 10.0, 10.0 Hz, 3-H), 3.65 (3H, s, NCH_3), 2.98 (3H, s, NCH_3), 2.95 (1H, br, 3-H), 2.43 (3H, s, $ArCH_3$), 1.40 (9H, s, *t*Bu); ^{13}C{^{1}H} NMR (101 MHz, $CDCl_3$) δ 165.6, 140.9 (q, J = 1 Hz), 133.3, 130.9, 129.0 (q, J = 33 Hz), 127.1 (q, J = 4 Hz), 125.2, 123.4 (q, J = 273 Hz), 120.5 (q, J = 322 Hz), 87.0, 85.9, 62.8, 51.0, 50.7, 27.2, 26.8, 20.7; HRMS (ESI): calcd. for $C_{18}H_{25}F_3NO_2$ [M – OTf]$^+$ 344.1832; found 344.1819.

2-4. 2-(*tert*-Butoxycarbonyl)-2-(2,5-dimethylphenyl)-1,1-dimethylazetidin-1-ium trifluoromethanesulfonate (**2d**)

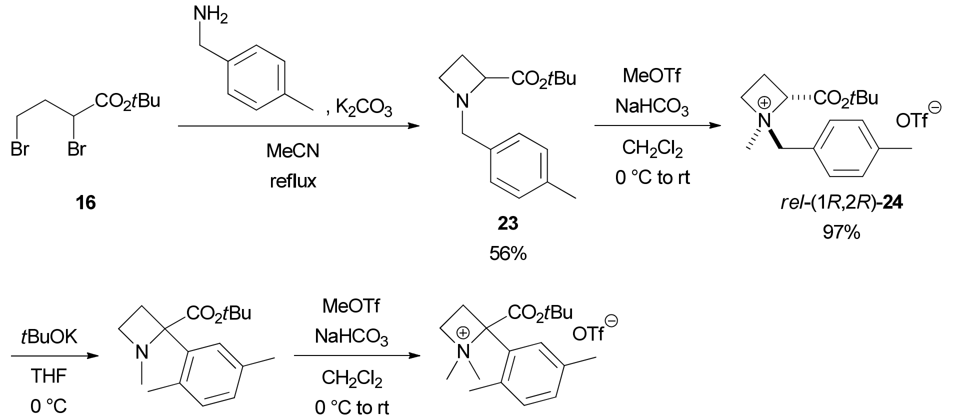

Prepared from **1d**[1] by the same procedures with the preparation of **2a** shown in the representative procedure (2-1). The yields were shown in the above scheme. Colourless crystals, mp 105-107 °C; IR (ATR) ν_{max}/ cm^{-1} 2976, 2936, 1731, 1505, 1477, 1458, 1396, 1371, 1296, 1254, 1224, 1146, 1105, 1078, 1029, 982, 969, 958, 872, 847, 827, 794, 765, 755, 739, 696; ^{1}H NMR (400 MHz, $CDCl_3$) δ 7.21 (1H, br s, ArH), 7.19 (1H, d,

J = 8.0 Hz, ArH), 7.14 (1H, d, J = 8.0 Hz, ArH), 4.52 (1H, ddd, J = 10.0, 10.0, 9.4 Hz, 4-H), 4.28 (1H, ddd, J = 10.2, 9.4, 3.0 Hz, 4-H), 3.90 (1H, ddd, J = 12.0, 10.2, 10.0 Hz, 3-H), 3.63 (3H, s, NCH_3), 3.02-2.80 (1H, br m, 3-H), 2.95 (3H, s, NCH_3), 2.39 (3H, s, $ArCH_3$), 2.28 (3H, s, $ArCH_3$), 1.40 (9H, s, *t*Bu); $^{13}C\{^1H\}$ NMR (101 MHz, $CDCl_3$) δ 166.6, 136.7, 132.7, 132.4, 131.4, 129.3, 129.1, 120.7 (q, J = 322 Hz), 86.8, 86.2, 62.5, 50.9, 50.6, 27.4, 27.0, 20.8, 20.2; HRMS (ESI): calcd. for $C_{18}H_{28}NO_2$ [M – OTf]$^+$ 290.2115; found 290.2110.

2-5. 2-(*tert*-Butoxycarbonyl)-2-(5-methoxy-2-methylphenyl)-1,1-dimethylazetidin-1-ium trifluoromethanesulfonate (**2e**)

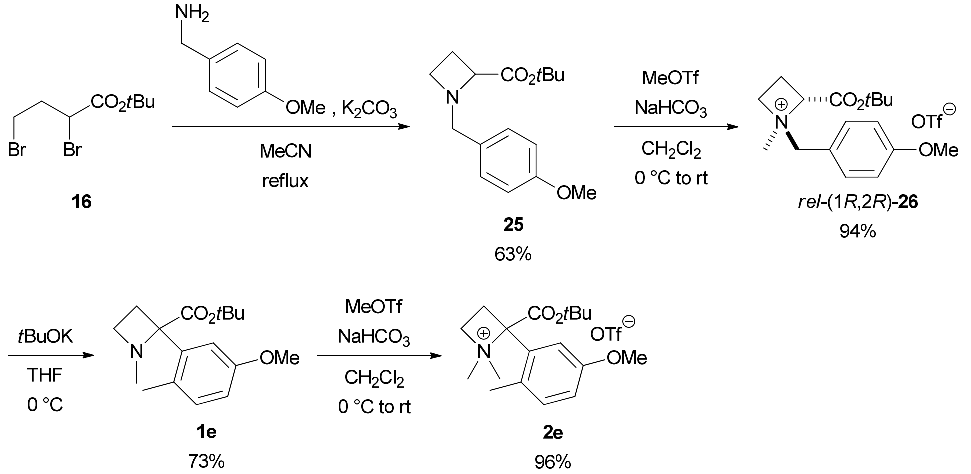

Prepared from **1e**[1] by the same procedures with the preparation of **2a** shown in the representative procedure (2-1). The yields were shown in the above scheme. Colourless crystals, mp 81-83 °C; IR (ATR) ν_{max}/cm^{-1} 3033, 2978, 2939, 1731, 1614, 1508, 1476, 1456, 1444, 1412, 1396, 1372, 1317, 1296, 1257, 1223, 1145, 1101, 1075, 1028, 971, 943, 844, 836, 820, 794, 769, 755, 739, 696; ^1H NMR (400 MHz, $CDCl_3$) δ 7.18 (1H, d, J = 8.4 Hz, ArH), 6.96-6.88 (2H, m, ArH), 4.52 (1H, ddd, J = 10.2, 10.2, 9.4 Hz, 4-H), 4.26 (1H, ddd, J = 10.2, 9.4, 3.2 Hz, 4-H), 3.91 (1H, ddd, J = 12.0, 10.2, 10.2 Hz, 3-H), 3.85 (3H, s, OCH_3), 3.63 (3H, s, NCH_3), 2.96 (3H, s, NCH_3), 2.91-2.76 (1H, br m, 3-H), 2.25 (3H, s, $ArCH_3$), 1.41 (9H, s, *t*Bu); $^{13}C\{^1H\}$ NMR (101 MHz, $CDCl_3$) δ 166.4, 158.2, 133.6, 130.5, 127.5, 120.6 (q, J = 322 Hz), 116.0, 114.4, 86.4, 86.3, 62.4, 55.5, 50.9, 50.6, 27.4, 27.0, 19.8; HRMS (ESI): calcd. for $C_{18}H_{28}NO_3$ [M – OTf]$^+$ 306.2064; found 306.2060.

2-6. 2-(4-Bromo-2-methylphenyl)-2-(*tert*-butoxycarbonyl)-1,1-dimethylazetidin-1-ium trifluoromethanesulfonate (**2f**)

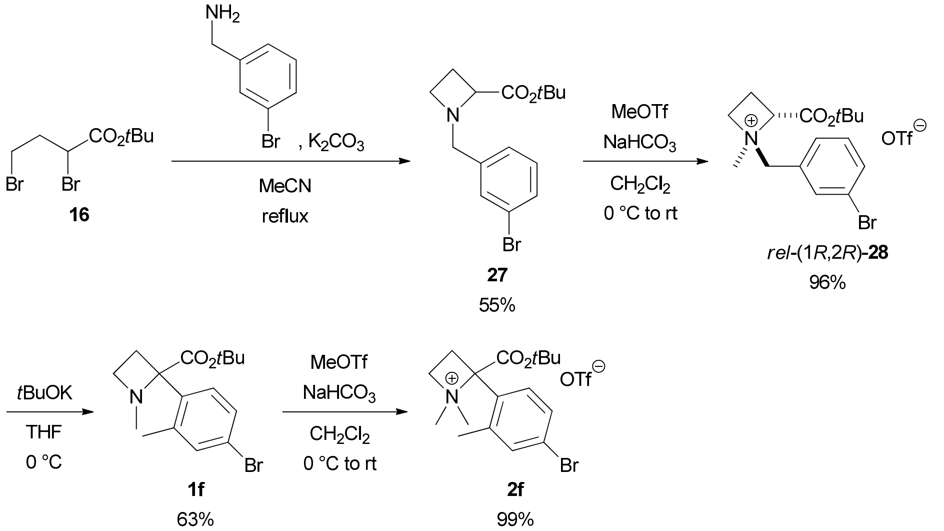

Prepared from *tert*-butyl 2-(4-bromo-2-methylphenyl)-1-methylazetidine-2-carboxylate (**1f**) by the same procedures with the preparation of **2a** shown in the representative procedure (2-1). The yields were shown in the above scheme. **1f**: colourless crystals, mp 133-135 °C; IR (ATR) ν_{max}/cm^{-1} 2972, 2931, 2854, 2829, 2780, 1712, 1591, 1564, 1474, 1442, 1392, 1367, 1254, 1237, 1207, 1159, 1123, 1085, 1053, 973, 955, 942, 910, 869, 840, 810, 768, 749, 724; ^1H NMR (400 MHz, $CDCl_3$) δ 7.39 (1H, d, J = 8.0 Hz, ArH), 7.32 (1H, dd, J = 8.0, 1.8 Hz, ArH), 7.22 (1H, d, J = 1.8 Hz, ArH), 3.47 (1H, ddd, J = 8.6, 6.1, 2.5 Hz, 4-H), 3.32 (1H, ddd, J = 8.6, 8.2, 6.1 Hz, 4-H), 2.94 (1H, ddd, J = 10.5, 8.2, 2.5 Hz, 3-H), 2.46 (3H, s, NCH_3), 2.10 (1H, ddd, J = 10.5, 8.6, 8.6 Hz, 3-H), 2.05 (3H, s, $ArCH_3$), 1.43 (9H, s, *t*Bu); $^{13}C\{^1H\}$ NMR (101 MHz, $CDCl_3$) δ 170.1, 141.8, 135.7, 132.8, 128.5, 127.0, 120.2, 81.9, 75.3, 52.0, 39.7, 28.9, 28.1, 18.8; HRMS (ESI): calcd. for $C_{16}H_{23}BrNO_2$ $[M + H]^+$ 340.0907, found 340.0902. **2f**: colourless gum; IR (ATR) ν_{max}/cm^{-1} 3034, 2980, 2937, 1731, 1590, 1561, 1466, 1397, 1372, 1250, 1224, 1142, 1098, 1079, 1028, 986, 941, 910, 857, 846, 825, 791, 756; ^1H NMR (400 MHz, $CDCl_3$) δ 7.51 (1H, dd, J = 8.6, 1.8 Hz, ArH), 7.42 (1H, d, J = 1.8 Hz, ArH), 7.35 (1H, d, J = 8.6 Hz, ArH), 4.50 (1H, ddd, J = 10.0, 10.0, 9.6 Hz, 4-H), 4.24 (1H, ddd, J = 9.6, 9.6, 2.8 Hz, 4-H), 3.99-3.84 (1H, br m, 3-H), 3.60 (3H, s, NCH_3), 2.98 (3H, s, NCH_3), 2.94-2.78 (1H, br m, 3-H), 2.31 (3H, s, $ArCH_3$), 1.41 (9H, s, *t*Bu); $^{13}C\{^1H\}$ NMR (101 MHz, $CDCl_3$) δ 165.7, 138.1, 134.9, 130.3, 129.8, 128.9, 124.7, 120.4 (q, J = 322 Hz), 86.6, 86.2, 62.6, 50.8, 50.5, 27.2, 26.8, 20.2; HRMS (ESI): calcd. for $C_{17}H_{25}BrNO_2$ $[M - OTf]^+$ 354.1063; found 354.1058.

2-7. 2-(*tert*-Butoxycarbonyl)-1,1-dimethyl-2-(2-methyl-4-(trifluoromethyl)phenyl)azetidin-1-ium trifluoromethanesulfonate (**2g**)

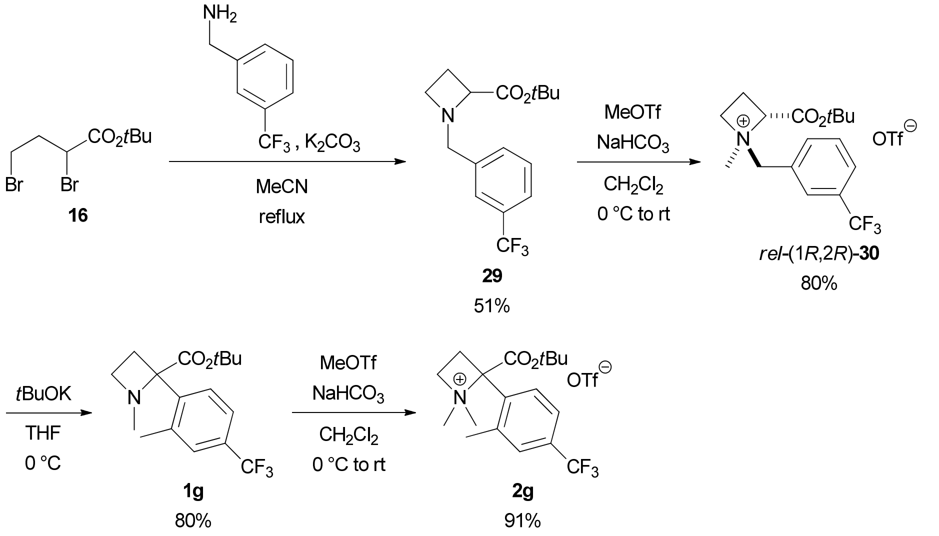

Prepared from *tert*-butyl 1-methyl-2-(2-methyl-4-(trifluoromethyl)phenyl)azetidine-2-carboxylate (**1g**) by the same procedures with the preparation of **2a** shown in the representative procedure (2-1). The yields were shown in the above scheme. **1g**: colourless crystals, mp 79-81 °C; IR (ATR) ν_{max}/cm^{-1} 3014, 2979, 2957, 2930, 2860, 2843, 2791, 1716, 1616, 1474, 1447, 1411, 1395, 1369, 1326, 1254, 1208, 1183, 1156, 1118, 1083, 998, 972, 956, 912, 894, 877, 844, 813, 771, 757, 739; ^{1}H NMR (400 MHz, CDCl$_3$) δ 7.66 (1H, d, J = 8.4 Hz, ArH), 7.46 (1H, d, J = 8.4 Hz, ArH), 7.32 (1H, s, ArH), 3.48 (1H, ddd, J = 8.6, 6.0, 2.6 Hz, 4-H), 3.36 (1H, ddd, J = 8.8, 8.2, 6.0 Hz, 4-H), 2.98 (1H, ddd, J = 10.5, 8.2, 2.6 Hz, 3-H), 2.49 (3H, s, NCH$_3$), 2.13 (3H, s, ArCH$_3$), 2.12 (1H, ddd, J = 10.5, 8.8, 8.6 Hz, 3-H), 1.43 (9H, s, *t*Bu); ^{13}C{^{1}H} NMR (101 MHz, CDCl$_3$) δ 169.9, 146.5, 134.2, 128.7 (q, J = 32 Hz), 126.8 (q, J = 4 Hz), 125.7, 124.4 (q, J = 273 Hz), 122.4 (q, J = 4 Hz), 82.1, 75.5, 52.0, 39.6, 28.8, 28.0, 19.0; HRMS (ESI): calcd. for $C_{17}H_{23}F_3NO_2$ [M + H]$^+$ 330.1675, found 330.1666. **2g**: colourless gum; IR (ATR) ν_{max}/cm^{-1} 3048, 2982, 2939, 1733, 1469, 1410, 1373, 1335, 1252, 1225, 1123, 1092, 1029, 941, 914, 880, 829, 795, 768, 756, 741, 715; ^{1}H NMR (400 MHz, CDCl$_3$) δ 7.68-7.60 (2H, m, ArH), 7.52 (1H, s, ArH), 4.55 (1H, ddd, J = 10.0, 10.0, 9.5 Hz, 4-H), 4.23 (1H, ddd, J = 10.0, 9.5, 2.6 Hz, 4-H), 4.04-3.88 (1H, br m, 3-H), 3.62 (3H, s, NCH$_3$), 3.10-2.90 (1H, br m, 3-H), 3.02 (3H, s, NCH$_3$), 2.40 (3H, s, ArCH$_3$), 1.41 (9H, s, *t*Bu); ^{13}C{^{1}H} NMR (101 MHz, CDCl$_3$) δ 165.5, 137.2, 133.7, 132.1 (q, J = 33 Hz), 129.5, 128.9 (q, J = 4 Hz), 123.5 (q, J = 4 Hz), 123.4 (q, J = 271 Hz), 120.5 (q, J = 318 Hz), 87.0, 86.2, 63.1, 51.1, 50.8, 27.2, 27.1, 20.5; HRMS (ESI): calcd. for $C_{18}H_{25}F_3NO_2$ [M – OTf]$^+$ 344.1832; found 344.1817.

2-8. 2-(3-Bromo-2-methylphenyl)-2-(*tert*-butoxycarbonyl)-1,1-dimethylazetidin-1-ium trifluoromethanesulfonate (**2h**)

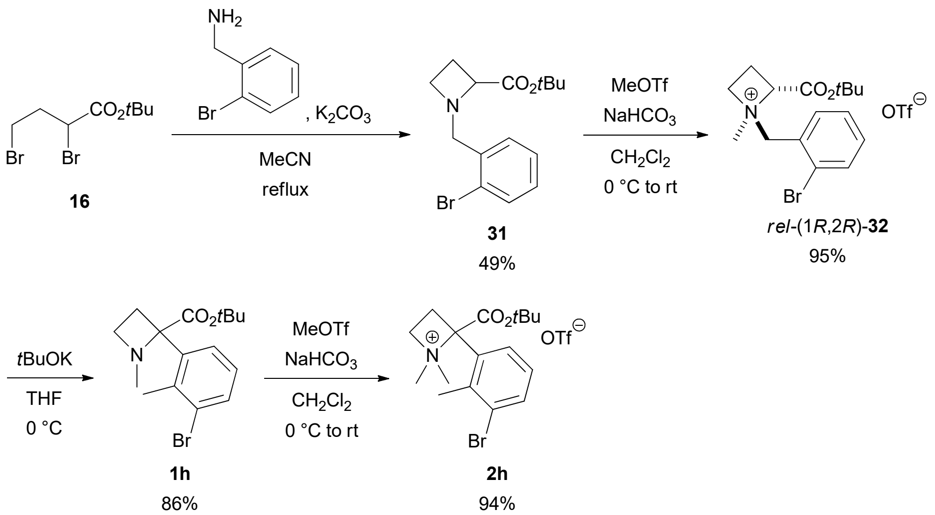

Prepared from *tert*-butyl 2-(3-bromo-2-methylphenyl)-1-methylazetidine-2-carboxylate (**1h**) by the same procedures with the preparation of **2a** shown in the representative procedure (2-1). The yields were shown in the above scheme. **1h**: yellow crystals, mp 42-44 °C; IR (ATR) ν_{max}/cm^{-1} 3002, 2971, 2933, 2860, 2837, 2788, 1713, 1593, 1560, 1458, 1433, 1393, 1366, 1279, 1253, 1210, 1195, 1159, 1149, 1126, 1088, 1055, 995, 971, 944, 909, 845, 822, 789, 771, 747, 717, 696; ^{1}H NMR (400 MHz, $CDCl_3$) δ 7.50 (1H, dd, J = 8.0, 1.2 Hz, ArH), 7.43 (1H, dd, J = 8.0, 1.2 Hz, ArH), 7.06 (1H, dd, J = 8.0, 8.0 Hz, ArH), 3.46 (1H, ddd, J = 8.5, 6.0, 2.6 Hz, 4-H), 3.33 (1H, ddd, J = 8.8, 8.2, 6.0 Hz, 4-H), 2.96 (1H, ddd, J = 10.7, 8.2, 2.6 Hz, 3-H), 2.47 (3H, s, NCH_3), 2.14 (1H, ddd, J = 10.7, 8.8, 8.5 Hz, 3-H), 2.12 (3H, s, $ArCH_3$), 1.43 (9H, s, *t*Bu); $^{13}C\{^1H\}$ NMR (101 MHz, $CDCl_3$) δ 170.2, 144.8, 133.0, 130.8, 126.7, 125.8, 124.6, 81.9, 76.0, 51.7, 39.6, 29.3, 28.1, 19.1; HRMS (ESI): calcd. for $C_{16}H_{23}BrNO_2$ $[M + H]^+$ 340.0907, found 340.0902. **2h**: colourless gum; IR (ATR) ν_{max}/cm^{-1} 2980, 2936, 1731, 1635, 1562, 1467, 1436, 1397, 1372, 1251, 1224, 1141, 1073, 1029, 1003, 984, 942, 854, 830, 781, 757, 735, 701; ^{1}H NMR (400 MHz, $CDCl_3$) δ 7.70 (1H, d, J = 7.8 Hz, ArH), 7.51 (1H, d, J = 7.8 Hz, ArH), 7.28 (1H, dd, J = 7.8, 7.8 Hz, ArH), 4.57 (1H, ddd, J = 10.0, 9.7, 9.7 Hz, 4-H), 4.24 (1H, ddd, J = 9.7, 9.7, 2.8 Hz, 4-H), 3.96 (1H, ddd, J = 10.4, 10.0, 9.7 Hz, 3-H), 3.64 (3H, s, NCH_3), 3.03-2.77 (1H, br, 3-H), 2.94 (3H, s, NCH_3), 2.34 (3H, s, $ArCH_3$), 1.41 (9H, s, *t*Bu); $^{13}C\{^1H\}$ NMR (101 MHz, $CDCl_3$) δ 166.3, 135.5, 135.0, 131.7, 128.6, 128.1, 127.6, 120.6 (q, J = 322 Hz), 86.8, 86.5, 62.4, 51.1, 50.2, 27.5, 27.3, 21.5; HRMS (ESI): calcd. for $C_{17}H_{25}BrNO_2$ $[M - OTf]^+$ 354.1063; found 354.1059.

2-9. (*S*)-*tert*-Butyl 1-methyl-2-(*o*-tolyl)azetidine-2-carboxylate [(*S*)-**1a**][1]

(*S*)-**1a** was prepared according to the slightly modified procedures reported by our group.[1]

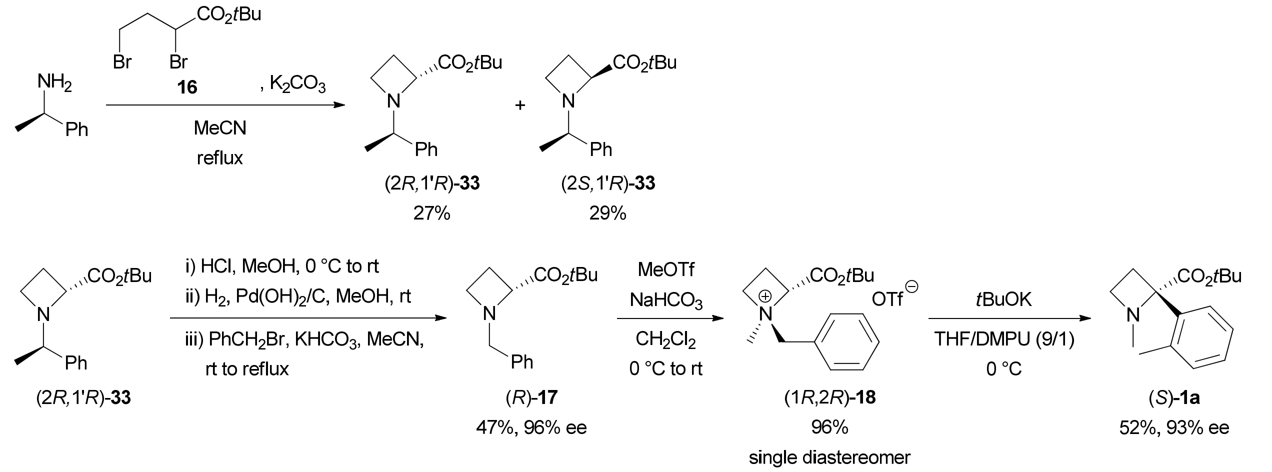

(Step 1) A mixture of **16** (3.02 g, 10.0 mmol), (*R*)-1-phenylethylamine (1.27 mL, 10.0 mmol), and K_2CO_3 (4.15 g, 30.0 mmol) in MeCN (50 mL) was refluxed for 7 h. The resulting mixture was cooled to room temperature followed by filtered. The filtrate was concentrated by evaporation and the residue was purified by chromatography on silica gel [*n*-hexane/EtOAc = 15/1 to 5/1 as the eluent, R_f: (2*R*,1′*R*) > (2*S*,1′*R*)] to obtain (2*R*,1′*R*)-*tert*-butyl 1-(1′-phenylethyl)azetidine-2-carboxylate (2*R*,1′*R*)-**33** (718 mg, 27% yield) as a pale yellow oil and (2*S*,1′*R*)-*tert*-butyl 1-(1′-phenylethyl)azetidine-2-carboxylate (2*S*,1′*R*)-**33** (768 mg, 29% yield) as a pale yellow oil. (2*R*,1′*R*)-**33**: $[\alpha]^{24}_{589}$ +108.6 (*c* 1.0 in EtOH); (2*S*,1′*R*)-**33**: $[\alpha]^{24}_{589}$ –58.7 (*c* 1.0 in EtOH). (Step 2) A solution of (2*R*,1′*R*)-**33** (718 mg, 2.75 mmol) in MeOH (14 mL) was treated with a 4 M HCl cyclopentyl methyl ether (CPME) solution (0.83 mL, 3.3 mmol) at 0 °C. After stirring for 30 min at room temperature, the solution was concentrated by evaporation. A mixture of the residue and $Pd(OH)_2/C$ (Pd 20%, wetted with ca. 50% H_2O) (0.20 g) in MeOH (14 mL) was stirred for 3 days at room temperature under a H_2 atmosphere. The resulting mixture was filtered through a pad of Celite and the filtrate was concentrated by evaporation. A mixture of the residue, benzyl bromide (327 μL, 2.75 mmol), and $KHCO_3$ (1.38 g, 13.8 mmol) in MeCN (14 mL) was stirred for 1 h at room temperature and refluxed for 0.5 h. The resulting mixture was cooled to room temperature and filtered. The filtrate was concentrated by evaporation and the residue was purified by chromatography on silica gel (CH_2Cl_2/EtOAc = 7/1 to 5/1 as the eluent) to obtain (*R*)-*tert*-butyl 1-benzylazetidine-2-carboxylate (*R*)-**17** (323 mg, 47% yield) as a colourless oil. 96% ee [determined by HPLC analysis: Daicel Chiralcel OJ-H column (25 cm), *n*-hexane/2-PrOH = 99/1 as the eluent, flow rate = 0.50 mL/min, t_R = 11.8 min for (*R*)-**17** (97.9%) and 14.4 min for (*S*)-**17** (2.1%)]. $[\alpha]^{24}_{589}$ +86.2 (*c* 1.0 in EtOH). (Step 3) A mixture of (*R*)-**17** (323 mg, 1.31 mmol, 96% ee) and $NaHCO_3$ (0.33 g, 3.9 mmol) in CH_2Cl_2 (6.6 mL) was treated with methyl trifluoromethanesulfonate (0.22 μL, 1.96 mmol) at 0 °C and stirred for 1 h at room temperature. The resulting mixture was concentrated by evaporation to ca. 1/2 volume and purified by chromatography on silica gel (CH_2Cl_2/MeOH = 15/1 to 6/1 as the eluent) to obtain (1*R*,2*R*)-1-benzyl-2-(*tert*-butoxycarbonyl)-1-methylazetidin-1-ium trifluoromethanesulfonate (1*R*,2*R*)-**18** (516 mg, 96% yield) as a colourless gum. $[\alpha]^{24}_{589}$ +28.6 (*c* 1.0 in EtOH). (Step 4) A solution of (1*R*,2*R*)-**18** (516 mg, 1.25 mmol) in

THF (10 mL) and DMPU[2] (1.3 mL) was treated with a 1 M *t*BuOK THF solution (1.5 mL, 1.5 mmol) at 0 °C under an Ar atmosphere. After stirring for 3 h at the same temperature, the resulting mixture was quenched with saturated aqueous NH_4Cl and extracted with *n*-hexane. The combined extracts were washed with saturated aqueous $NaHCO_3$ followed by brine. The solution was dried over Na_2SO_4 and concentrated by evaporation. Purification by chromatography on silica gel (*n*-hexane/EtOAc = 10/0 to 10/1 as the eluent) gave (*S*)-**1a** (171 mg, 52% yield) as a colourless oil. 93% ee [determined by HPLC analysis: Daicel Chiralcel OJ-H column (25 cm), *n*-hexane/EtOH = 99/1 as the eluent, flow rate: 0.50 mL/min, *t*R = 8.1 min for (*S*)-**1a** (96.7 %) and 18.6 min for (*R*)-**1a** (3.3 %)]. $[a]^{23}_{589}$ –165.2 (*c* 1.0 in EtOH).

2-10. 1-Benzyl-2-(*tert*-butoxycarbonyl)-2-ethyl-1-methylazetidin-1-ium trifluoromethanesulfonate (**12**)

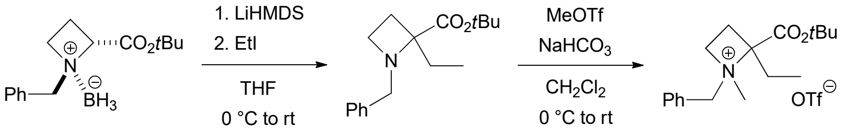

(Step 1) A solution of *rel*-((1*R*,2*R*)-1-benzyl-2-(*tert*-butoxycarbonyl)azetidin-1-ium-1-yl)trihydroborate[3] [*rel*-(1*R*,2*R*)-**34**] (346 mg, 1.32 mmol) in THF (7.5 mL) was treated with a 1.0 M LiHMDS solution in THF (3.2 mL, 3.2 mmol) at 0 °C under an Ar atmosphere. The solution was stirred for 30 min at 0 °C and treated with iodoethane (275 μL, 3.42 mmol) at the same temperature. The mixture was allowed to warm at room temperature and stirred for 3 h. The resulting mixture was quenched with saturated aqueous NH_4Cl and extracted with EtOAc. The combined organic extracts were washed with saturated aqueous $NaHCO_3$ followed by brine. The solution was dried over Na_2SO_4 and concentrated by evaporation. The residue was purified by chromatography on silica gel (*n*-hexane/EtOAc = 20/1, 10/1 to 5/1 as the eluent) to obtain *tert*-butyl 1-benzyl-2-ethylazetidine-2-carboxylate (**35**) (176 mg, 48% yield) as a coluorless oil. IR (ATR) ν_{max}/cm^{-1} 3087, 3062, 3028, 3004, 2967, 2931, 2877, 2828, 2799, 1716, 1602, 1495, 1476, 1455, 1391, 1366, 1338, 1312, 1248, 1227, 1175, 1140, 1028, 1004, 946, 908, 849, 828, 799, 732, 697; ^1H NMR (400 MHz, $CDCl_3$) δ 7.33-7.24 (4H, m, Ph), 7.24-7.17 (1H, m, Ph), 3.75 (1H, d, J = 12.8 Hz, CH_2Ph), 3.67 (1H, d, J = 12.8 Hz, CH_2Ph), 3.14 (1H, ddd, J = 8.3, 6.3, 5.9 Hz, 4-H), 3.07 (1H, ddd, J = 8.3, 6.3, 5.9 Hz, 4-H), 2.51 (1H, dddd, J = 10.7, 8.3, 5.9, 0.6 Hz, 3-H), 2.01-1.86 (2H, m, 3-H and CH_2CH$_3$), 1.83 (1H, dq, J = 13.2, 7.5 Hz, CH_2CH$_3$), 1.50 (9H, s, *t*Bu), 0.87 (3H, dd, J = 7.5, 7.5 Hz, CH$_2$CH_3); $^{13}C\{^1H\}$ NMR (101 MHz, $CDCl_3$) δ 172.7, 138.9, 128.5, 128.1, 126.7, 80.5, 72.9, 55.8, 49.4, 28.1, 26.5, 25.4, 8.0; HRMS (ESI): calcd. for $C_{17}H_{26}NO_2$ $[M + H]^+$ 276.1958, found 276.1952. (Step 2) A mixture of **35** (92 mg, 0.33 mmol) and $NaHCO_3$ (84 mg, 1.0 mmol) in CH_2Cl_2 (1.7 mL) was treated with methyl trifluoromethanesulfonate (74 μL, 0.65 mmol) at 0 °C and stirred for 2 h at room temperature. The mixture was concentrated by evaporation to ca. 1/2 volume and purified by chromatography on silica gel (CH_2Cl_2/MeOH = 20/1 to 10/1 as the eluent) to obtain **12** (146 mg, quant.) as a colourless gum. The product **12** was obtained as an almost single diastereomer. The relative stereochemistry of **12** was not determined. IR (ATR) ν_{max}/cm^{-1} 3040, 2981, 2942, 2887, 1733,

[2] Addition of DMPU [1,3-dimethyl-3,4,5,6-tetrahydro-2(1*H*)-pyrimidinone] improve the yield of the Sommelet–Hauser rearrangement product: E. Tayama, K. Hirano and S. Baba, *Tetrahedron*, 2020, **76**, 131064. See also ref. 1.

[3] E. Tayama, R. Nishio and Y. Kobayashi, *Org. Biomol. Chem.*, 2018, **16**, 5833.

1499, 1461, 1425, 1397, 1372, 1357, 1327, 1254, 1223, 1141, 1078, 1029, 958, 930, 877, 837, 802, 768, 754, 736, 706; ^{1}H NMR (400 MHz, $CDCl_3$) δ 7.56-7.36 (5H, m, Ph), 4.98 (1H, ddd, J = 10.4, 10.0, 9.7 Hz, 4-H), 4.83 (1H, d, J = 12.6 Hz, CH_2Ph), 4.08 (1H, d, J = 12.6 Hz, CH_2Ph), 3.51 (1H, ddd, J = 9.9, 9.7, 2.4 Hz, 4-H), 3.16 (1H, ddd, J = 11.2, 10.4, 9.9 Hz, 3-H), 3.02-2.82 (1H, m, 3-H), 2.95 (3H, s, NCH_3), 2.45-2.28 (2H, m, CH_2CH_3), 1.53 (9H, s, tBu), 0.97 (3H, t, J = 7.2 Hz, CH_2CH_3); ^{13}C{^{1}H} NMR (101 MHz, $CDCl_3$) δ 165.7, 131.8, 130.5, 129.4, 126.4, 120.6 (q, J = 322 Hz), 86.2, 85.4, 61.3, 58.9, 46.3, 27.6, 25.5, 22.7, 7.5; HRMS (ESI): calcd. for $C_{18}H_{28}NO_2$ $[M – OTf]^+$ 290.2115, found 290.2108.

3. Copies of ^{1}H, ^{13}C, and ^{19}F NMR (representative) spectra of substrates and products

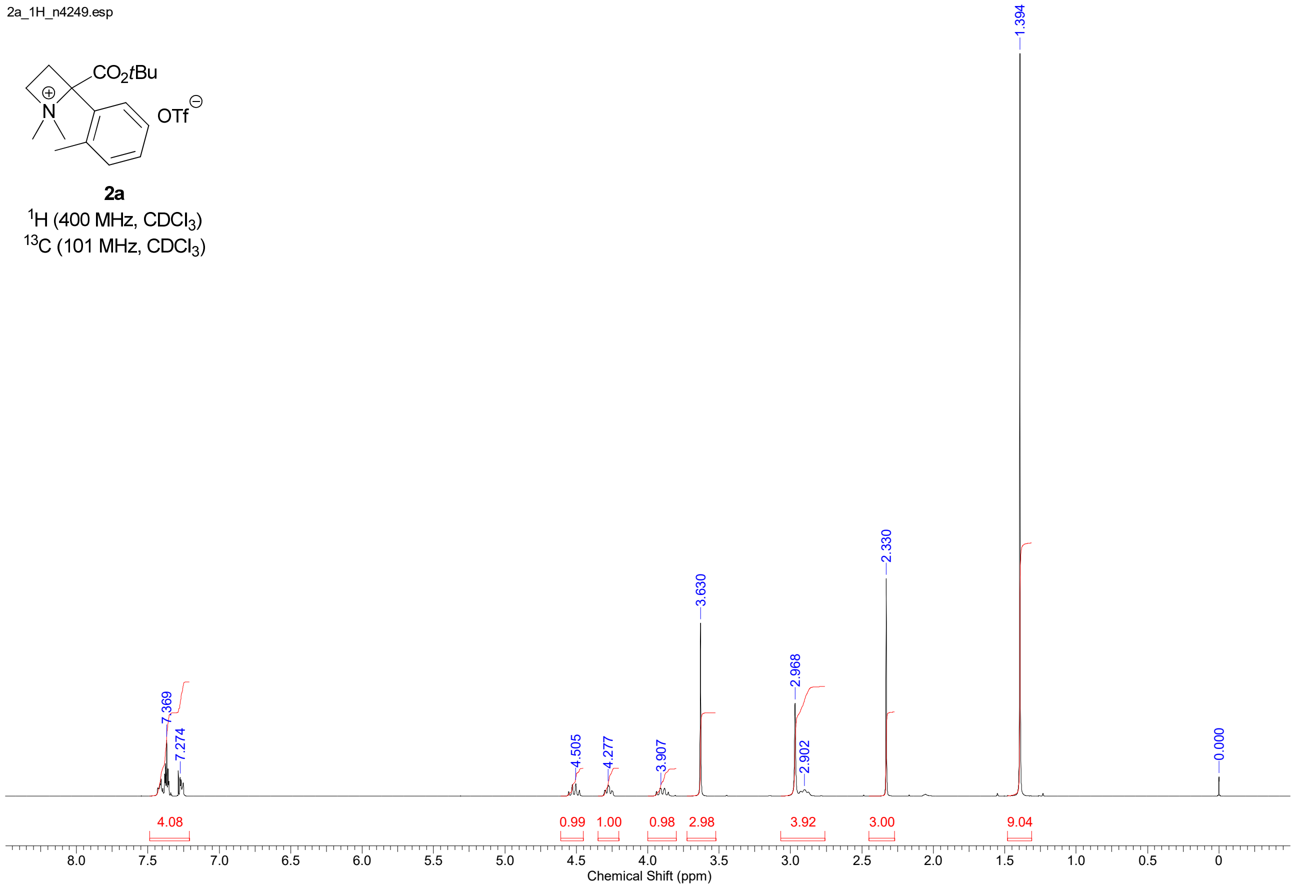

2a

^{1}H (400 MHz, $CDCl_3$)

^{13}C (101 MHz, $CDCl_3$)

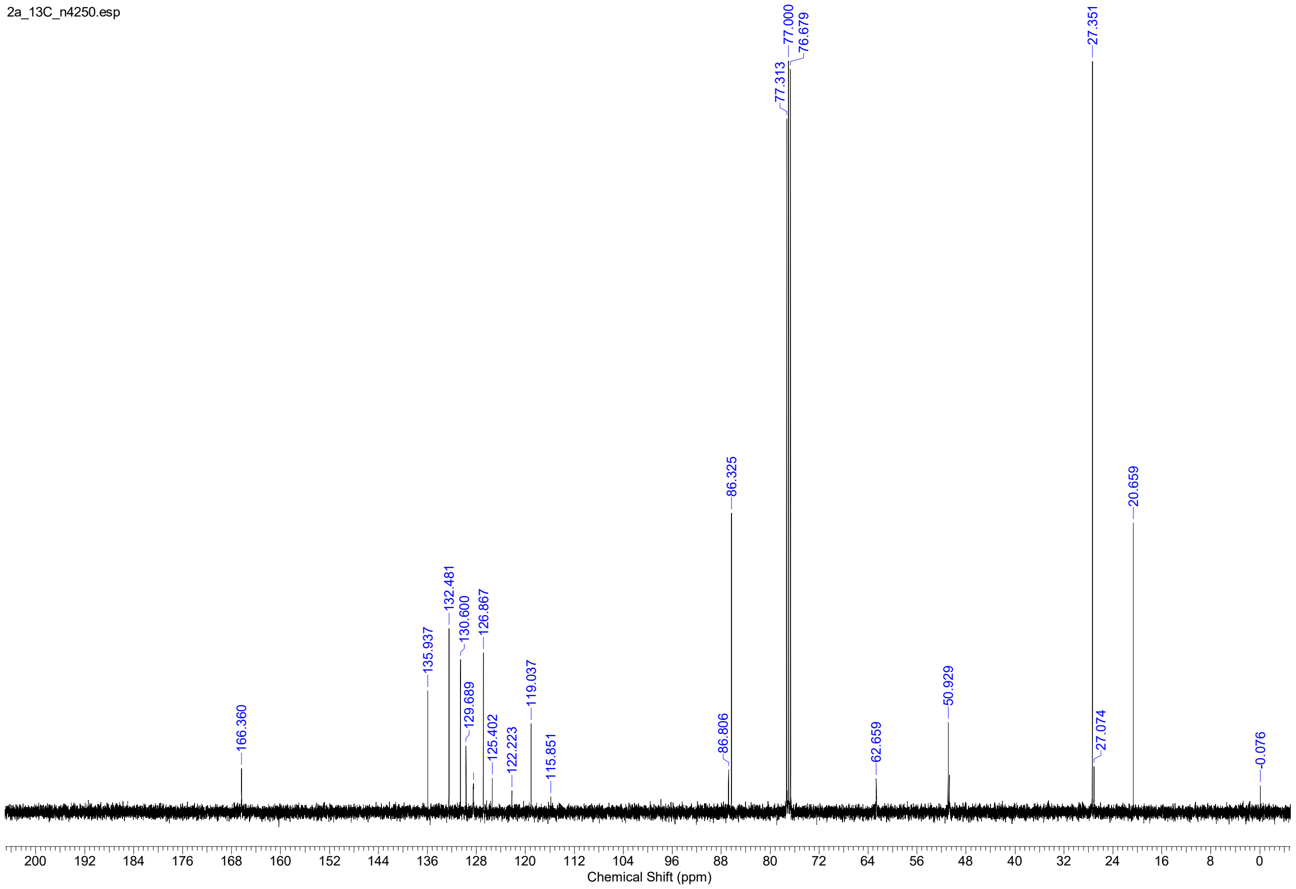

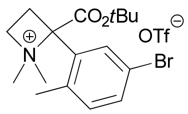

2b

^{1}H (400 MHz, $CDCl_3$)

^{13}C (101 MHz, $CDCl_3$)

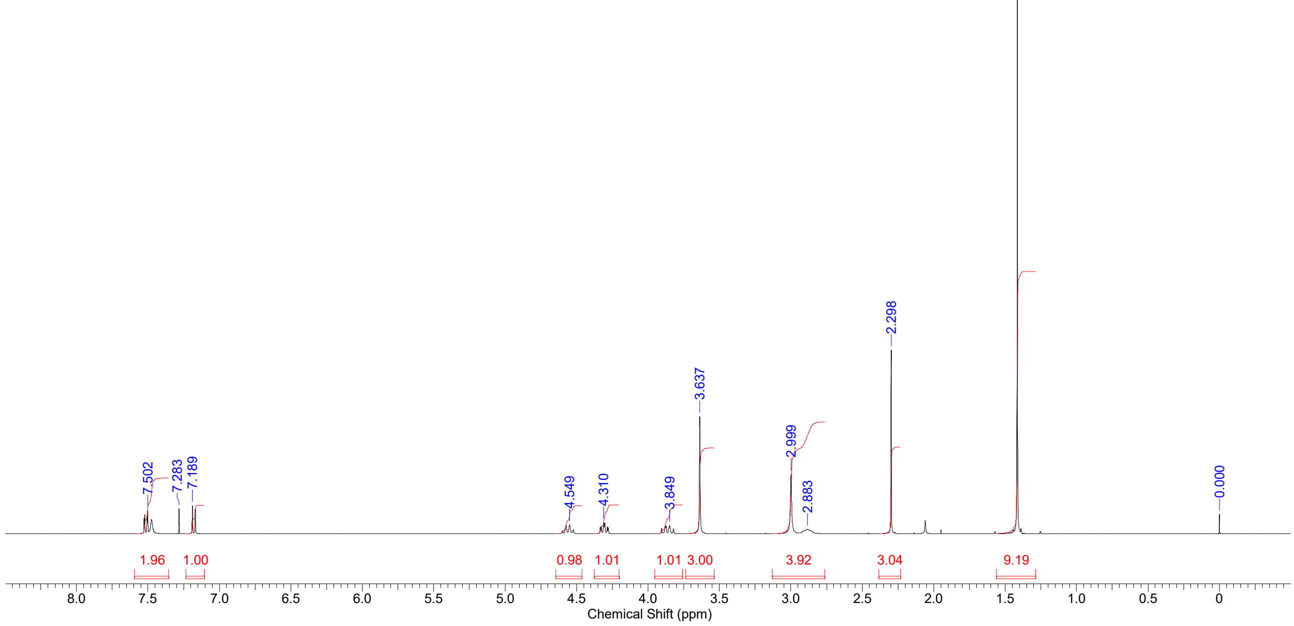

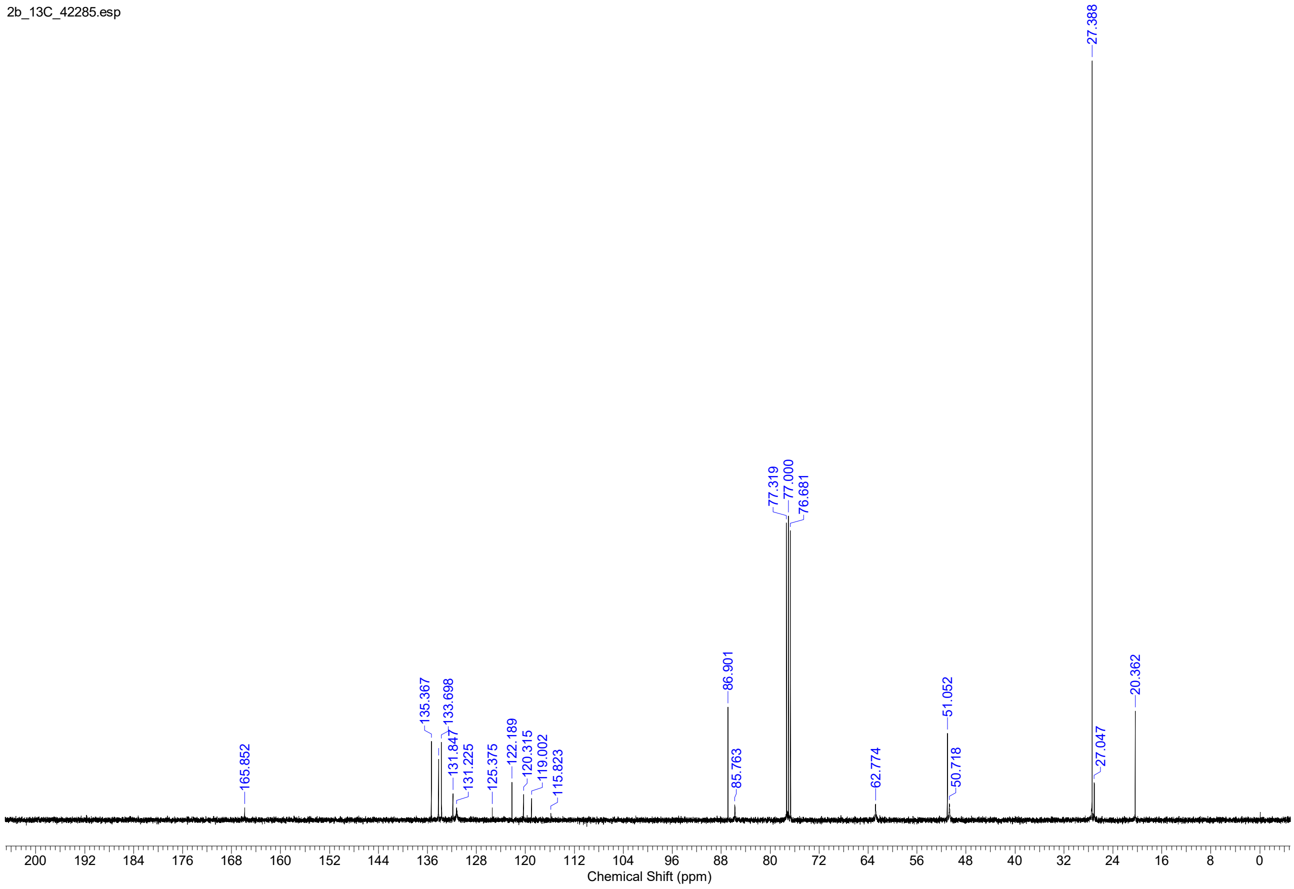

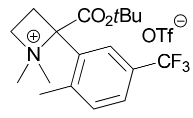

2c
^{1}H (400 MHz, $CDCl_3$)
^{13}C (101 MHz, $CDCl_3$)

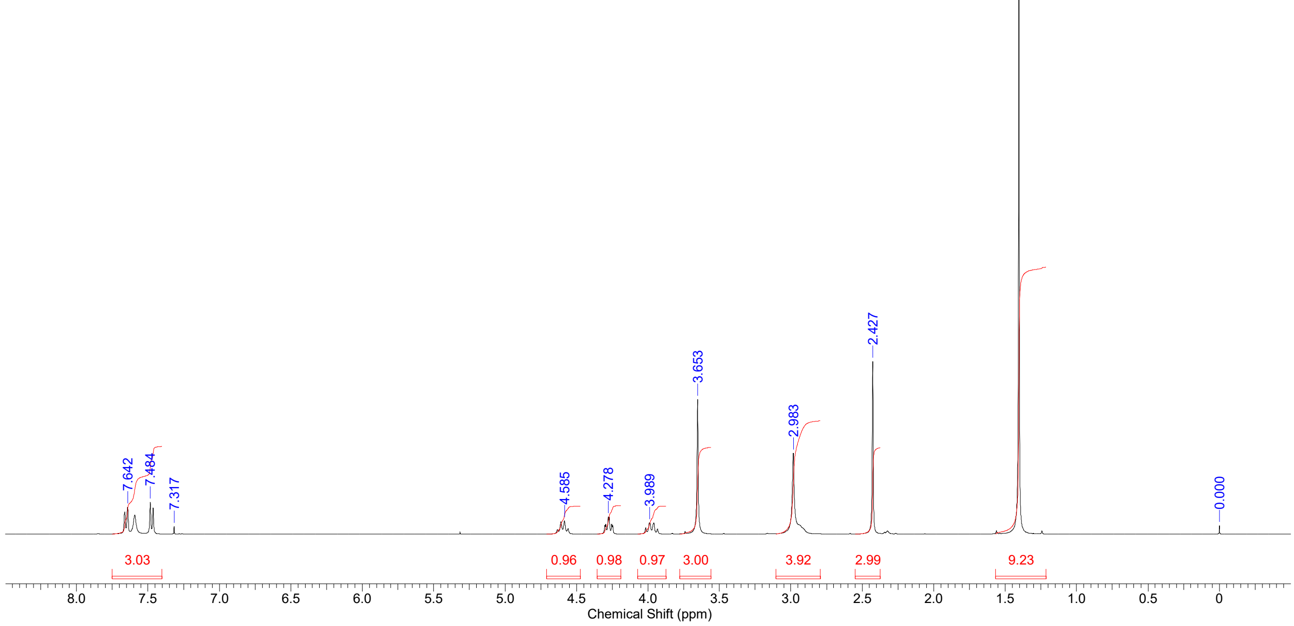

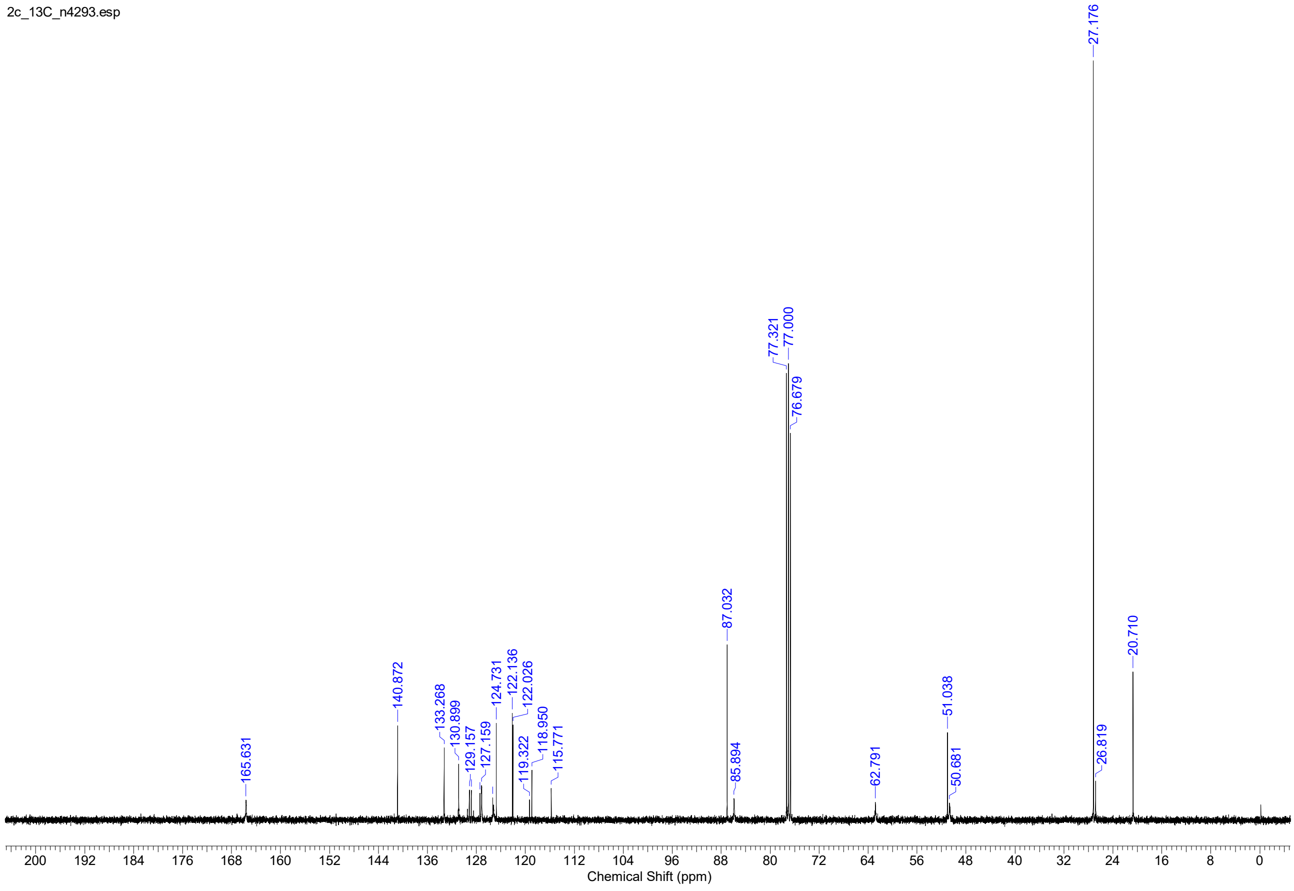

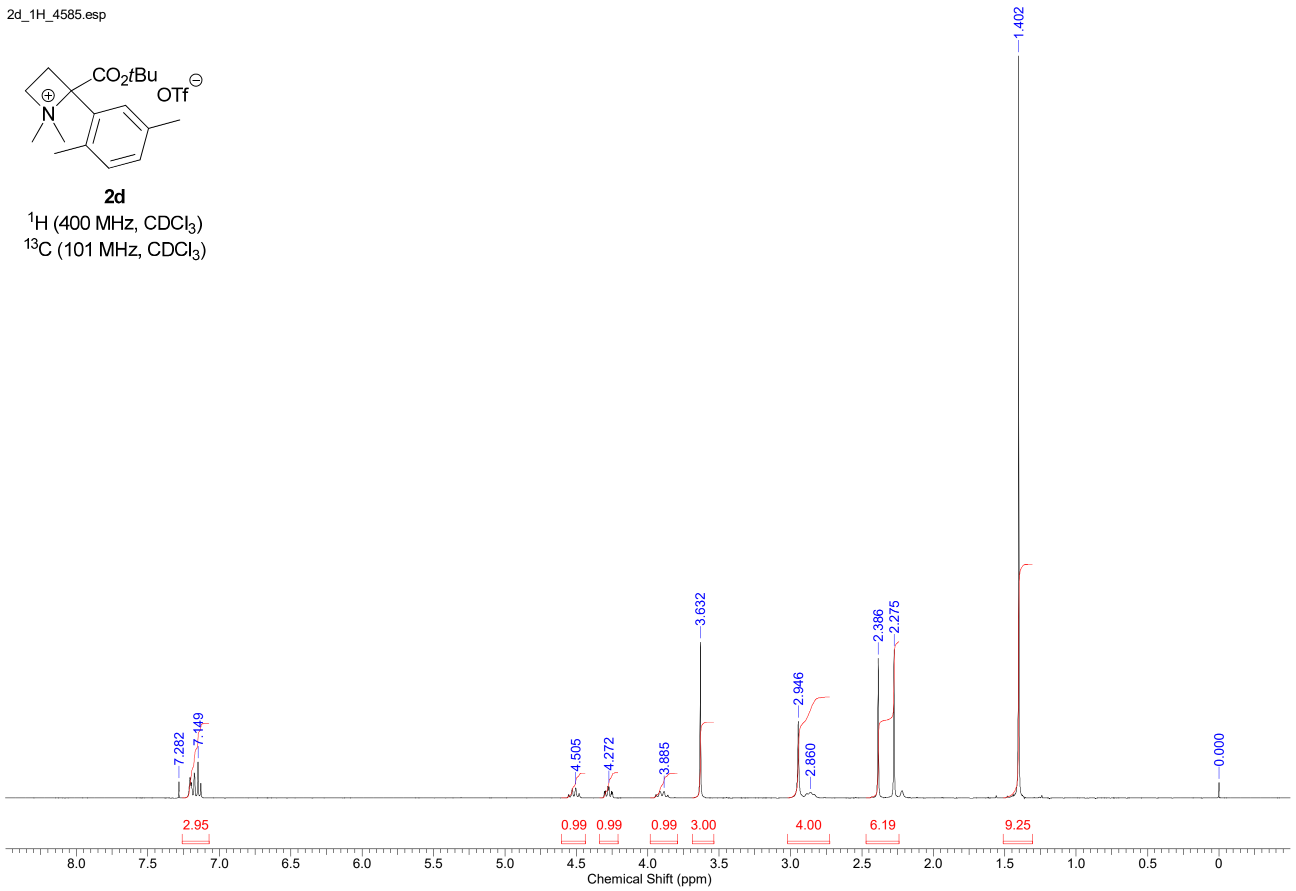

2d_1H_4585.esp
CO2tBu
OTf
N
2d
1H (400 MHz, CDCl3)
13C (101 MHz, CDCl3)
7.282
7.149
4.505
4.272
3.885
3.632
2.946
2.860
2.386
2.275
1.402
0.000
2.95
0.99
0.99
0.99
3.00
4.00
6.19
9.25
8.0 7.5 7.0 6.5 6.0 5.5 5.0 4.5 4.0 3.5 3.0 2.5 2.0 1.5 1.0 0.5 0
Chemical Shift (ppm)

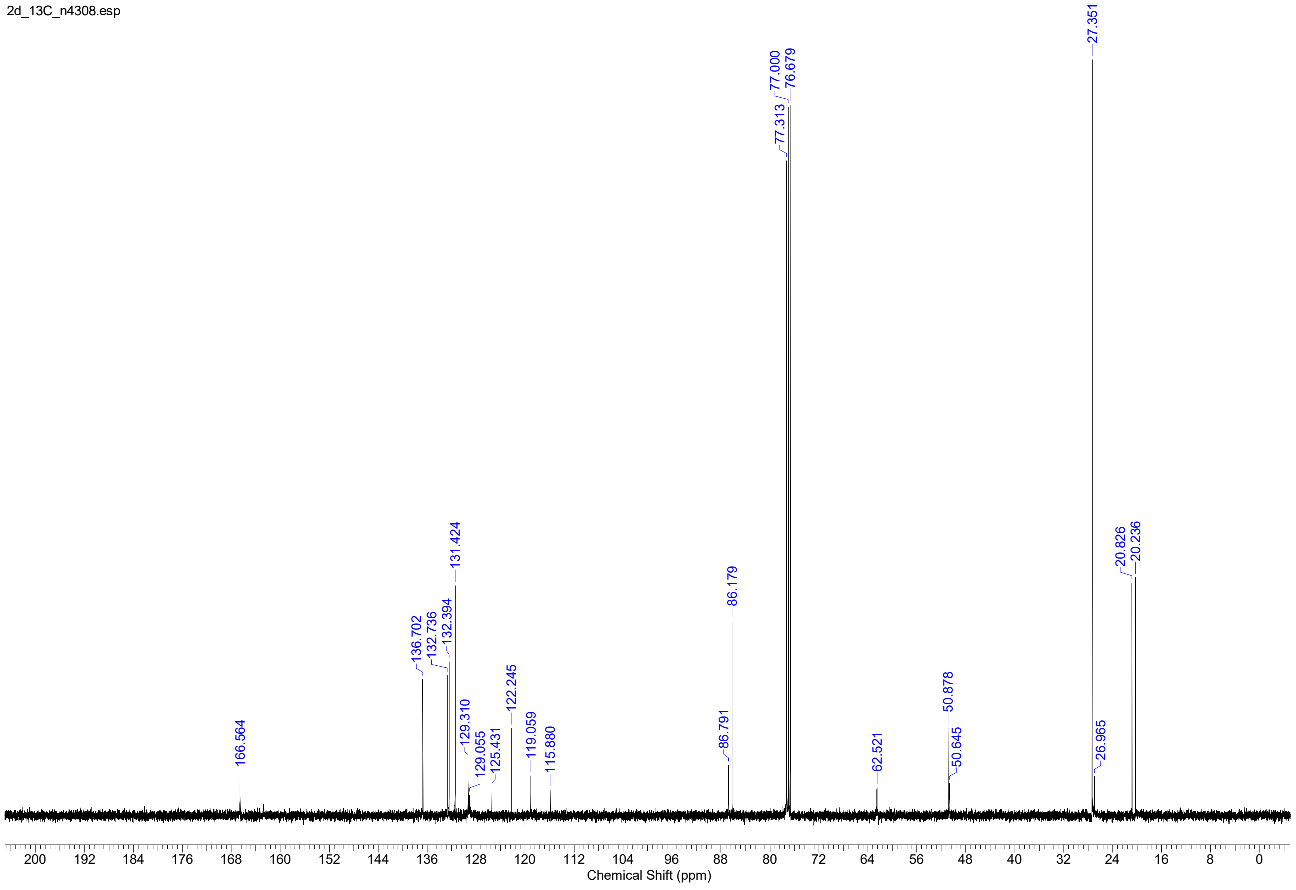

2d_13C_n4308.esp
166.564
136.702
132.736
132.394
131.424
129.310
129.055
125.431
122.245
119.059
115.880
86.791
86.179
77.313
77.000
76.679
62.521
50.878
50.645
27.351
26.965
20.826
20.236
200 192 184 176 168 160 152 144 136 128 120 112 104 96 88 80 72 64 56 48 40 32 24 16 8 0
Chemical Shift (ppm)

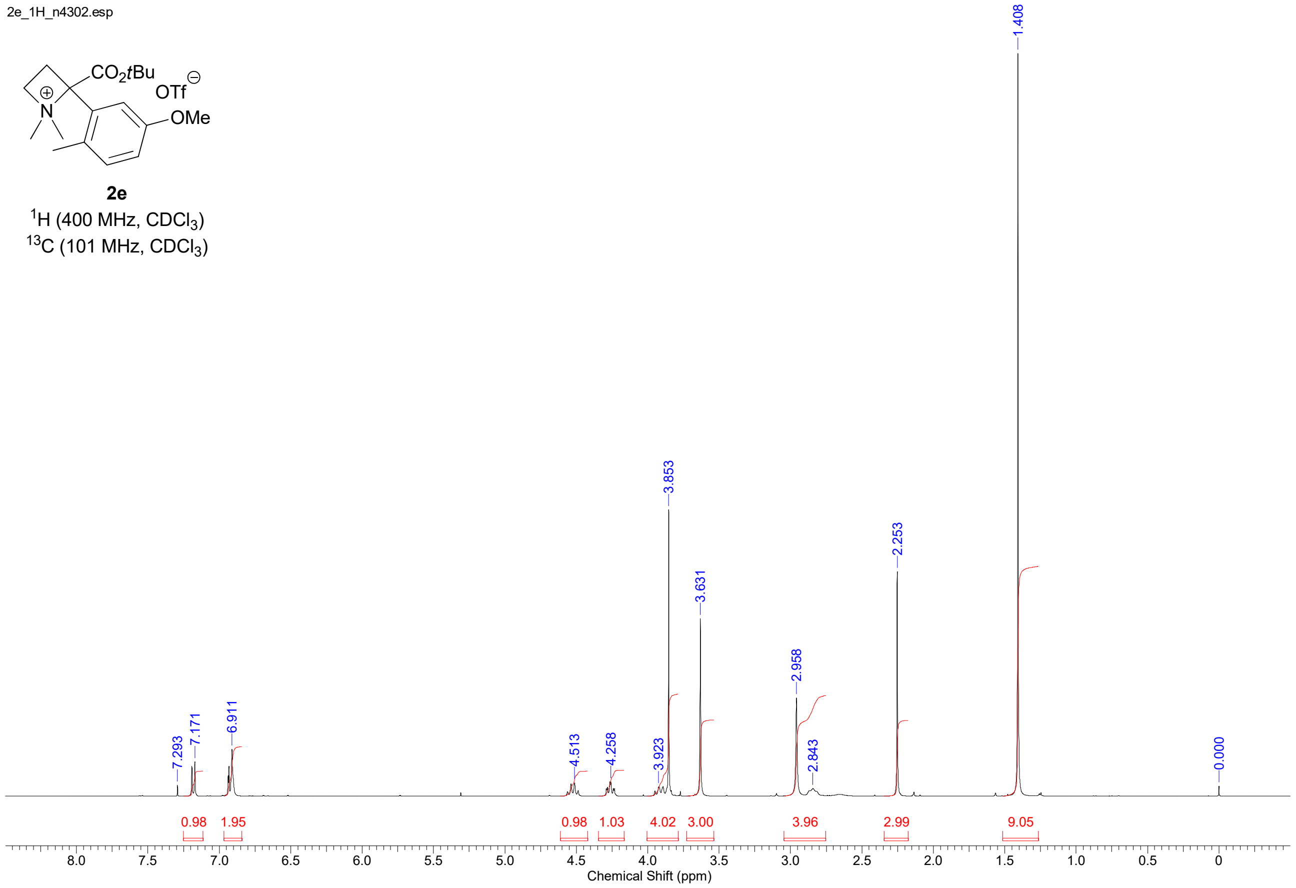

2e

^{1}H (400 MHz, $CDCl_3$)

^{13}C (101 MHz, $CDCl_3$)

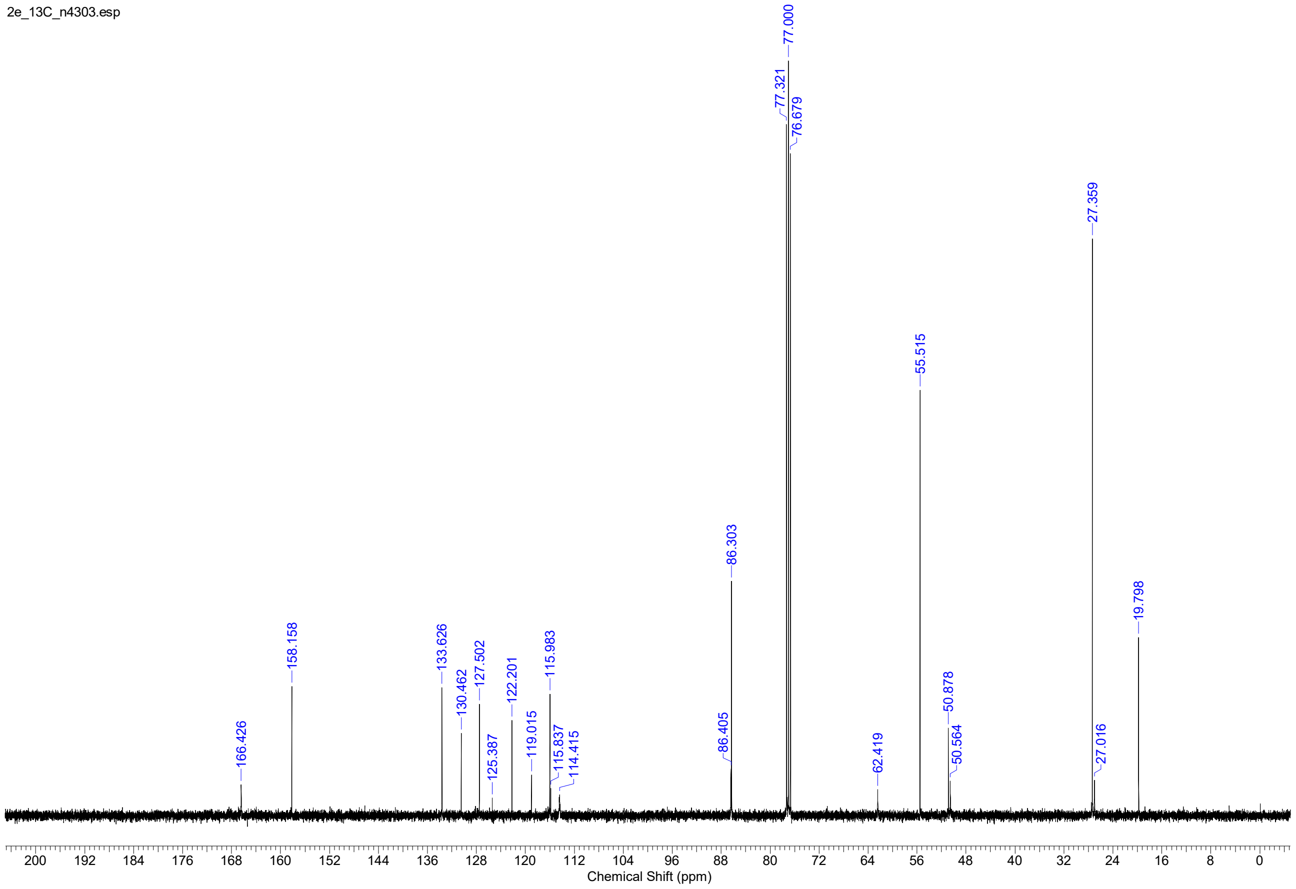

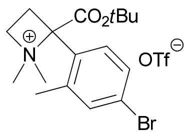

2f

^{1}H (400 MHz, $CDCl_3$)

^{13}C (101 MHz, $CDCl_3$)

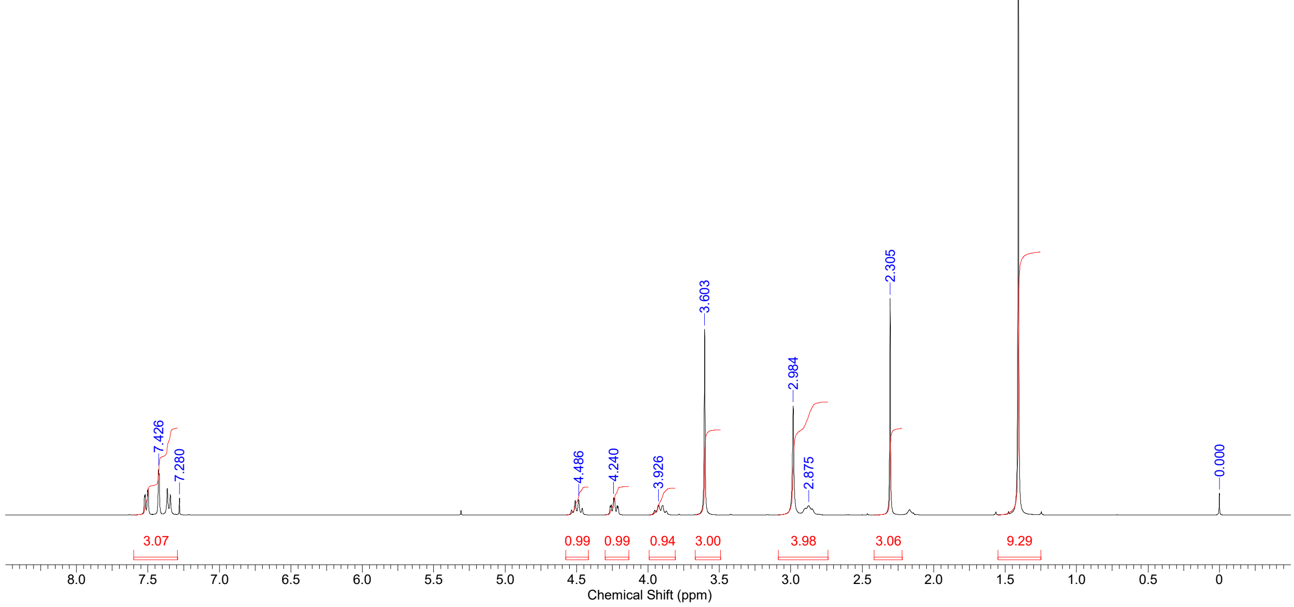

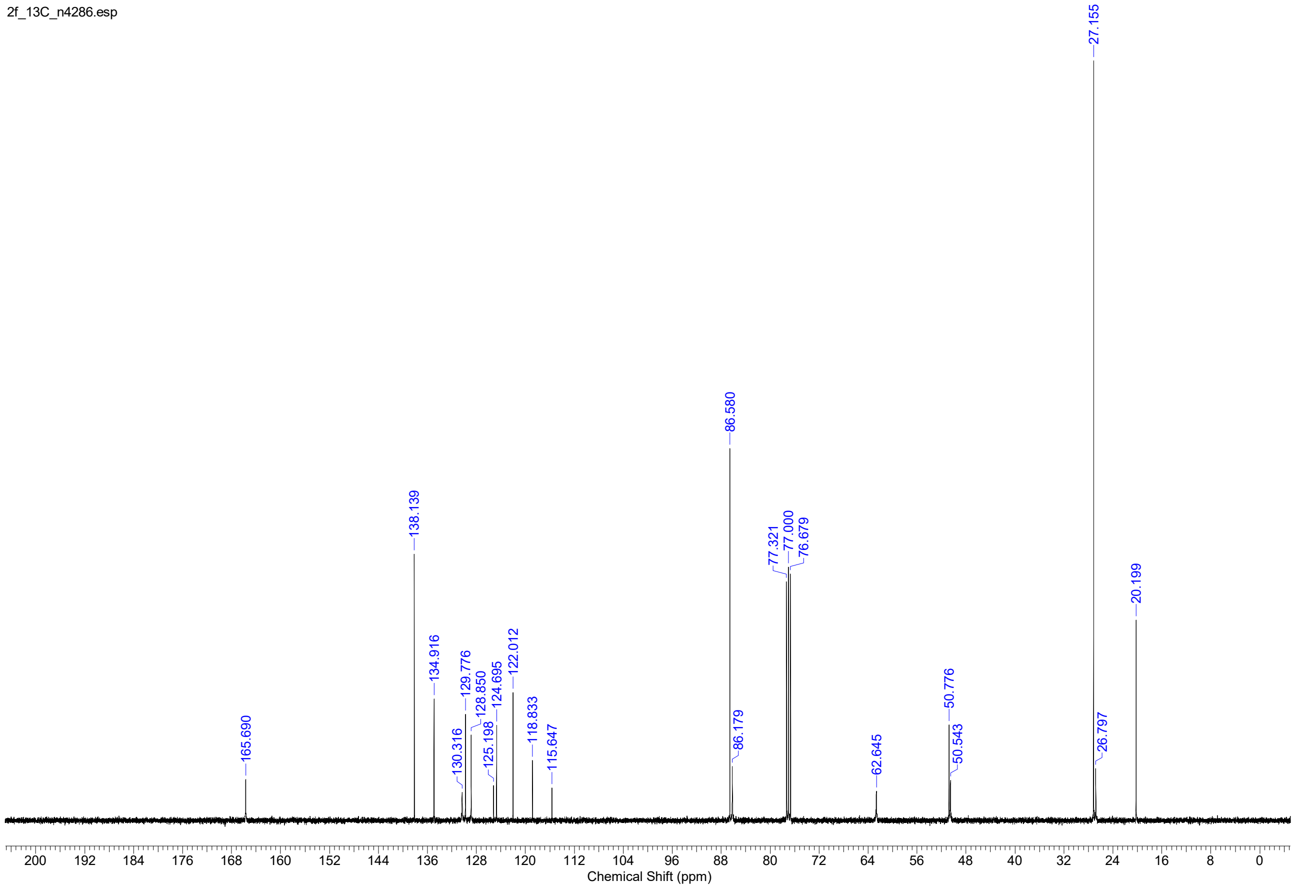

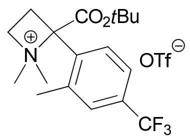

2g

^{1}H (400 MHz, $CDCl_3$)
^{13}C (101 MHz, $CDCl_3$)

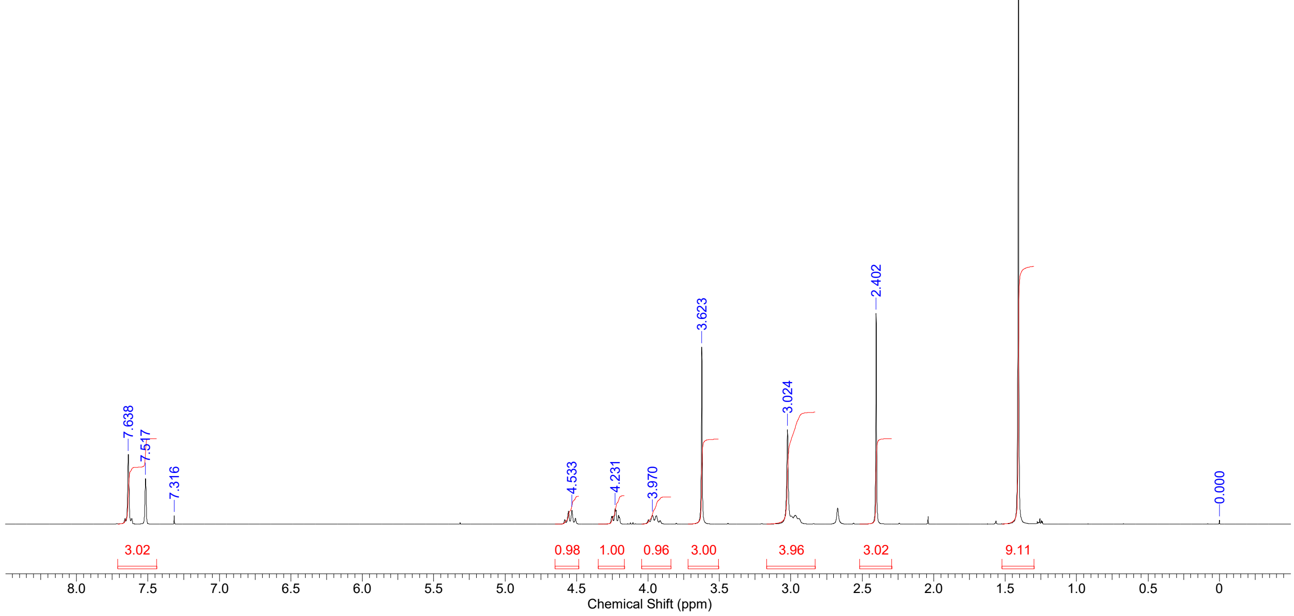

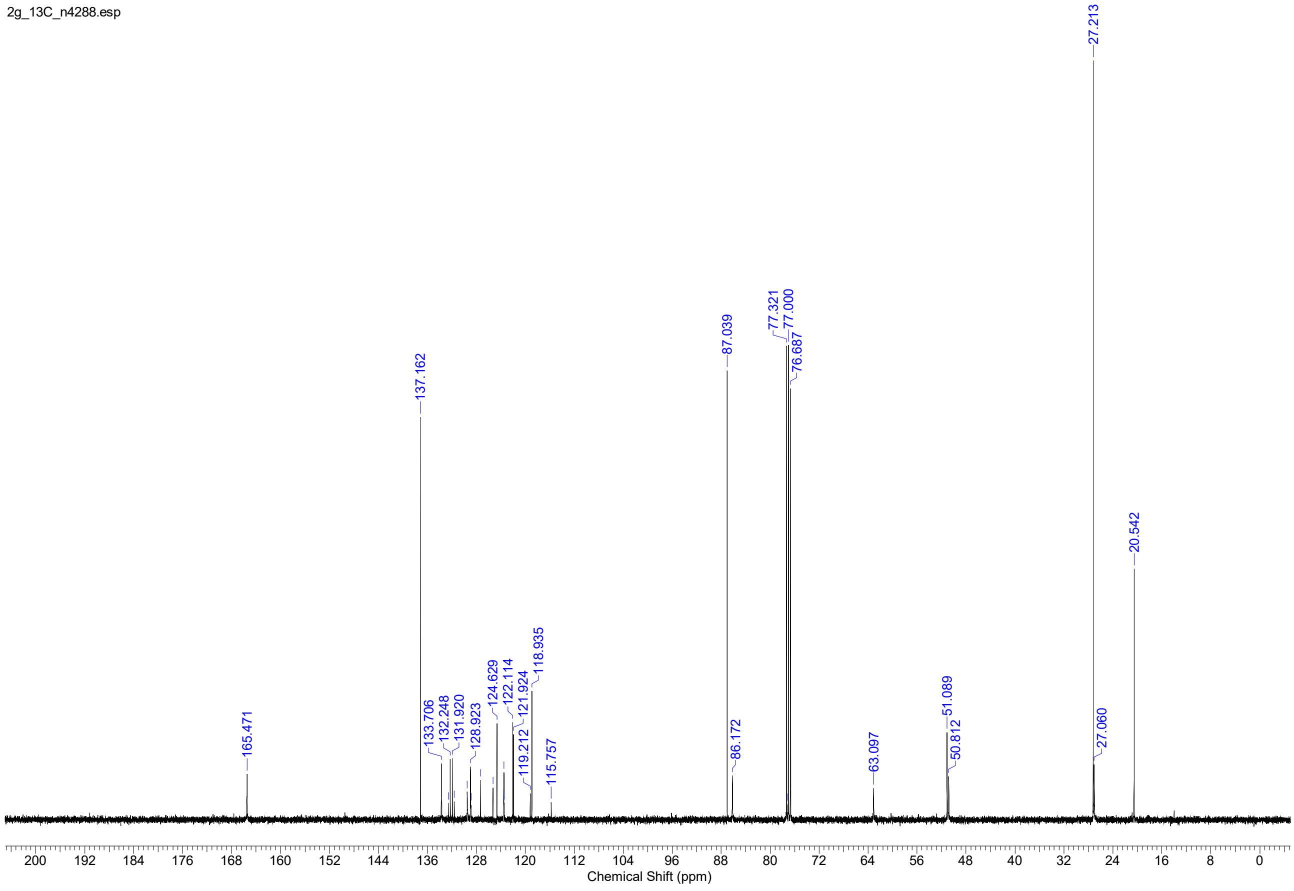

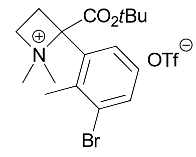

2h

^{1}H (400 MHz, $CDCl_3$)

^{13}C (101 MHz, $CDCl_3$)

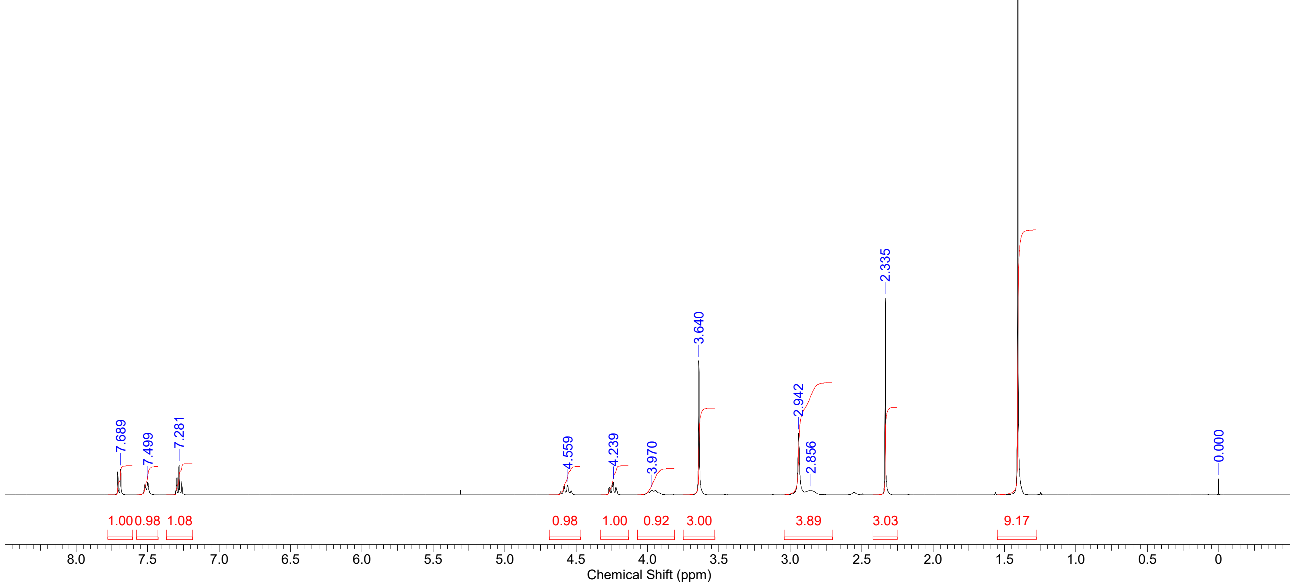

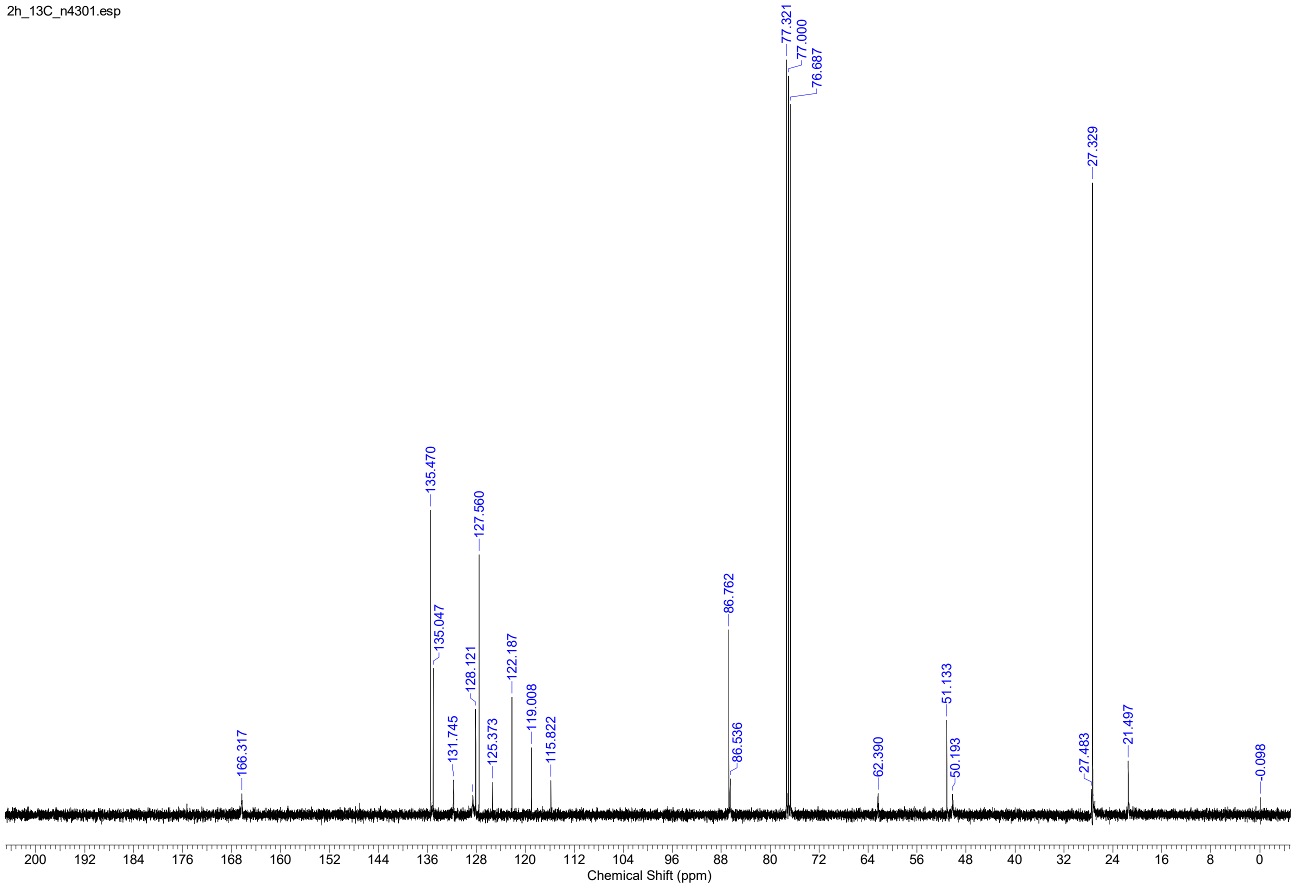

3aa
^{1}H (400 MHz, $CDCl_3$)
^{13}C (101 MHz, $CDCl_3$)

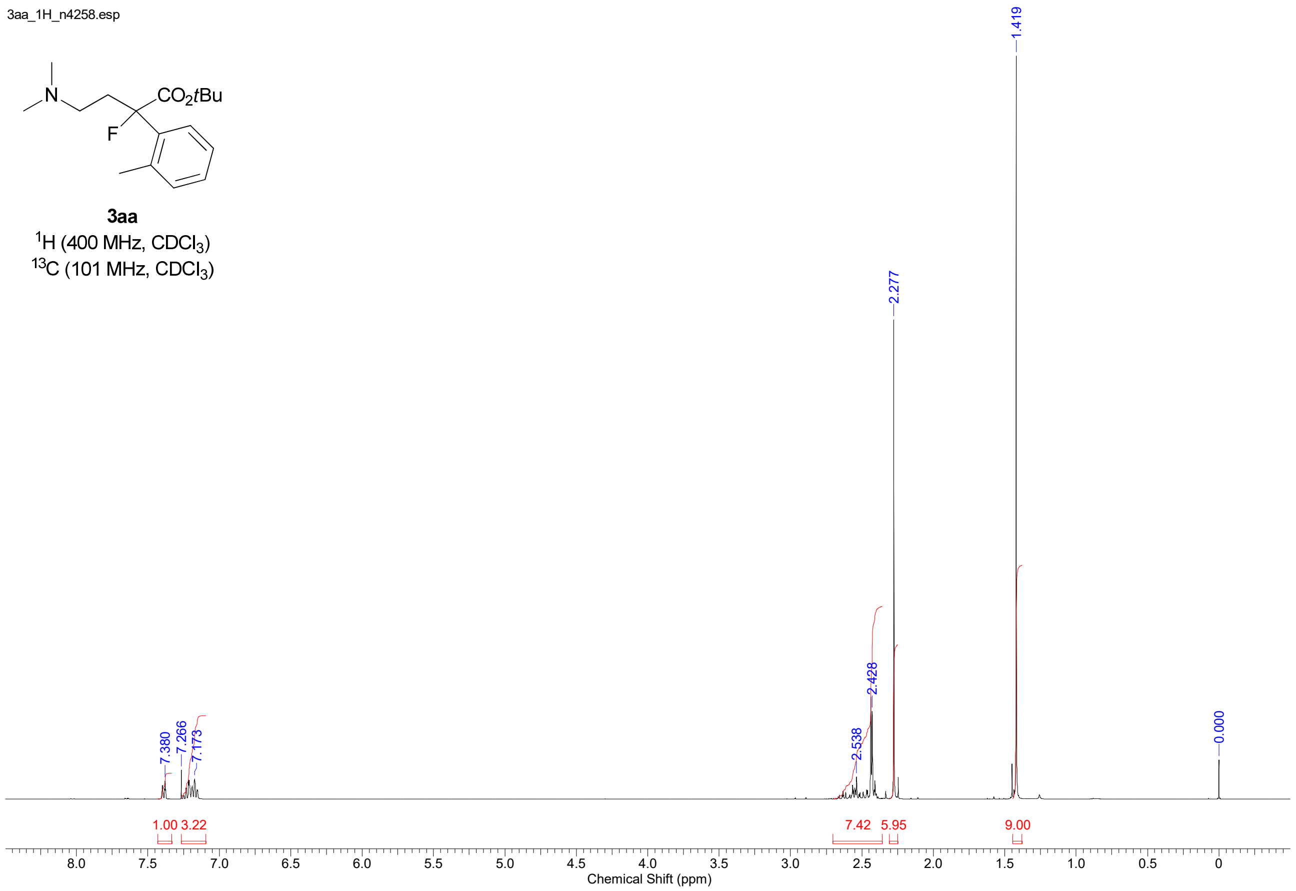

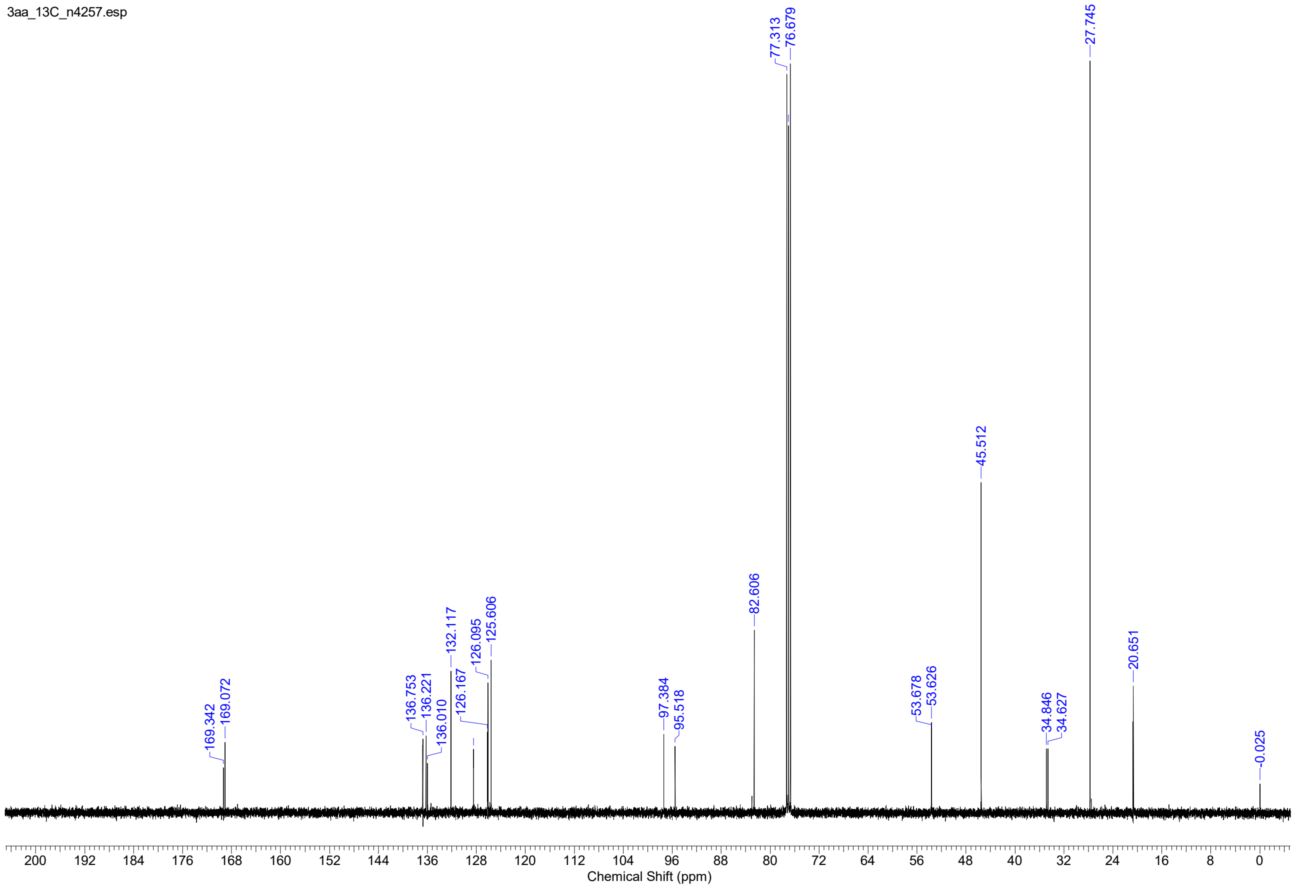

3aa_19F_4549.esp

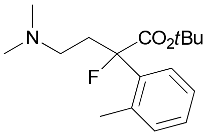

3aa
^{19}F (376 MHz, $CDCl_3$)
C_6F_6: δ –162.9 ppm

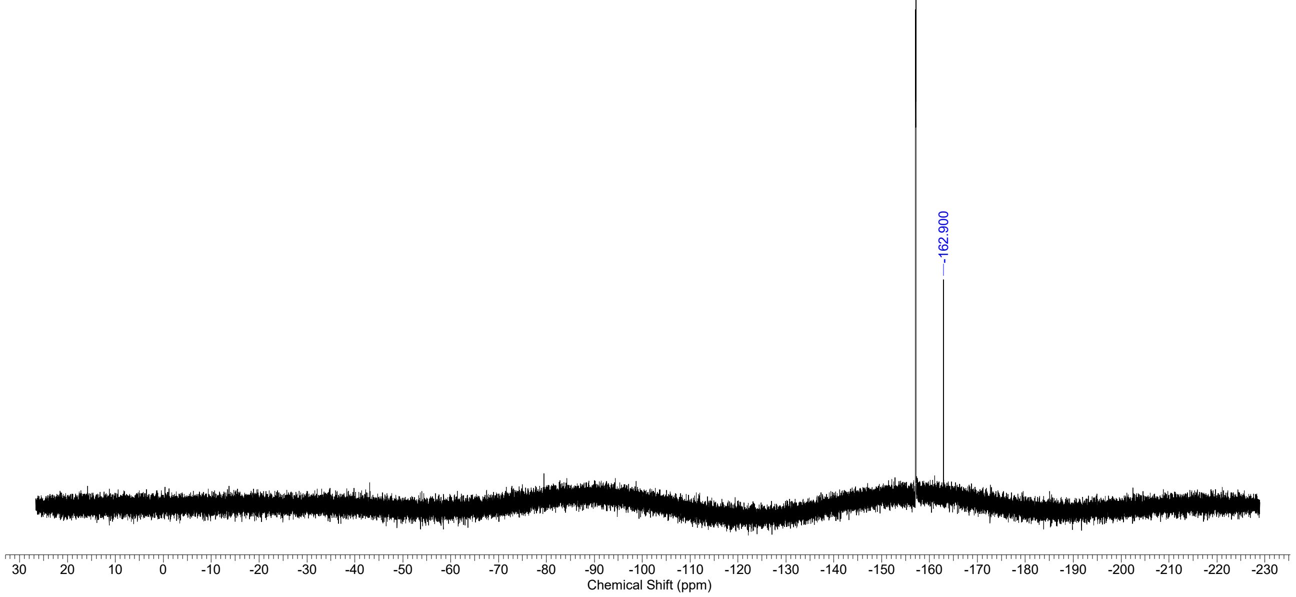

4aa

^{1}H (400 MHz, $CDCl_3$)

^{13}C (101 MHz, $CDCl_3$)

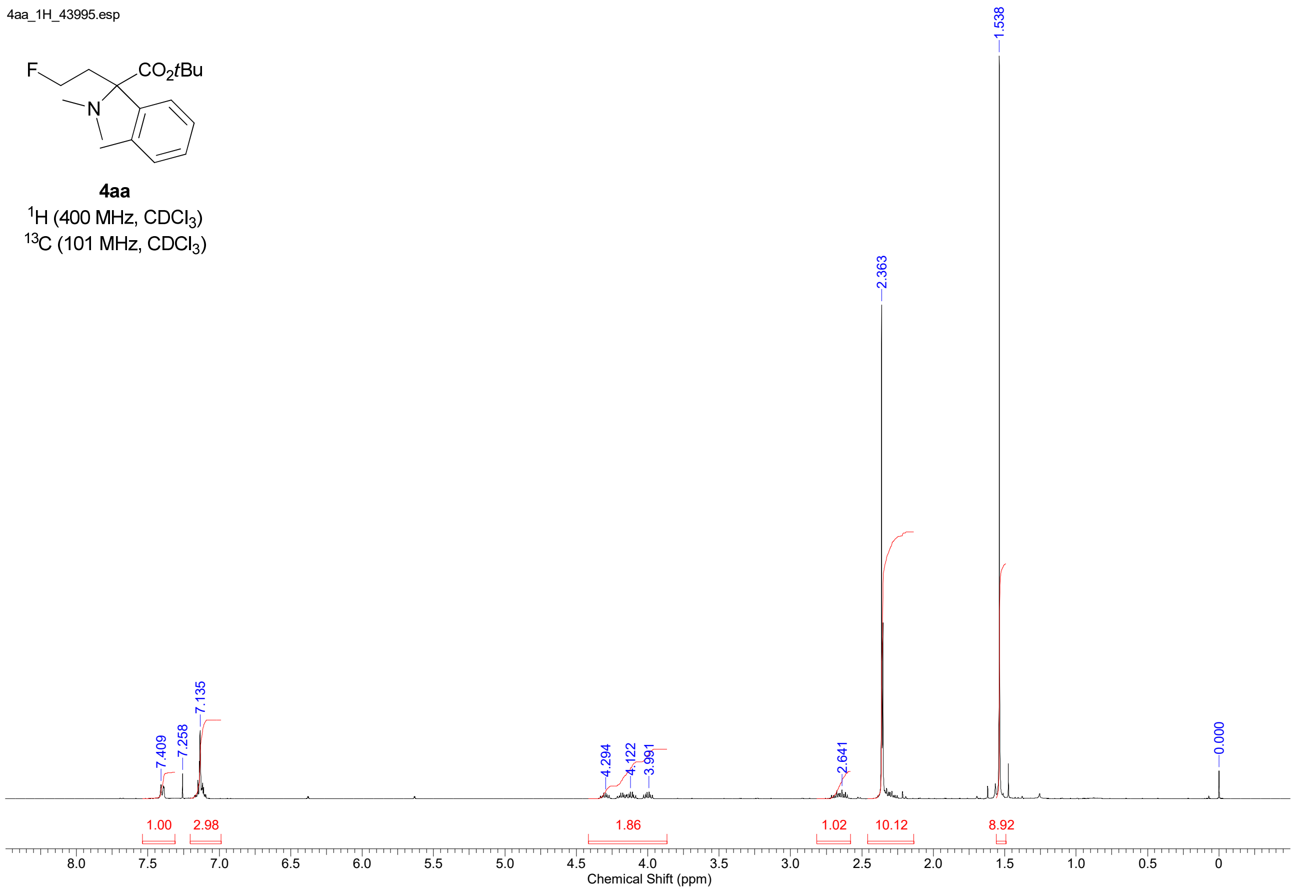

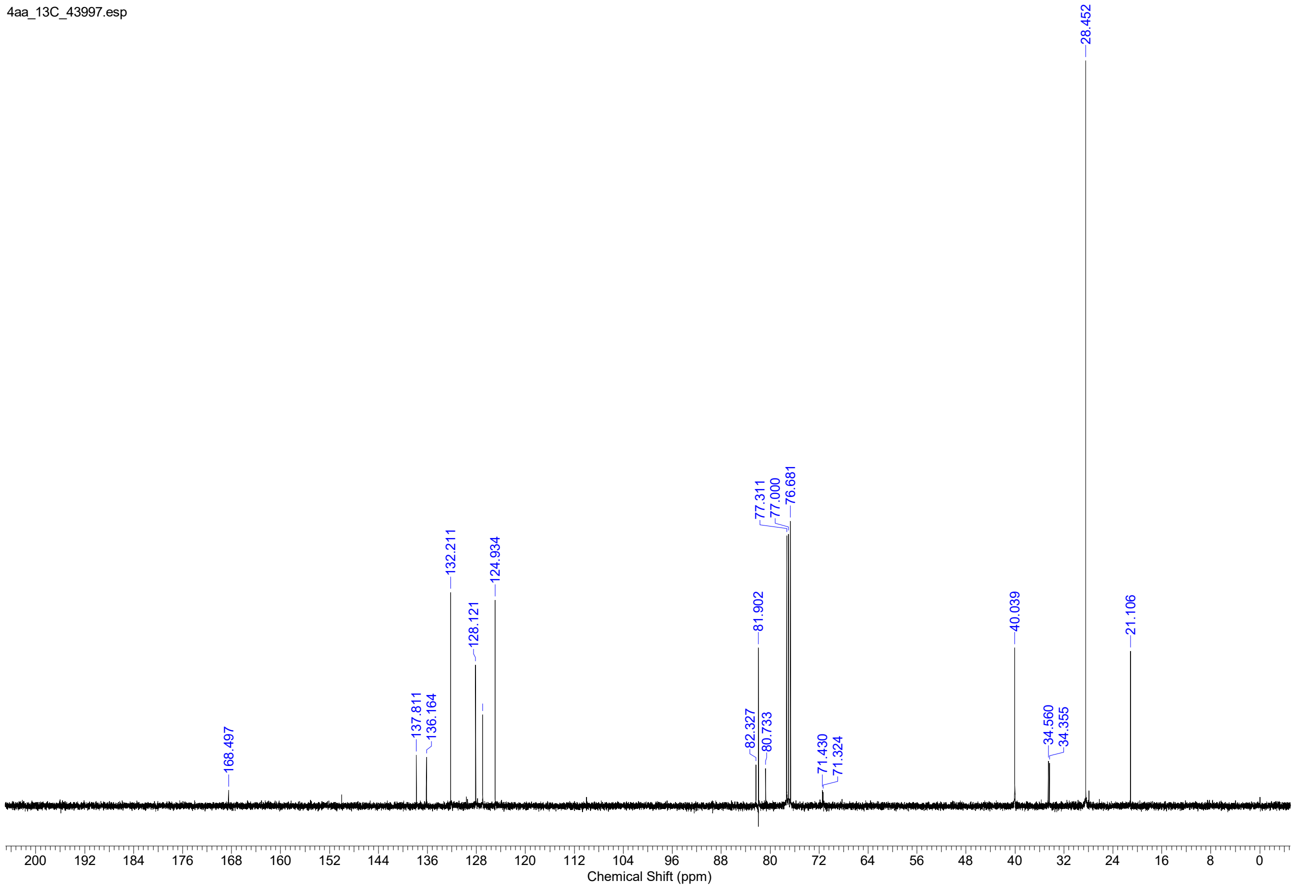

4aa_19F_44002.esp

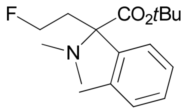

4aa

^{19}F (376 MHz, $CDCl_3$)

C_6F_6: δ –162.9 ppm

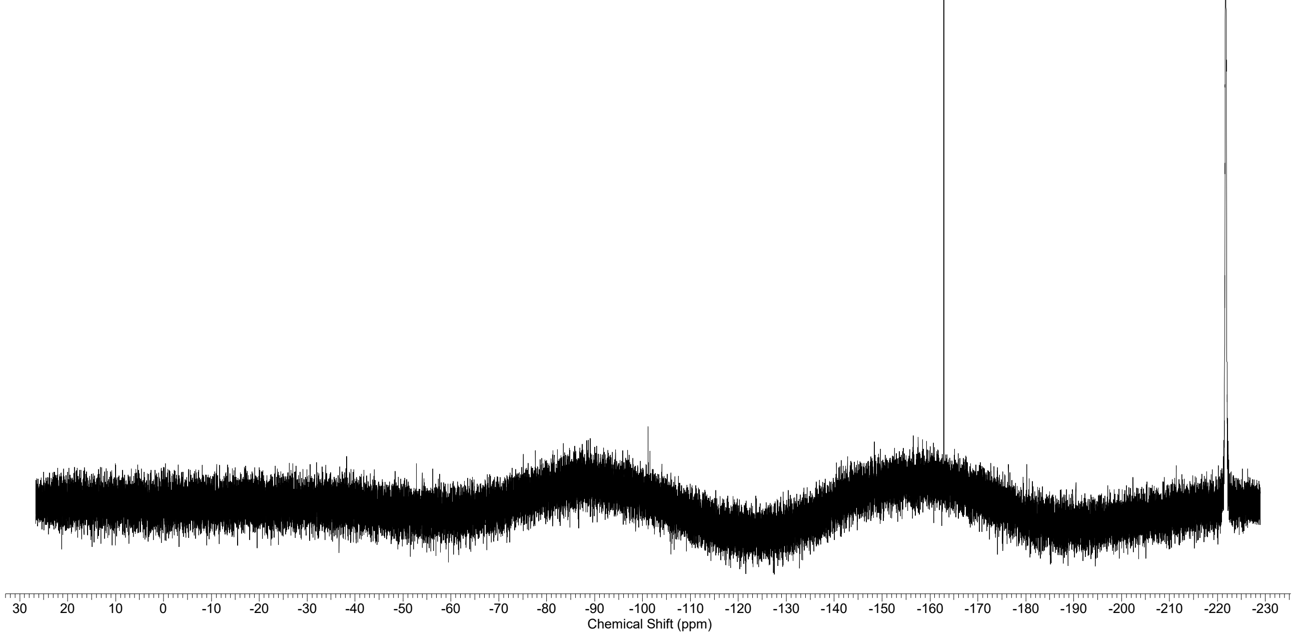

3ab

^1H (400 MHz, $CDCl_3$)

^{13}C (101 MHz, $CDCl_3$)

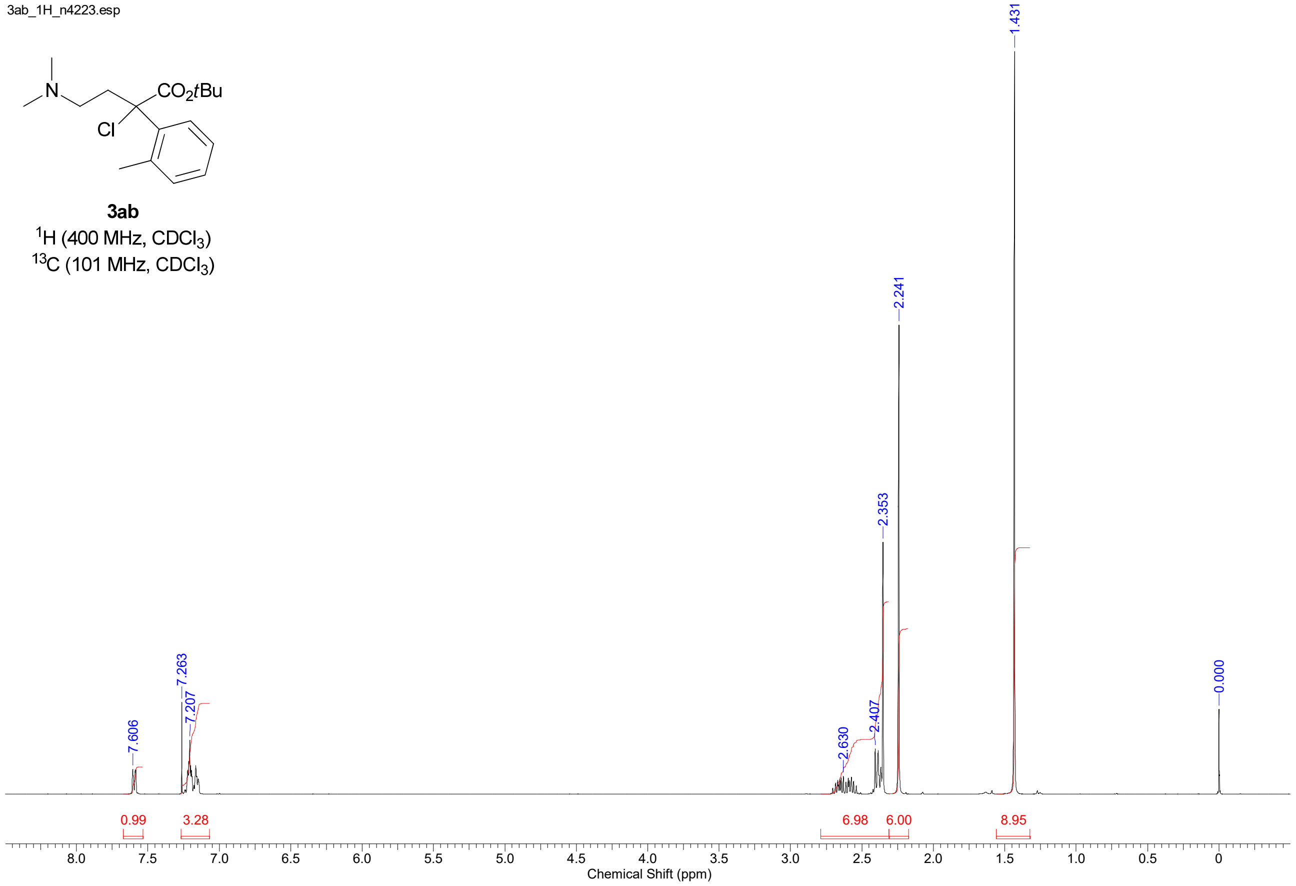

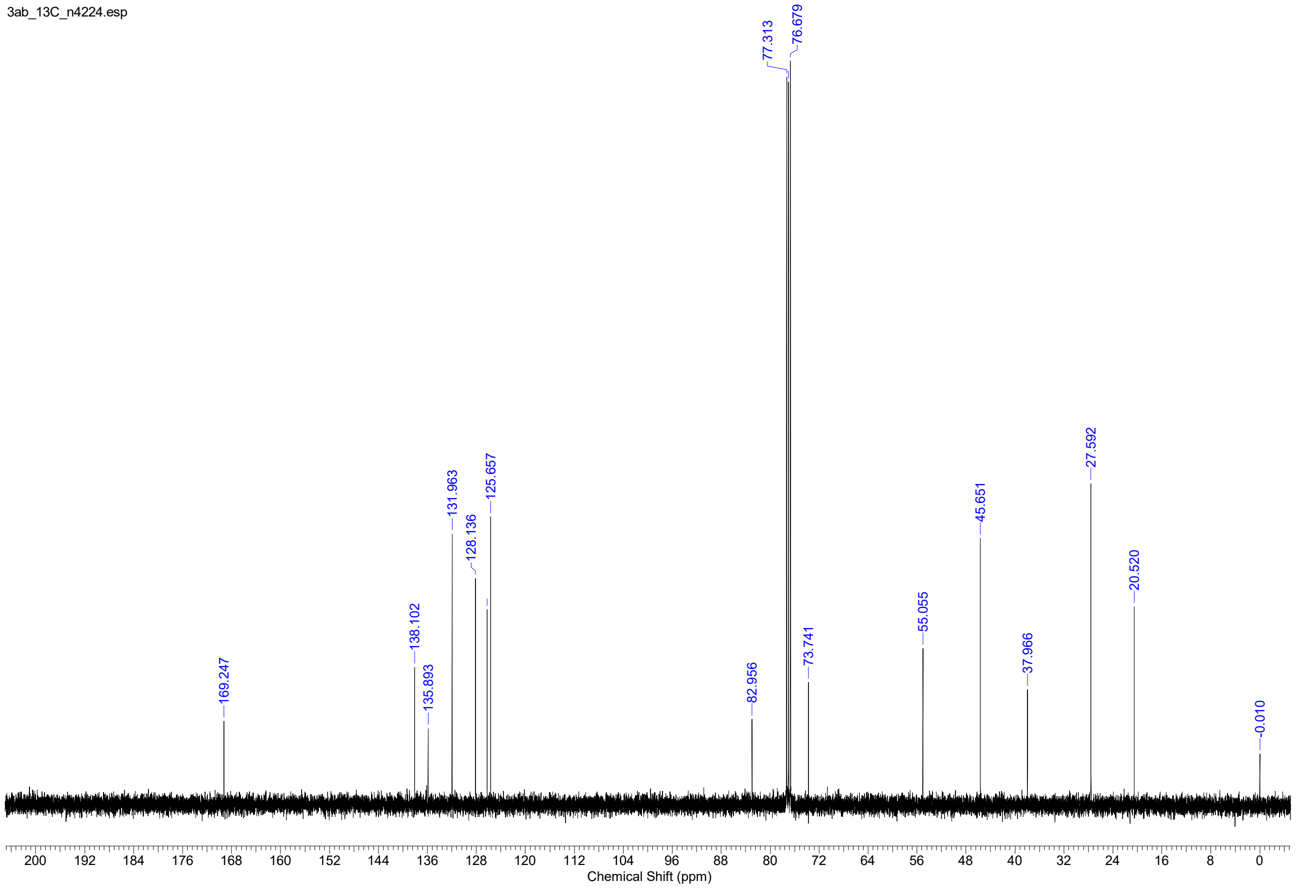

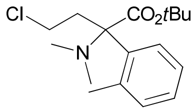

4ab

^{1}H (400 MHz, $CDCl_3$)

^{13}C (101 MHz, $CDCl_3$)

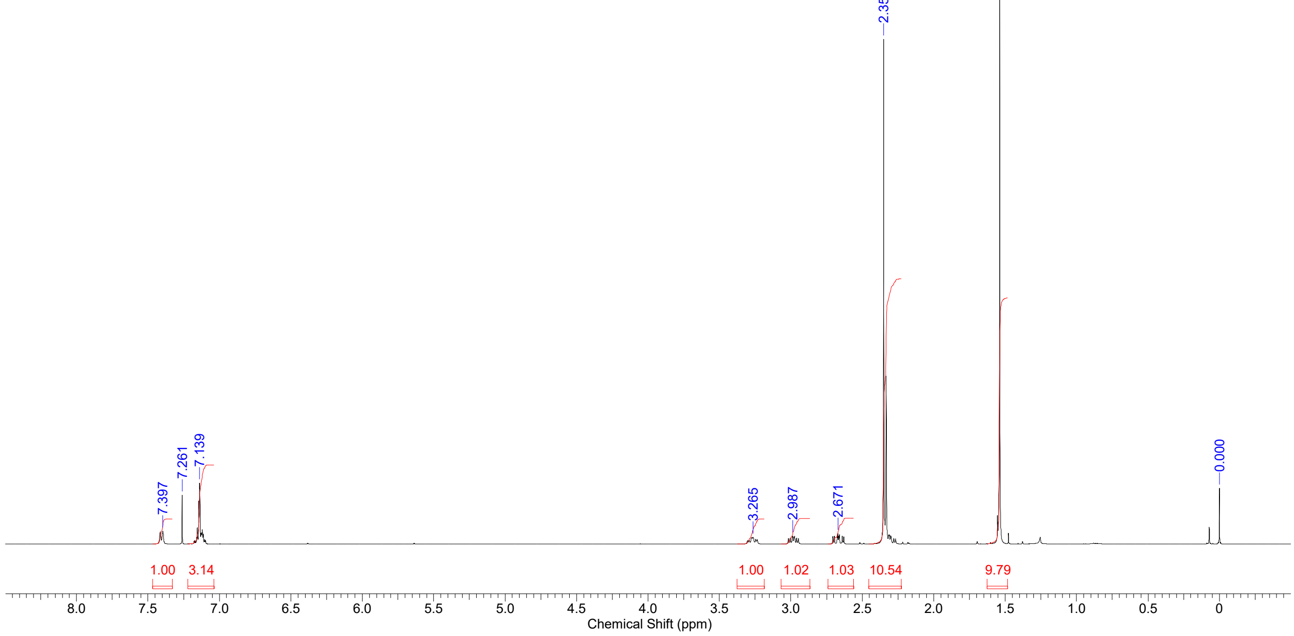

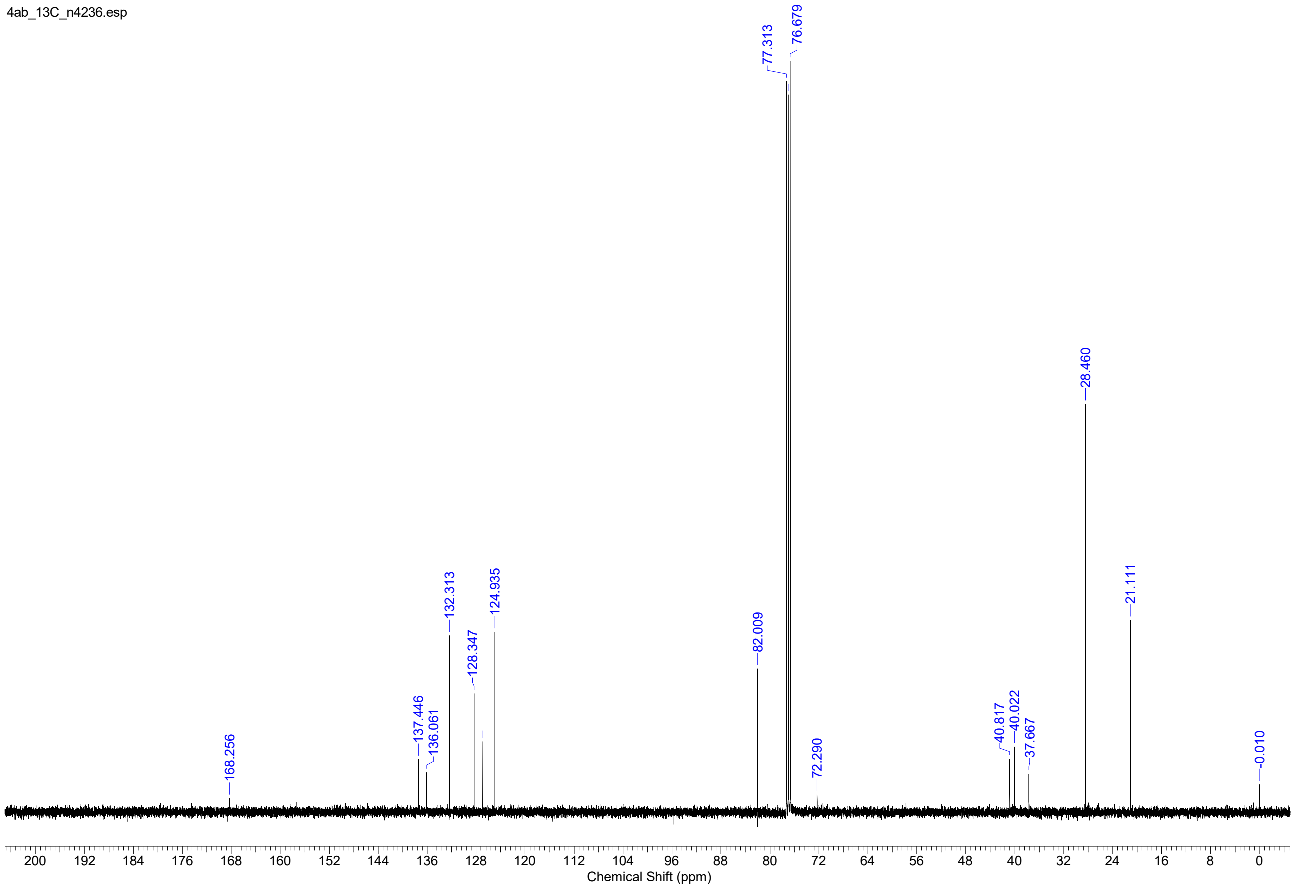

3ac_1H_n4247.esp

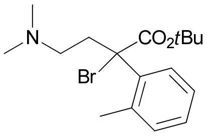

3ac
^1H (400 MHz, $CDCl_3$)
^{13}C (101 MHz, $CDCl_3$)

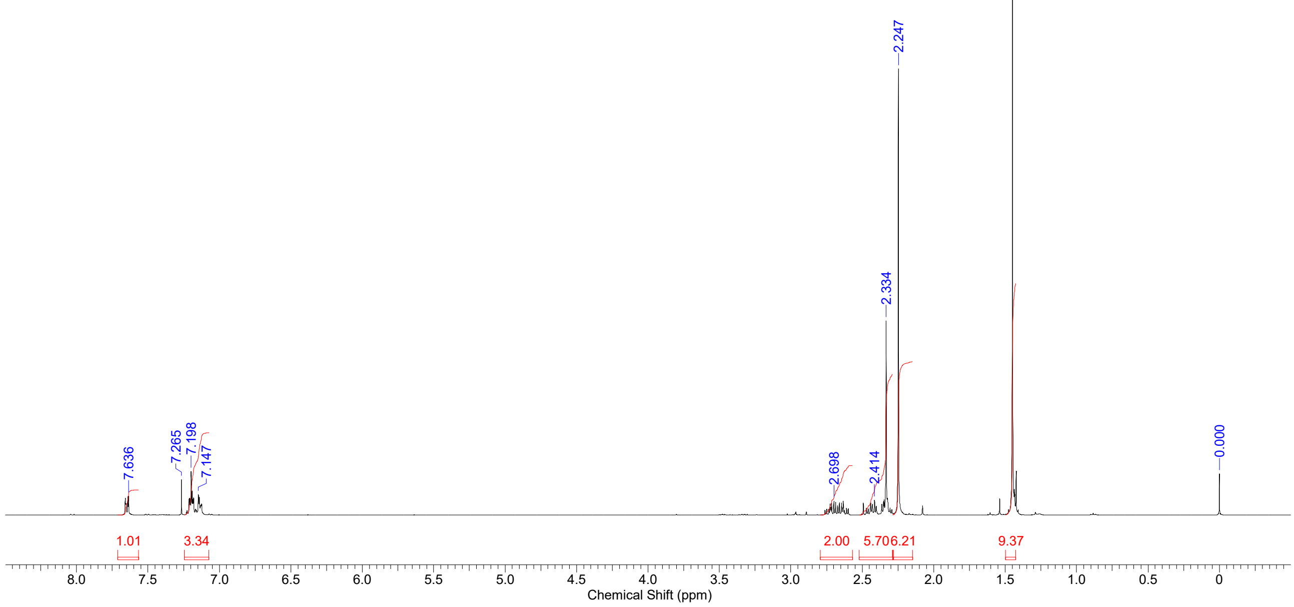

3ac_13C_n4248.esp

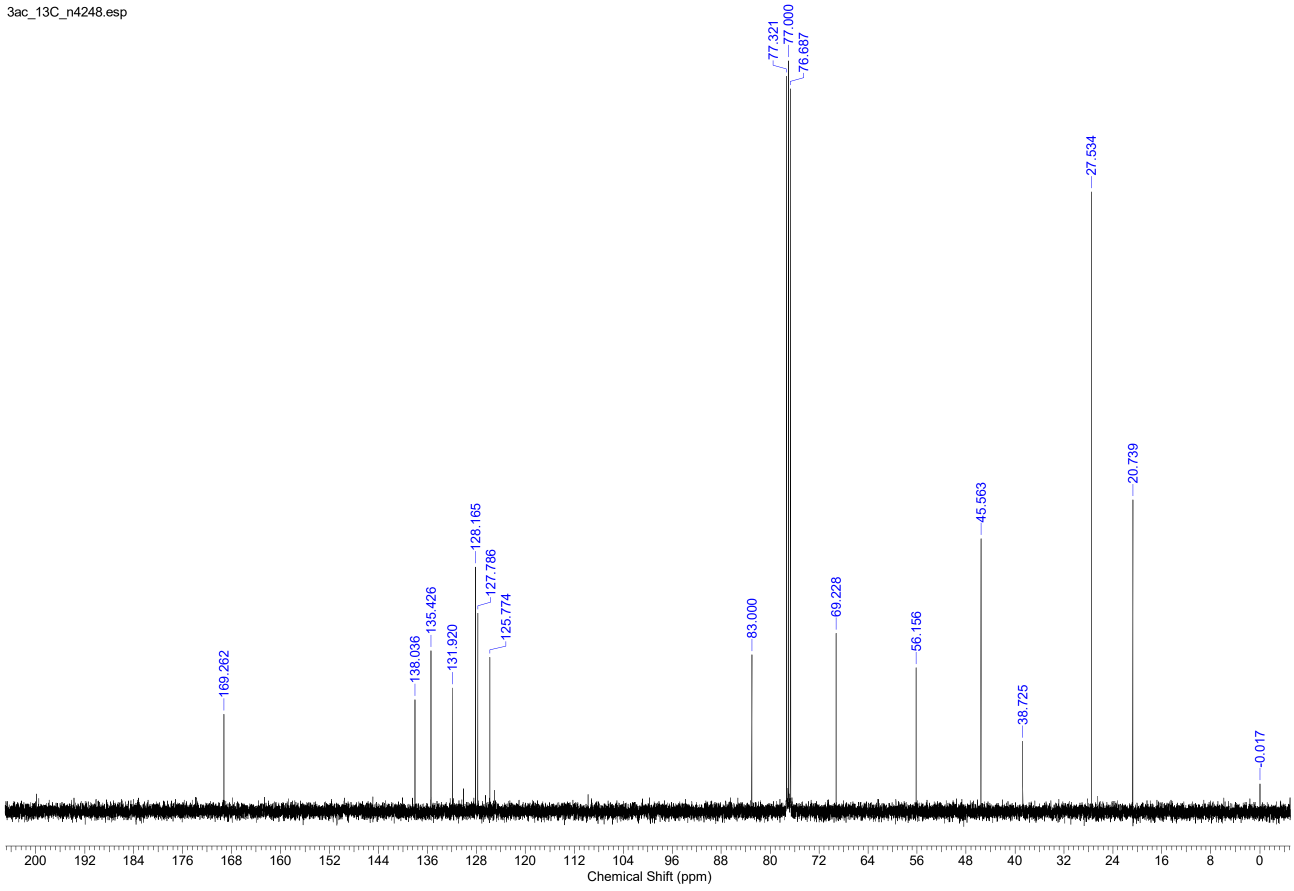

4ac_1H_n4245.esp

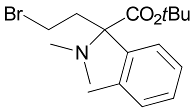

4ac

^{1}H (400 MHz, $CDCl_3$)

^{13}C (101 MHz, $CDCl_3$)

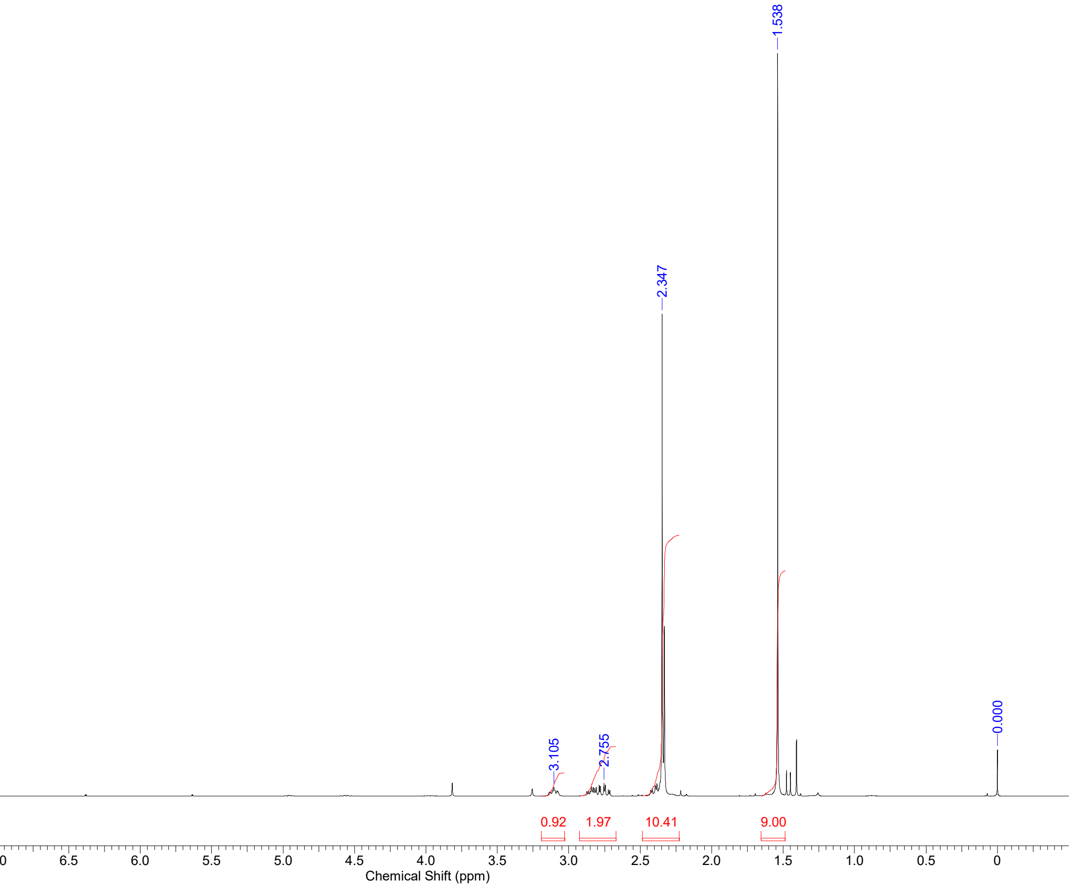

4ac_13C_n4246.esp

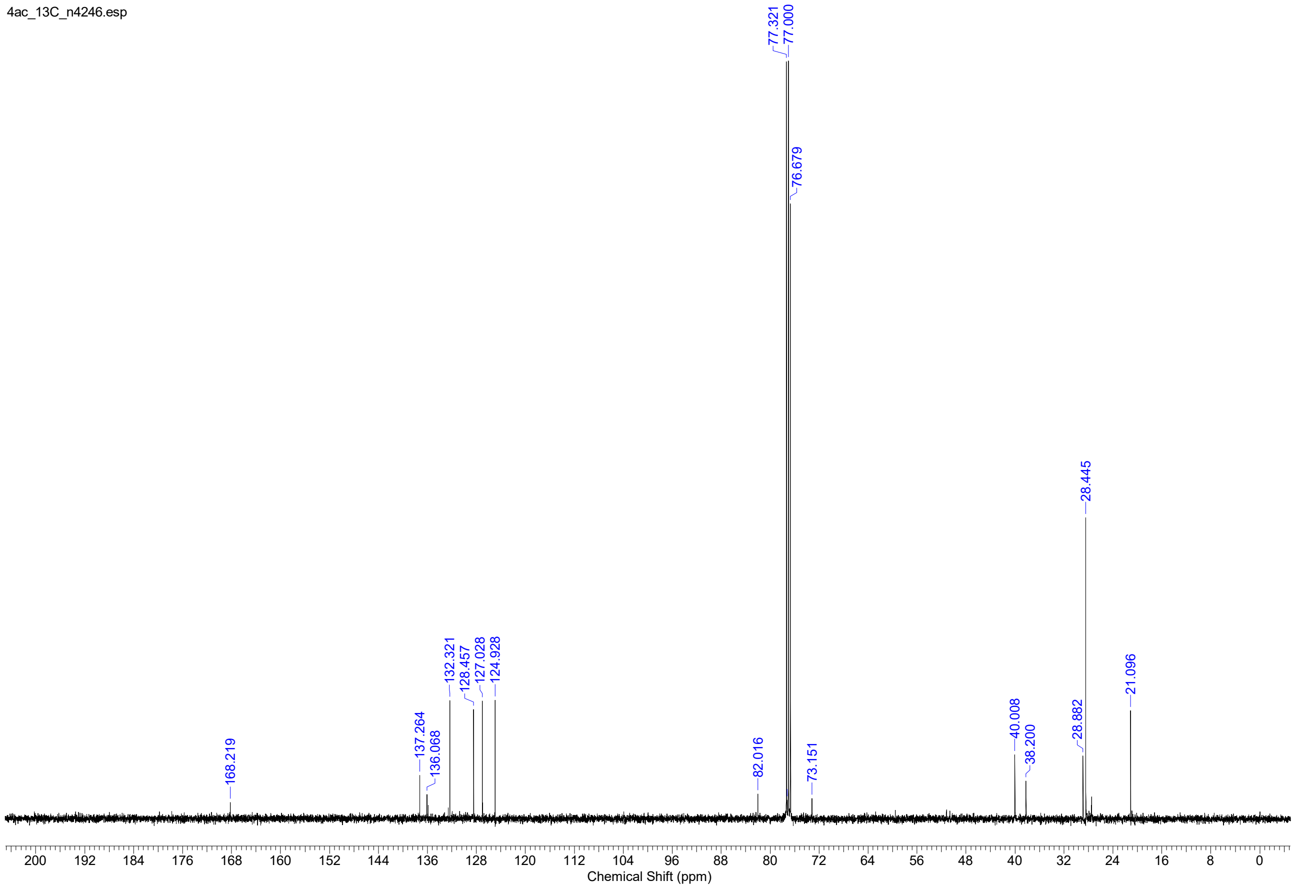

3ae_1H_4280.esp

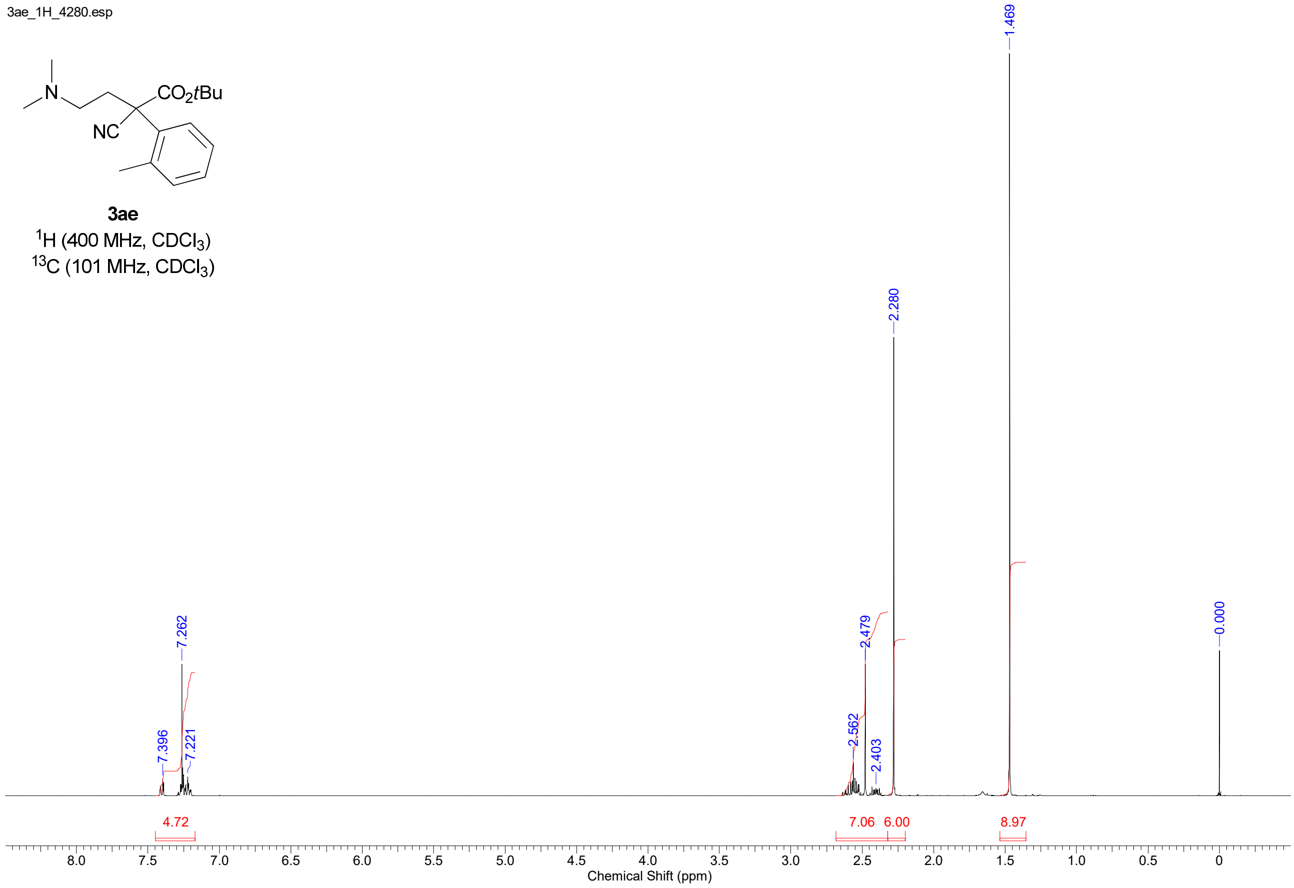

3ae_13C_n4193.esp

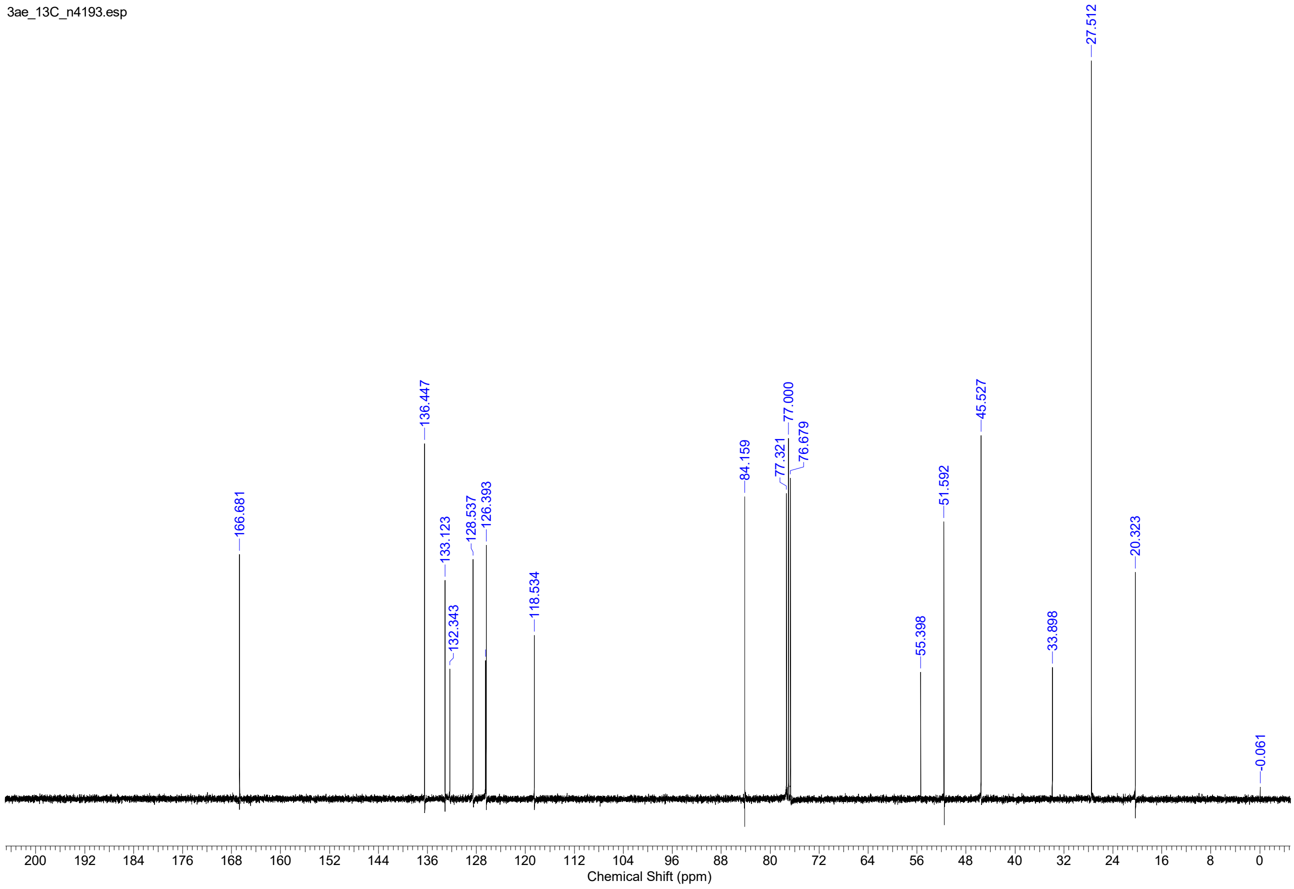

4ae_1H_4532.esp

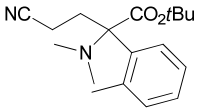

4ae

^{1}H (400 MHz, $CDCl_3$)

^{13}C (101 MHz, $CDCl_3$)

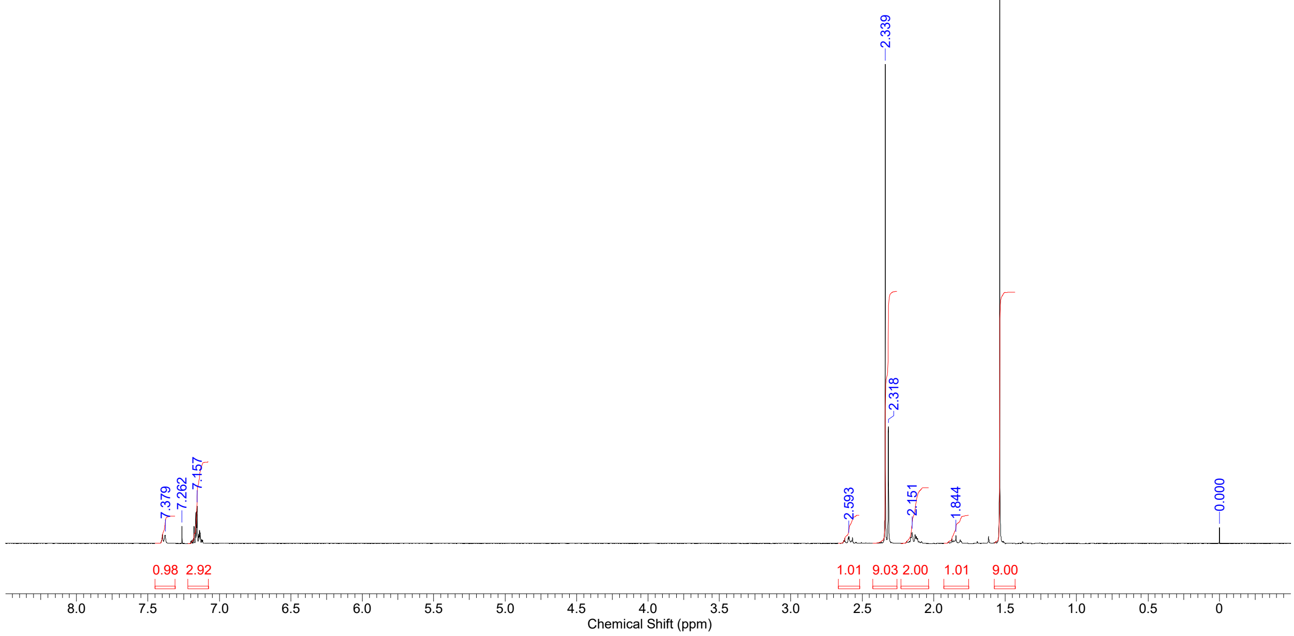

4ae_13C_4438.esp

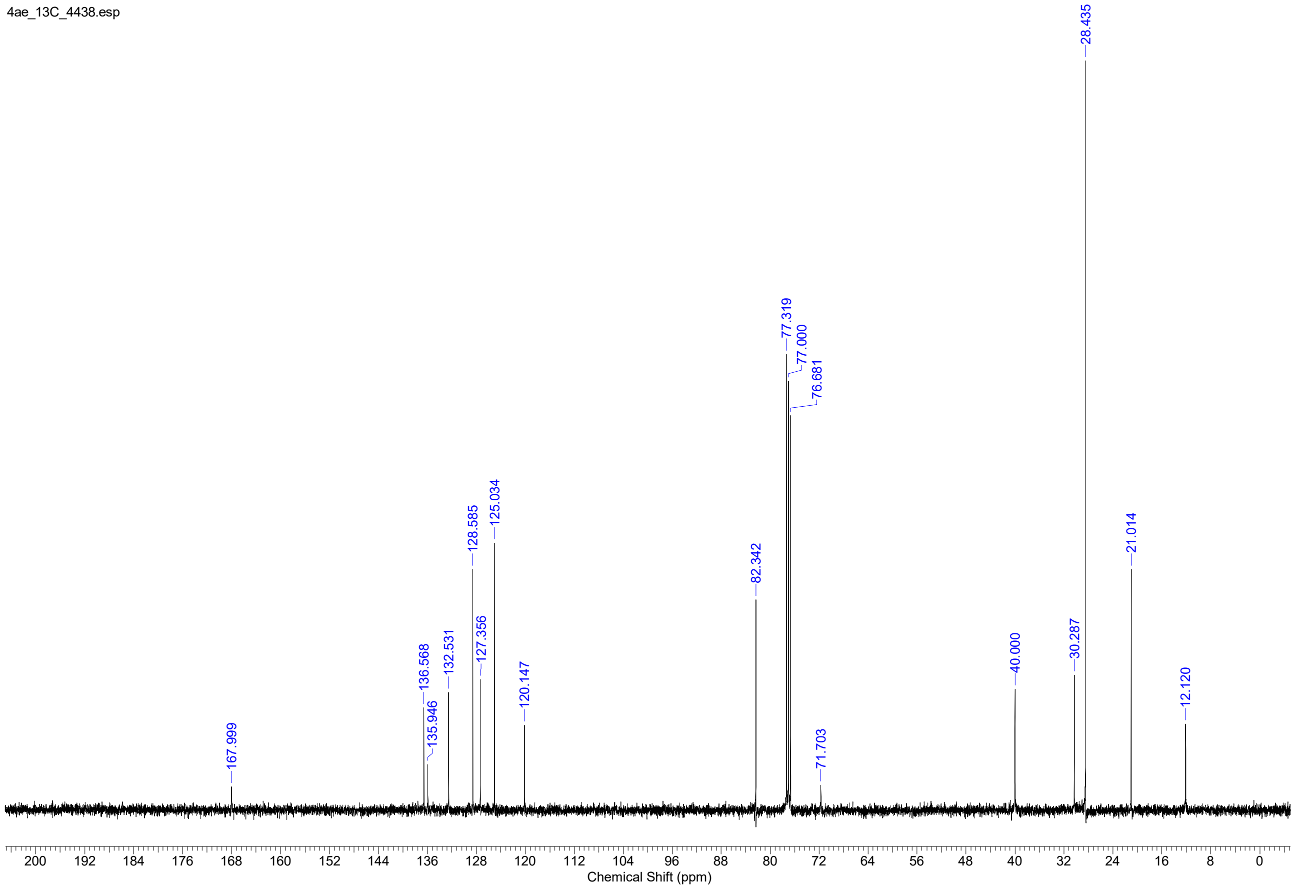

3ba_1H_n4232.esp

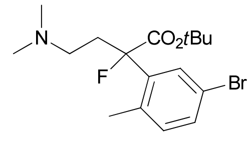

3ba

^{1}H (400 MHz, $CDCl_3$)

^{13}C (101 MHz, $CDCl_3$)

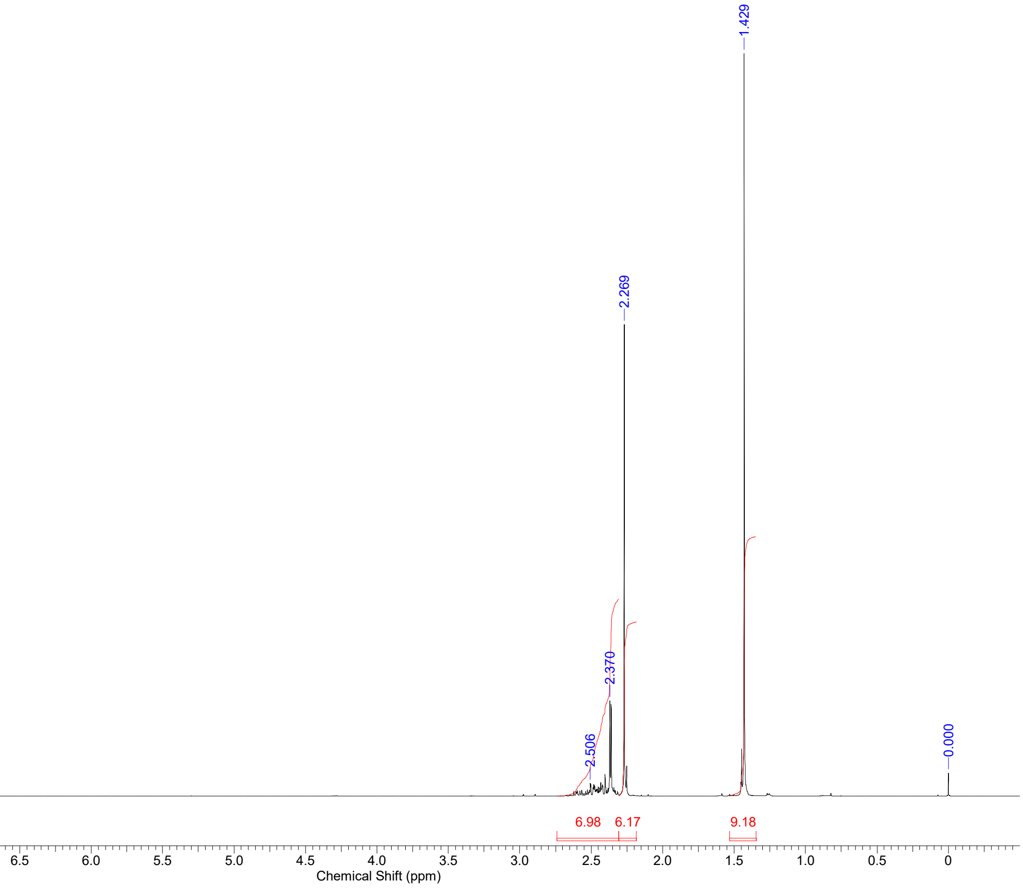

3ba_13C_4583.esp

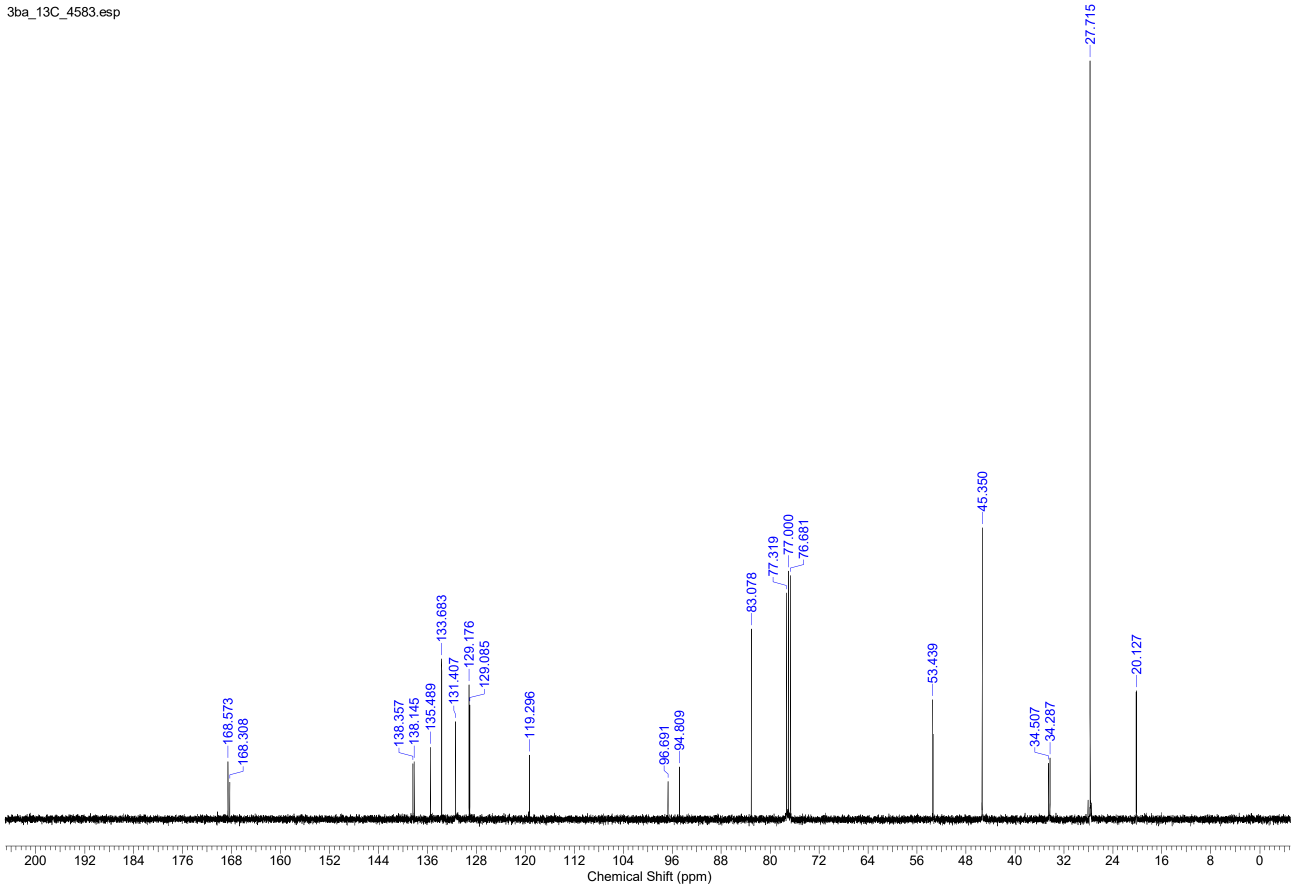

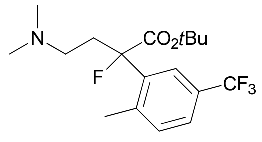

3ca

^{1}H (400 MHz, $CDCl_3$)

^{13}C (101 MHz, $CDCl_3$)

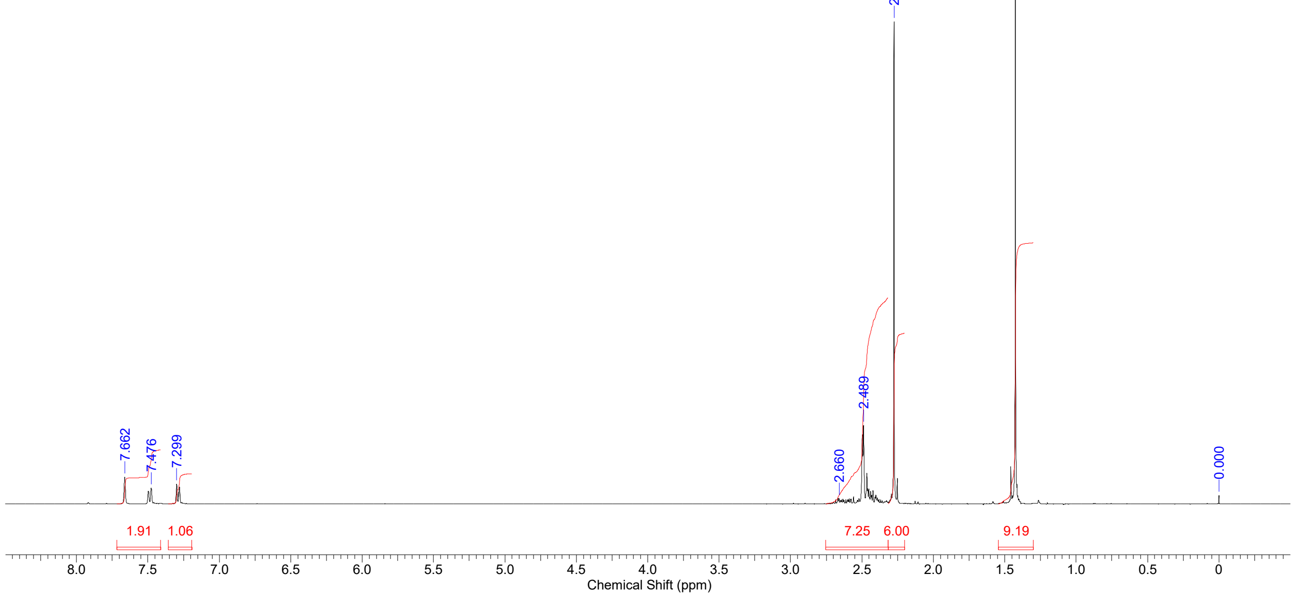

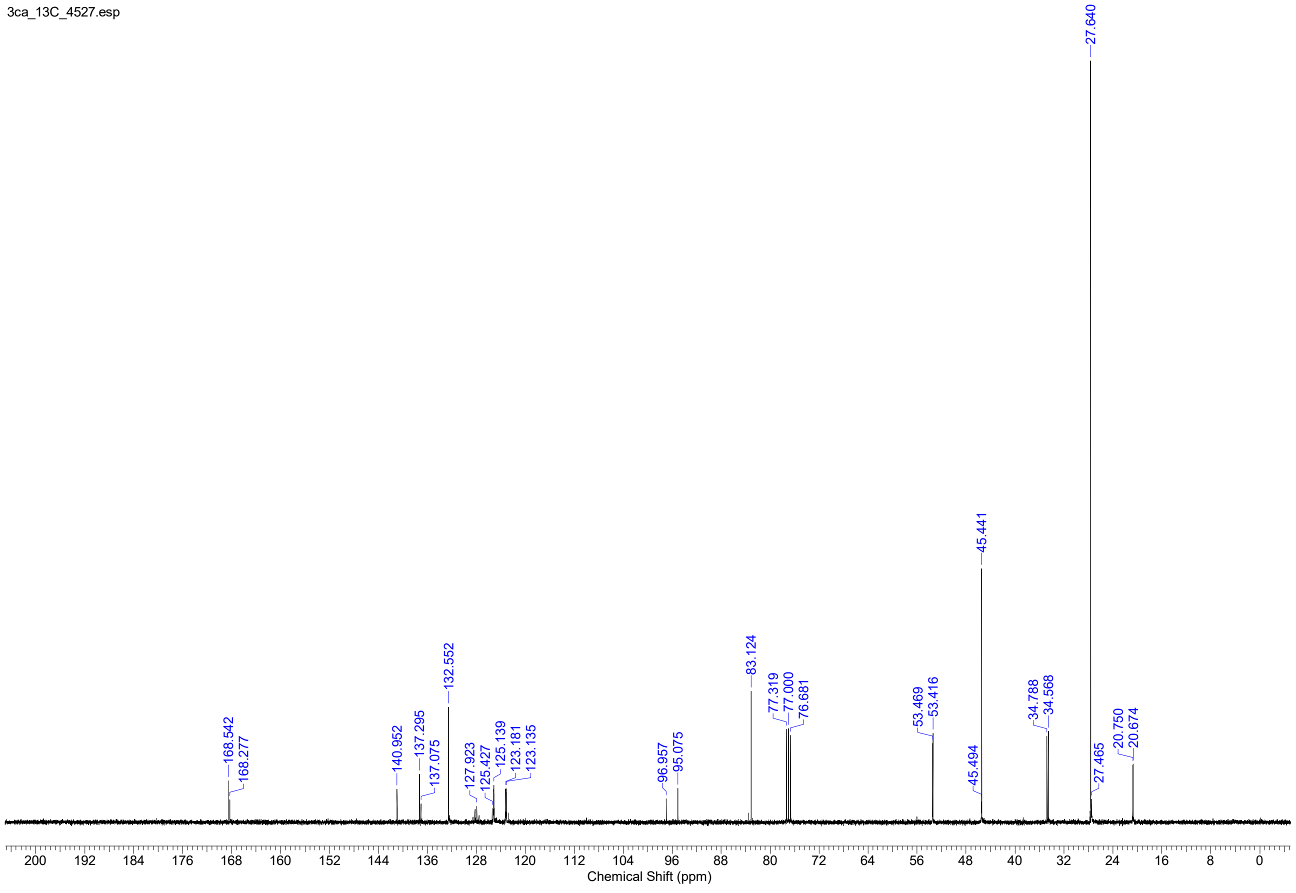

3da_1H_4504.esp

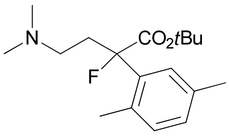

3da

^{1}H (400 MHz, $CDCl_3$)

^{13}C (101 MHz, $CDCl_3$)

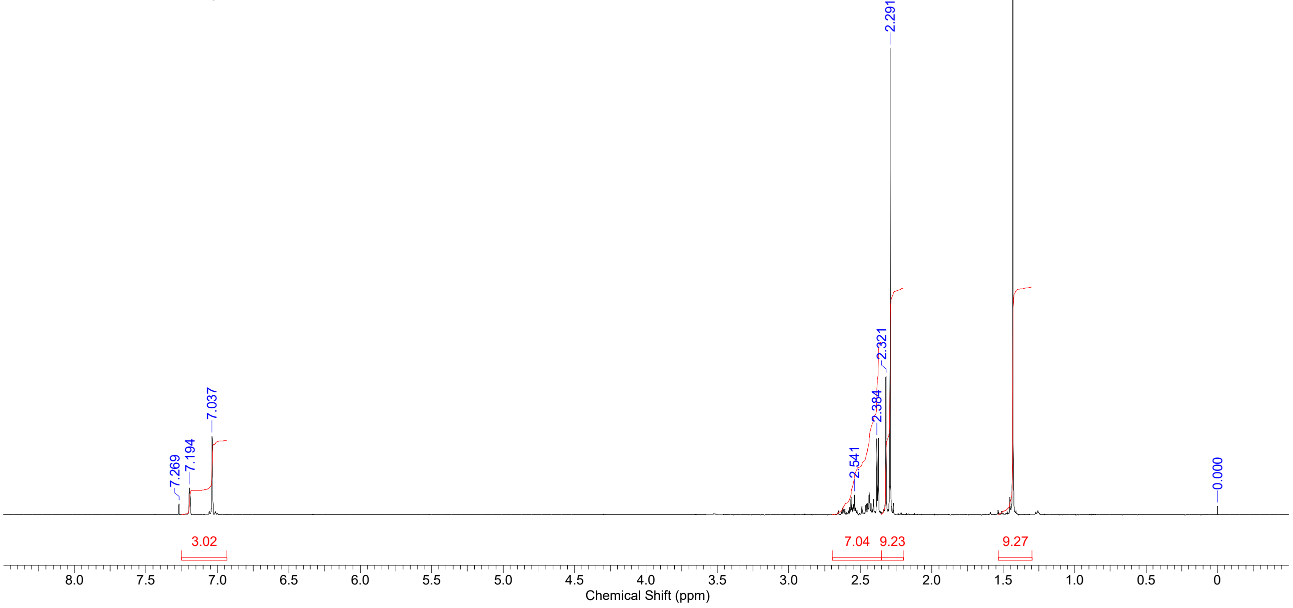

3da_13C_4505.esp

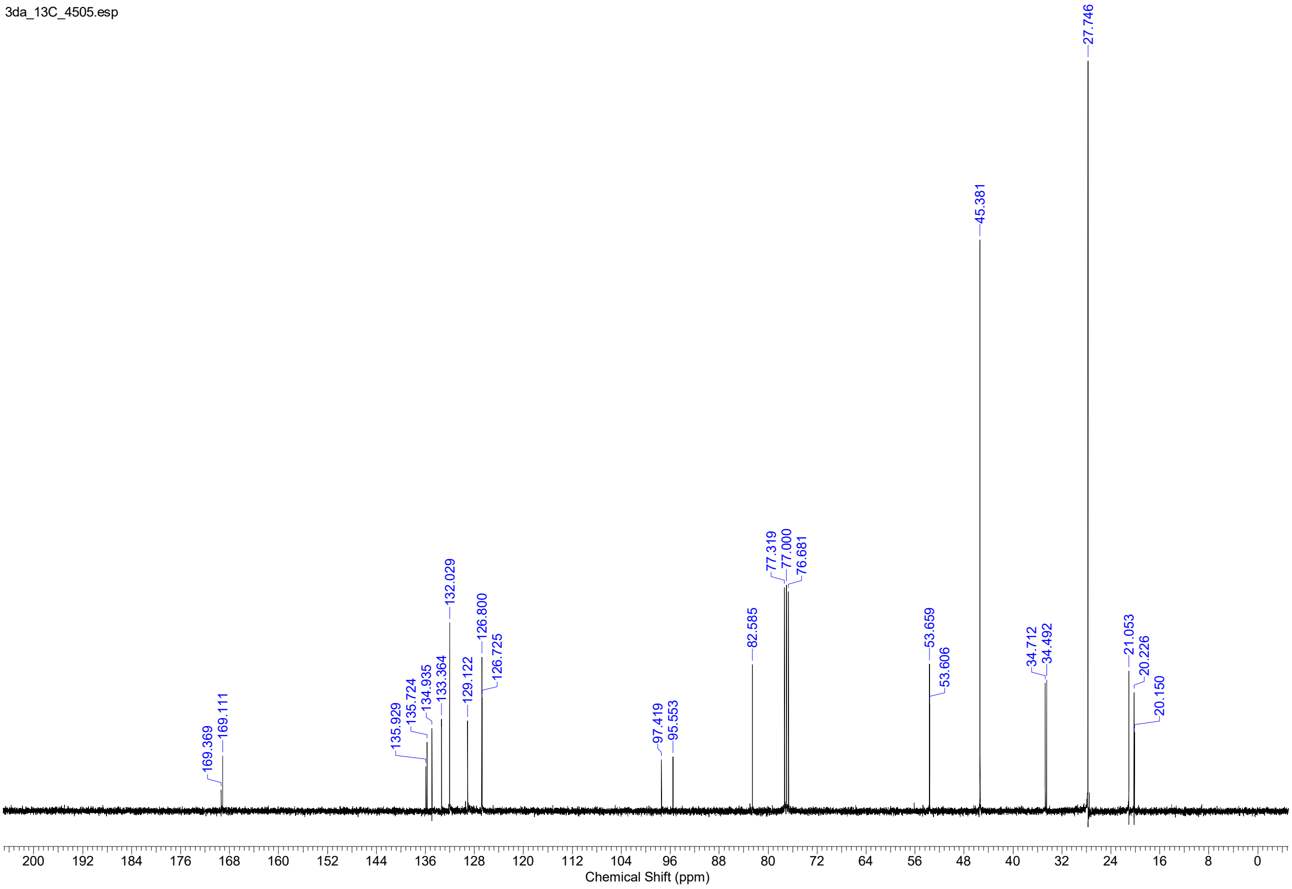

3ea_1H_4517.esp

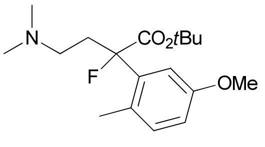

3ea

^{1}H (400 MHz, $CDCl_3$)

^{13}C (101 MHz, $CDCl_3$)

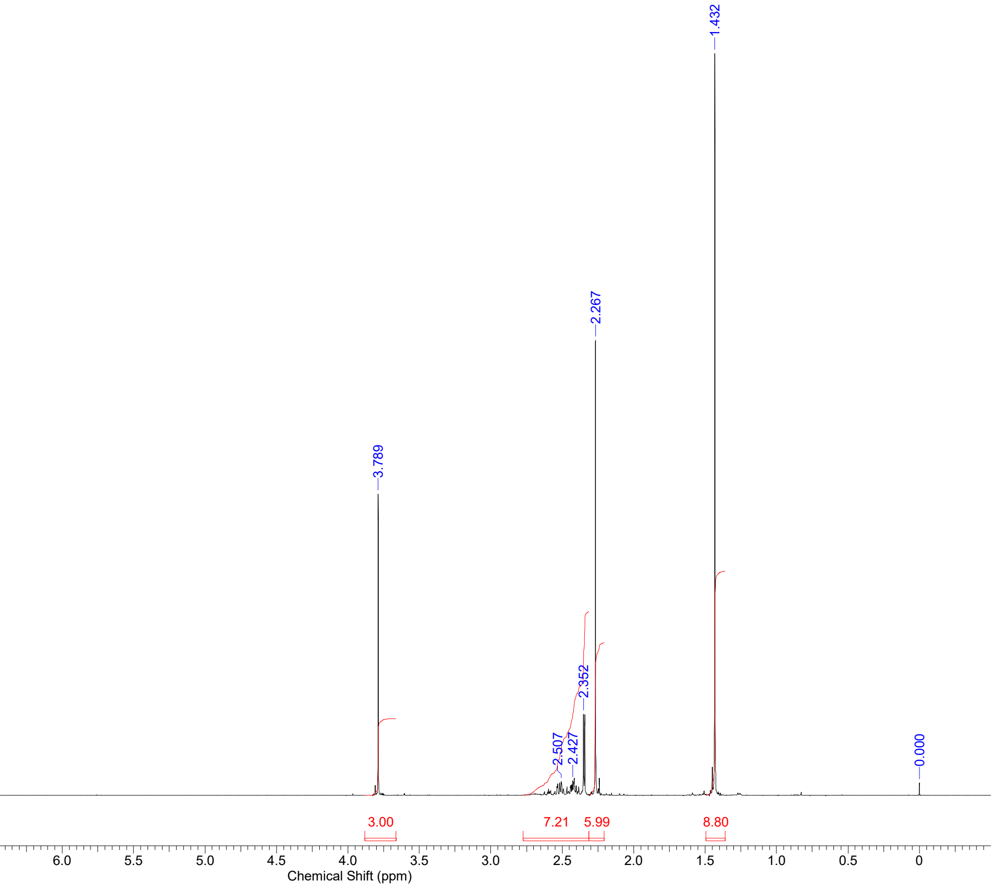

3ea_13C_4518.esp

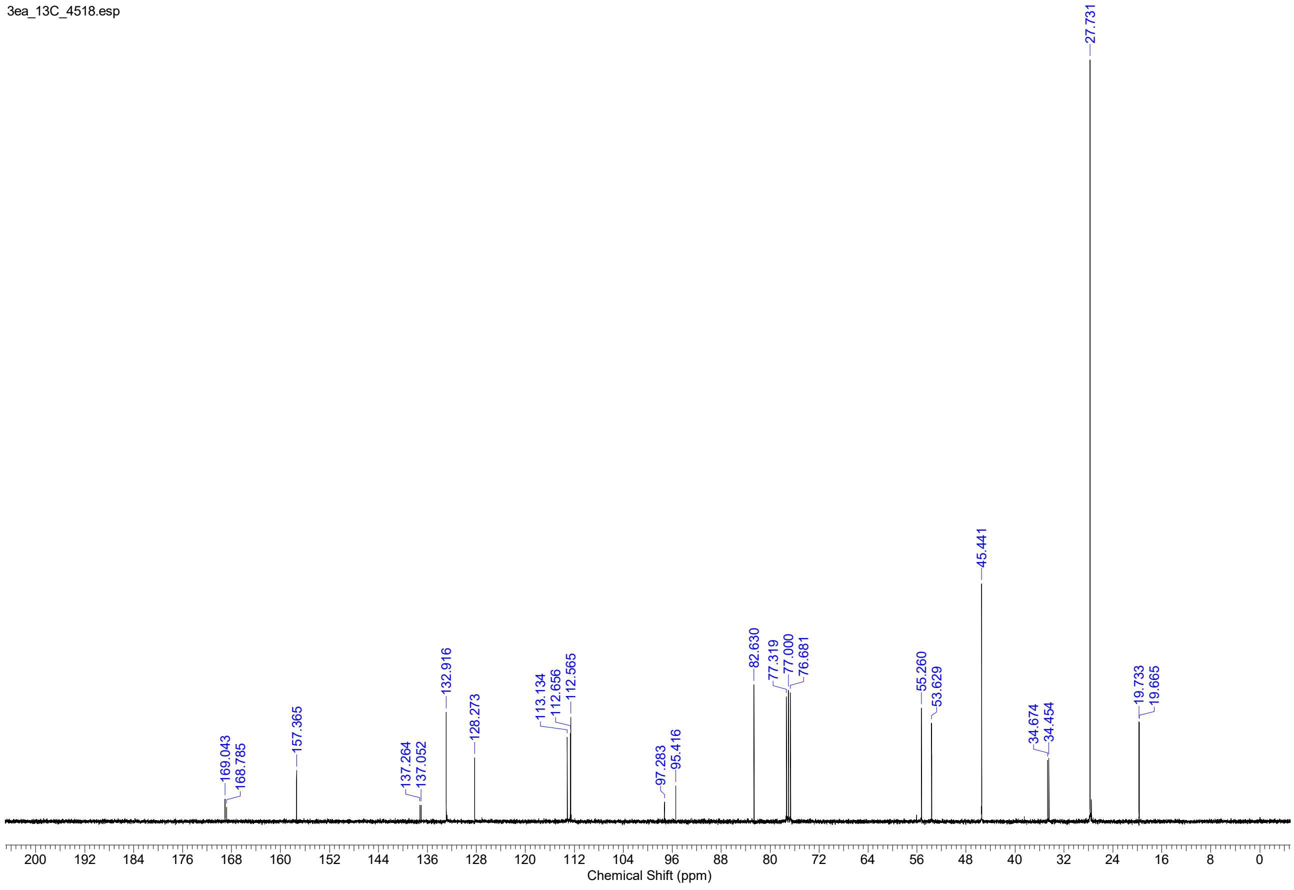

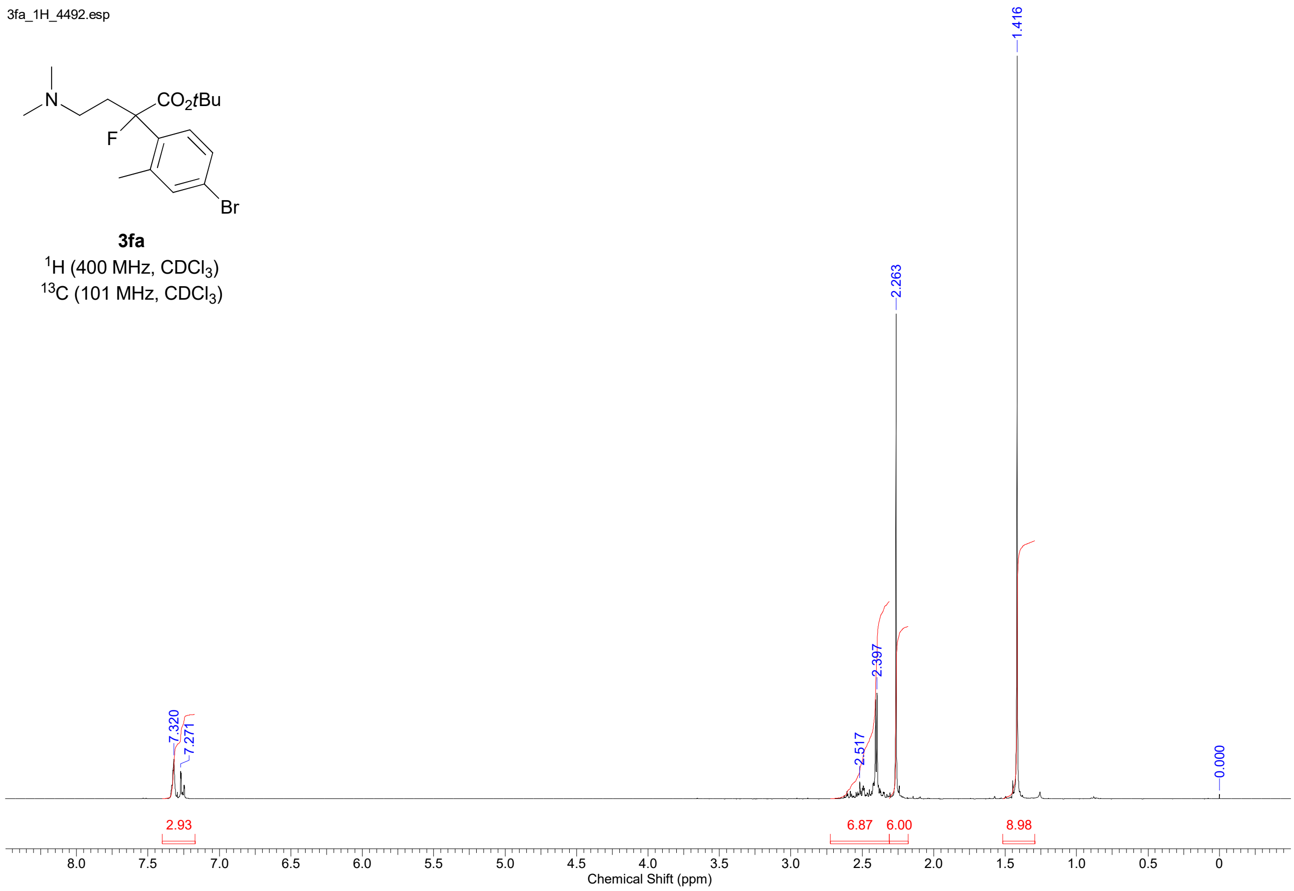
3fa_1H_4492.esp
CO2tBu
N
F
Br
3fa
1H (400 MHz, CDCl3)
13C (101 MHz, CDCl3)
1.416
2.263
2.397
2.517
7.320
7.271
0.000
2.93
6.87
6.00
8.98
Chemical Shift (ppm)

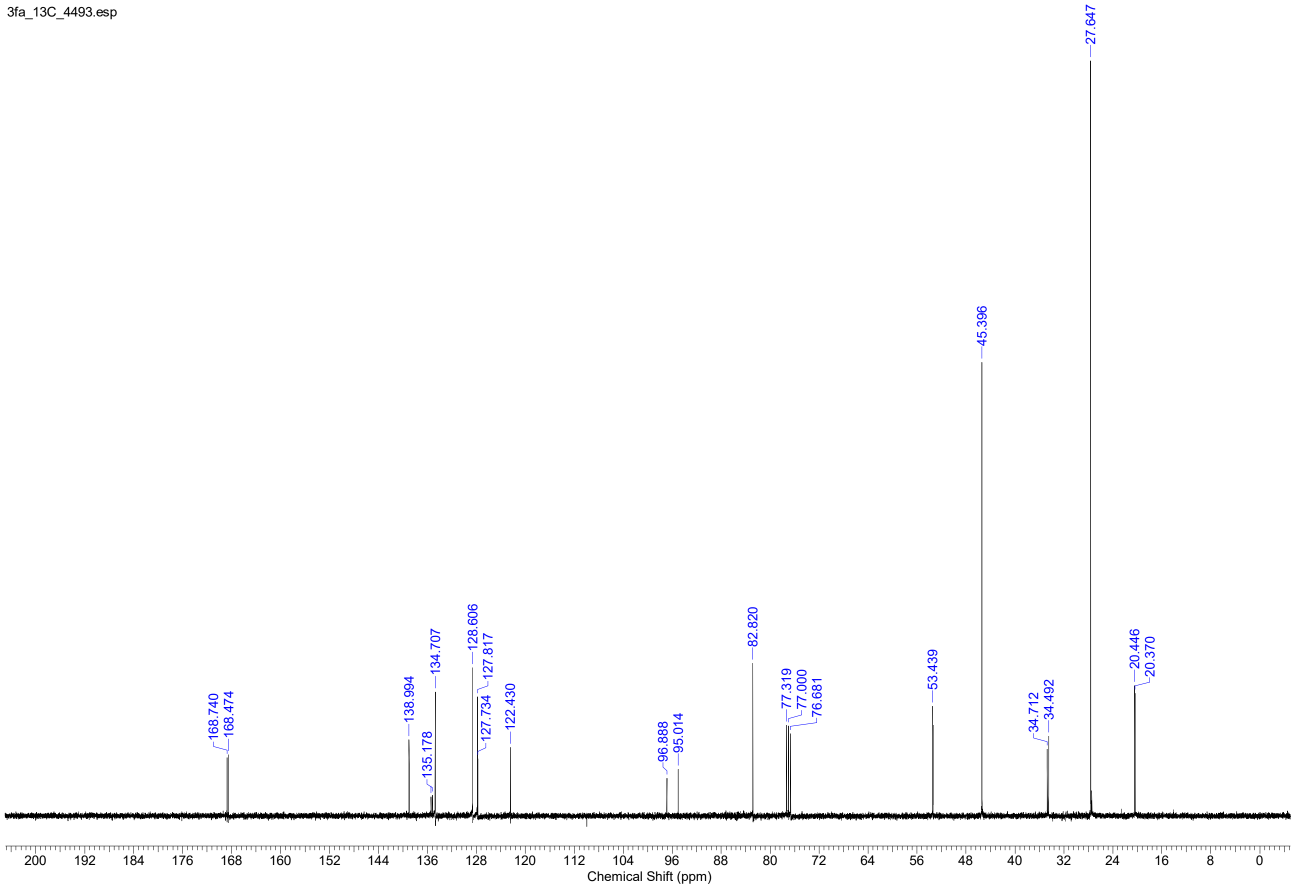
3fa_13C_4493.esp
27.647
45.396
168.740
168.474
138.994
135.178
134.707
128.606
127.817
127.734
122.430
96.888
95.014
82.820
77.319
77.000
76.681
53.439
34.712
34.492
20.446
20.370
Chemical Shift (ppm)

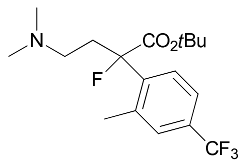

3ga

^{1}H (400 MHz, $CDCl_3$)

^{13}C (101 MHz, $CDCl_3$)

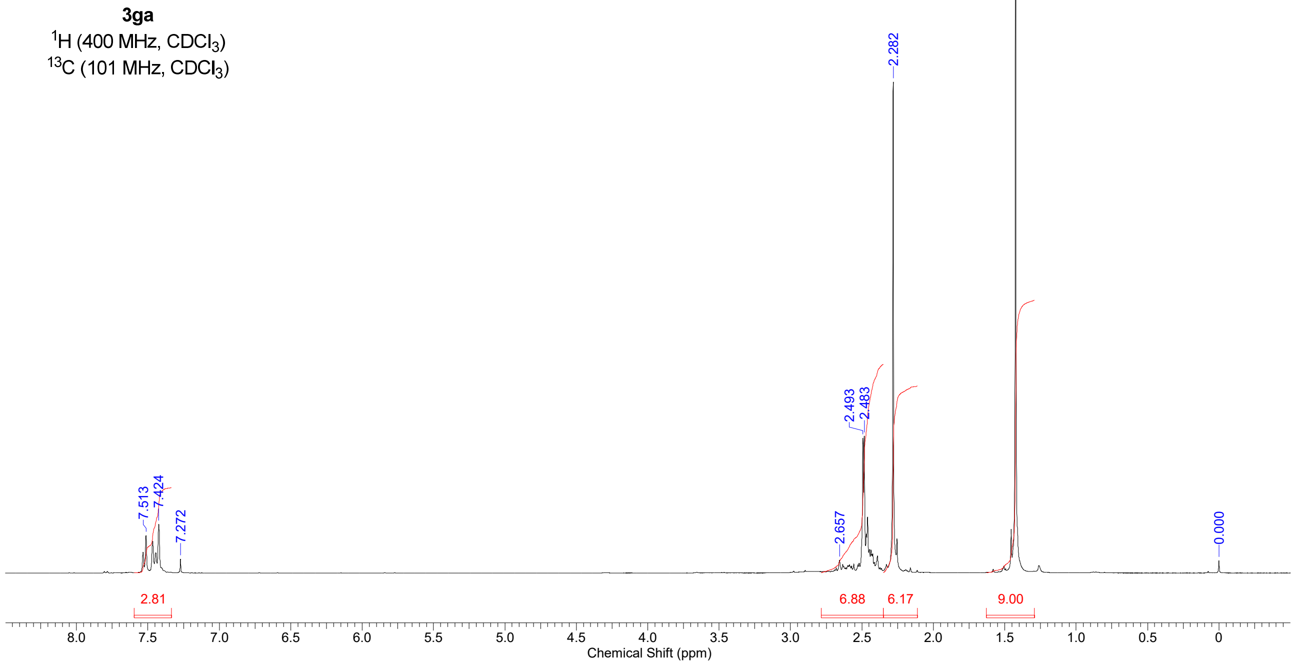

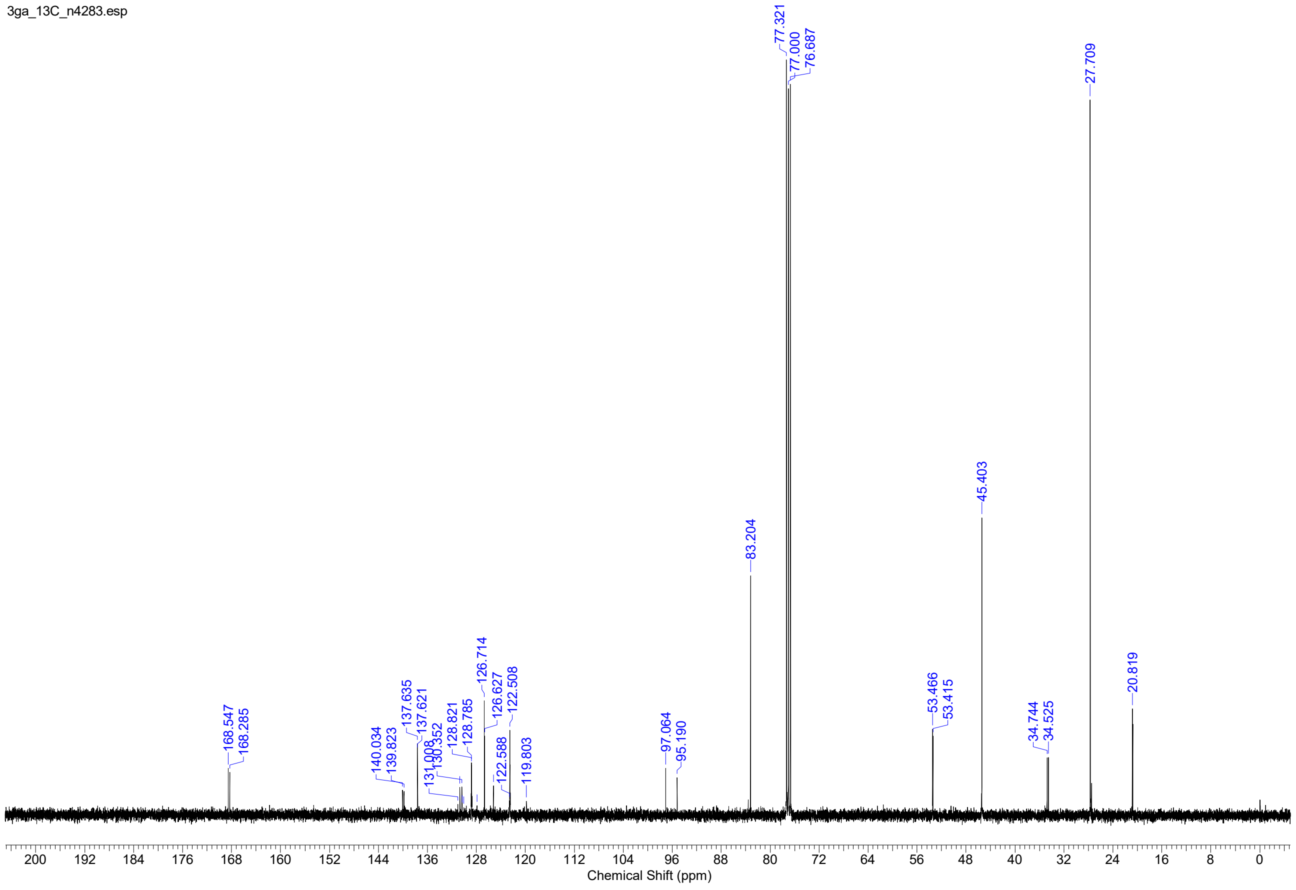

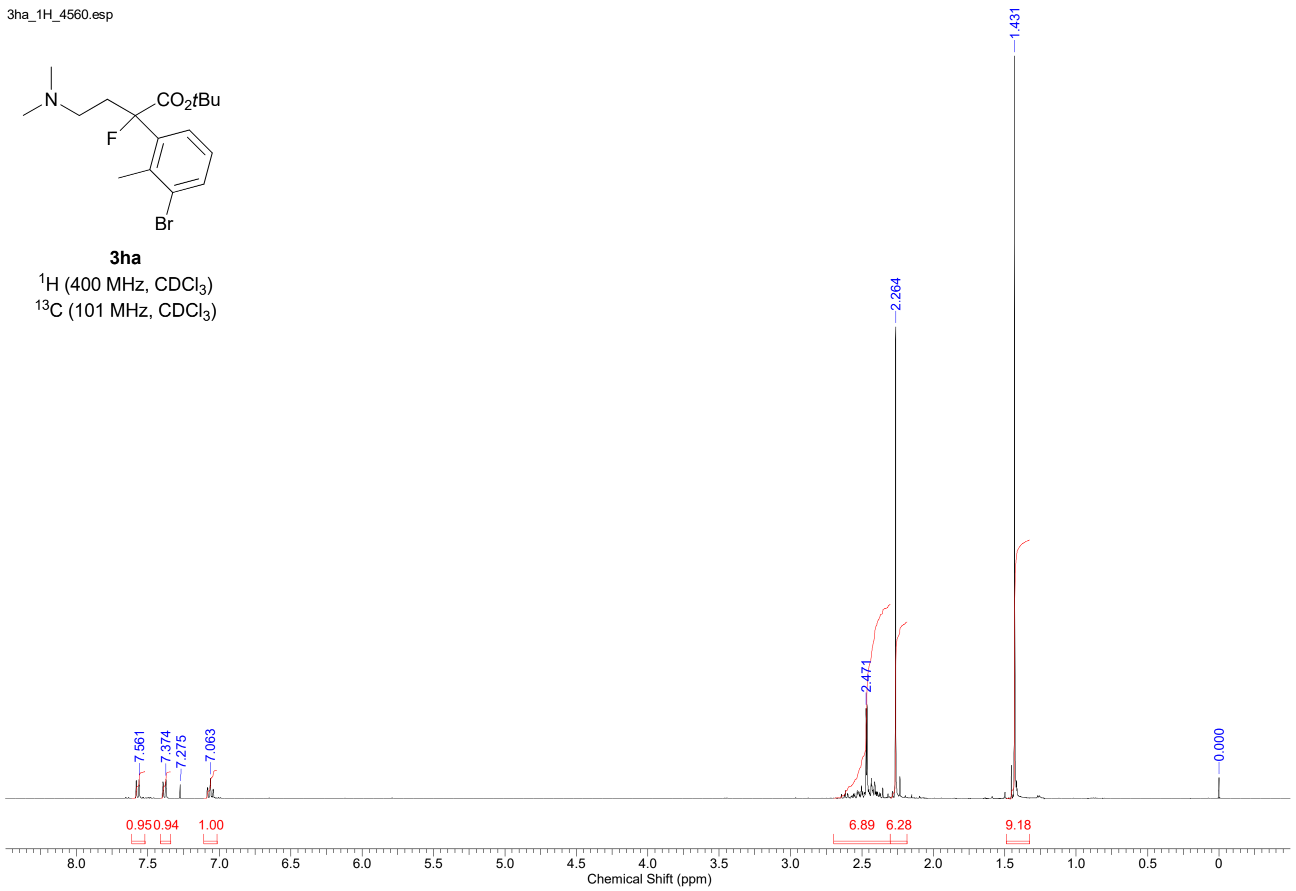
3ha_1H_4560.esp
N
CO2tBu
F
Br
3ha
1H (400 MHz, CDCl3)
13C (101 MHz, CDCl3)
1.431
2.264
2.471
7.561
7.374
7.275
7.063
0.000
0.95
0.94
1.00
6.89
6.28
9.18
Chemical Shift (ppm)

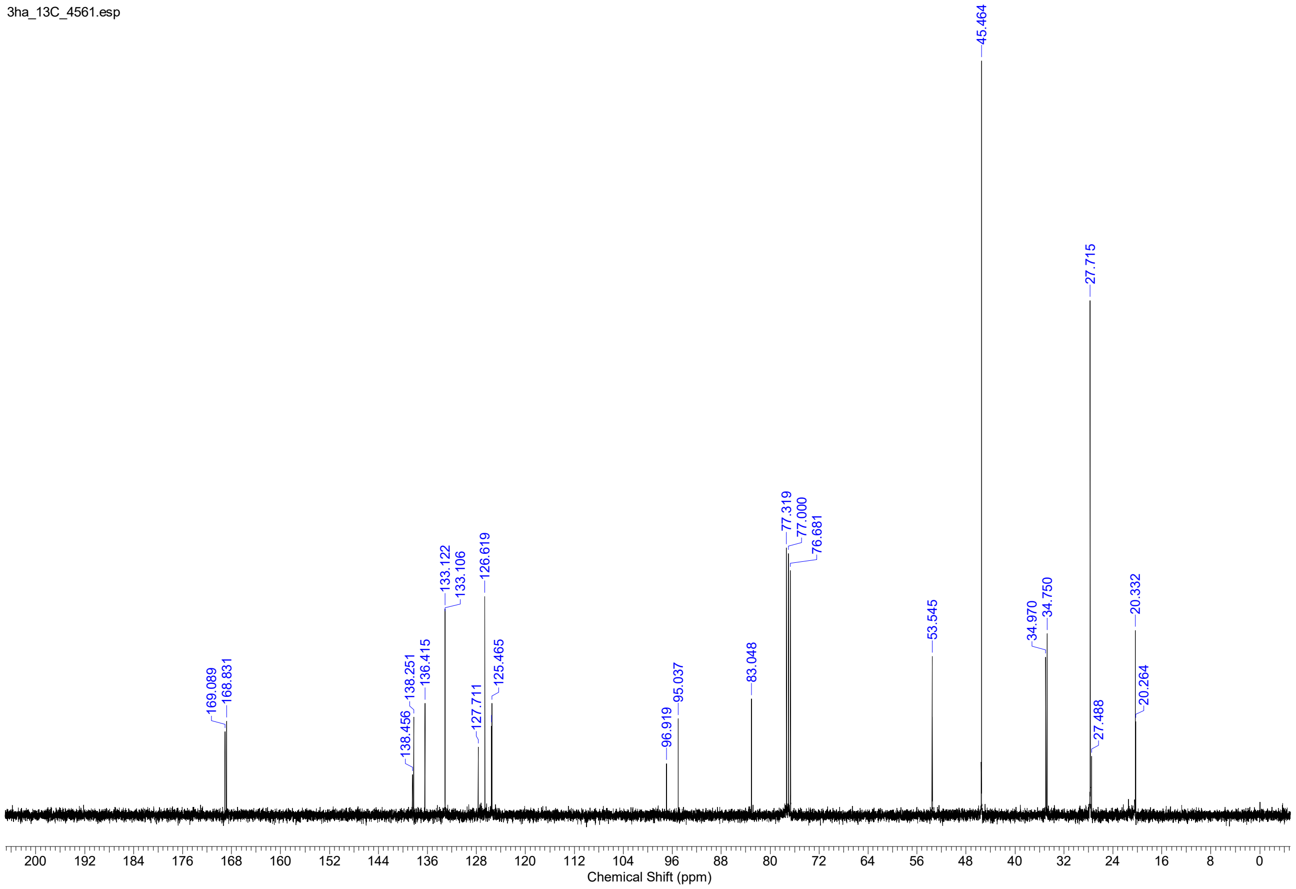
3ha_13C_4561.esp
45.464
27.715
77.319
77.000
76.681
126.619
133.122
133.106
136.415
138.251
138.456
127.711
125.465
169.089
168.831
96.919
95.037
83.048
53.545
34.970
34.750
20.332
20.264
27.488
Chemical Shift (ppm)

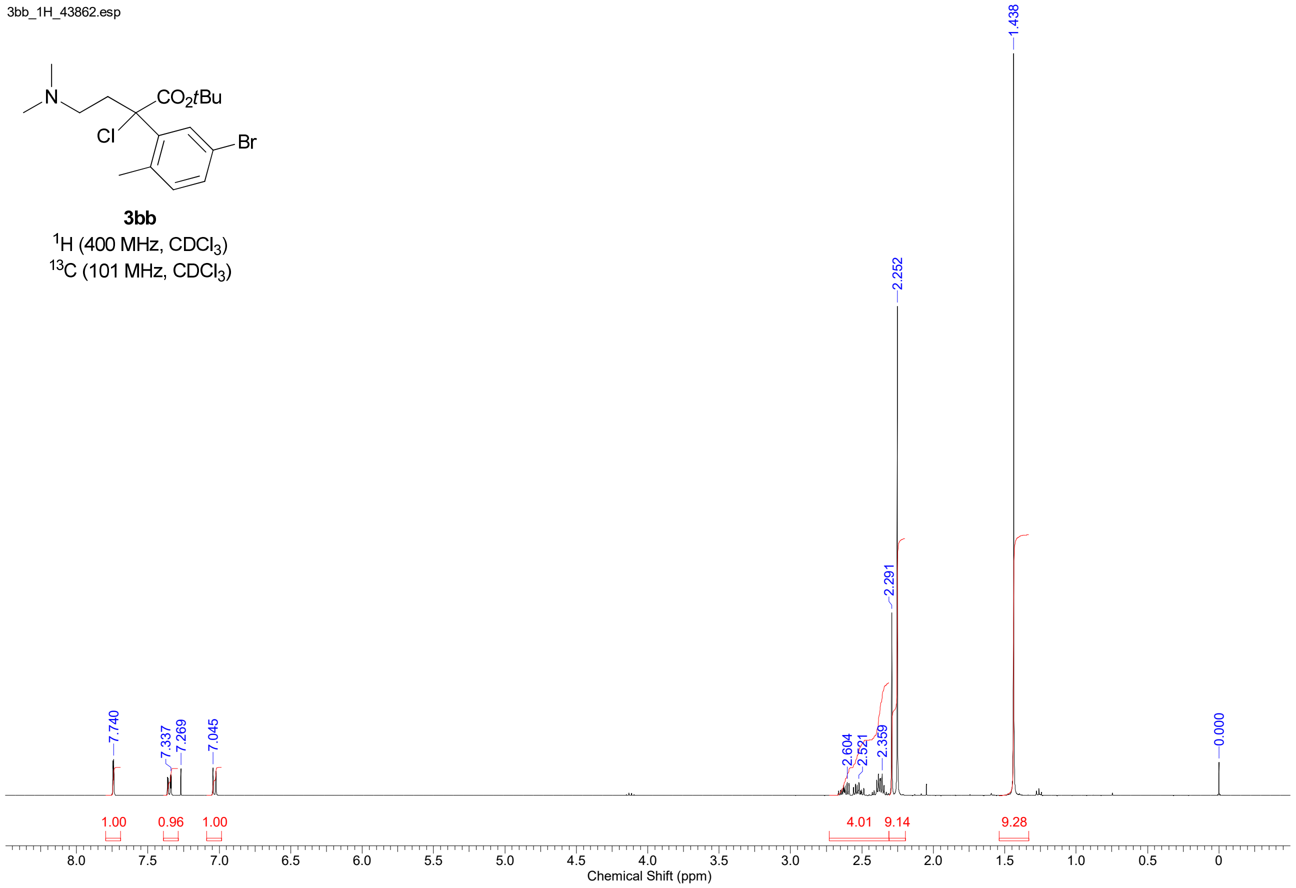

3bb

^{1}H (400 MHz, $CDCl_3$)

^{13}C (101 MHz, $CDCl_3$)

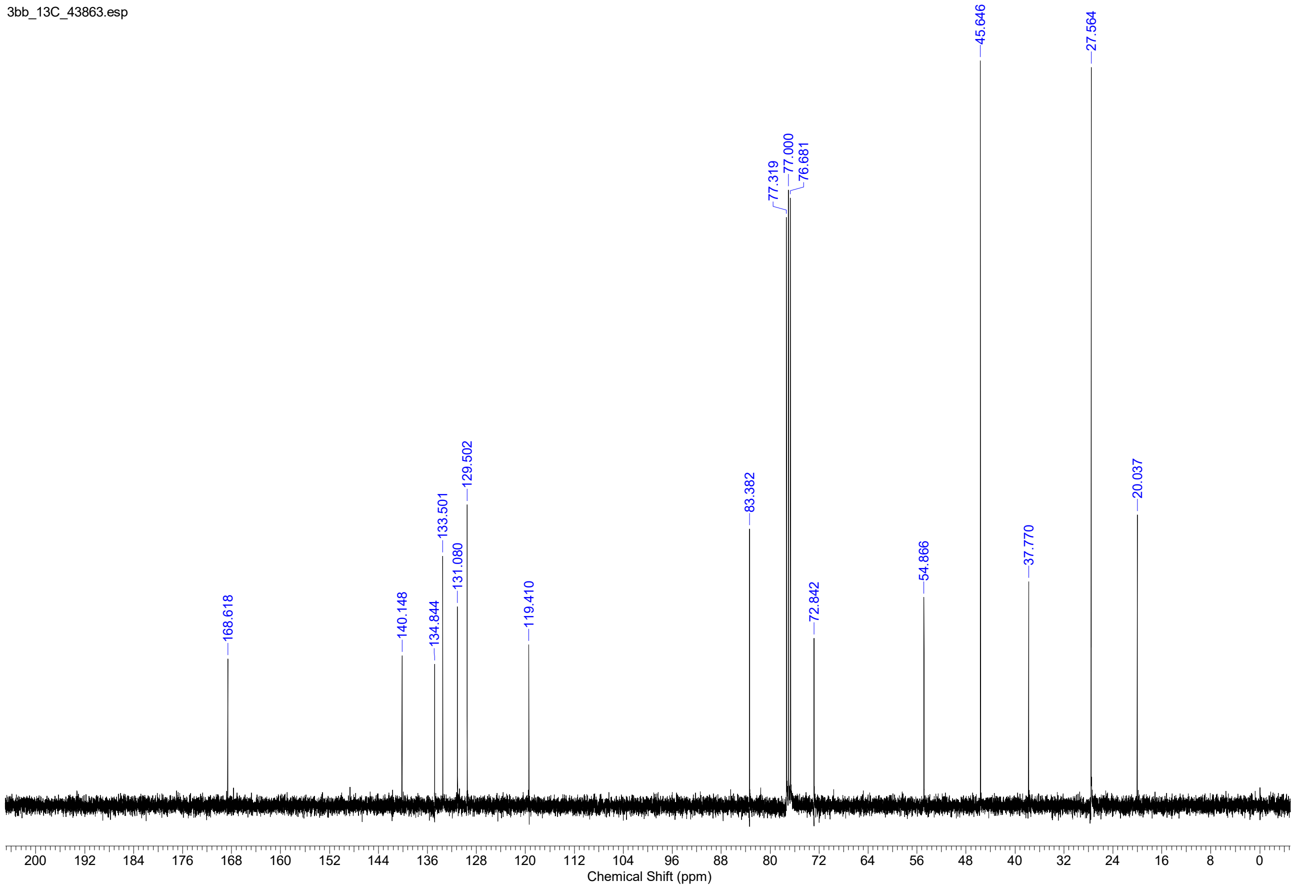

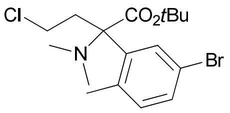

4bb

^{1}H (400 MHz, $CDCl_3$)

^{13}C (101 MHz, $CDCl_3$)

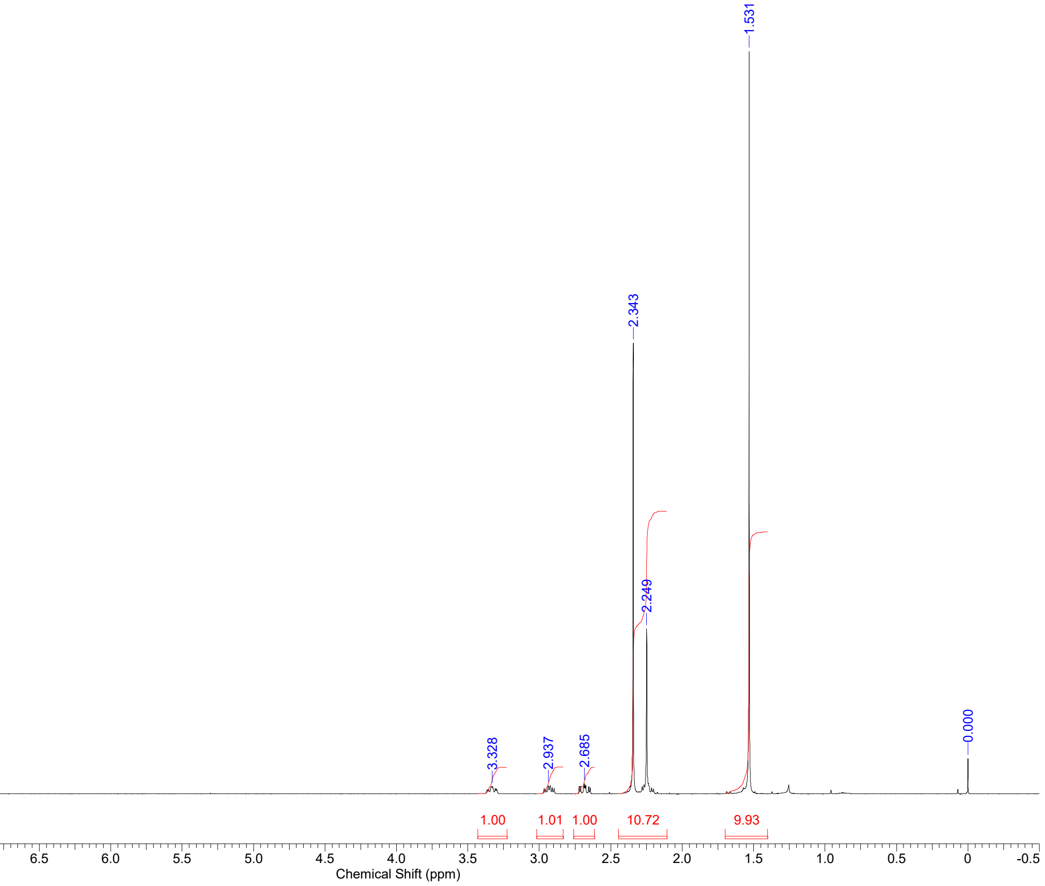

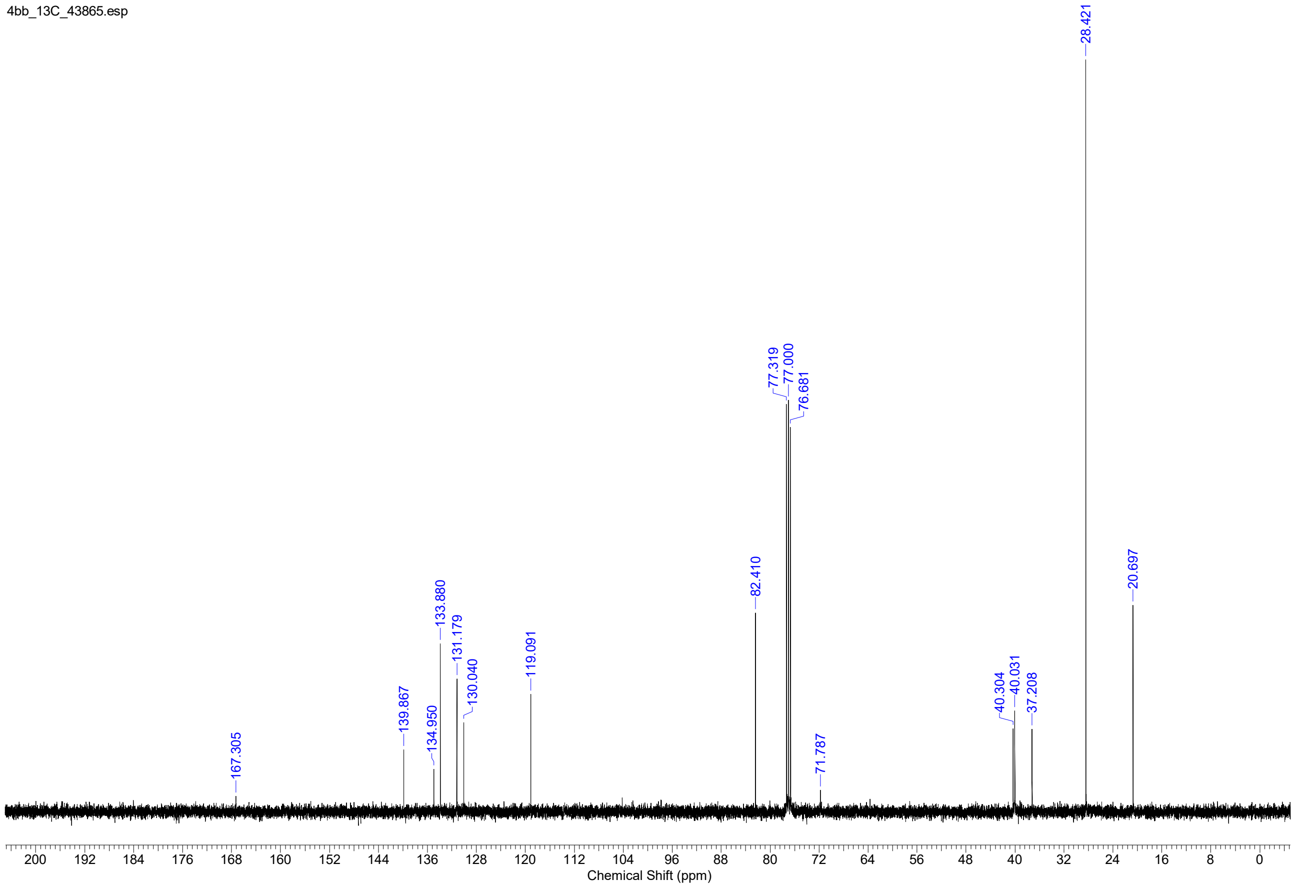

3db_1H_4524.esp

3db

^{1}H (400 MHz, $CDCl_3$)

^{13}C (101 MHz, $CDCl_3$)

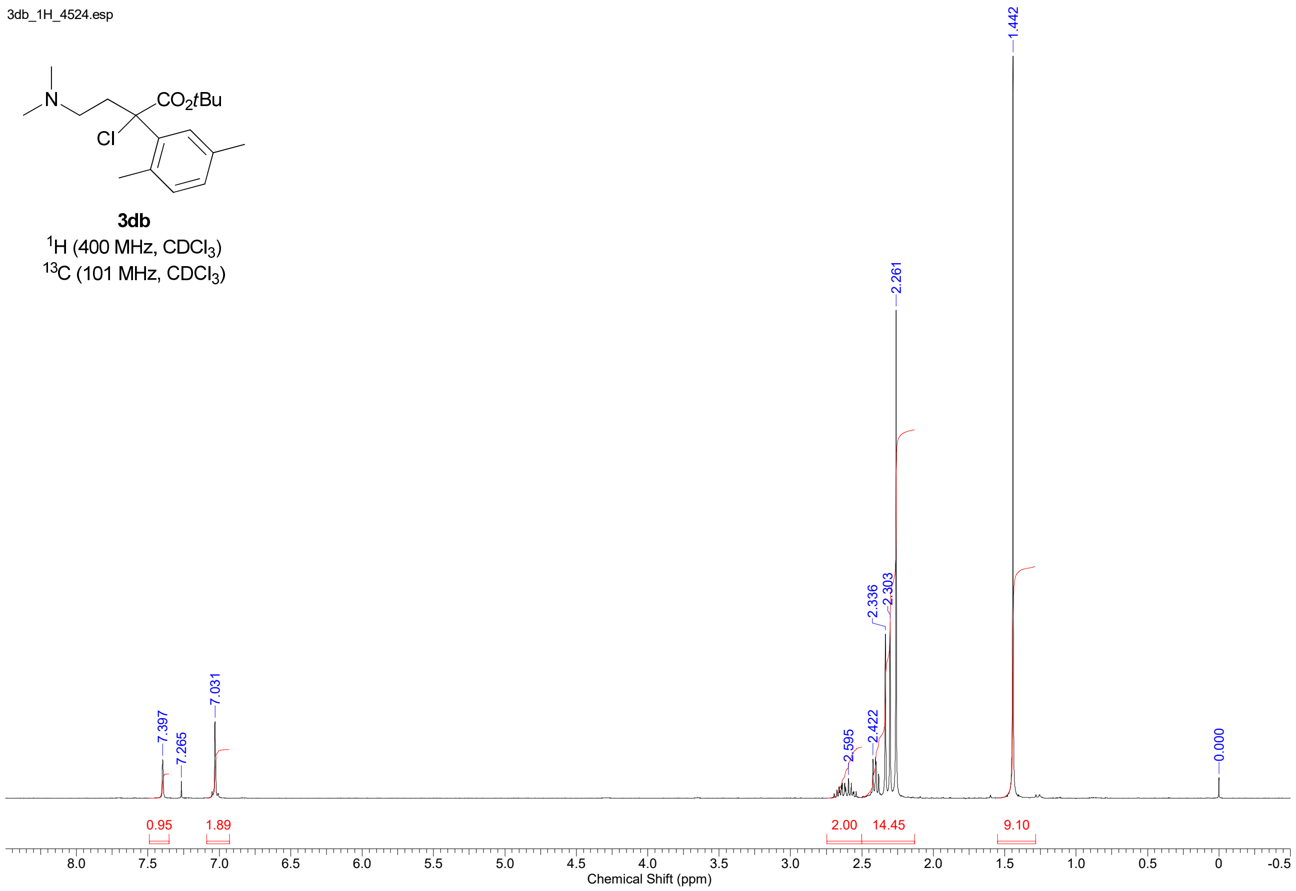

3db_13C_4525.esp

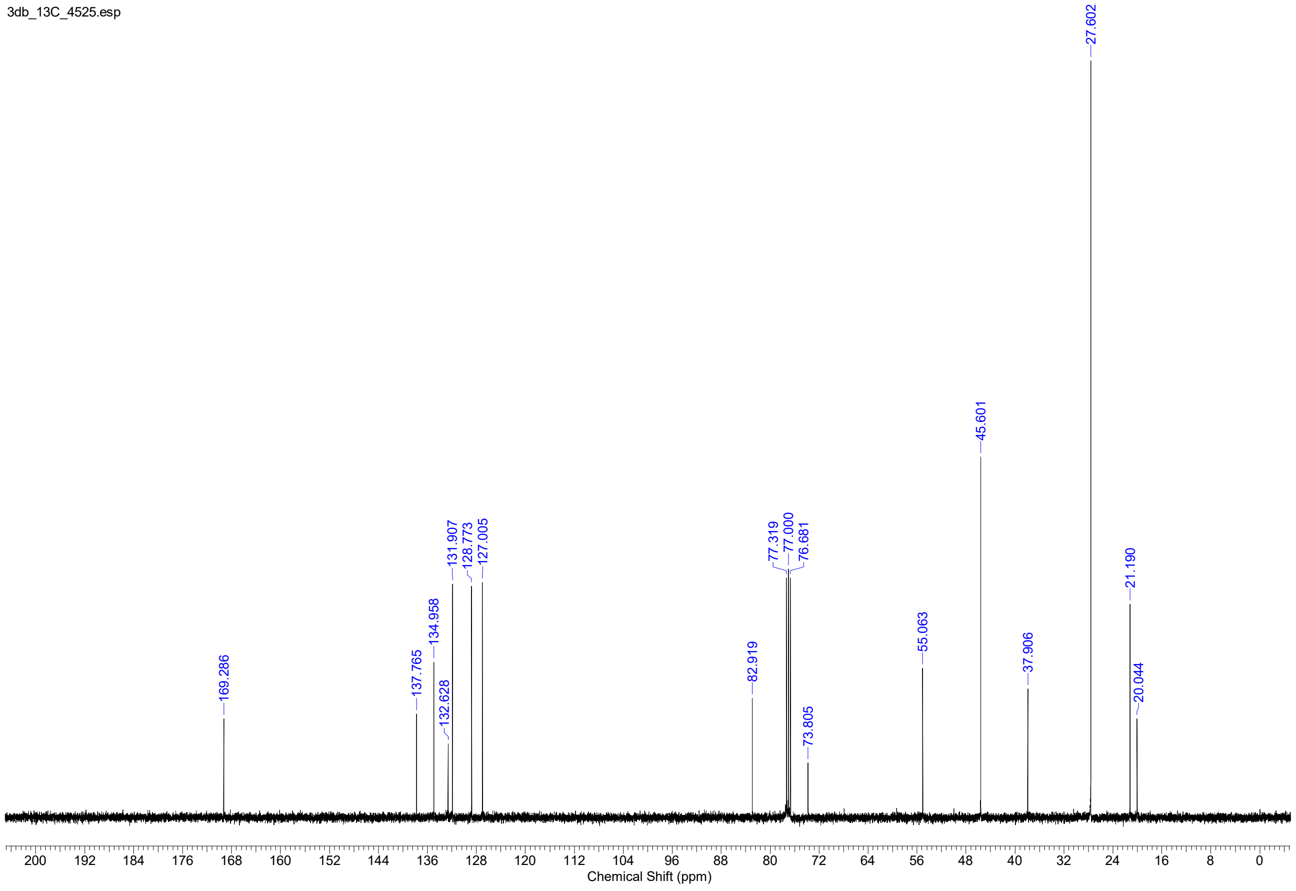

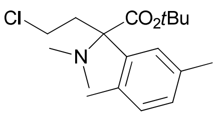

4db

^{1}H (400 MHz, $CDCl_3$)

^{13}C (101 MHz, $CDCl_3$)

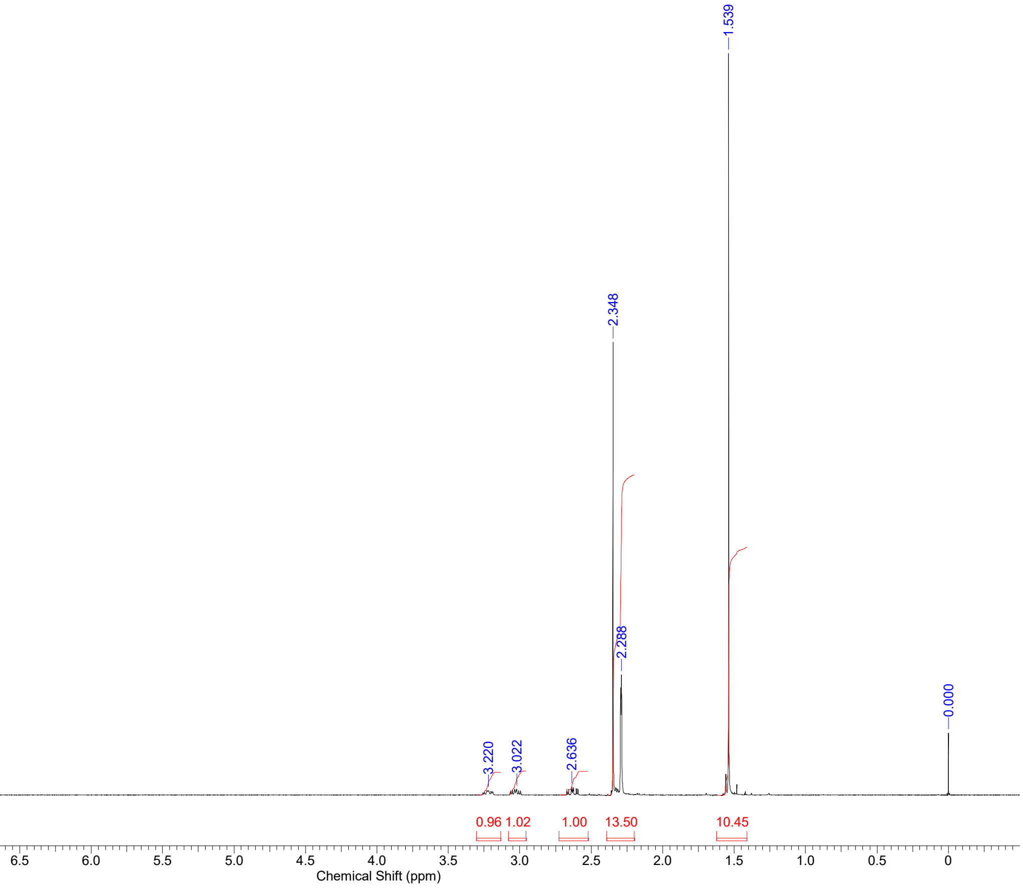

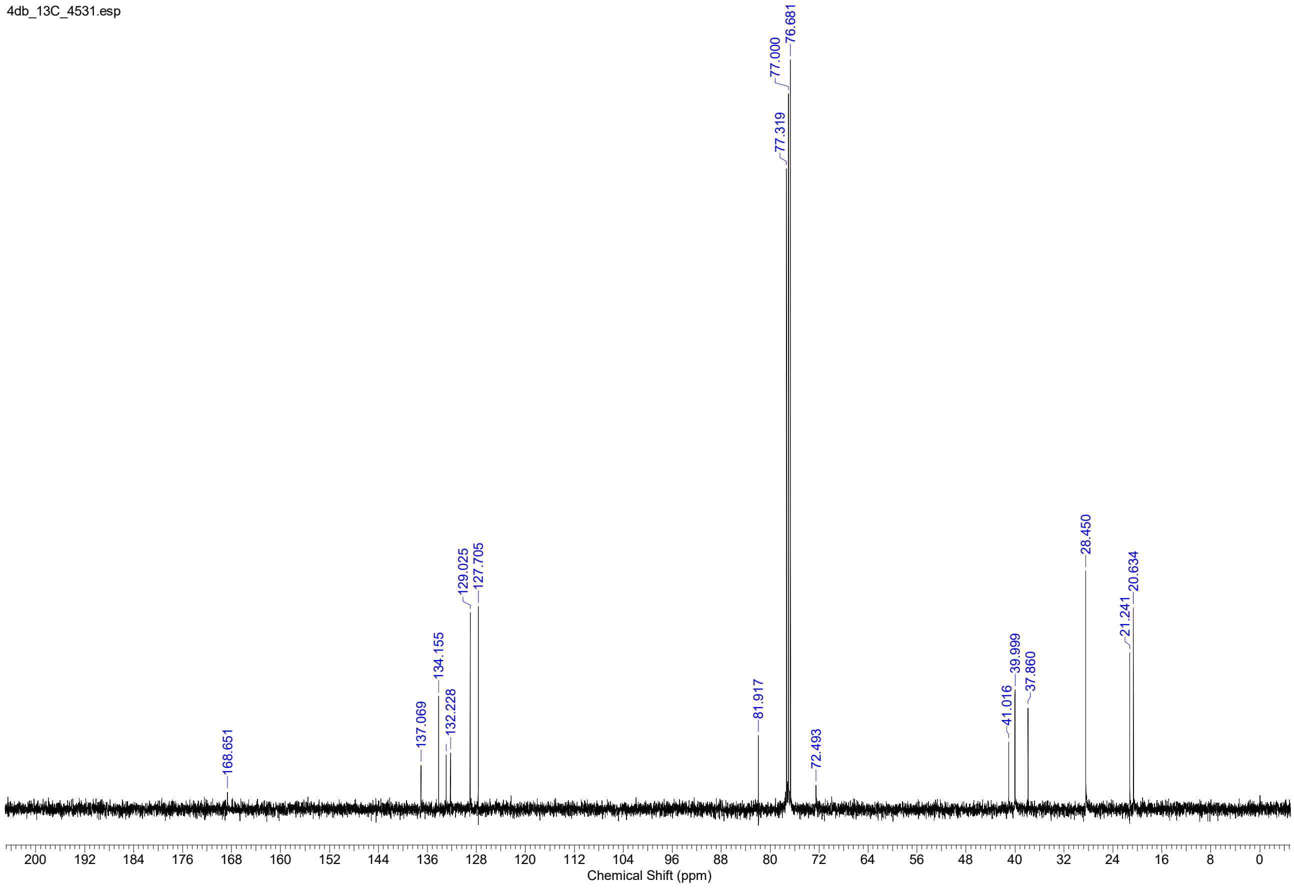

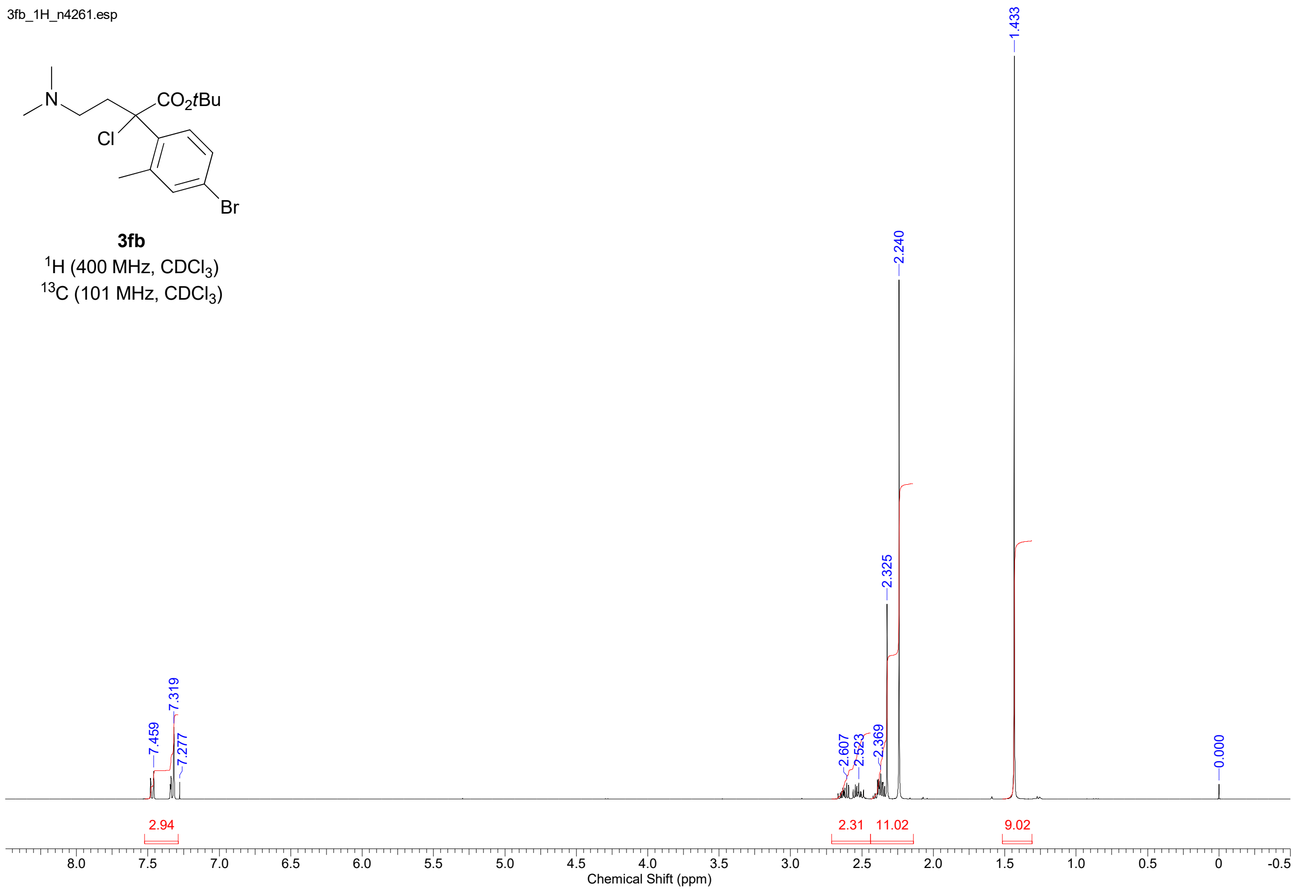

3fb

^{1}H (400 MHz, $CDCl_3$)

^{13}C (101 MHz, $CDCl_3$)

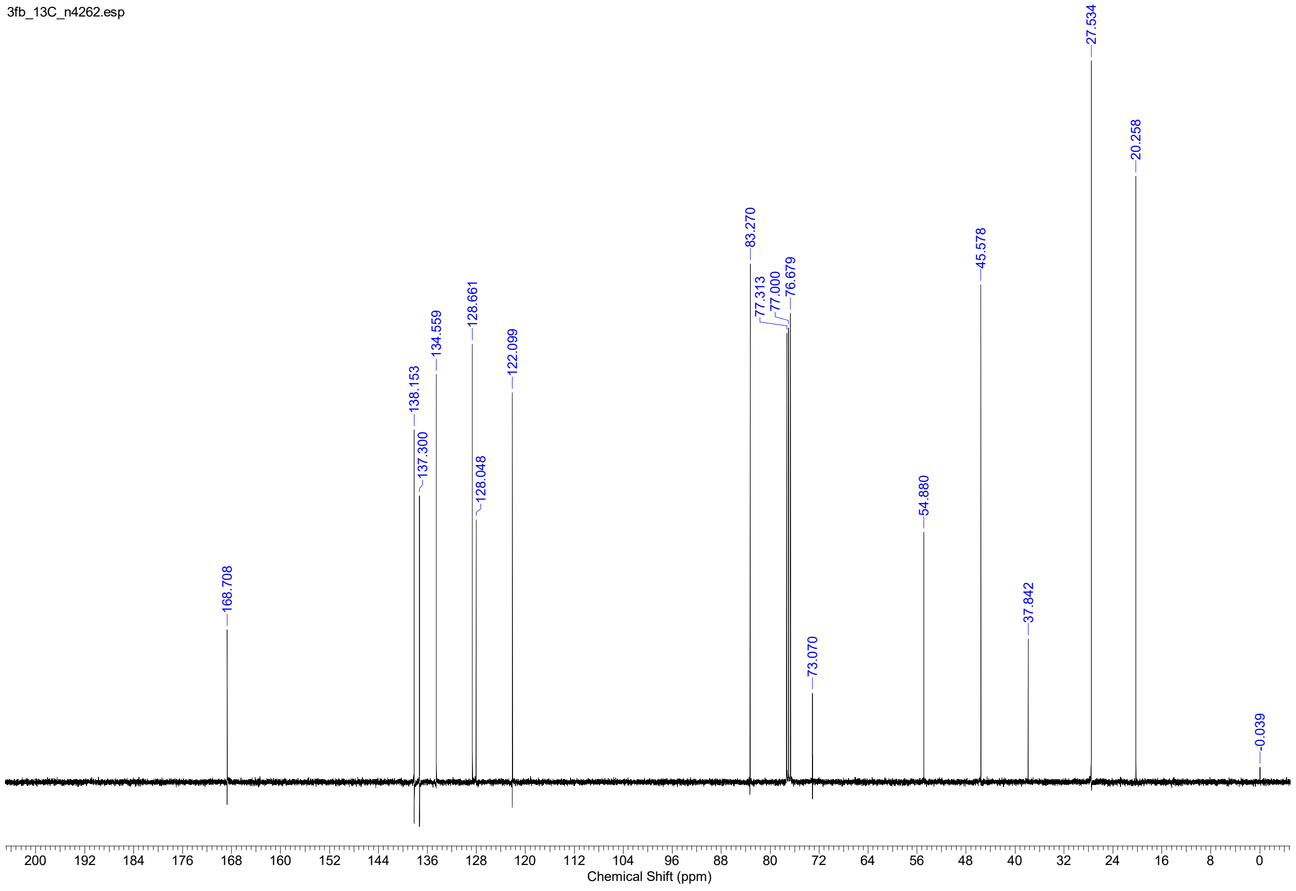

4fb_1H_n4263.esp

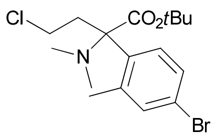

4fb

^{1}H (400 MHz, $CDCl_3$)

^{13}C (101 MHz, $CDCl_3$)

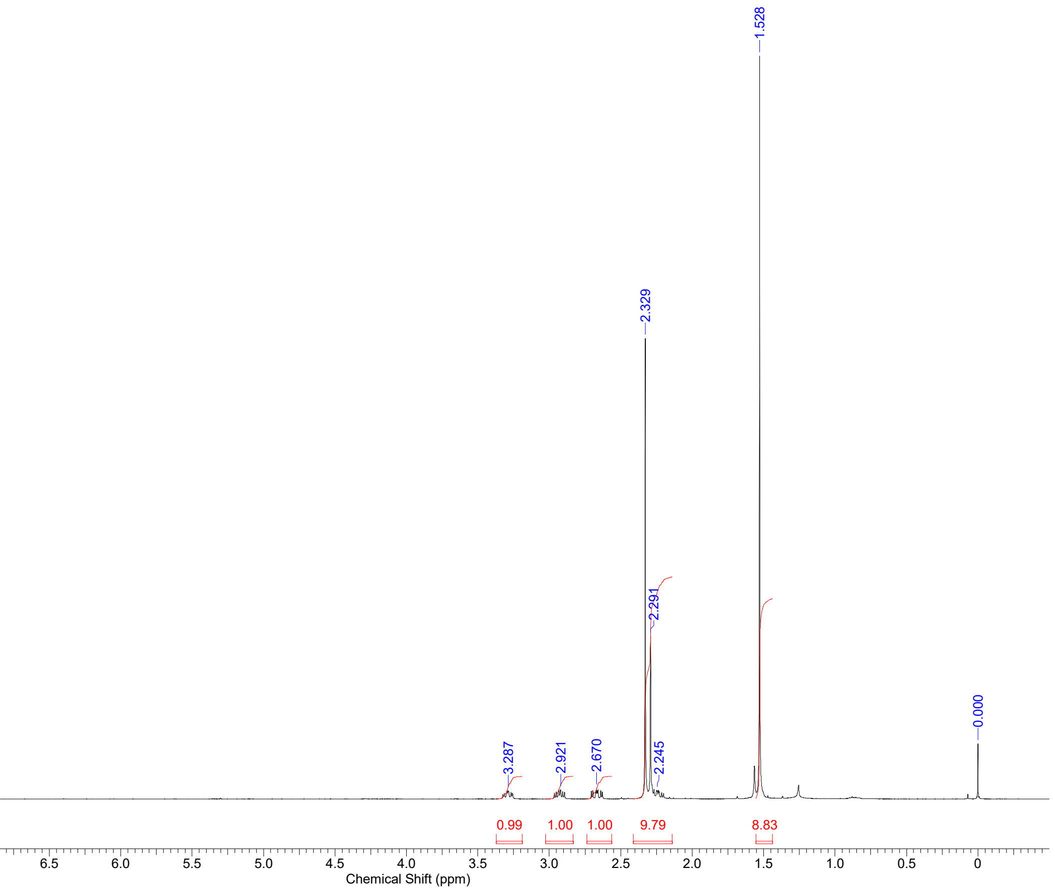

4fb_13C_n4264.esp

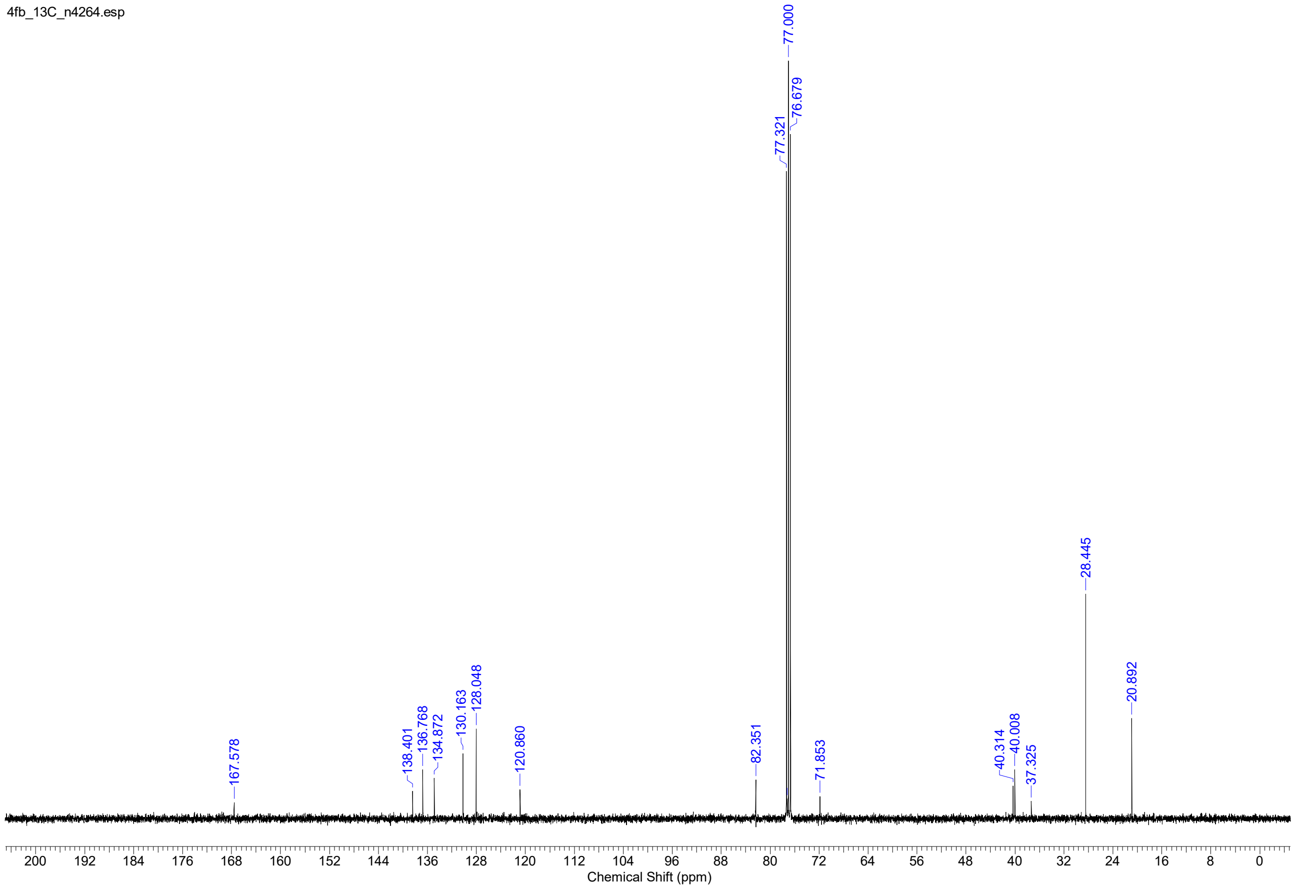

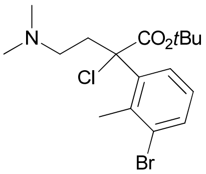

3hb

^{1}H (400 MHz, $CDCl_3$)

^{13}C (101 MHz, $CDCl_3$)

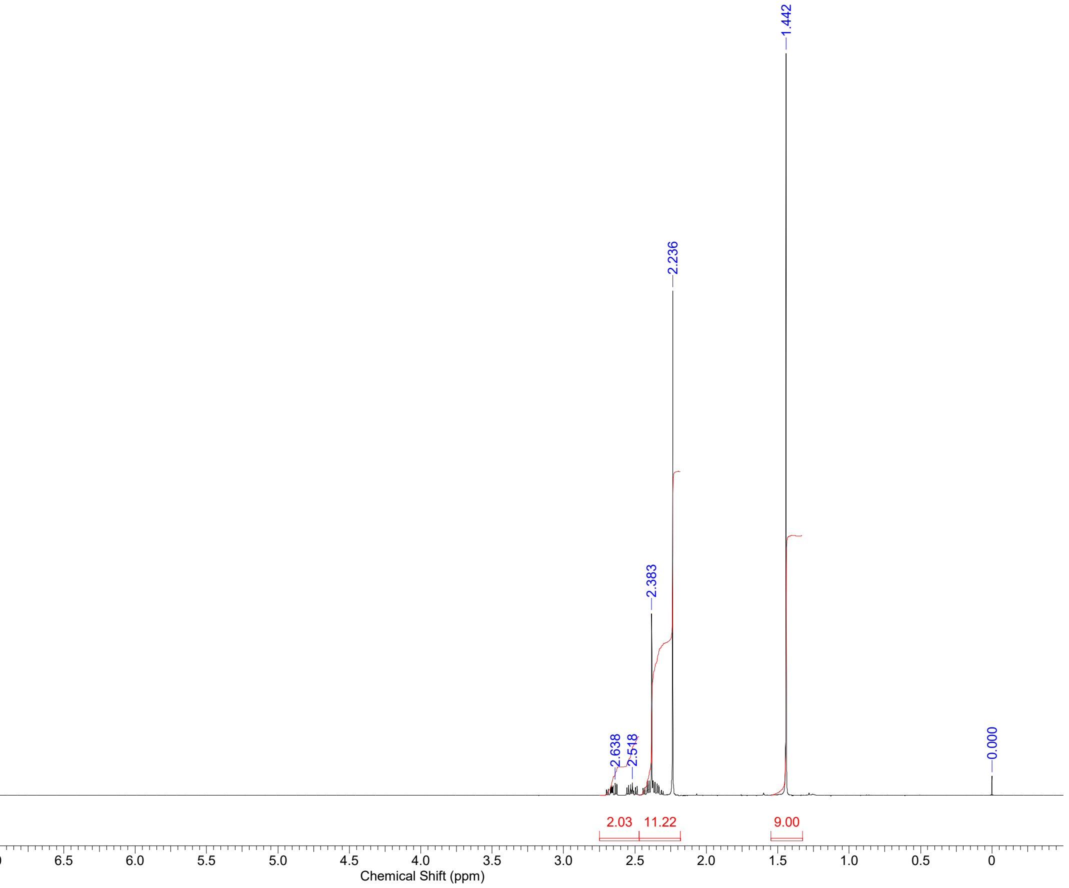

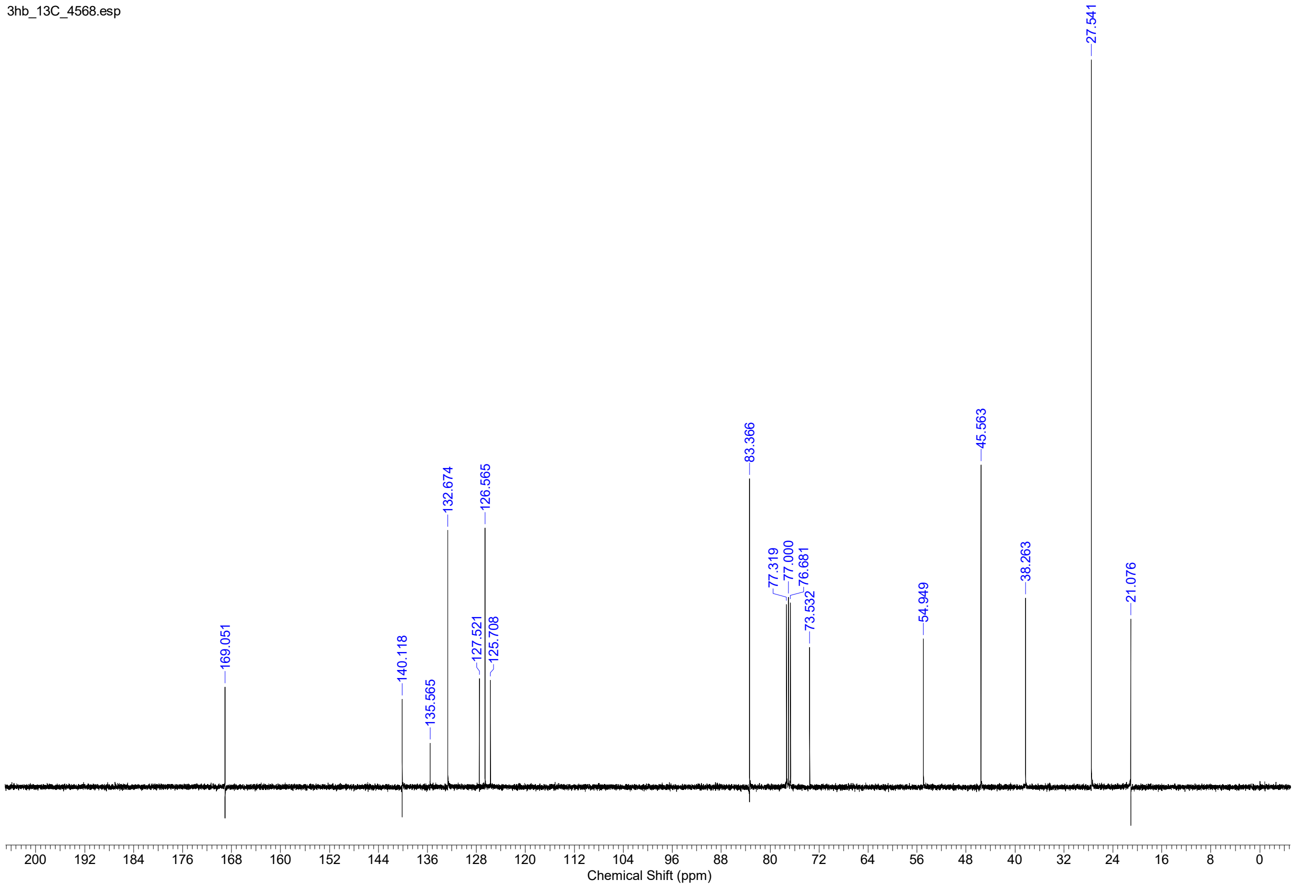

4hb_1H_4563.esp

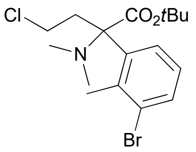

4hb

^{1}H (400 MHz, $CDCl_3$)

^{13}C (101 MHz, $CDCl_3$)

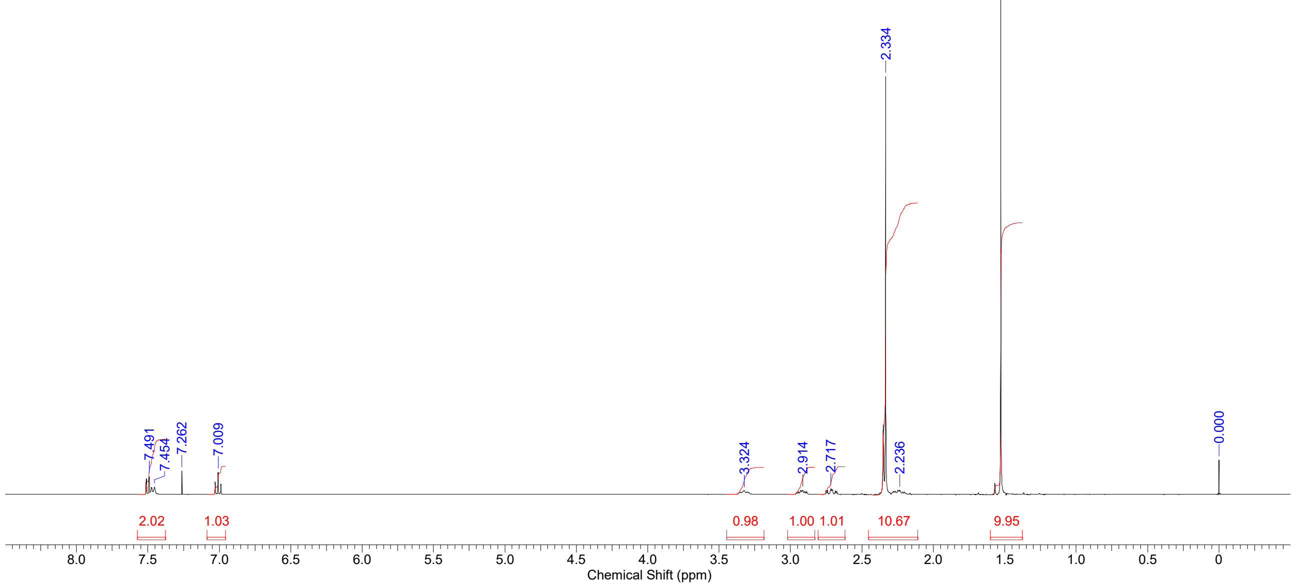

4hb_13C_4566.esp

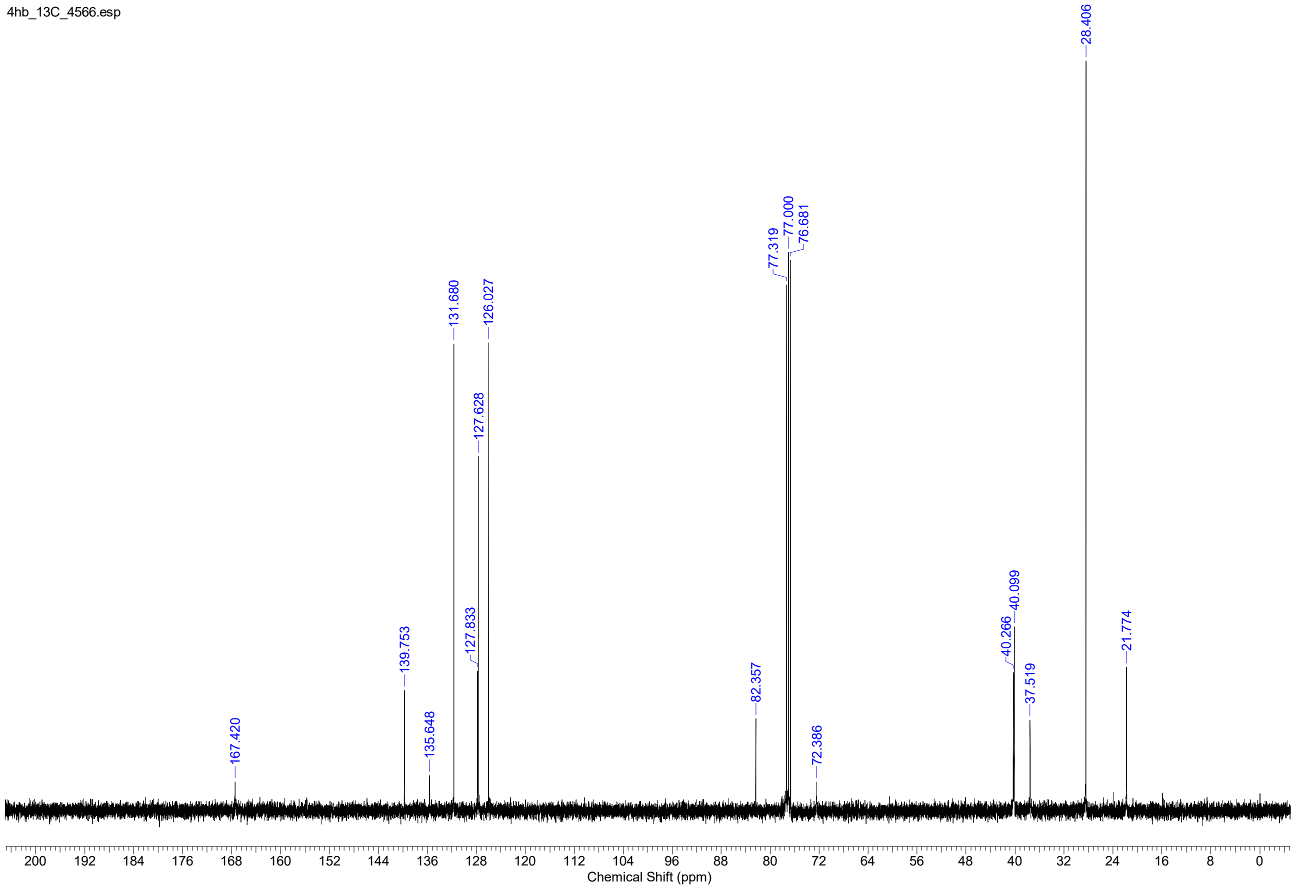

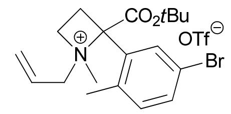

5

8/2 dr

^{1}H (400 MHz, acetone-d_6)

^{13}C (101 MHz, acetone-d_6)

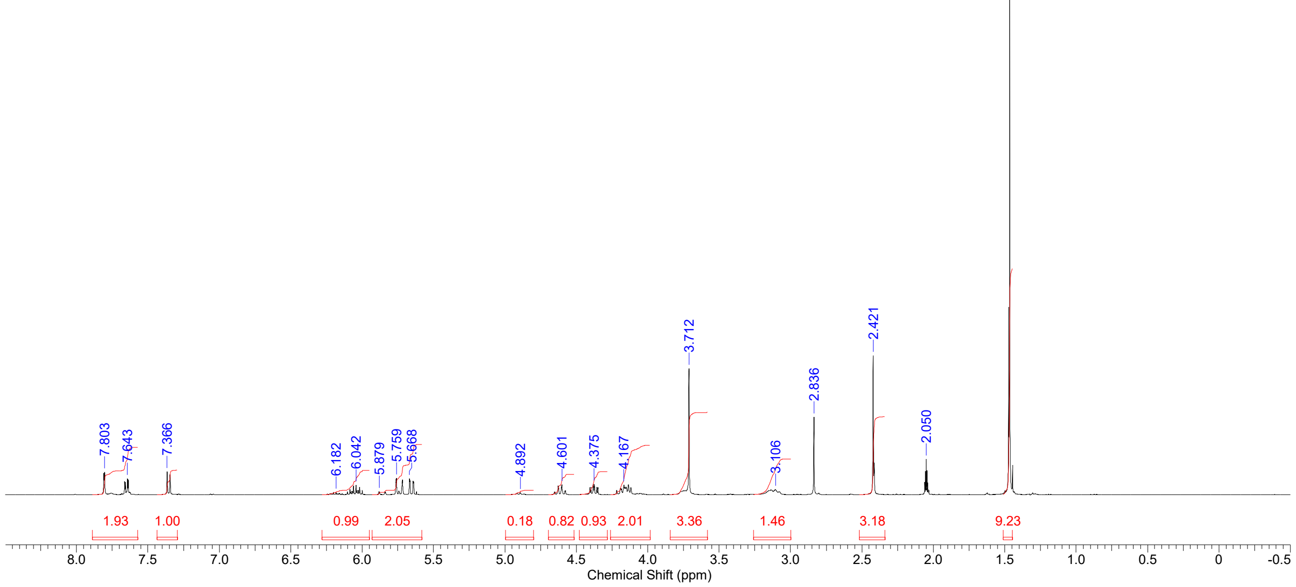

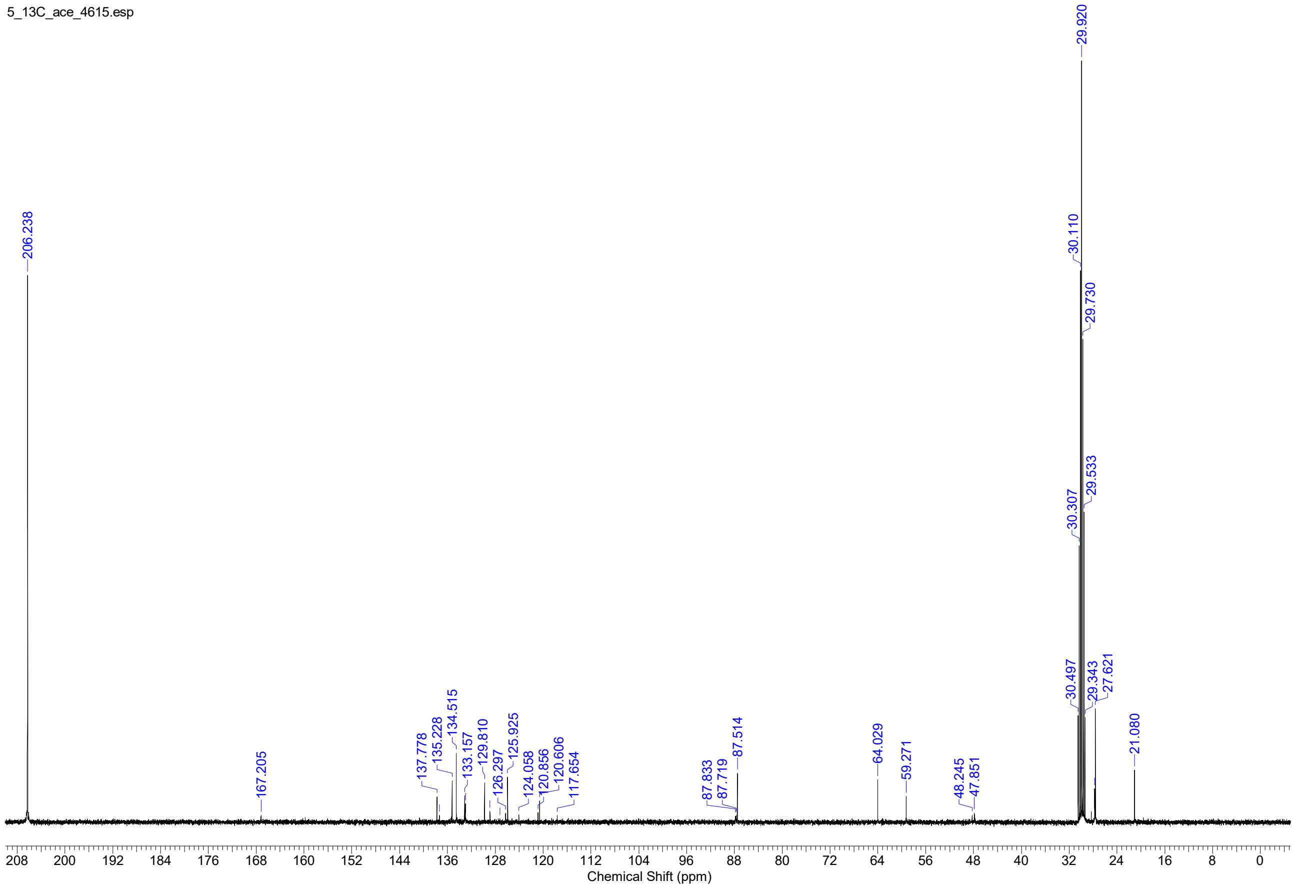

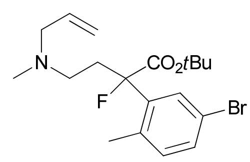

6

^{1}H (400 MHz, $CDCl_3$)
^{13}C (101 MHz, $CDCl_3$)

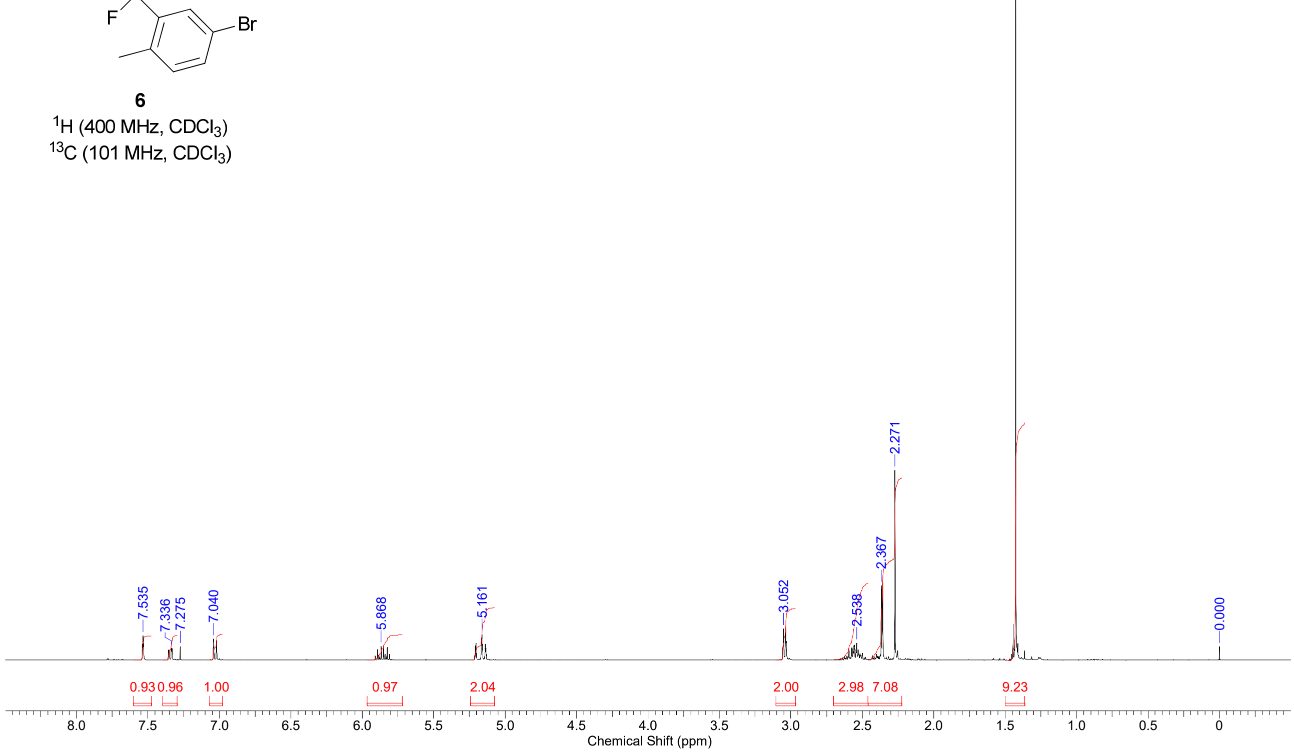

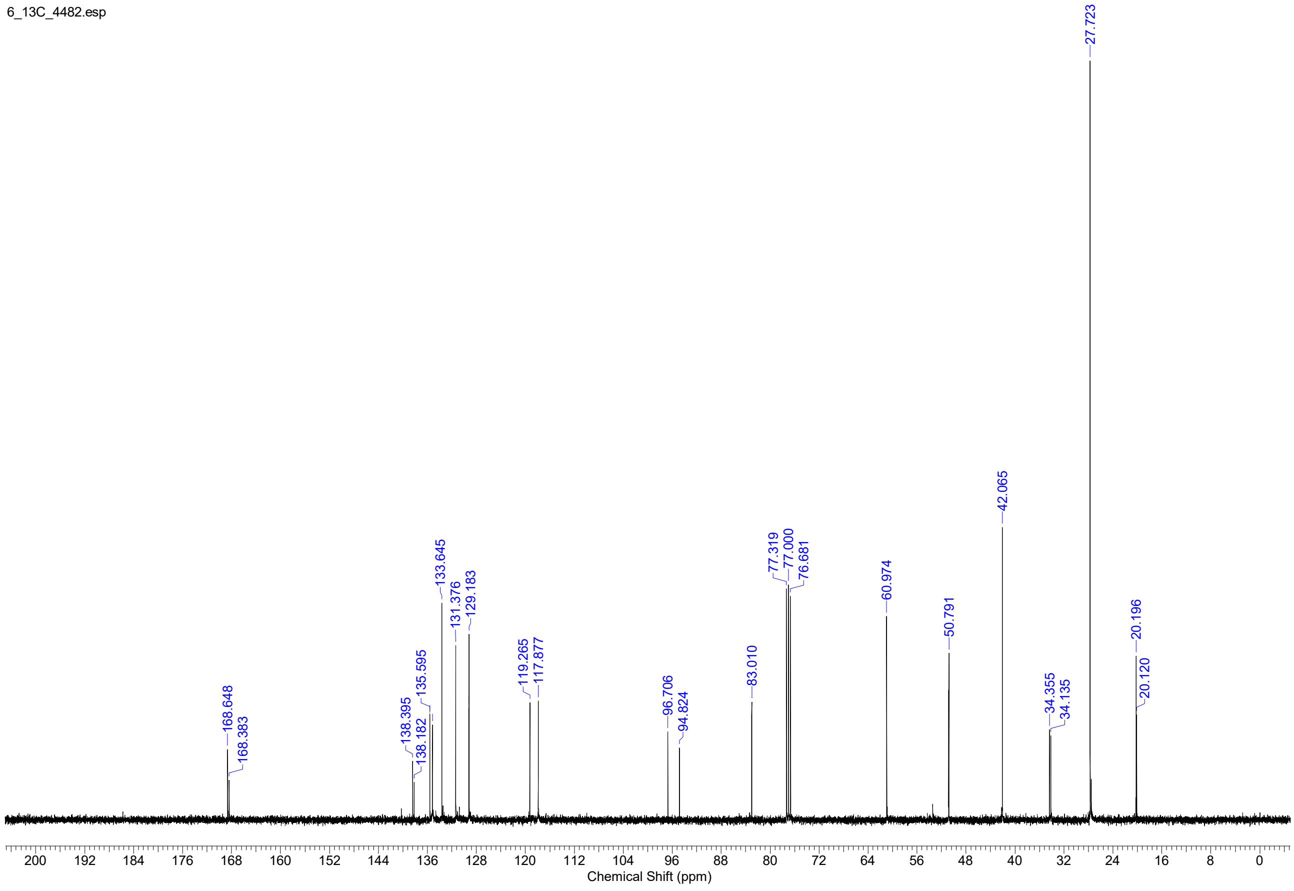

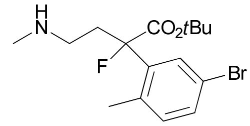

7

^1H (400 MHz, $CDCl_3$)
^{13}C (101 MHz, $CDCl_3$)

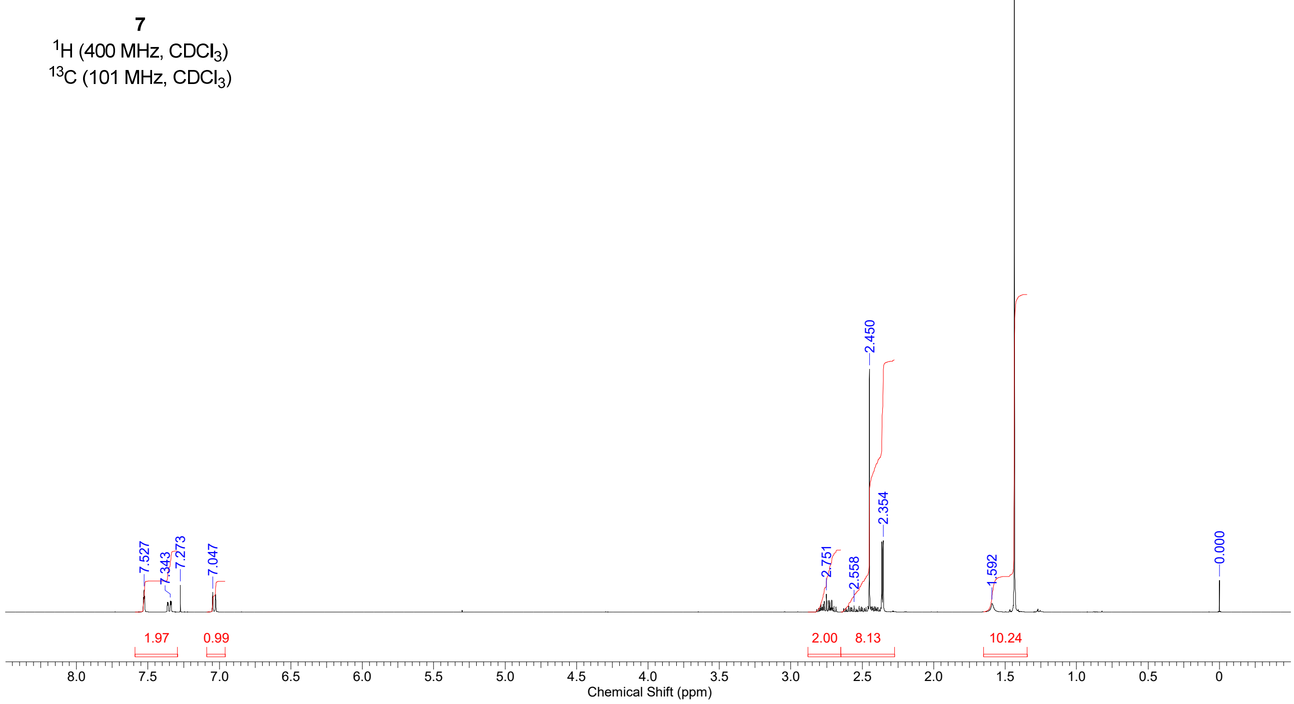

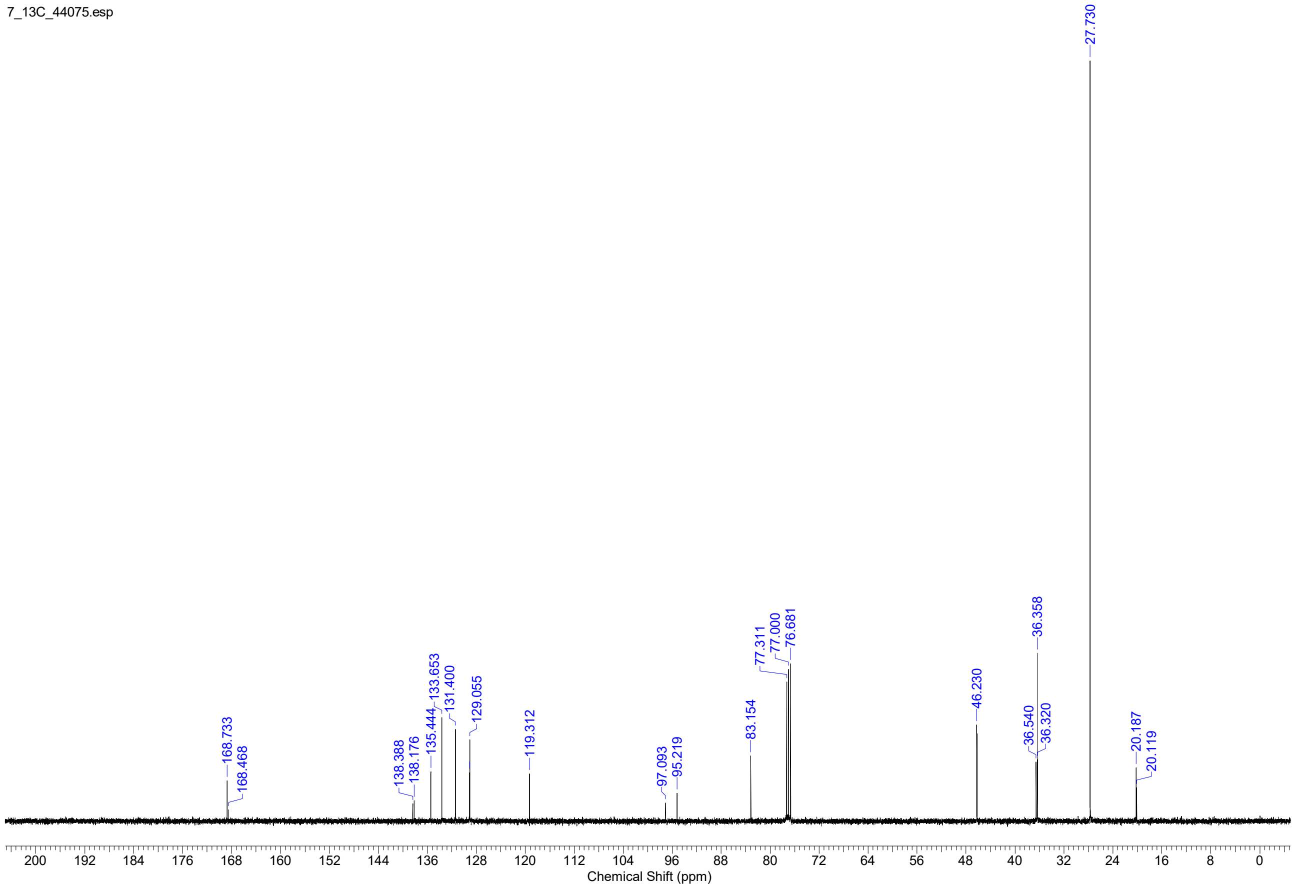

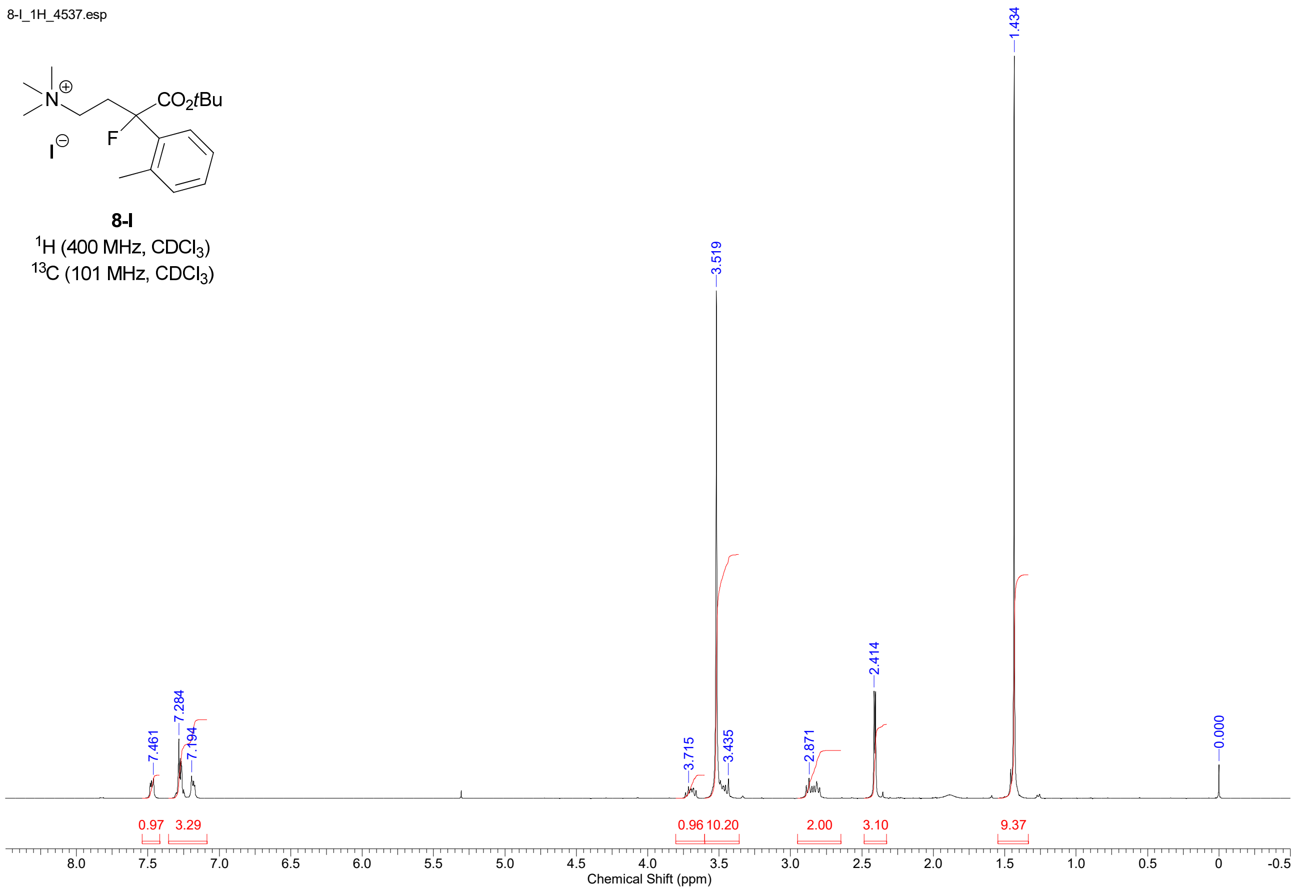

8-I

^{1}H (400 MHz, $CDCl_3$)

^{13}C (101 MHz, $CDCl_3$)

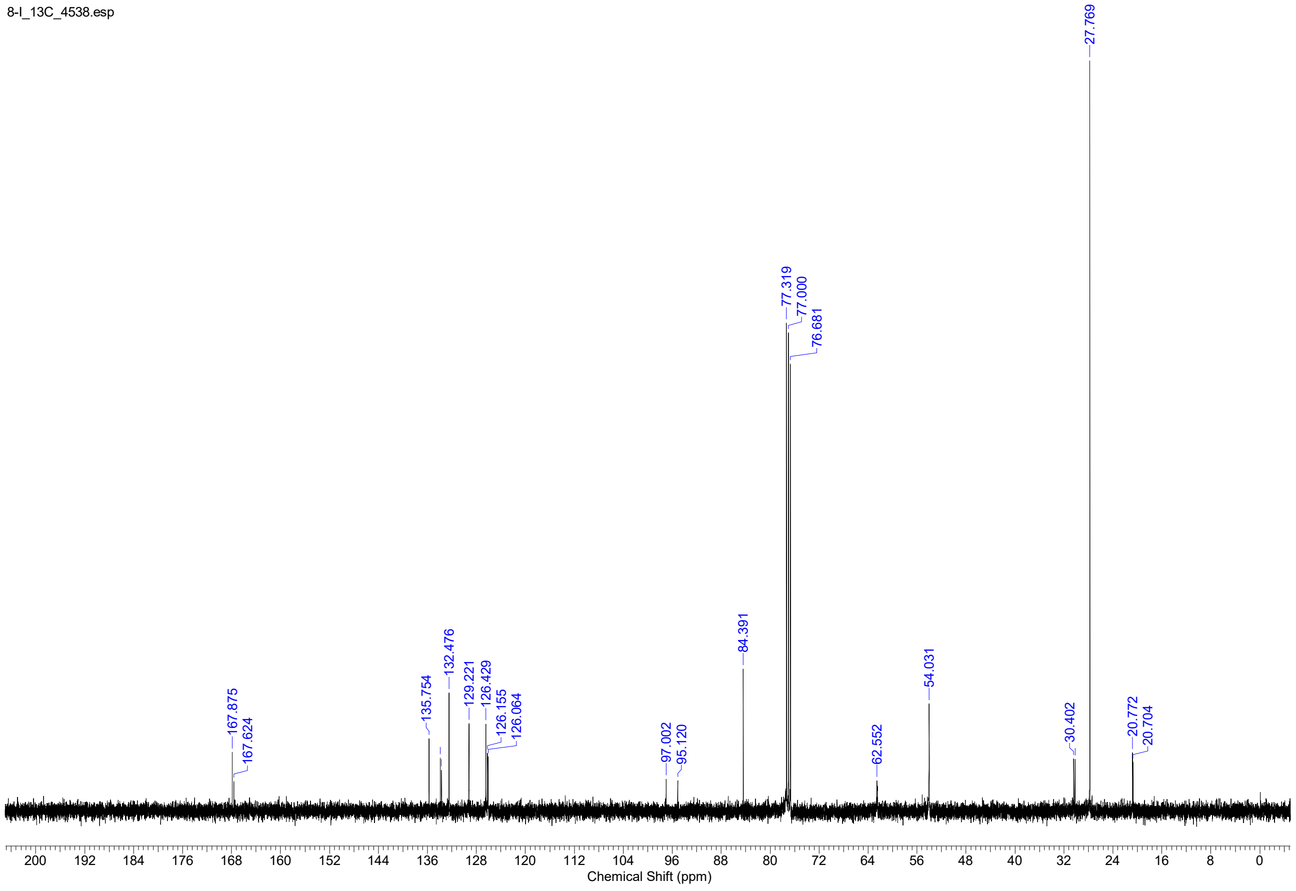

8-I_19F_4590.esp

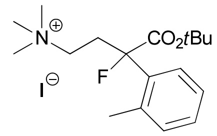

8-I

^{19}F (376 MHz, $CDCl_3$)

C_6F_6: δ –162.9 ppm

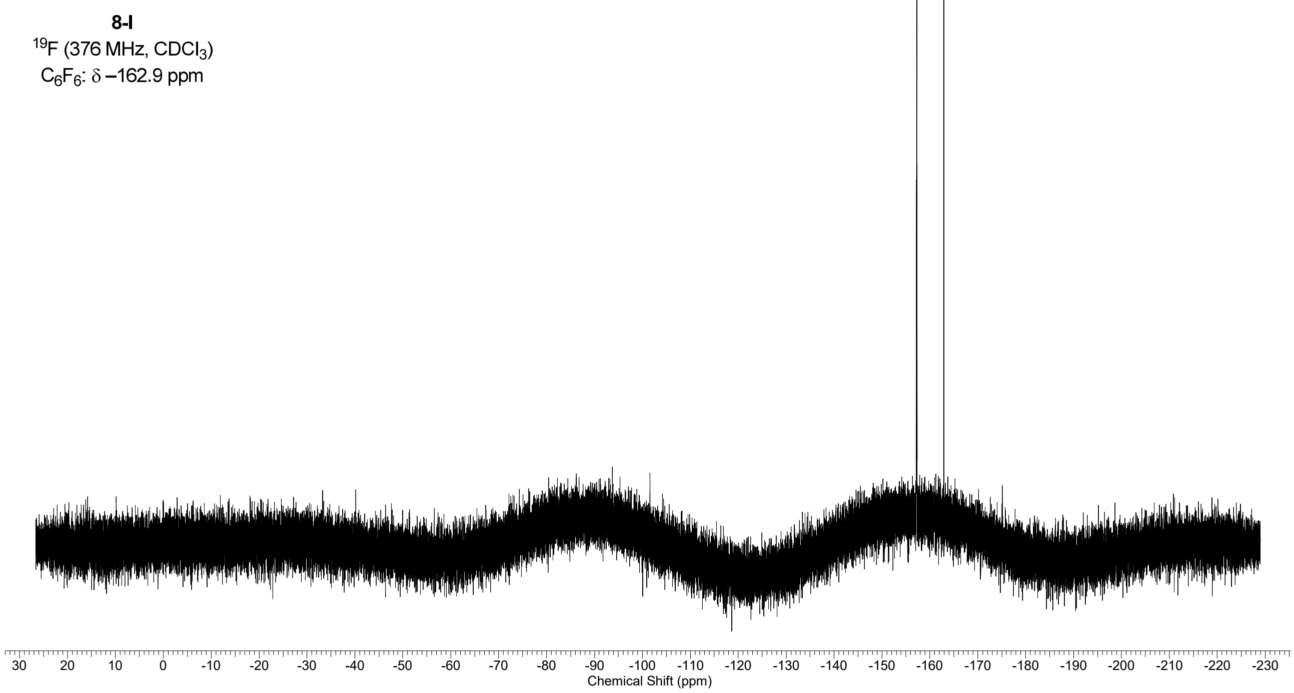

8-OTf_1H_4571.esp

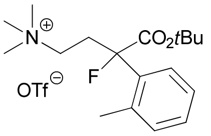

8-OTf
^{1}H (400 MHz, $CDCl_3$)
^{13}C (101 MHz, $CDCl_3$)

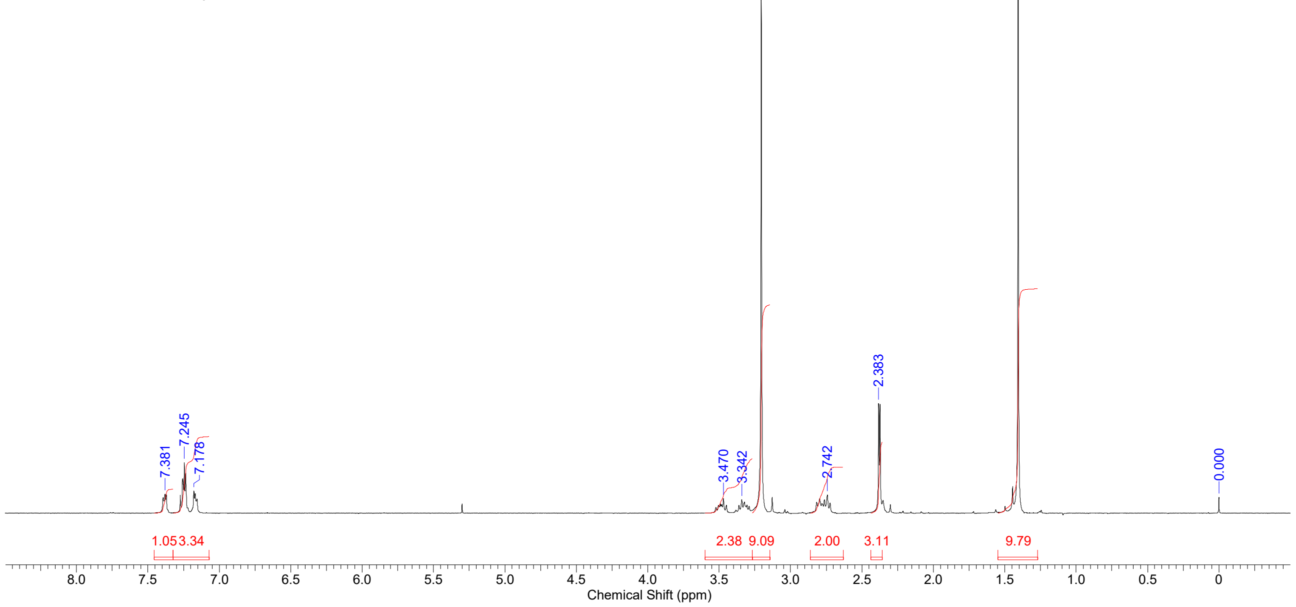

8-OTf_13C_4573.esp

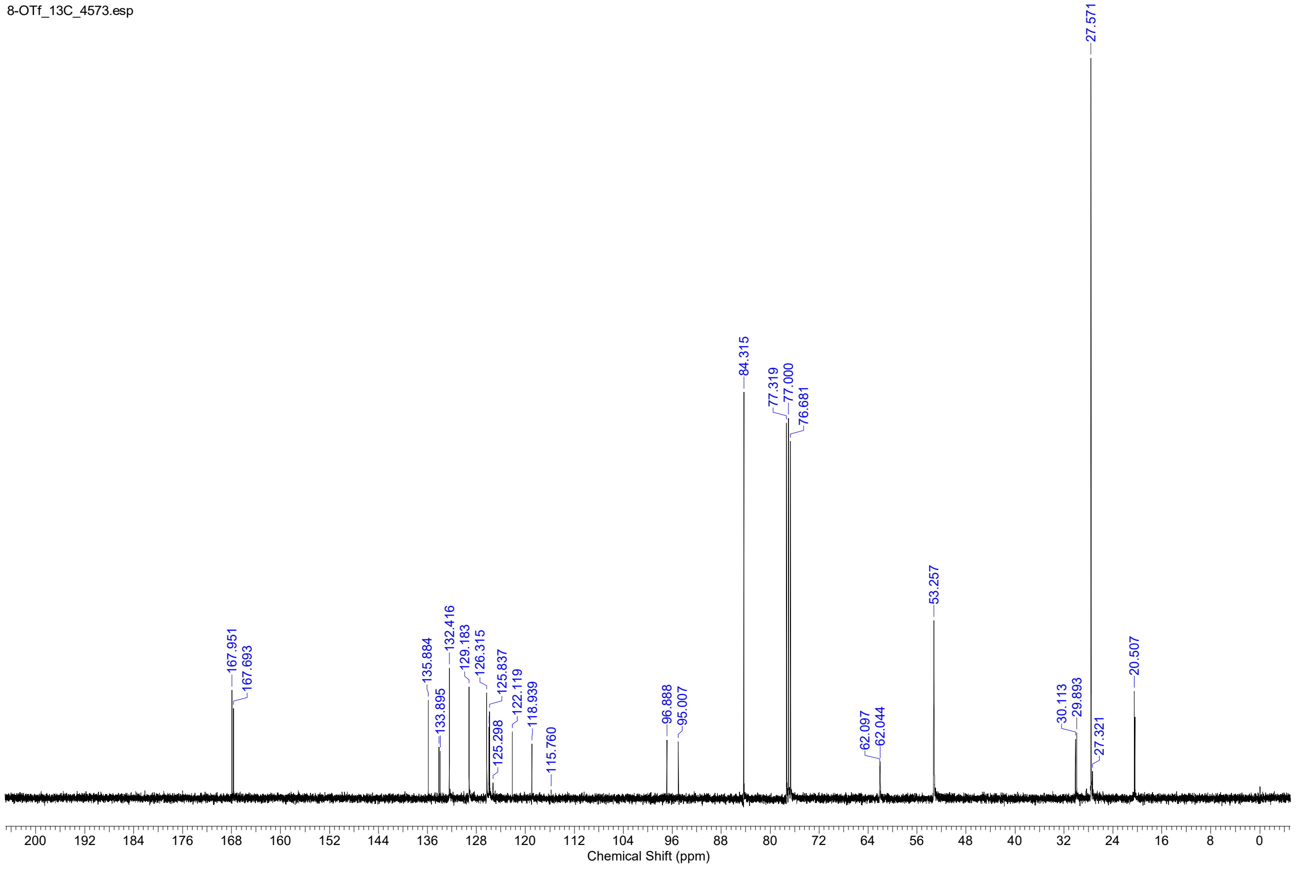

8-OTf_19F_4594.esp

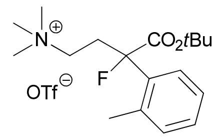

8-OTf
^{19}F (376 MHz, $CDCl_3$)
C_6F_6: δ –162.9 ppm

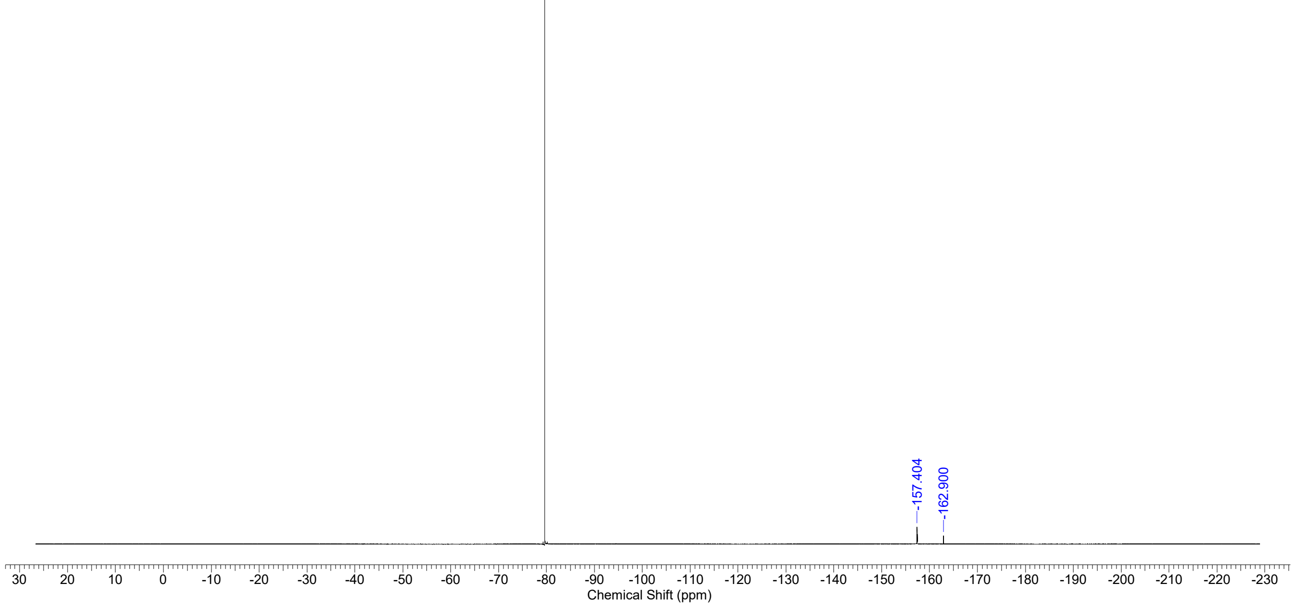

9_1H_4562.esp

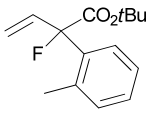

9

^{1}H (400 MHz, $CDCl_3$)

^{13}C (101 MHz, $CDCl_3$)

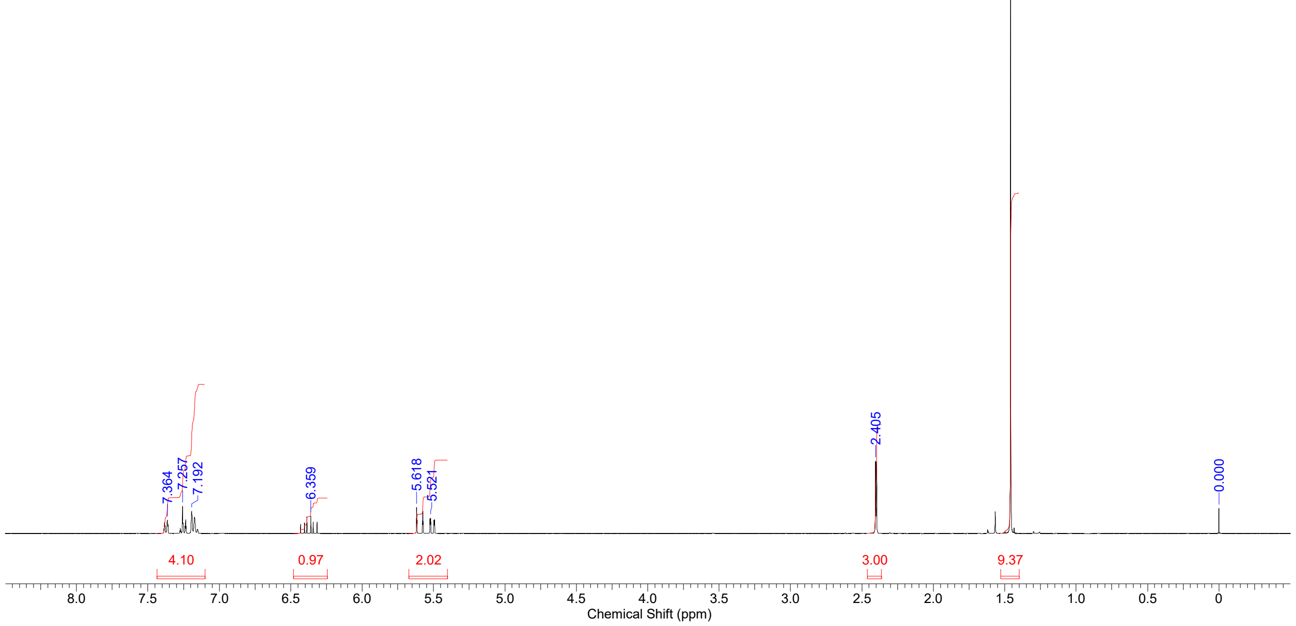

9_13C_4569-2.esp

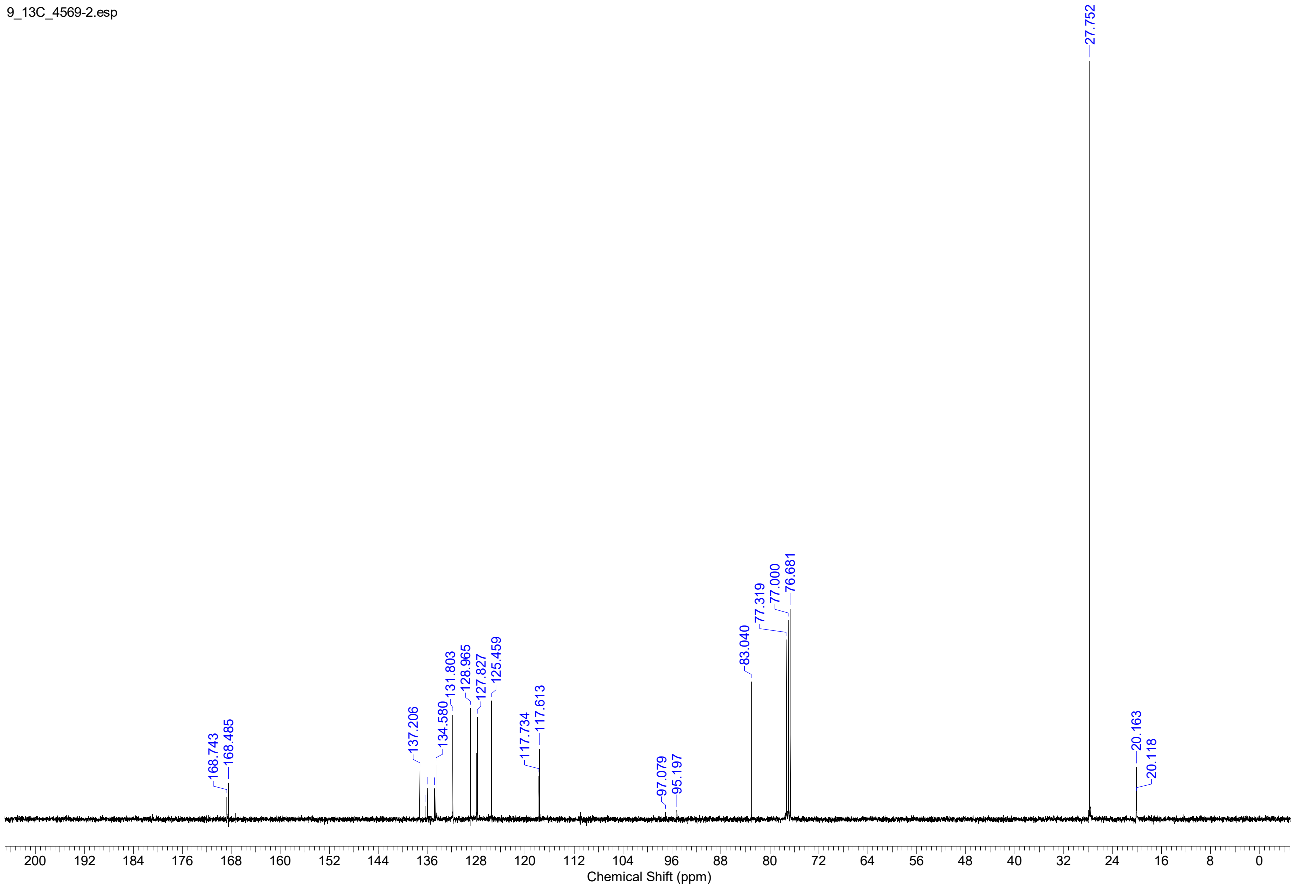

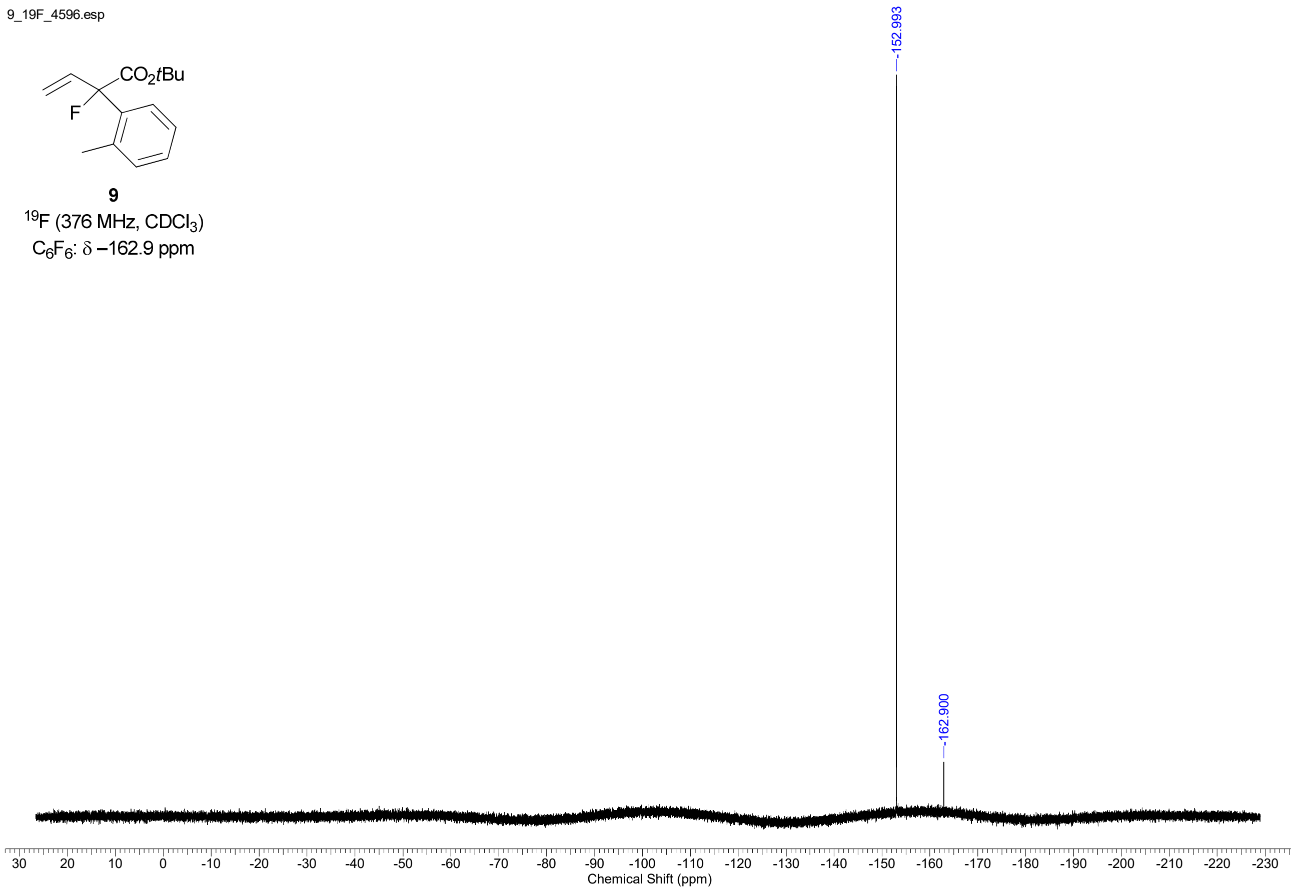
9_19F_4596.esp
CO_2tBu
F
9
^{19}F (376 MHz, $CDCl_3$)
C_6F_6: δ −162.9 ppm
-152.993
-162.900
30 20 10 0 -10 -20 -30 -40 -50 -60 -70 -80 -90 -100 -110 -120 -130 -140 -150 -160 -170 -180 -190 -200 -210 -220 -230
Chemical Shift (ppm)

10_1H_44010.esp

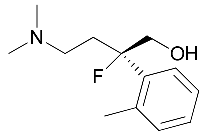

(*R*)-**10**

^{1}H (400 MHz, $CDCl_3$)

^{13}C (101 MHz, $CDCl_3$)

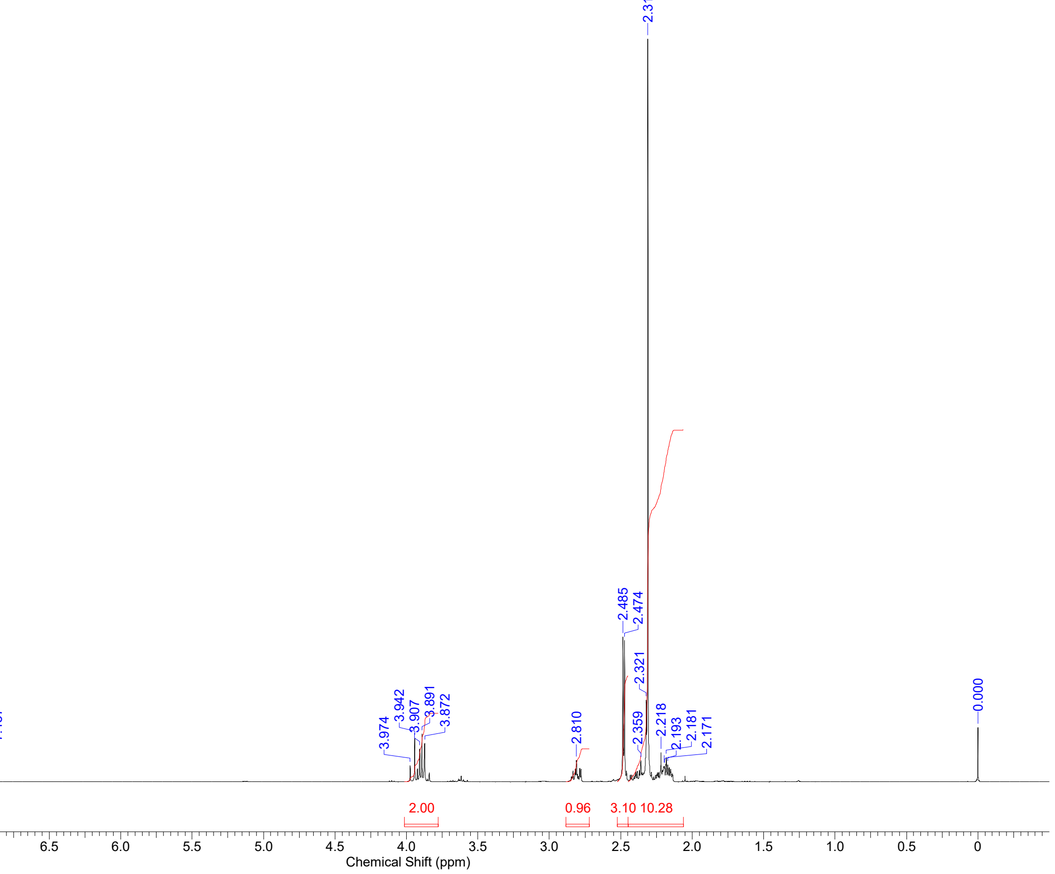

10_13C_44014.esp

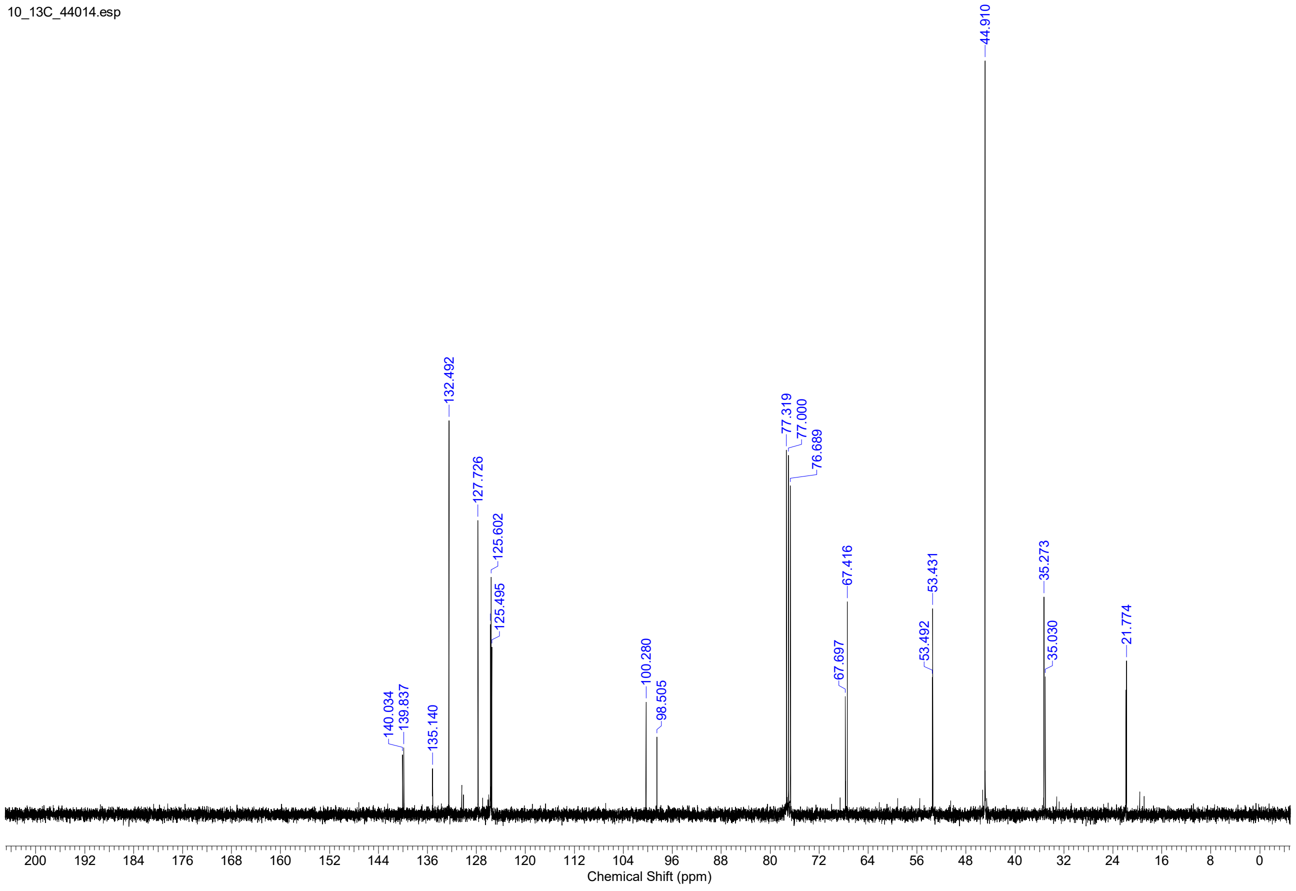

10_19F_44011.esp

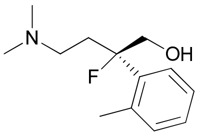

(*R*)-**10**

^{19}F (376 MHz, $CDCl_3$)

C_6F_6: δ –162.9 ppm

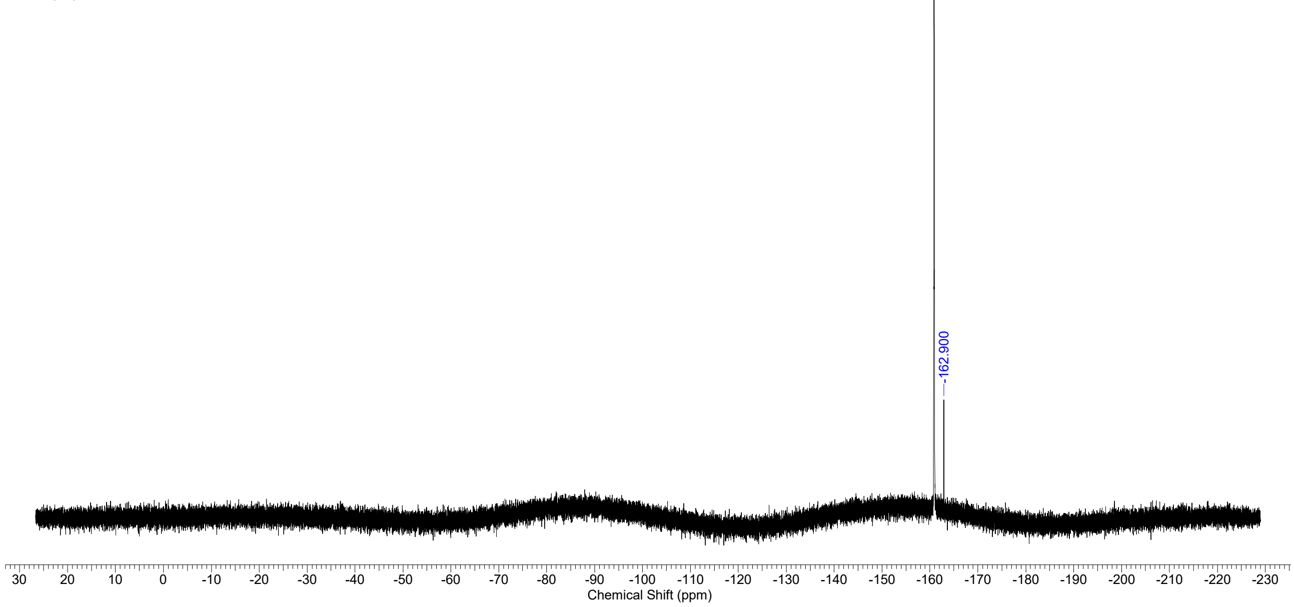

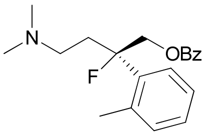

(*R*)-**11**

^{1}H (400 MHz, $CDCl_3$)

^{13}C (101 MHz, $CDCl_3$)

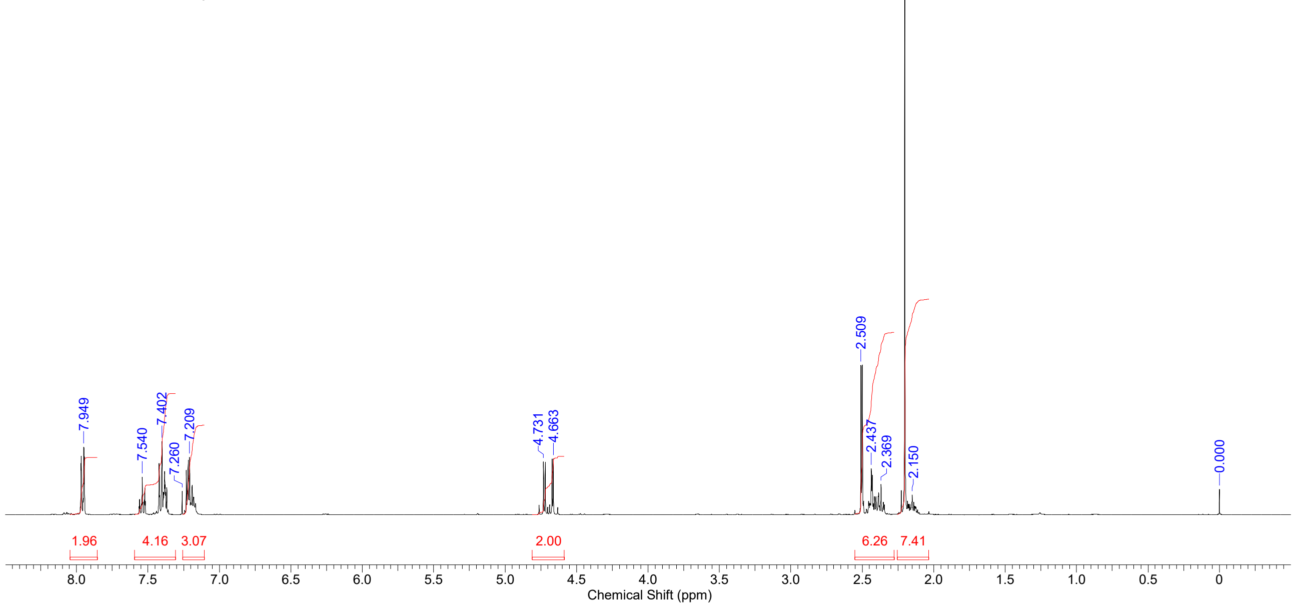

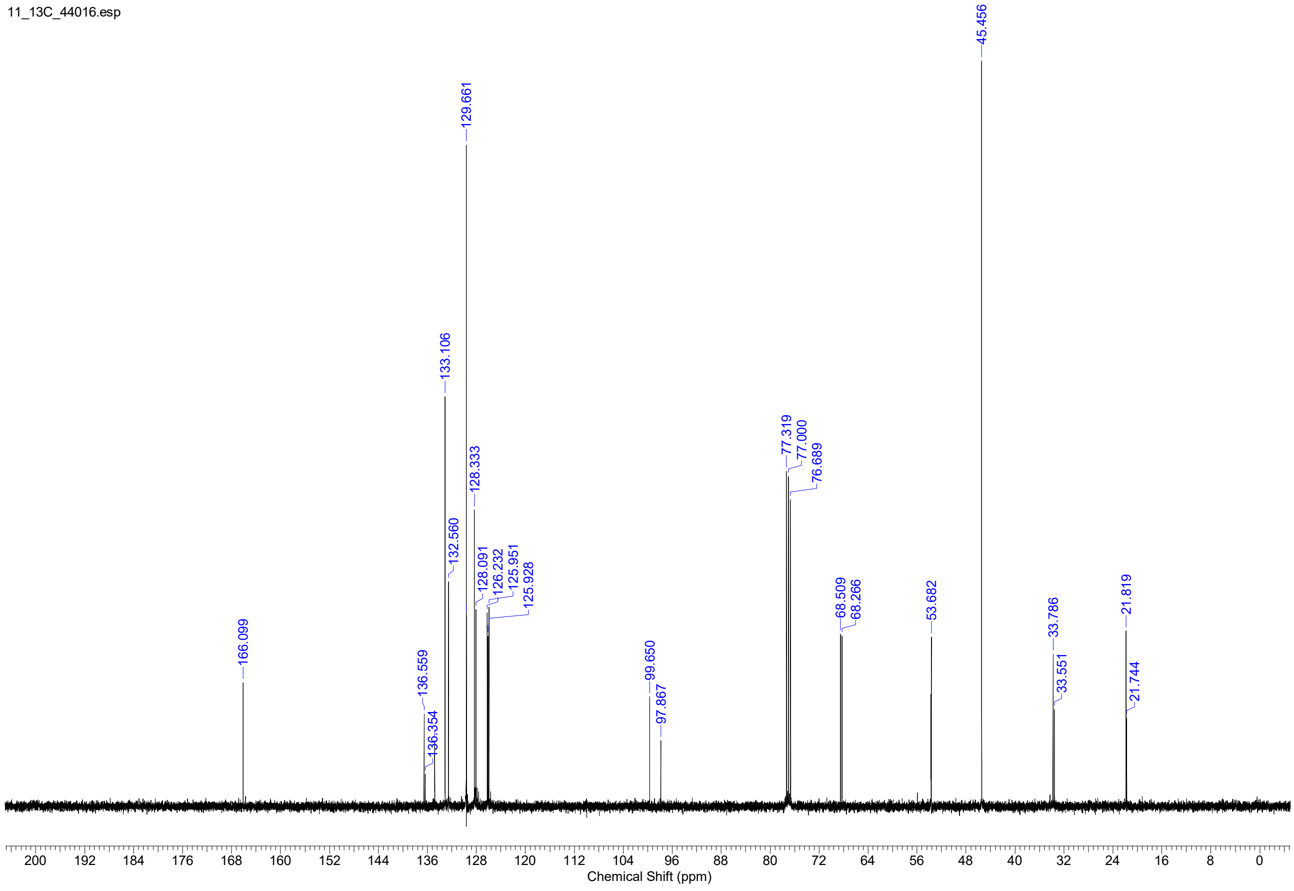

11_19F_44017.esp

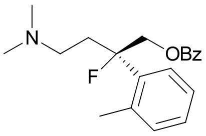

(*R*)-**11**
^{19}F (376 MHz, $CDCl_3$)
C_6F_6: δ –162.9 ppm

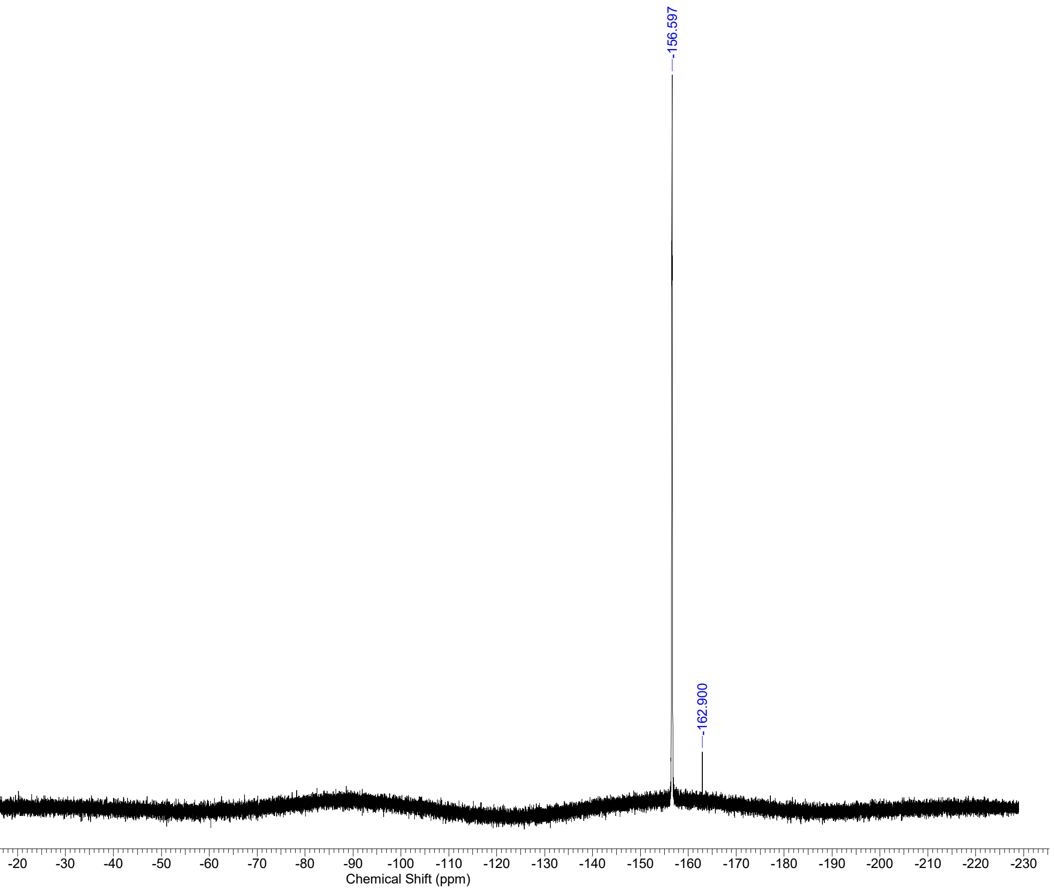

12_1H_n4317.esp

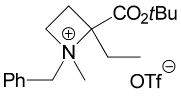

12

^1H (400 MHz, $CDCl_3$)

^{13}C (101 MHz, $CDCl_3$)

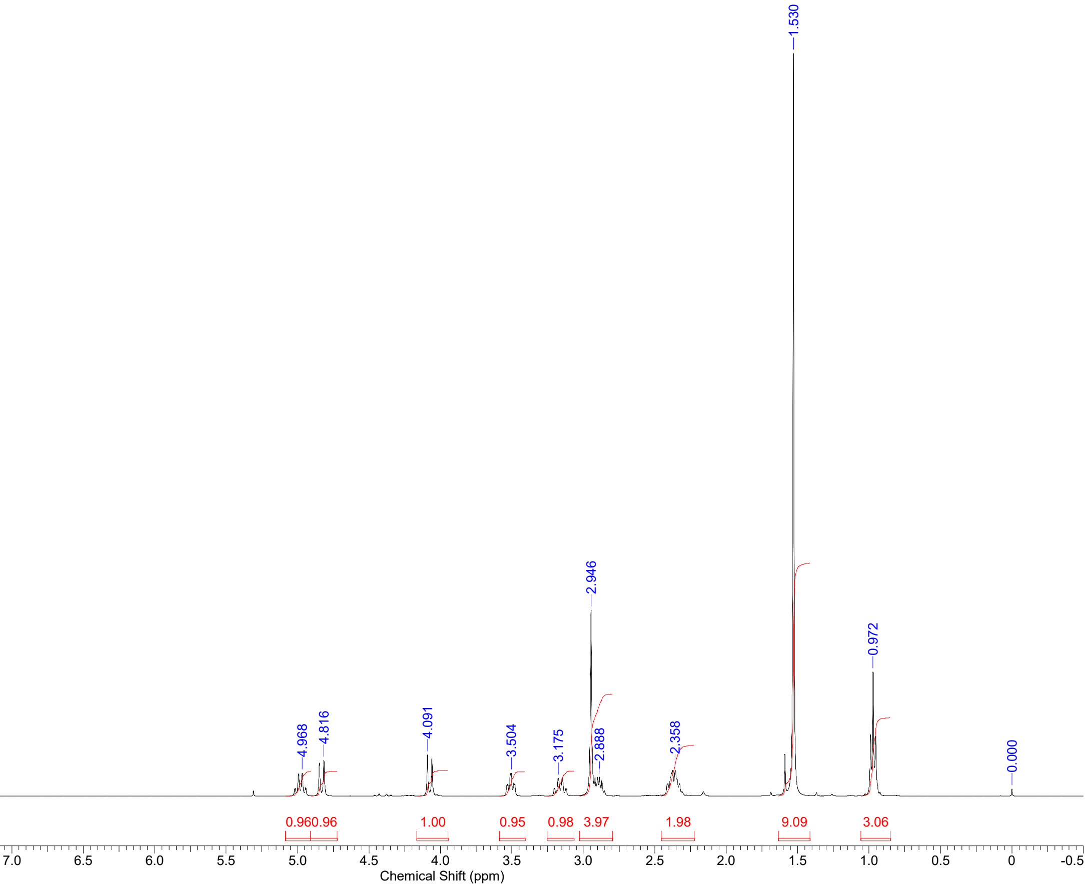

12_13C_n4318.esp

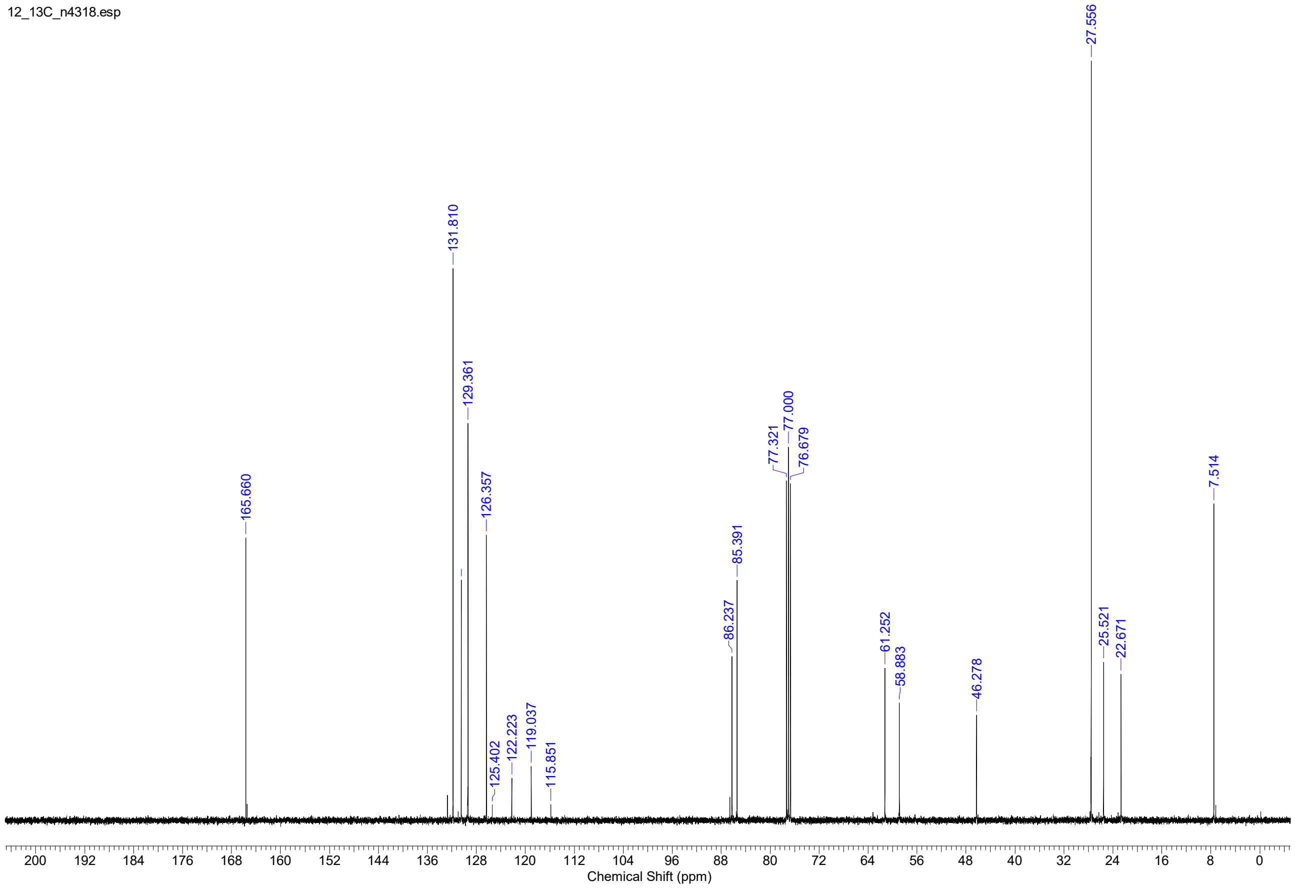

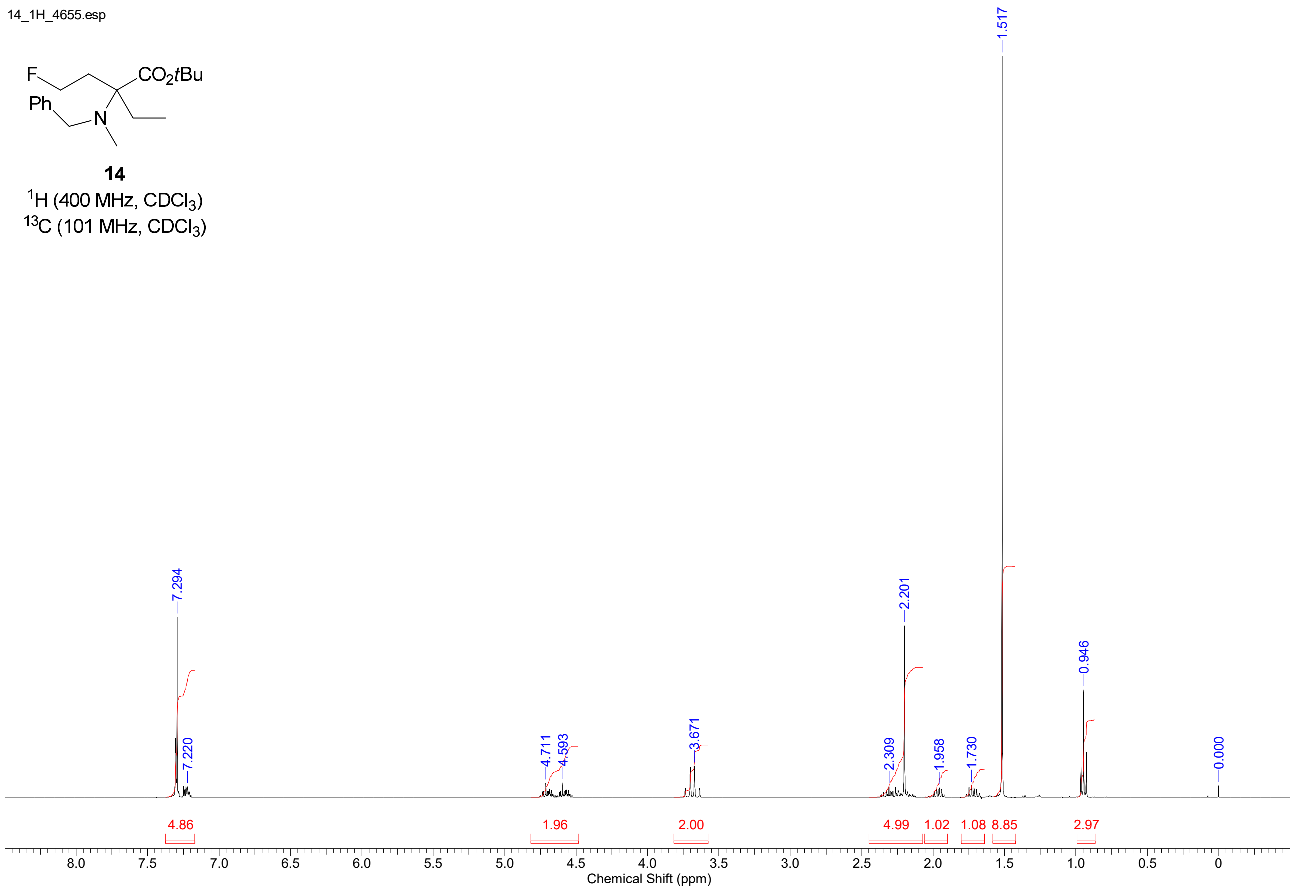
14_1H_4655.esp
F
CO2tBu
Ph
N
14
1H (400 MHz, CDCl3)
13C (101 MHz, CDCl3)
7.294
7.220
4.711
4.593
3.671
2.309
2.201
1.958
1.730
1.517
0.946
0.000
4.86
1.96
2.00
4.99
1.02
1.08
8.85
2.97
Chemical Shift (ppm)

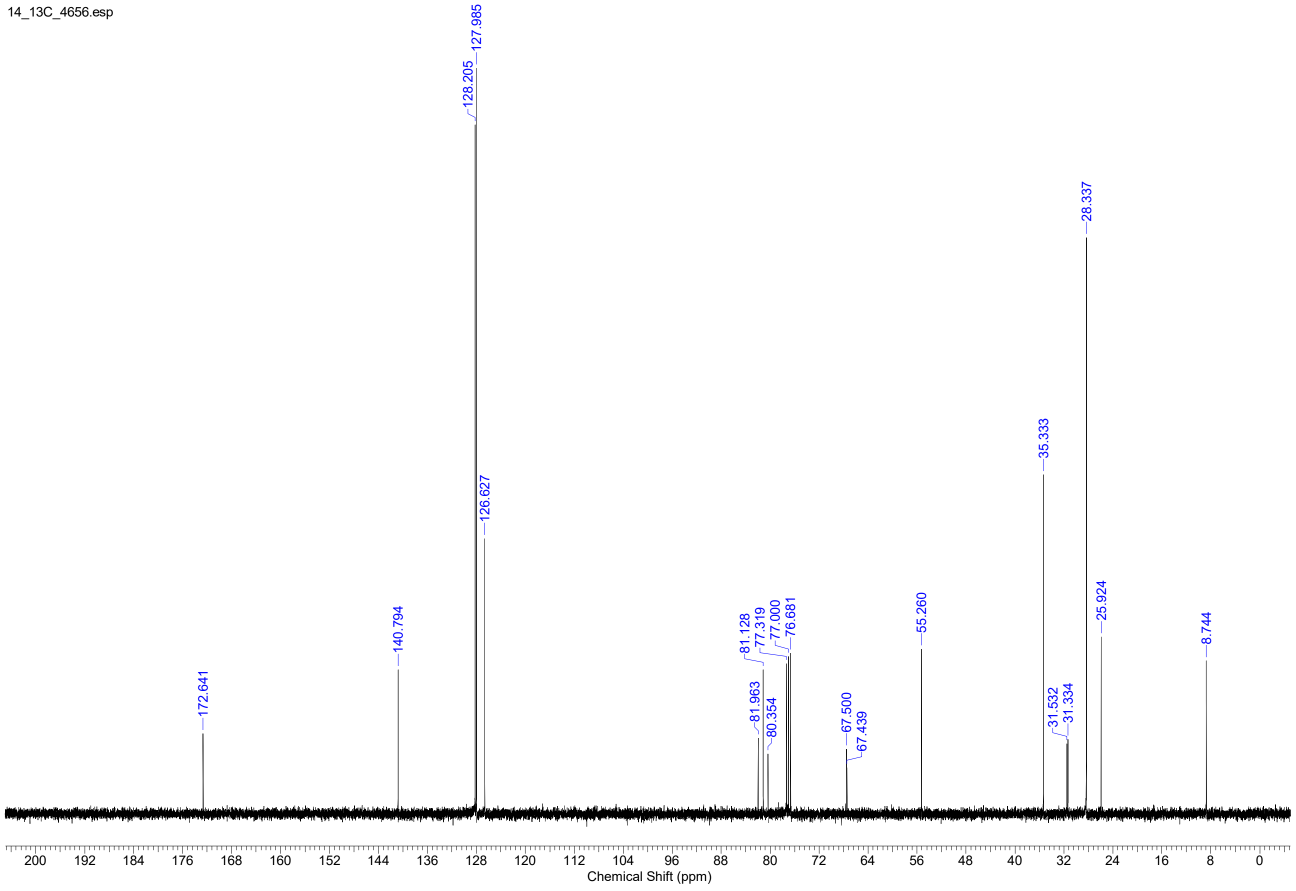
14_13C_4656.esp
172.641
140.794
128.205
127.985
126.627
81.963
81.128
80.354
77.319
77.000
76.681
67.500
67.439
55.260
35.333
31.532
31.334
28.337
25.924
8.744
Chemical Shift (ppm)

14_19F_4658.esp

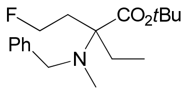

14

^{19}F (376 MHz, $CDCl_3$)

C_6F_6: δ –162.9 ppm

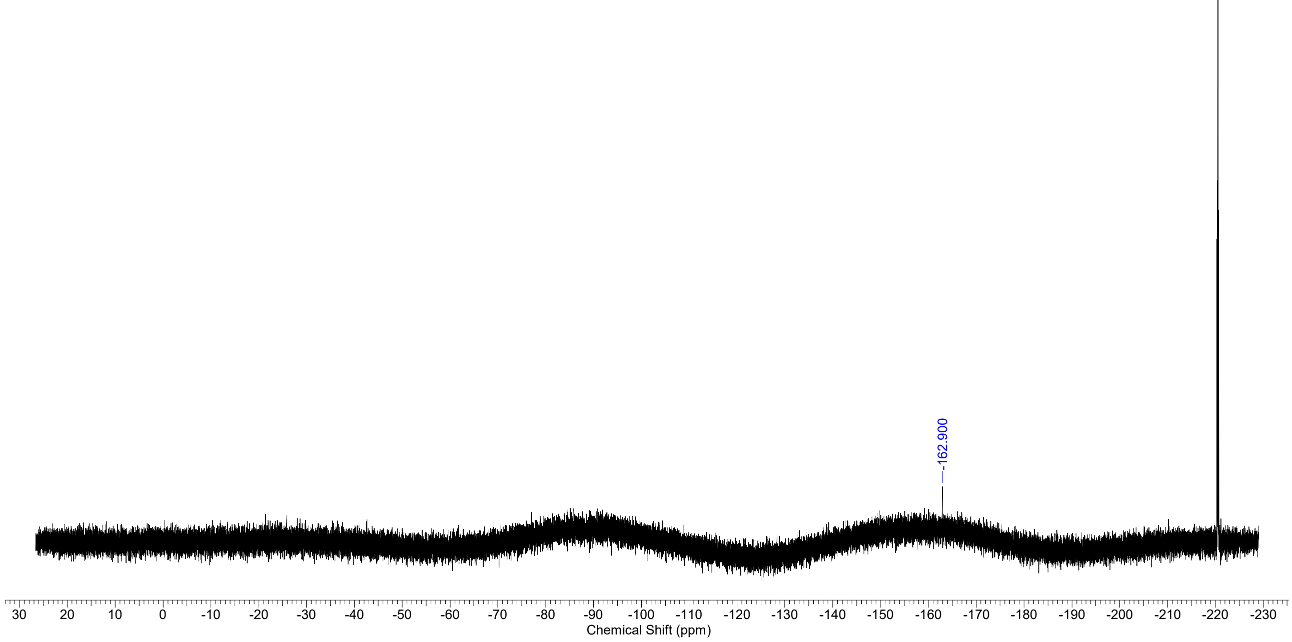

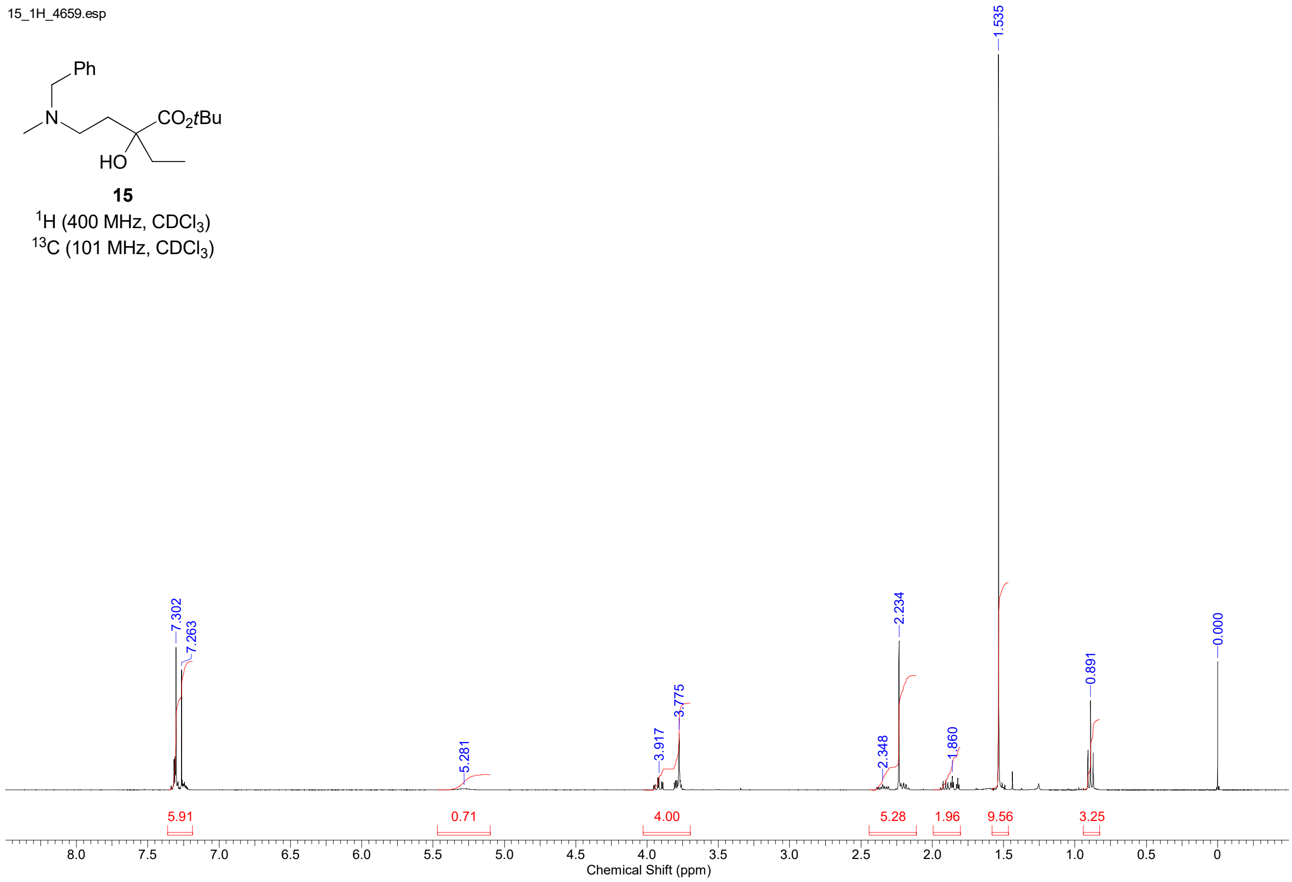
15_1H_4659.esp
Ph
N
CO2tBu
HO
15
1H (400 MHz, CDCl3)
13C (101 MHz, CDCl3)
7.302
7.263
5.281
3.917
3.775
2.348
2.234
1.860
1.535
0.891
0.000
5.91
0.71
4.00
5.28
1.96
9.56
3.25
8.0 7.5 7.0 6.5 6.0 5.5 5.0 4.5 4.0 3.5 3.0 2.5 2.0 1.5 1.0 0.5 0
Chemical Shift (ppm)

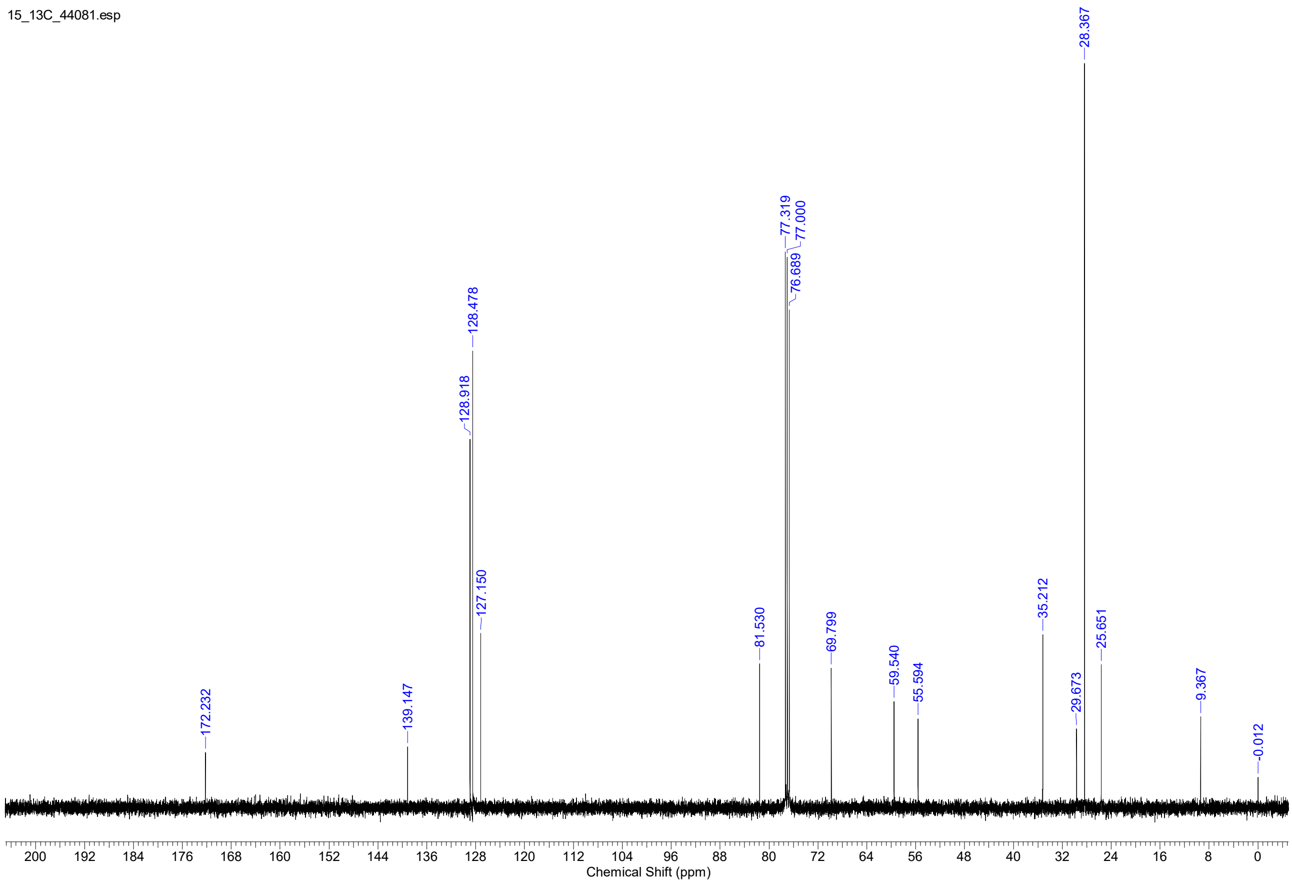
15_13C_44081.esp
172.232
139.147
128.918
128.478
127.150
81.530
77.319
77.000
76.689
69.799
59.540
55.594
35.212
29.673
28.367
25.651
9.367
-0.012
200 192 184 176 168 160 152 144 136 128 120 112 104 96 88 80 72 64 56 48 40 32 24 16 8 0
Chemical Shift (ppm)
